# Development of High-Affinity CHD1 Chromodomain Inhibitors

**DOI:** 10.1021/acs.jmedchem.5c03690

**Published:** 2026-05-05

**Authors:** Holger Greschik, Florian Friedrich, Ludwig Seifert, Farnoush Mousavizadeh, Francesco Fiorentino, Johannes Walz, Lin Zhang, Jianyu Li, Emanuele Fabbrizi, Stefano Tomassi, Farhad Panahi, Niklas Papenkordt, Silas L. Wurnig, Johannes Osterroth, Anna M. Strasser, Jan Ruprecht, Aurélien F. A. Moumbock, Martin Hügle, Manuela Sum, Ling Peng, Sheng Wang, Adina A. Baniahmad, Laura Pulido-Cortés, H. Th. Marc Timmers, Ralf Flaig, Eric Metzger, Bernhard Breit, Oliver Einsle, Stefan Günther, Dante Rotili, Antonello Mai, Roland Schüle, Manfred Jung

**Affiliations:** † Department of Urology and Center for Clinical Research, University Freiburg Medical Center, Breisacher Str. 66, 79106 Freiburg, Germany; ‡ Institute of Pharmaceutical Sciences, 9174 University of Freiburg, Albertstr. 25, 79104 Freiburg, Germany; § Institute of Organic Chemistry, University of Freiburg, Albertstr. 21, 70104 Freiburg, Germany; ∥ Department of Biochemical Sciences, 9311 Sapienza University of Rome, P.le Aldo Moro 5, 00185 Rome, Italy; ⊥ Institute of Biochemistry, University of Freiburg, 79104 Freiburg, Germany; # Institute of Pharmaceutical Sciences, University of Freiburg, Hermann-Herder-Str. 9, 79104 Freiburg, Germany; ∇ Department of Drug Chemistry and Technologies, Sapienza University of Rome, P.le Aldo Moro 5, 00185 Rome, Italy; ○ Department of Life Science, Health and Health Professions, 207131 LINK Campus University, Via del Casale di San Pio V, 44, CAP 00165 Rome, Italy; ◆ German Cancer Consortium (DKTK), Partner Site Freiburg, 14879 University Medical Center Freiburg, Breisacher Str. 66, 79106 Freiburg, Germany; ¶ 120796 Diamond Light Source Ltd, Harwell Science & Innovation Campus, Didcot, Oxfordshire OX11 0DE, United Kingdom; †† Research Complex at Harwell, Rutherford Appleton Laboratory, Didcot OX11 0FA, United Kingdom; ‡‡ Department of Science, Roma Tre University of Rome, Viale Guglielmo Marconi 446, 00146 Rome, Italy

## Abstract

The chromatin remodeler CHD1, a regulator of gene activity and potential drug target in prostate cancer (PCa), contains a tandem chromodomain (tCD) binding histone H3 trimethylated at lysine 4 (H3K4me3). We developed the first submicromolar inhibitors (**2n** and **2s**) that target the H3K4me3 binding site of the CHD1 tCD with *K*_d_ values of 0.15 μM and 0.14 μM, respectively. Co-crystal structures of these quinoline-based compounds revealed aromatic cage interactions and extended ligand contacts in other parts of the H3K4me3 peptide pocket as the main determinants of high-affinity ligand binding. **2n** and **2s** engage endogenous CHD1 in cell lysates or the exogenous CHD1 tCD in cells. Furthermore, we provide evidence for selectivity against a panel of methyl-lysine readers and epigenetic enzymes as well as impairment of PCa cell viability. Due to their high potency and defined binding mode, our ligands offer new directions for further optimization.

## Graphical abstract

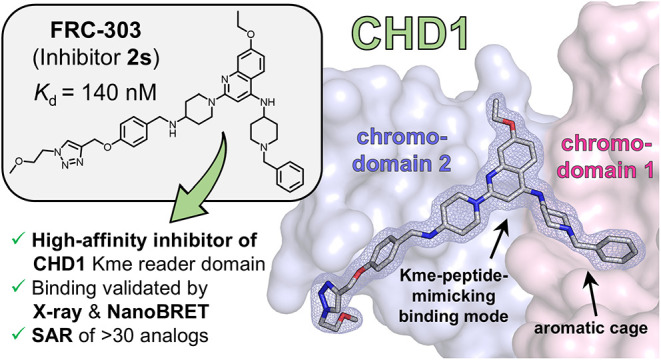

## Introduction

The chromodomain helicase DNA-binding (CHD) family of ATP-dependent chromatin remodelers consists of nine members (CHD1–9) exerting multiple functions during development and in adulthood.^1–3^ All family members harbor a tandem chromodomain (tCD), which consists of two chromo “subdomains” forming a functional unit, a sucrose nonfermentable (SNF)­2-like ATPase/helicase domain, and distinct additional domains. The CHD1 tCD was shown to bind H3K4me3,^4^ lysine-specific demethylase (LSD)­1 (also termed KDM1A) dimethylated at K114,^5^ and the viral influenza nonstructural protein (NS)­1 di- or trimethylated at K229.^6^ As in the case of other methyl-lysine (Kme) reader domains including Tudor, malignant brain tumor (MBT), proline–tryptophan–tryptophan–proline (PWWP), tryptophan−aspartate (WD)­40 repeat, and plant homeodomain (PHD), the methylated residues bind to a so-called “aromatic cage” formed by aromatic amino acids.^7^ Kme reader domains have become attractive targets for drug development,^8–11^ and several inhibitors have been developed, selected examples of which are depicted in Figure 1.^12–22^

**Figure 1:**
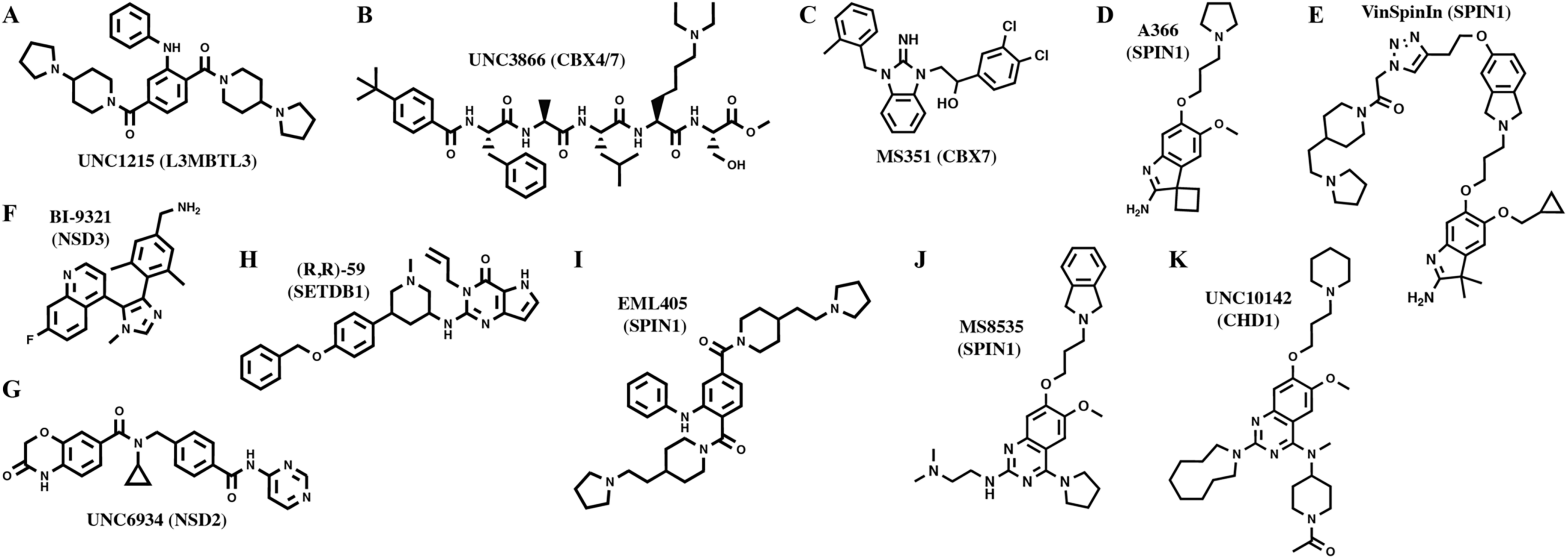
Selected small-molecule inhibitors of Kme reader proteins. (A) **UNC1215** (MBT domain of L3MBTL3),^12^ (B) **UNC3866** (chromodomain of CBX4/7),^13^ (C) **MS351** (chromodomain of CBX7),^14^ (D) **A366** (Tudor domain 2 of SPIN1),^15^ (E) **VinSpinIn** (Tudor domains 1 and 2 of SPIN1),^16^ (F) **BI-9321** (PWWP domain of NSD3),^17^ (G) **UNC6934** (PWWP domain of NSD2),^18^ (H) **(R,R)-59** (Tudor domain of SETDB1),^19^ (I) **EML405** (Tudor domain 2 of SPIN1),^20^ (J) **MS8535** (Tudor domain 2 of SPIN1),^21^ and (K) **UNC10142** (CHD1 tCD).^22^

CHD1 has been implicated in diseases including cancer.^1–3,23,24^ Mutation or deletion of *CHD1*, for example, has been observed in about 8 to 10% of prostate cancer (PCa) cases and often co-occurs with other genomic alterations,^23,25–29^ which suggests that the protein can act as a tumor suppressor. In contrast, CHD1 was observed to increase oncogenic gene rearrangements at androgen receptor target genes in PCa upon interaction with LSD1-K114me2 ^5^ hinting at tumor-promoting CHD1 activity. Furthermore, survival of phosphatase and tensin homologue (PTEN)-deficient PCa cells was reported to depend on the presence of CHD1, providing an example for PTEN/CHD1 synthetic essentiality.^30^ In prostate tumors with PTEN loss, CHD1 contributes to an immunosuppressive tumor microenvironment.^31^ Finally, CHD1 was observed to promote sensitivity of PCa cells to aurora kinase A inhibitors,^32^ and CHD1 loss sensitizes PCa cell lines to DNA-damaging therapy^33^ as well as poly­(ADP-ribose)­polymerase inhibitors.^34^ Together, these reports suggest that CHD1 may be a therapeutic target in certain subtypes of PCa.

While CHD1 has been regarded as a potential target in cancer, in a computational study, the Kme-binding cleft of the CHD1 tCD was characterized as relatively shallow and thus challenging as drug target.^35^ A H3K4me3 peptide, mimicking the natural histone ligand, was reported to bind to the CHD1 tCD in a fluorescence polarization (FP)-based assay with a dissociation constant (*K*_d_) of 5 μM.^4^ Notably, structural data showed that the H3K4me3 peptide binds across the interface of the CHD1 chromo subdomains,^4^ a feature that may be exploited for the development of high-affinity inhibitors. Recently, a first inhibitor (**UNC10142**; Figure 1K) targeting the CHD1 tCD with moderate affinity [*K*_d_ = 4.3 μM determined by isothermal titration calorimetry (ITC); IC_50_ = 1.7 μM determined by time-resolved Förster resonance energy transfer (TR-FRET)] was reported.^22^ The ligand is based on a quinazoline scaffold that has also been used, for example, in the case of inhibitors of the Kme reader SPIN1 [e.g., **MS8535** (Figure 1J)].^21^ Furthermore, the quinazoline scaffold is found in inhibitors of nonreader proteins, including the histone demethylase LSD1 (compound **29**)^36^ and the histone methyltransferases SETD8 (**UNC0379**)^37^ and GLP/G9a (also known as EHMT1/2) (**BIX01294**)^38^ (Figure S1A–C).

In this study, the second report on CHD1 tCD inhibitors, we identified alkyloxy quinazolines and alkyloxy quinolines as high-affinity binders. Our most potent quinoline-based compounds, **2n** (**FRC-222**) and **2s** (**FRC-303**), exhibit *K*_d_ values determined by ITC of 0.15 μM and 0.14 μM, respectively. Compared to **UNC10142**, our ligands exhibit aromatic cage binding with a *N*-benzylpiperidine moiety mimicking Kme interactions and form additional contacts in other parts of the peptide binding pocket. Thus, we identified the first submicromolar CHD1 tCD inhibitors and present a comprehensive structure–activity relationship (SAR) analysis explaining the determinants of high-affinity binding.

## Results and Discussion

### Identification of Quinazolines as CHD1 tCD Ligands

To identify inhibitors of the CHD1 tCD, we first established a FRET-based assay system using purified, recombinant green fluorescent protein (GFP)–CHD1 tCD fusion protein and 5-carboxytetramethylrhodamine (TAMRA)-labeled H3K4me3 peptide (Figure S1D). In this system, we determined an apparent *K*_d_,_app_ of 0.49 μM for TAMRA-H3K4me3/GFP-CHD1 tCD interaction (Figure S1E). H3K4me or LSD1-K114me peptides with distinct methylation states at K4 or K114, respectively, inhibited the FRET signal with half-maximal inhibition constants (IC_50_) ranging from 20 μM to about 4 mM (Figure S1F–S1I). H3K4me2/3 and LSD1-K114me2/3 peptides were more effective inhibitors than monomethylated, unmethylated or mutant peptides, in which K4 or K114 was replaced with alanine (Figure S1F–S1I). These results are in full accordance with previous observations for H3K4 and LSD1 peptide binding to the CHD1 tCD.^4,5^ Using our newly developed FRET assay, we subsequently screened an in-house, targeted library containing Kme mimics. Among other potential hits, we identified compound **1a** with an IC_50_ of 42 μM (Figure 2). By ITC, we determined a *K*_d_ of 20 μM (Figures 2 and S2). **1a** consists of a 6,7-dimethoxy-quinazoline scaffold with two potential Kme-mimicking moieties, *N* ^3^ *,N* ^3^-dimethylpropane-1,3-diamine (DPD) at C2 and (*N*-benzylpiperidin-4-yl)­amine (BPA) at C4.

**Figure 2:**
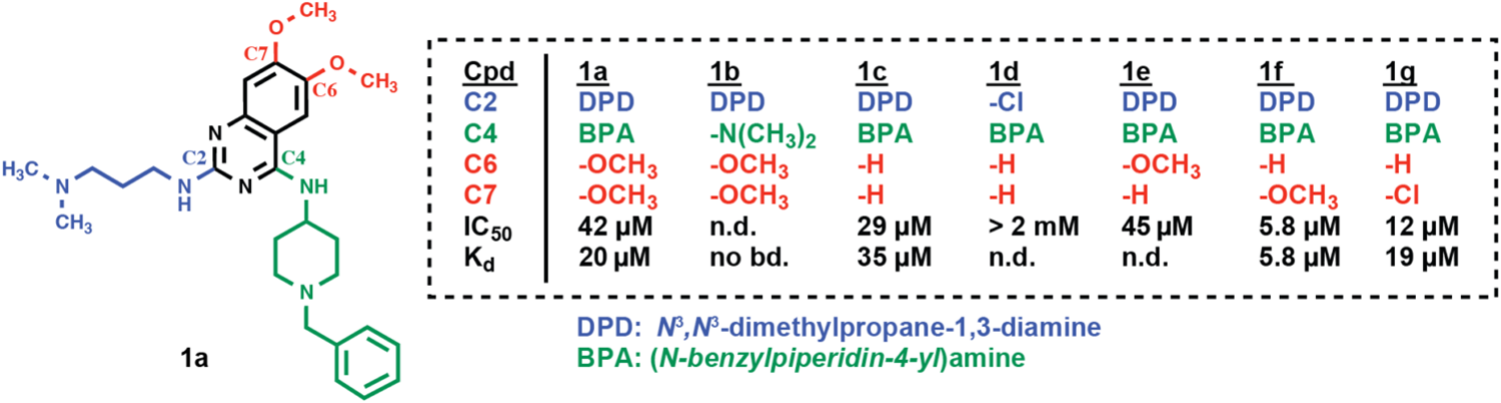
Identification of an initial hit and truncation at C2, C4, C6, and C7 of the quinazoline scaffold (compounds **1b**–**1g**). IC_50_ and *K*_d_ values for compound binding to the CHD1 tCD were determined by FRET and ITC, respectively. Original ITC data are shown in Figure S2. (Cpd: compound; no bd.: no binding; n.d.: not determined.).

To evaluate whether the substituents at positions C2, C4, C6, or C7 of the quinazoline core were required for binding, we tested a series of truncated compounds (**1b**–**1g**) (Figure 2). While truncations at C2 and C4 strongly reduced or abolished binding (**1b**, **1d**), the removal of both methoxy groups from C6 and C7 had no clear effect on the affinity (**1c**). Similarly, a **1a** derivative harboring only a methoxy group at C6 (**1e**) did not exhibit altered binding (IC_50_ = 45 μM), whereas **1f** with only a methoxy group at C7 bound to the CHD1 tCD with the highest affinity at that stage of the project (IC_50_ = 5.8 μM, *K*_d_ = 5.8 μM). Replacement of the 7-methoxy with a chloro substituent reduced binding (**1g**, IC_50_ = 12 μM, *K*_d_ = 19 μM). These results identified **1f** as the best hit for further optimization and defined a minimal 7-methoxyquinazoline scaffold with two potential Kme mimics as important determinants of CHD1 tCD binding.

### Optimization of Substituents and Transition to a Quinoline Scaffold

Next, we tried to optimize **1f** by replacing the (*N*-benzylpiperidin-4-yl)­amine moiety at C4 with other substituents (**1h**–**1n**) (Figures 3 and S3). However, all tested substitutions decreased binding. Pursuing a “scaffold hopping” strategy, we also evaluated the quinoline analog of **1f**, which exhibited a higher binding affinity (**2a**, *K*_d_ = 3.2 μM) (Figures 4 and S4). We next tested alternatives of the 7-methoxy group in the context of the quinoline scaffold (**2b**–**2h**). Notably, replacement of the 7-methoxy with an ethoxy, cyclohexyloxy, or phenyloxy group further increased the binding affinity about three- to four-fold (**2b**, **2f**, and **2g**, *K*_d_ = 0.9 μM, 0.64 μM, and 0.68 μM, respectively), while other moieties showed no further improvement. The quinazoline derivative of **2b** (**1o**) bound to the CHD1 tCD with slightly lower affinity (*K*_d_ = 1.2 μM). Together, replacement of the 7-methoxy moiety with (slightly) larger substituents increased the ligand binding affinity of the quinolines to a submicromolar range.

**Figure 3:**
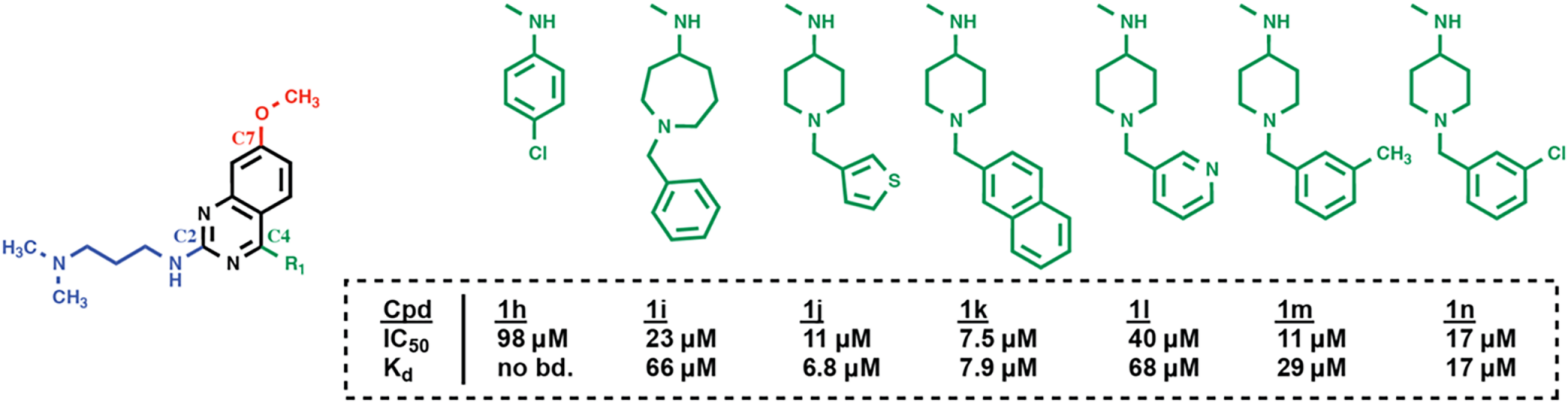
Focused structure–activity relationship (SAR) study at C4 of the quinazoline scaffold (compounds **1h**–**1n**). IC_50_ and *K*_d_ values for compound binding to the CHD1 tCD were determined by FRET and ITC, respectively. Original ITC data are shown in Figure S3. (Cpd: compound; no bd.: no binding.).

**Figure 4:**
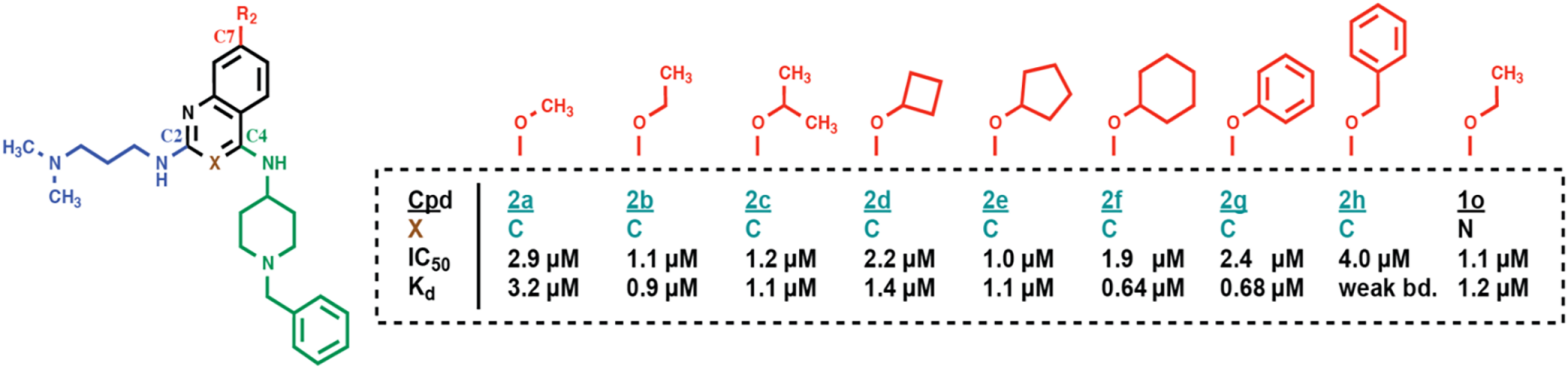
Quinazoline-to-quinoline switch and focused SAR study at C7 of the quinoline or quinazoline scaffold (compounds **2b**–**2h** and **1o**). Compound numbers of quinolines (X=C) are colored in green, compound numbers of quinazolines (X=N) in black. IC_50_ and *K*_d_ values for compound binding to the CHD1 tCD were determined by FRET and ITC, respectively. Original ITC data are shown in Figure S4. (Cpd: compound; weak bd.: weak binding.)

In the following step, we explored alternative substituents at C2 to rigidify the *N* ^3^ *,N* ^3^-dimethylpropane-1,3-diamine moiety of **2b** (Figures 5 and S5). Replacement with a 1-methylpiperidine-4-amine or a 2-imidazole moiety reduced or abolished ligand binding, respectively (**2i**, *K*_d_ = 4.6 μM; **2j**, no binding in ITC). In contrast, 4-alkylamino-piperidine substituents increased binding by a factor of about three to four (**2k**, **2l**, and **2m**, *K*_d_ = 0.28 μM, 0.44 μM, and 0.20 μM, respectively). When we applied the same modifications to the quinazoline scaffold (**1p**, **1q**, and **1r**), we observed weaker binding relative to the quinoline derivatives for all three compounds (*K*_d_ = 1.6 μM, 0.55 μM, and 1.5 μM, respectively). Overall, the 4-alkylaminopiperidine substituents favorably affected ligand binding to the CHD1 tCD. However, this was most noticeable in the context of the quinoline scaffold, hinting at differences in quinoline vs quinazoline binding.

**Figure 5:**
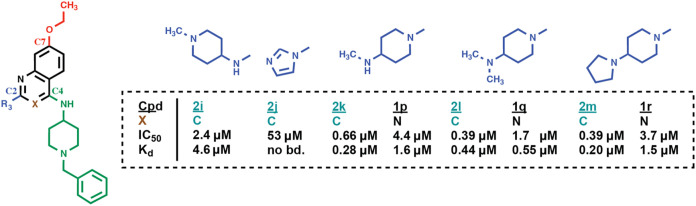
Focused SAR study at C2 of the quinoline or quinazoline scaffold. (compounds **2i**–**2m** and **1p**–**1r**). Compound numbers of quinolines (X=C) are colored in green, compound numbers of quinazolines (X=N) in black. IC_50_ and *K*_d_ values for compound binding to the CHD1 tCD were determined by FRET and ITC, respectively. Original ITC data are shown in Figure S5. (Cpd: compound; no bd.: no binding.).

### Crystal Structures of CHD1 tCD/Compound Complexes

For **2b**, **2l**, and **1q**, we obtained cocrystal structures upon soaking of CHD1 tCD/peptide crystals (Figures 6A–C and S6A, and Table S1). All crystal structures, as exemplified by the CHD1 tCD/**2l** complex refined at a resolution of 1.35 Å, showed that the *N*-benzylpiperidine moiety of the ligands acts as the Kme mimic, inserting into the aromatic cage formed by W322 and W325 of CHD1 chromo subdomain 1 (Figures 6A,C and S6A). The 7-ethoxyquinoline core binds to a shallow, mostly hydrophobic groove (which we termed “R-cleft” because it accommodates R113 of LSD1 in the CHD1 tCD/LSD1-K114me2 complex structure)^5^ at the interface of the two chromo subdomains. The 7-ethoxyquinoline scaffold adapts well to the shape of the R-cleft with a minimal distance of about 3.4 Å between C6 and the side chain of Y295. Accordingly, an additional methoxy substituent at C6 as e.g., in **1a** would force the ligand to partially rotate out of the groove to avoid a too close contact with the side chain of Y295. The 4-dimethylaminopiperidine moiety of **2l** binds in a region that is occupied by peptide N-terminal residues in CHD1 tCD/peptide complexes (see next paragraphs) and forms contacts with the “upper rim” of the ligand binding pocket.

**Figure 6:**
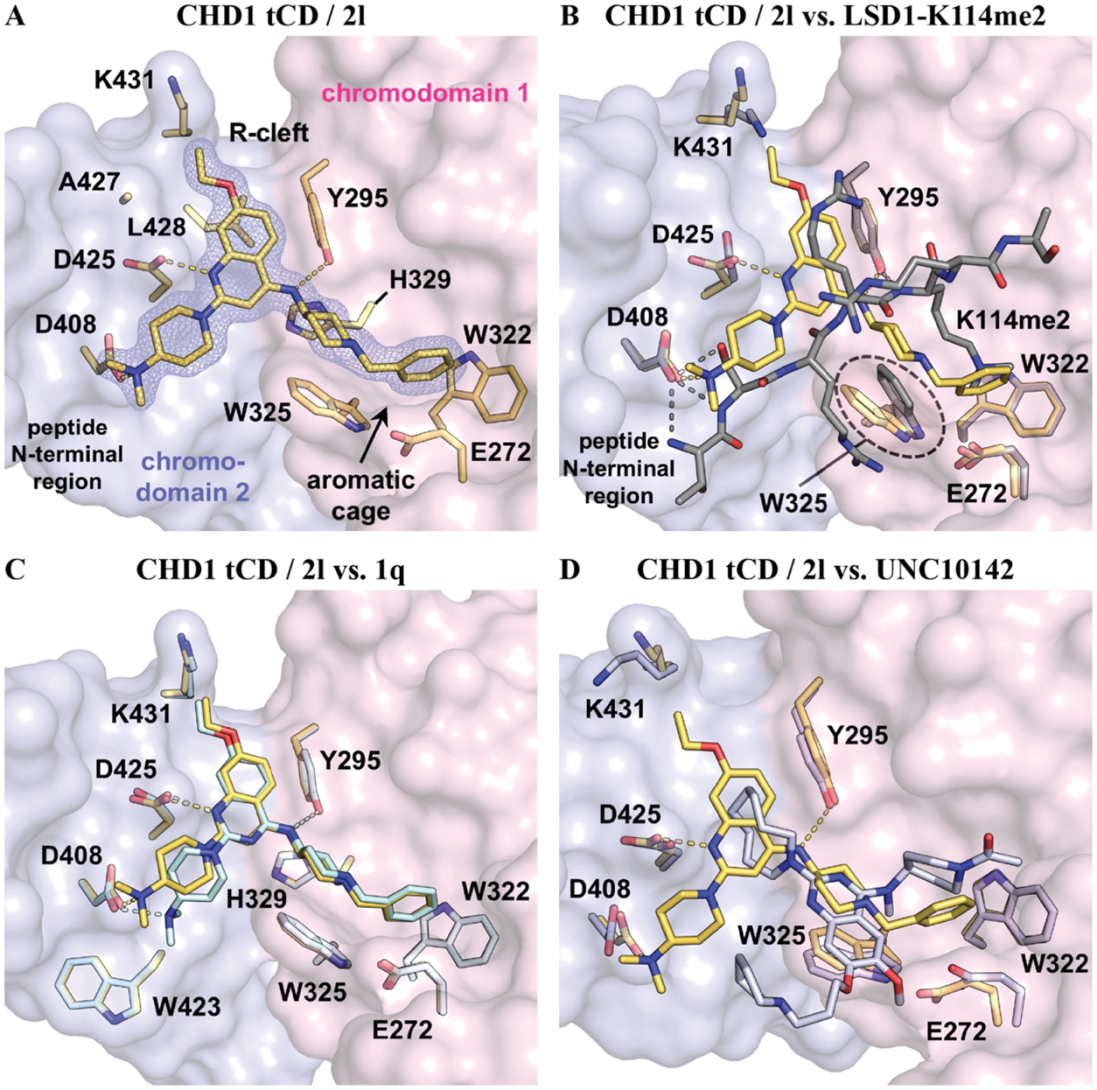
Crystallographic analysis of compound binding to the CHD1 tCD. (A) Crystal structure of the CHD1 tCD in complex with **2l** (yellow; PDB code 9T9F). The compound binds to the aromatic cage formed by W322 and W325, the “R-cleft” at the interface of the chromo subdomains 1 and 2 (colored light pink and light blue, respectively), and the “peptide N-terminal region”. Interactions of **2l** with the side chains of Y295, D408, and D425 contribute to binding. The electron density of the ligand was contoured at 1.0 σ. (B) Superimposition of the crystal structures of CHD1 tCD/LSD1-K114me2 peptide (gray; PDB code 5AFW) and CHD1 tCD/**2l** (yellow). The side chain of W325 is tilted in the CHD1 tCD/**2l** relative to its position in the CHD1 tCD/LSD1-K114me2 peptide complex, thereby accommodating the bulkier *N*-benzylpiperidine moiety of **2l**. (C) Superimposition of the CHD1 tCD cocrystal structures of **2l** (quinoline, yellow) and **1q** (quinazoline, pale cyan; PDB code 9T9G). Note the differences in the orientation of 4-dimethylaminopiperidine substituent in the peptide N-terminal region. (D) Superimposition of the CHD1 tCD cocrystal structures of **2l** (yellow) and **UNC10142** (light blue; PDB code 8UMG).^22^

Protein–ligand contacts were analyzed with the help of the Protein–Ligand Interaction Profiler.^39^ In the aromatic cage, the binding of **2l** is determined by a cation−π interaction between the tertiary amine of the piperidine moiety and W325 as well as an edge-to-face π-stacking (T-stacking) between the benzyl moiety of the ligand and W322. Other interactions contributing to ligand binding include hydrophobic contacts, a hydrogen bond with the side chain of Y295 (at a donor–acceptor distance of 3.5 Å), a salt bridge with the side chain of D408 (3.0 Å) and an interaction with the side chain of D425 (2.7 Å). Under physiological conditions, D425 would be expected to be deprotonated and not to form a hydrogen bond with the ligand. However, D425 is located at the N-terminus of an α-helix and forms, in addition to ligand contacts, a hydrogen bond with a main chain nitrogen, which may affect the local environment and thus protonation (Figure S6C). Furthermore, we noted a reorientation of the side chain of D425 toward N1 of the quinoline scaffold in CHD1 tCD/ligand but not CHD1 tCD/peptide complexes (Figures 6B and S6C). To validate the relevance of the interaction of D425 with **2l**, we tested ligand binding of a CHD1 tCD (D425A) mutant in ITC assays and observed a strongly reduced affinity (*K*_d_ = 9.7 μM; Figure S6D). Together, these observations define the determinants of binding of **2l** and related ligands to the CHD1 tCD.

Superimposition of the CHD1 tCD/**2l** complex with the crystal structures of the CHD1 tCD in complex with LSD1-K114me2 (PDB code 5AFW)^5^ or H3K4me3 (PDB code 2B2W)^4^ peptide showed that the ligand occupies a similar region as the peptides (Figures 6B and S6B). The aromatic benzyl substituent of **2l** occupies the position of the methylammonium moiety in the peptide complexes. In the CHD1 tCD/**2l** complex, the side chain of W325 is tilted relative to its conformation in both peptide complexes, thereby accommodating the bulkier *N*-benzylpiperidine moiety of the ligand and allowing formation of the cation−π interaction and the edge-to-face π-stacking. In the CHD1 tCD/LSD1-K114me2 complex, R113 of LSD1 binds to the R-cleft, which is occupied by the 7-ethoxyquinoline moiety in the **2l** complex. The 4-dimethylaminopiperidine moiety of **2l** binds to the region occupied by peptide N-terminal residues in the peptide complexes (Figures 6B and S6B).

The comparison of the **2l** (quinoline) and **1q** (quinazoline) cocrystal structures showed similar binding modes in the aromatic cage and the R-cleft (Figure 6C). C3 of the quinoline (corresponding to N3 of the quinazoline) core is located above carbons 5 and 6 of the indole side chain of W325 (at distances of 4.2 and 4.5 Å, respectively). In the peptide N-terminal region, however, we observed small differences between quinolines and quinazolines, which appeared as a small “rotation” of the 4-alkylaminopiperidine substituents relative to one another (Figure 6C). This “rotation” may result from slightly different carbon–nitrogen bond lengths in quinazolines compared to carbon–carbon bond lengths in quinolines, which, in consequence, alter bond angles and the spatial orientation of attached substituents. Thus, the 4-aminopiperidine-derived substituents of **2k**/**1p**, **2l**/**1q**, or **2m**/**1r** appear to form distinct contacts with the “upper rim” of the ligand pocket in quinolines compared to quinazolines, which may, at least in part, explain distinct binding affinities (Figure 5). In comparison, we did not observe differences for superimposed CHD1 tCD complexes of the quinolines **2b** and **2l** (Figure S6A).

Finally, we compared the cocrystal structure of **2l** with that of the recently reported CHD1 tCD inhibitor **UNC10142** (PDB code: 8UMG).^22^ Despite the similarity of our ligands and **UNC10142** in the molecule backbone, the molecules exhibit different binding modes (Figure 6D). Notably, whereas the *N*-benzylpiperidine moiety of our ligands forms strong cation−π and edge-to-face π-stacking interactions in the aromatic cage, the binding of **UNC10142** depends to a large extent on π−π stacking interactions between the quinazoline core and W325.^22^ We evaluated CHD1 tCD binding of **UNC10142** and two other related compounds, compound **29** ^36^ (Figure S1A) and **MS8535** ^21^ (Figure 1J) by ITC and observed under our experimental conditions K_d_ values of 2.0 μM, 10.1 μM, and 20.4 μM, respectively (Figure S6E). The higher relative affinity of **UNC10142** probably reflects its particular binding mode (Figure 6D).^22^ More importantly, these observations supported our decision to transition from a dimethoxyquinazoline to an ethoxyquinoline scaffold, resulting in submicromolar ligand affinities (*K*_d_ = 0.28 μM, 0.44 μM, 0.20 μM for **2k**, **2l**, **2m**, respectively). Together, our CHD1 tCD/ligand crystal structures provided a view of compound binding and explained the gains in potency during the steps of compound optimization.

### Further Ligand Extension

Aiming to further extend the 4-alkylaminopiperidine moiety in the peptide N-terminal region, we reasoned that it might be possible to achieve π-stacking with the side chain of W423 (see Figure 6C) by incorporation of an aromatic moiety into **2k** or **2l**. Accordingly, we tested a series of extended compounds (**2n**–**2q**, **1s**, and **1t**) (Figures 7A,B and S7A). **2n** was the most potent compound in this series, and we observed an increase in potency compared to its “precursor” **2l** (*K*_d_ = 0.15 μM vs *K*_d_ = 0.44 μM). **2o** (*K*_d_ = 0.23 μM) was only slightly less potent than **2n** (Figure 7A). In comparison, we observed lower binding affinities for the corresponding quinazolines **1s** and **1t** (*K*_d_ = 0.74 μM and *K*_d_ = 1.9 μM, respectively) (Figures 7A and S7A). Finally, replacement of the methoxybenzyl moiety of **2n** with methylbenzyl (**2p**) or nitrobenzyl (**2q**) negatively affected binding to the CHD1 tCD. Thus, **2n** (*K*_d_ = 0.15 μM) was identified as the most potent CHD1 tCD ligand.

**Figure 7:**
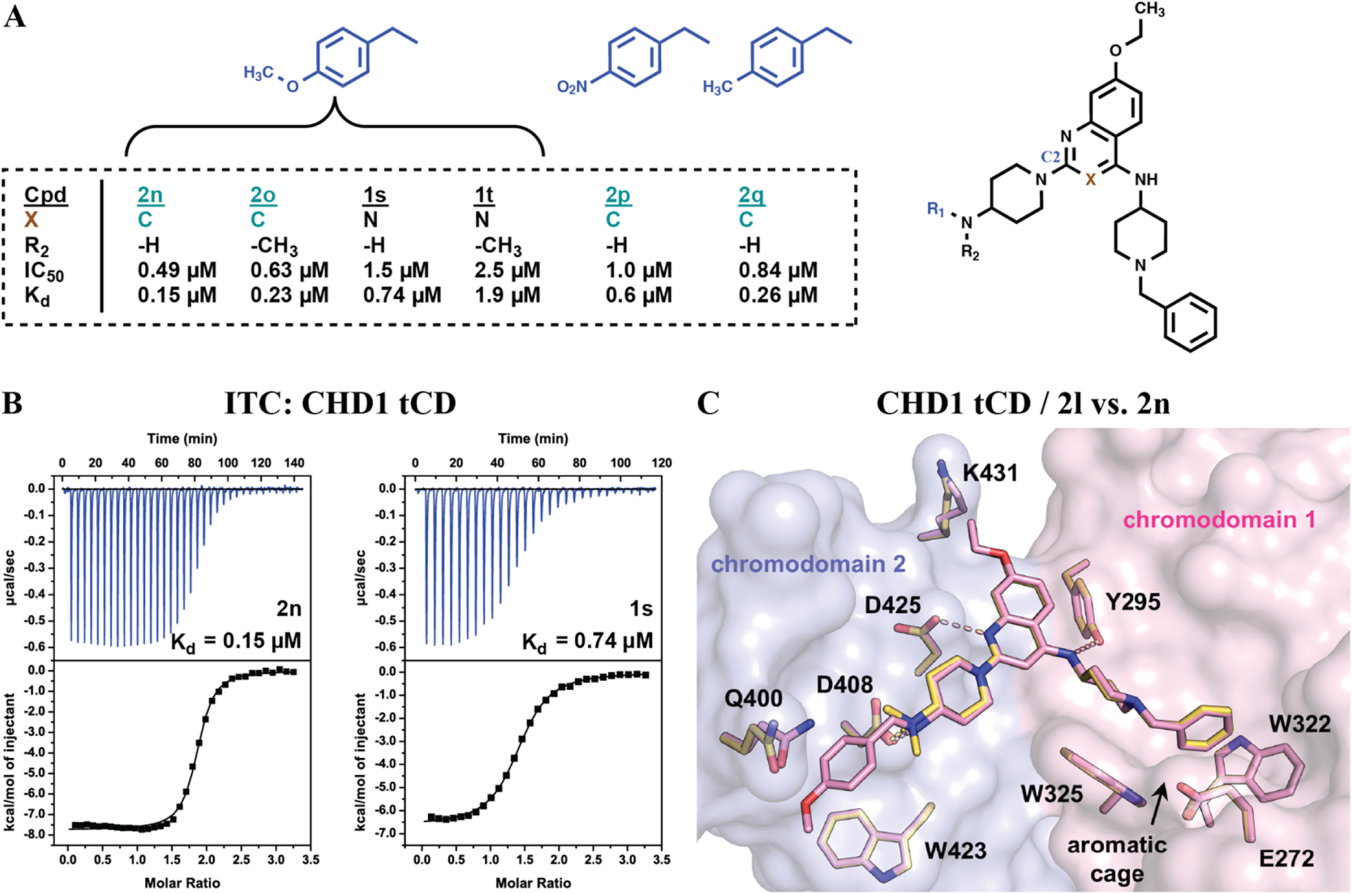
Further ligand extension. (A) Extension of the 4-alkylaminopiperidine moiety at C2 of the quinoline (**2n–2q**) or quinazoline scaffold (**1s** and **1t**). IC_50_ and *K*_d_ values for compound binding to the CHD1 tCD are listed. (**B**) ITC measurements for the CHD1 tCD and compounds **2n** and **1s**. (C) Superimposition of the CHD1 tCD cocrystal structures of **2l** (yellow; PDB code 9T9F) and **2n** (pink; PDB code 9T9H). Note the small conformational adaptation of the side chain of Q400 stacking with the methoxyphenyl moiety in the CHD1 tCD/**2n** complex.

We next solved the CHD1 tCD/**2n** cocrystal structure at a resolution of 1.45 Å. Superimposition of the CHD1 tCD/**2n** and CHD1 tCD/**2l** complexes showed almost identical binding modes (Figure 7C). Unintendedly, the additional methoxybenzyl moiety did not form π-stacking with the side chain of W423, but rather stacked with the side chain of Q400. This interaction appears to depend on a small conformational adaptation of the side chain of Q400, which is neither observed in the cocrystal structure of **2l** (Figure 7C) nor in that of **1q** (Figure S7B). This observation suggests that the stacking of Q400 with the methoxyphenyl moiety of **2n** contributes to the increased binding affinity relative to compounds such as **2l**. Due to the subtle geometric differences between the quinoline and the quinazoline scaffold (Figure 6C), the “C2-extended” quinazolines **1s** and **1t** probably cannot reach Q400 to form additional contacts. In summary, our structure–activity relationship (SAR) study led to the identification of high-affinity CHD1 tCD ligands and revealed the structural determinants of ligand binding.

### Cellular Target Engagement, Selectivity, and Effects on Cell Proliferation of CHD1 tCD Inhibitors

To allow the investigation of cellular target engagement of **2n**, we next aimed to generate a TAMRA probe using a click-chemistry approach. Precursor molecules of the click-chemistry reaction, **2r** and **2s**, exhibited binding affinities (*K*_d_ = 0.18 and *K*_d_ = 0.14 μM, respectively) similar to **2n** (Figures 8A and S8A). Furthermore, **2n** and **2s** exhibited similar stabilization of the CHD1 tCD in fluorescent thermal shift assays (FTSA) (Figure S8B). Therefore, further molecule extension did not negatively affect ligand binding. Accordingly, the CHD1 tCD/**2s** cocrystal structure, refined at a resolution of 1.55 Å, showed binding similar to **2n** with the exception of additional contacts of **2s** in the peptide N-terminal region (Figures 8B and S8C). In the crystal, the triazole moiety of **2s** interacts with the N-terminus of an α-helix including residues S398, N399, and Q400. However, these interactions do not result in an increased binding affinity of **2s** compared to **2n**. The TAMRA derivative of **2s** (**2s-TAMRA**) (Figure S8D) bound to the CHD1 tCD in ITC assays (Figure S8E). We next assayed in bioluminescence resonance energy transfer (NanoBRET) assays **2s-TAMRA** binding to exogenous GFP-CHD1 tCD in transfected human embryonic kidney HEK293T cells and observed a dose-dependent target engagement (IC_50_ = 15 μM; Figure 8C). To address the question whether our ligands could bind full-length CHD1, we performed a peptide-based pulldown assay as previously described by Johnson et al. for **UNC10142** ^22^ using a biotinylated LSD1-K114me3 peptide and HEK293T cell extract preincubated with **2s** or DMSO vehicle control. The potential alternative, a cellular thermal shift assay (CETSA), failed, most likely because the CHD1 tCD accounts for only about 10% of the full-length protein.^22^ In our pulldown assay, we observed enrichment of cellular CHD1 by LSD1-K114me3 and a concentration-dependent competition of this interaction by **2s** (Figure 8D). We noted, however, that the required concentrations of **2s** were relatively high. Yet, this observation is in accordance with data by Johnson et al.^22^

**Figure 8:**
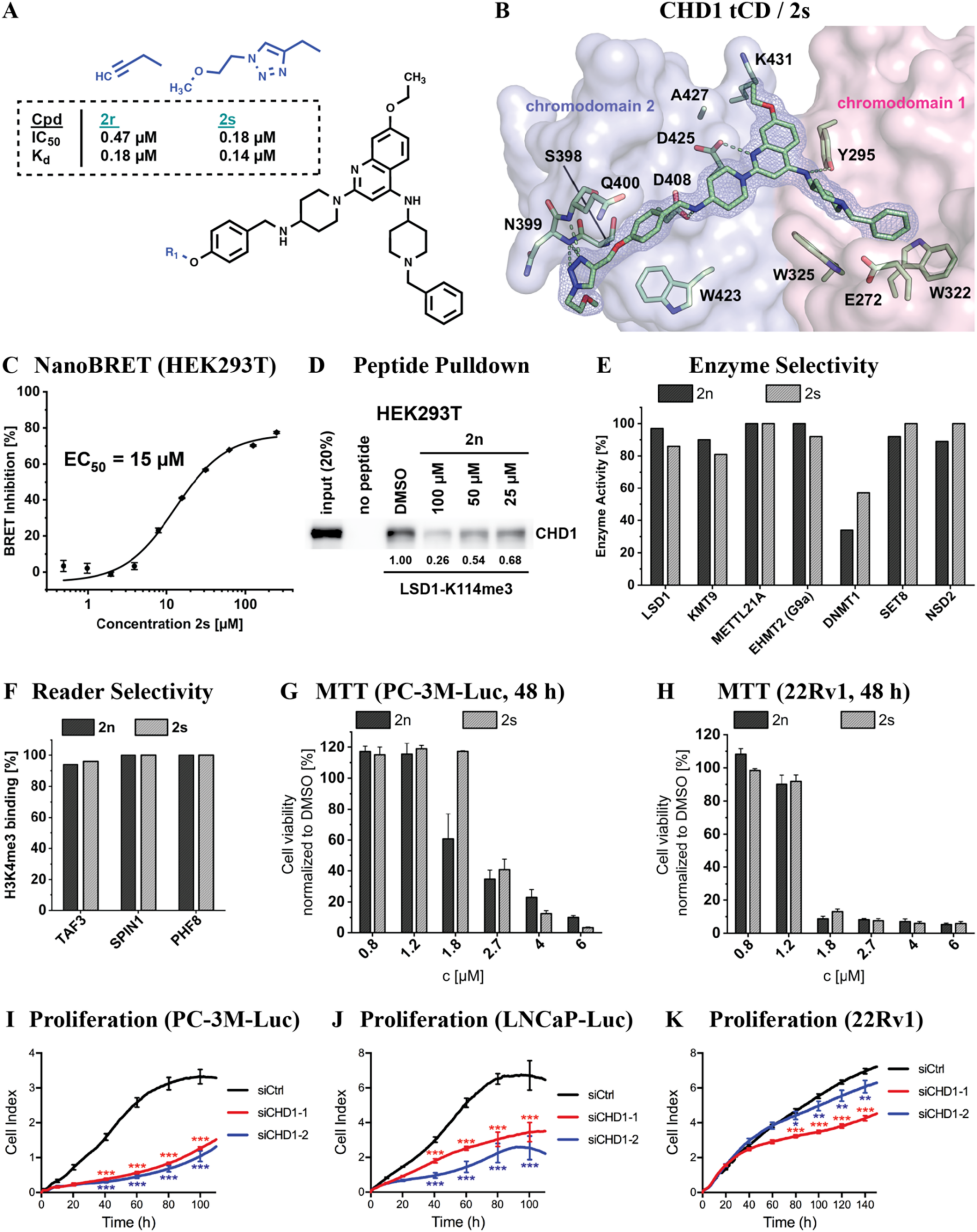
Target engagement and selectivity of CHD1 tCD ligands. (A) Further extension of **2n** to mimic click-chemistry modification (**2r** and **2s**) for the generation of a TAMRA probe. IC_50_ and *K*_d_ values for compound binding to the CHD1 tCD are listed. (B) Crystal structure of the CHD1 tCD/**2s** complex (PDB code 9T9I). The electron density of the ligand was contoured at 1.0 σ. (C) Displacement of **2s-TAMRA** from nanoluciferase (NLuc)–CHD1 tCD by **2s** in transfected HEK293T cells determined by NanoBRET assay. (D) Inhibition of binding of full-length CHD1 to a LSD1-K114me3 peptide by **2n**. A biotinylated LSD1-K114me3 peptide was immobilized on streptavidin sepharose beads and incubated with HEK293T cell extract treated with the indicated concentrations of **2s** or DMSO vehicle control. Bound CHD1 was detected by Western blot. Relative, normalized signal intensities are indicated. (E) Inhibition of catalytic activity of the indicated epigenetic enzymes by **2n** and **2s** at 10 μM was determined by activity assays described in the Experimental Section. (F) Inhibition of H3K4me3 peptide binding to the reader domains of TAF3, SPIN1, or PHF8 by **2n** and **2s** (at 16 μM) determined by FRET assay. (G, H) Cell viability of PC-3M-Luc (G) and 22Rv1 (**H**) cells after 48 h treatment with **2n** or **2s** determined by MTT assay. (**I**–**K**) Proliferation of PC-3M-Luc (I), LNCaP-Luc (J), and 22Rv1 (K) cells upon treatment with two different siRNAs directed against CHD1 (siCHD1–1 and siCHD1–2) or control siRNA (siCtrl). Knockdown efficiencies are shown in Figure S8J.

The CHD1 tCD inhibitors developed in this study are based on a quinazoline/quinoline scaffold, which was previously used in inhibitors of other epigenetic regulators including SPIN1 (Figure 1J),^21^ LSD1 (Figure S1A),^36^ SETD8 (Figure S1B),^37^ and GLP/G9a (EHMT1/2) (Figure S1C).^38^ Furthermore, our initial hit **1a** was previously identified first as GLP/G9a inhibitor (compound E11),^40^ and then as dual G9a/LSD1 inhibitor.^41^ Therefore, we tested the target selectivity of **2n** and **2s** against a panel of epigenetic regulators with known, structurally similar inhibitors. We observed that **2n** and **2s** neither inhibited the activities of LSD1, KMT9, METTL21A, G9a (EHMT2), SET8, or NSD2 (Figure 8E) nor bound to the reader proteins TAF3, SPIN1, or PHF8 (Figure 8F). Furthermore, **2n** did not significantly increase the melting temperature of METTL21B in FTSA assays (Figure S8F). We also checked **2s** against a panel of kinases and observed no relevant inhibition of kinase activity at 10 μM (Table S2). However, we noted a weak inhibition of DNMT1 with IC_50_ = 6.2 μM (**2n**) and IC_50_ = 17.5 μM (**2s**), which is about 40- and >100-fold, respectively, above the *K*_d_ of about 0.15 μM determined for CHD1 tCD binding of both compounds (Figure 8E). These observations suggest that **2n** and **2s** selectively bind to the CHD1 tCD despite structural similarity with other quinazoline/quinoline inhibitor scaffolds.

In the final set of experiments, we evaluated potential effects of **2n** and **2s** on the viability of PTEN-negative (PC-3M-Luc) or PTEN-positive (22Rv1) PCa cells. As a control compound, we chose **2j** (see Figure 5). In 3-(4,5-dimethylthiazol-2-yl)-2,5-diphenyl-2*H*-tetrazolium bromide (MTT) assays, **2n** and **2s** affected the viability of both cell lines after 48 h of treatment with IC_50_ values of around 1.3–2.5 μM (Figures 8G,H, and S8H,I). In comparison, for the control compound **2j**, we only observed a small effect on cell viability at 10 μM after 5 days of treatment (Figure S8G). Compared to our ligands, the recently reported CHD1 tCD inhibitor **UNC10142** was observed to suppress the growth of prostate cancer cells at high ligand concentrations (87% and 54% viability loss of PC-3 and LNCaP cells, respectively, at 75 μM).^22^

The effect of **2n** and **2s** on the viability of PTEN-positive 22Rv1 cells was unexpected, given previous observations by Zhao et al. showing that CHD1 depletion had minimal effect on the growth of tumors derived from these cells in mice.^30^ To address this issue, we investigated proliferation of PC-3M-Luc, LNCaP-Luc, and 22Rv1 cells upon knockdown of CHD1. While proliferation of PC-3M-Luc and LNCaP-Luc cells was strongly compromised by CHD1 knockdown using two different siRNAs (siCHD1–1, siCHD1–2) compared to control (siCtrl), we observed a small effect on the proliferation of 22Rv1 cells (Figures 8I–K and S8J). On the one hand, these data suggest that ligand effects on 22Rv1 cell proliferation may, at least in part, be mediated by inhibition of CHD1. On the other hand, our observations hint at some off-target activity. Together, our data suggest that more work is required to precisely characterize the cellular activities and potential off-target effects of our inhibitors.

### Synthesis

The synthesis and characterization of compounds **1a**,^41^ **1b**,^41^ **1c**,^42^ and **1d**,^42^ were previously reported. The synthetic routes for compounds **1c** and **1d** are depicted in Scheme 1.

**Scheme 1:**
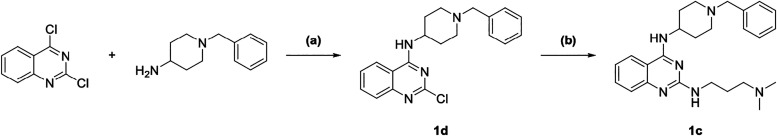
Synthesis of Compounds 1c and 1d^a^

a Reagent and conditions: (a) DIPEA, NMP, 150 °C, overnight, 80%; (b) *N* ^1^,*N* ^1^-dimethylpropane-1,3-diamine, HCl (4 M), *i*-PrOH, microwave, 160 °C, 3 h, 68%.

The synthetic routes for the preparation of the final derivatives **1e**, **1g**, and **1i**–**1t** are depicted in Scheme 2. The respective substituted quinazoline-2,4­(1*H*,3*H*)-diones **3** (commercially available) and **4** (prepared as reported in Scheme S1) were treated with diethylaniline and phosphorus oxychloride (POCl_3_) at 150 °C, affording the respective intermediates **6** ^43^ and **8**. These derivatives, along with commercially available **5** and **7**, were then treated with the appropriate amine (**9**, **10**, **14**, **15** were commercially available, **11–13** were prepared as described in Scheme S2) in the presence of *N*,*N*-diisopropylethylamine (DIPEA) in anhydrous *N*,*N*-dimethylformamide (DMF) at room temperature (rt), leading to regioselective C4 nucleophilic displacement and furnishing intermediates **16**–**24**. These intermediates were subsequently converted into final derivatives **1e**, **1g**, **1i**–**1o**, **1r**, and **1t**, as well as the intermediates **25** and **26**, via the C2 displacement on the quinazoline ring using either commercially available amines or the in-house prepared intermediate **36** (prepared as reported in Scheme S3) under microwave irradiation at 130 °C in isopropanol. Final compound **1p** and intermediate **27** were obtained by removal of the *tert*-butoxycarbonyl protection group from **25** and **26**, respectively, via acidic treatment (4 M hydrochloric acid in 1,4-dioxane) in anhydrous methanol (MeOH) with triisopropylsilane (TIPS) at rt. Finally, a reductive amination reaction yielded compound **1s**. In this step, intermediate **27** was first treated with 4-methoxybenzaldehyde and glacial acetic acid at rt, followed by reduction with sodium cyanoborohydride at rt (Scheme 2).

**Scheme 2:**
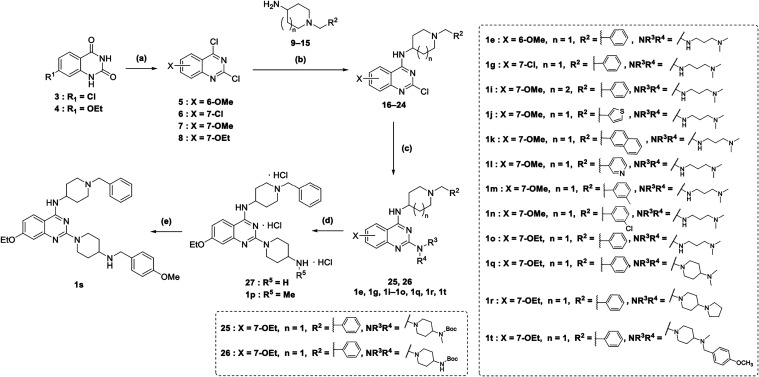
Synthesis of Compounds 1e, 1g, and 1i–1t^a^

a Reagents and conditions: (a) diethylaniline, POCl_3_, 150 °C, 4 h, 76–81%; (b) DIPEA, anhydrous DMF, 0 °C to rt, 6 h, 75–92%; (c) appropriate amine, *i*-PrOH, microwave, 130 °C, 1 h, 49–82%; (d) TIPS, HCl (4 M), anhydrous MeOH, 0 °C to rt, 5 h, 85–93%; (e) 4-methoxybenzaldehyde, glacial AcOH, anhydrous MeOH, molecular sieves (4 Å), 0 °C to rt, N_2_, 2.5 h, then NaBH_3_CN, rt, 25 h, 44%.

The synthetic routes for final derivatives **2b**–**2e** are depicted in Scheme 3. Intermediate **39** (prepared as reported in Scheme S4) was alkylated with the corresponding commercially available alkyl bromides under basic conditions (K_2_CO_3_) and in the presence of KI at 80 °C in anhydrous DMF affording intermediates **40**–**43** in varying yields. These intermediates were subsequently treated with 1-benzylpiperidine-4-amine in the presence of DIPEA in *N*-methyl-2-pyrrolidone (NMP) at 150 °C, yielding compounds **44**–**47**. Final compounds **2b**–**2e** were obtained by treatment of **44**–**47** with *N* ^1^,*N* ^1^-dimethylpropane-1,3-diamine and 4 M HCl in isopropanol under microwave irradiation at 160 °C.

**Scheme 3:**
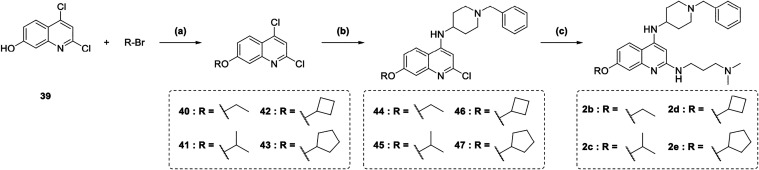
Synthesis of Compounds 2b–2e^a^

a Reagent and conditions: (a) K_2_CO_3_, KI, DMF, 80 °C, overnight, 26–93%; (b) 1-benzylpiperidine-4-amine, DIPEA, NMP, 150 °C, overnight, 31–43%; (c) *N* ^1^,*N* ^1^-dimethylpropane-1,3-diamine, HCl (4 M), *i*-PrOH, microwave, 160 °C, 3 h, 31–75%.

In the first step of the preparation of quinolines **2a** and **2g**, a chlorination-driven condensation of anilines with malonic acid was performed using POCl_3_ at 95 °C for 30 min. Subsequently, additional POCl_3_ was added, and the reaction mixture was heated to 120 °C, affording intermediates **48** and **49** (Scheme 4). In the next step, a nucleophilic aromatic substitution S_N_Ar was carried out with 1-benzylpiperidine-4-amine, yielding compounds **50** and **51** in moderate yields. Final products **2a** and **2g** were obtained through a second S_N_Ar reaction under acidic, microwave-assisted conditions, using intermediates **50**, **51**, and *N* ^1^,*N* ^1^-dimethylpropane-1,3-diamine.

**Scheme 4:**
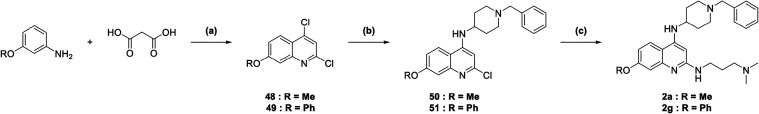
Synthesis of Compounds 2a and 2g^a^

a Reagent and conditions: (a) POCl_3_, rt to 95 °C, 30 min, then add more POCl_3_, rt to 120 °C, 3 h, 45–58%; (b) 1-benzylpiperidine-4-amine, DIPEA, NMP, 150 °C, overnight, 28–40%; (c) *N* ^1^,*N* ^1^-dimethylpropane-1,3-diamine, HCl (4 M), *i*-PrOH, microwave, 160 °C, 3 h, 67–79%.

Quinolines **2h** and **2f** were prepared via Williamson ether synthesis of previously described intermediate **39** with either (bromomethyl)­benzene or under Mitsunobu reaction conditions with cyclohexanol, yielding intermediates **52** and **53** (Scheme 5). These were subsequently subjected to an S_N_Ar with 1-benzylpiperidine-4-amine, following the same procedure as described for **2b**–**2e** (Scheme 3), to afford intermediates **54** and **55**. A second microwave-assisted S_N_Ar under acidic conditions using *N* ^1^,*N* ^1^-dimethylpropane-1,3-diamine then furnished the final products **2h** and **2f**.

**Scheme 5:**
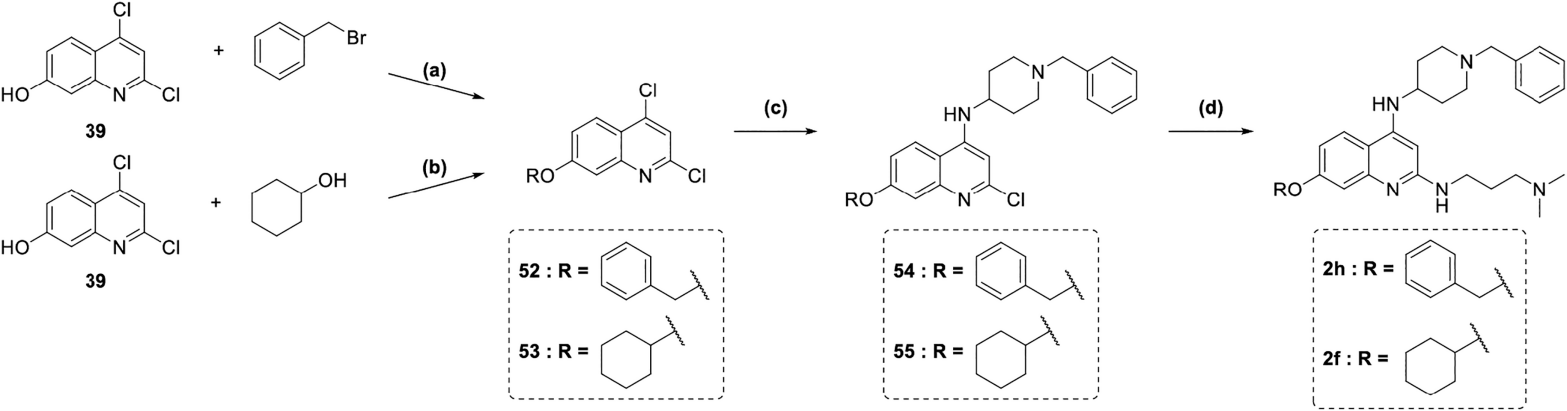
Synthesis of Compounds 2f and 2h^a^

a Reagent and conditions: (a) NaH, DMF, 0 °C to rt, 1 h, 75%; (b) DIAD, PPh_3_, THF, 80 °C, 36 h, 61%; (c) 1-benzylpiperidine-4-amine, DIPEA, NMP, 150 °C, overnight, 37%; (d) *N* ^1^,*N* ^1^-dimethylpropane-1,3-diamine, HCl (4 M), *i*-PrOH, microwave, 160 °C, 3 h, 42–80%.

Final compounds **2i**–**2m** were synthesized by treating previously described intermediate **44** (Scheme 3) with the corresponding amines under microwave-assisted S_N_Ar conditions (Scheme 6).

**Scheme 6:**
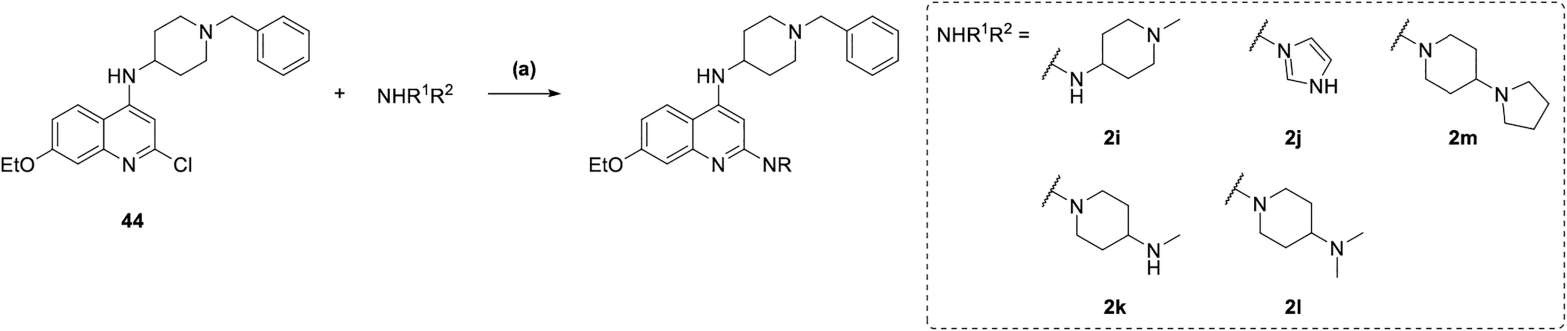
Synthesis of Compounds 2i–2m^a^

a Reagent and conditions: (a) HCl (4 M), *i*-PrOH, microwave, 160 °C, 3 h, 43–87%.

Commercially available *tert*-butyl 4-oxopiperidine-1-carboxylate and (4-methoxyphenyl)­methanamine were subjected to reductive amination conditions with sodium cyanoborohydride and glacial acetic acid in dry MeOH at rt, yielding intermediate **56** (Scheme 7). Deprotection with trifluoroacetic acid (TFA) in dichloromethane (DCM) at rt furnished **57**. In parallel, treatment of **56** with formaldehyde under reductive amination conditions gave methylated amine **58**, which was likewise deprotected using TFA to afford **59**. To access the nitro- and methyl-substituted intermediates **62** and **63**, an N-alkylation of Boc-protected piperidine amine with a bromobenzyl derivative was first performed, yielding intermediates **60** and **61**. Subsequent deprotection with TFA provided amines **62** and **63**.

**Scheme 7:**
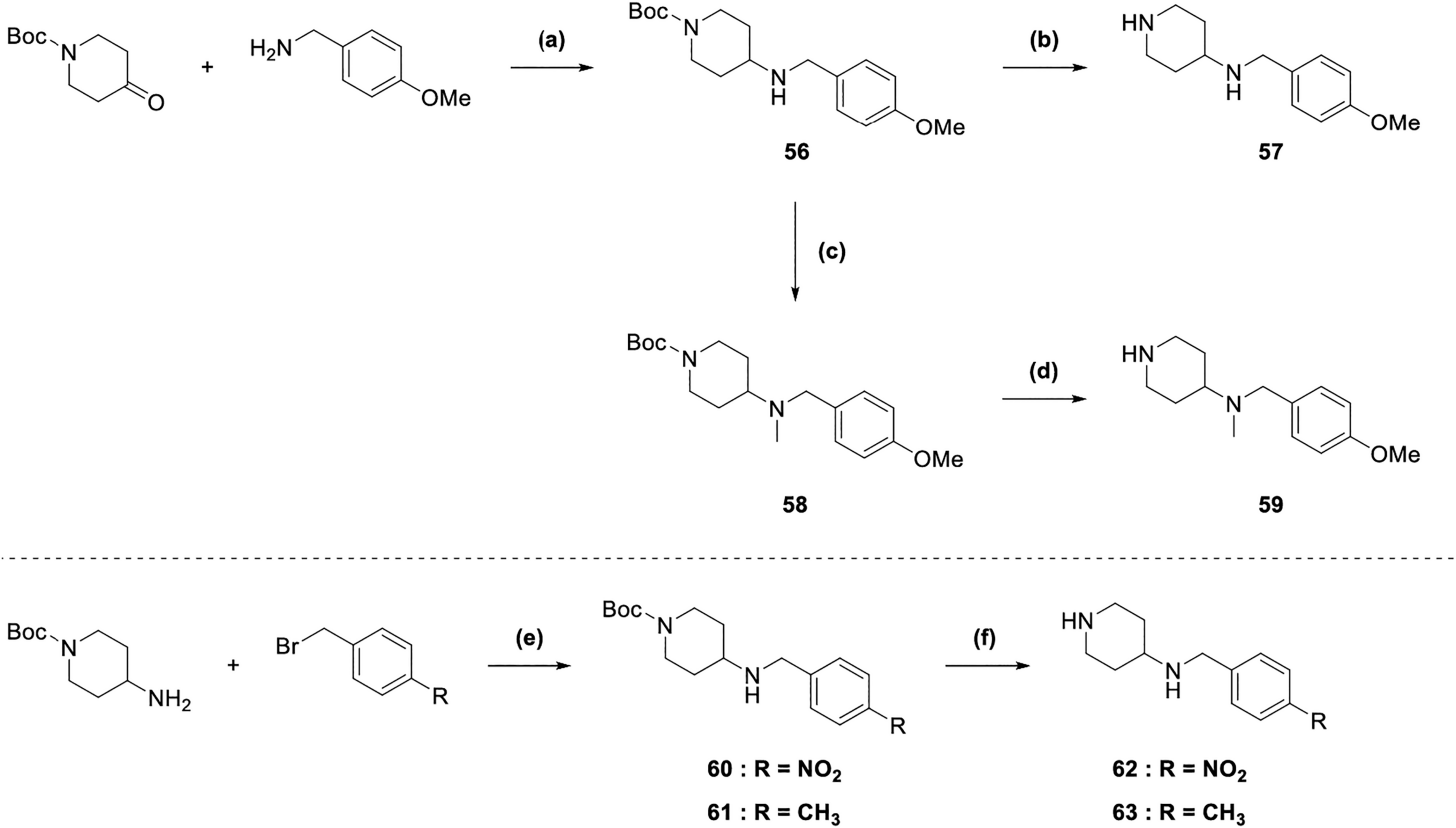
Synthesis of Intermediates 56–63^a^

a Reagent and conditions: (a) NaBH_3_CN, AcOH, MeOH, rt, overnight, 92%; (b) TFA, DCM, rt, overnight, quant.; (c) HCHO, NaBH_3_CN, AcOH, MeCN, rt, overnight, 87%; (d) TFA, DCM, rt, overnight, quant.; (e) K_2_CO_3_, KI, MeCN, reflux, overnight, 39–65%; (f) TFA, DCM, rt, overnight, quant.

A microwave-assisted S_N_Ar under acidic conditions was conducted using amines **57**, **59**, **62**, and **63** with quinoline **44** (Scheme 8) affording the final compounds **2n**–**2q**.

**Scheme 8:**
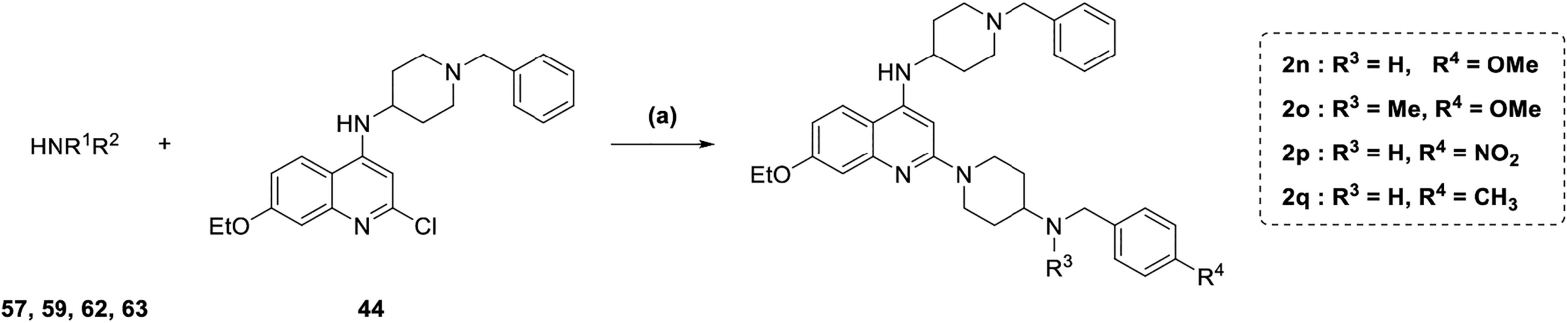
Synthesis of Compounds 2n–2q^a^

a Reagent and conditions: (a) HCl (4 M), *i*-PrOH, microwave, 160 °C, 3 h, 63–80%.

The preparation of **64** (Scheme 9) was carried out via reductive amination with (4-(prop-2-yn-1-yloxy)­phenyl)­methanamine. Subsequent deprotection with TFA afforded **65**. A microwave-assisted S_N_Ar of **44** with **65** furnished compound **2r** in good yields. A subsequent copper­(I)-catalyzed azide–alkyne cycloaddition (CuAAC) afforded the 1,4-disubstituted triazole derivatives **2s** and **2s-TAMRA**.

**Scheme 9:**
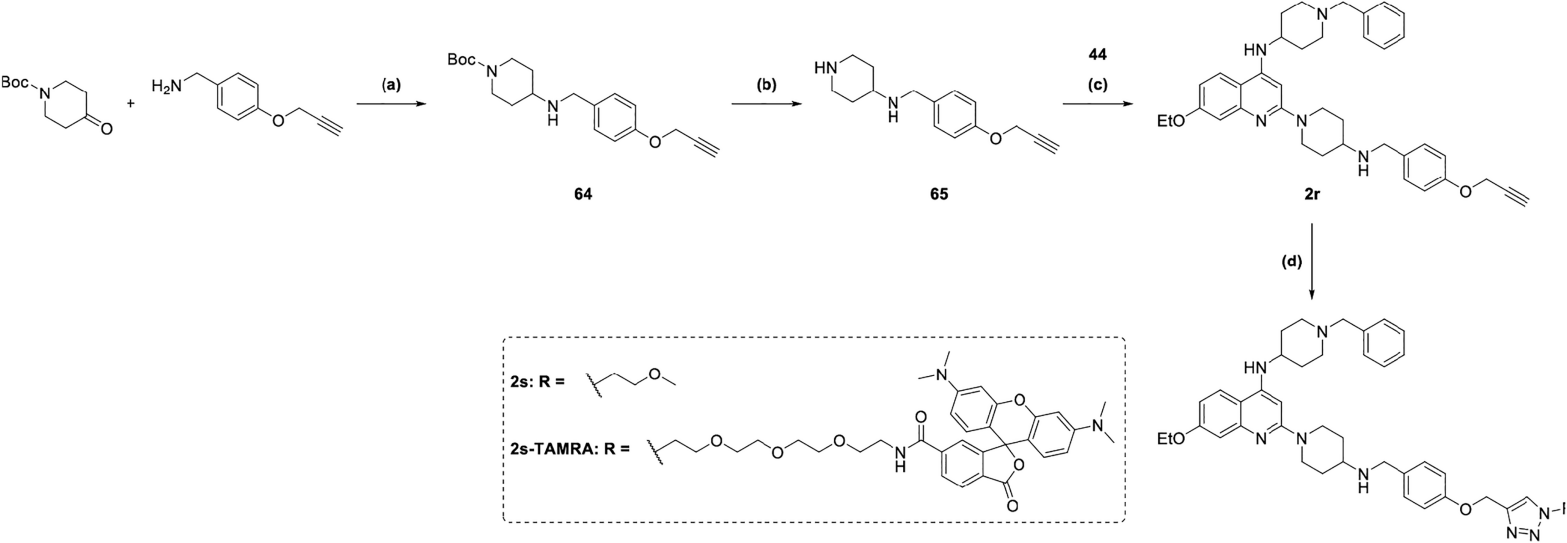
Synthesis of Compound 2r, 2s, and 2s-TAMRA^a^

a Reagent and conditions: (a) NaBH_3_CN, AcOH, MeOH, rt, overnight, 39%; (b) TFA, DCM, rt, overnight, quant.; (c) intermediate **44**, HCl (4 M), *i*-PrOH, microwave, 160 °C, 3 h, 69%; (d) 1-azido-2-methoxyethane (for compound **2s**) or *N*-(2-(2-(2-(2-azidoethoxy)­ethoxy)­ethoxy)­ethyl)-3′,6′-bis­(dimethylamino)-3-oxo-3*H*-spiro­[isobenzofuran-1,9′-xanthene]-6-carboxamide (N_3_–PEG_3_-TAMRA) (for compound **2s-TAMRA**), Tris­[(1-benzyl-1*H*-1,2,3-triazol-4-yl)­methyl]­amin (TBTA) (for **2s-TAMRA**), CuSO_4_·5H_2_O, sodium ascorbate, *t*-BuOH/H_2_O (1:1) and DMF (for **2s-TAMRA**), 40 °C or rt (for **2s-TAMRA**), 5 or 12 h (for **2s-TAMRA**), 40–84%.

## Conclusions

In this study, we developed inhibitors, **2n** (**FRC-222**) and **2s** (**FRC-303**), binding to the CHD1 tCD with affinities of about 0.15 μM. This is only the second report on CHD1 tCD inhibitors and the first one describing ligands with submicromolar affinities. The features of our compounds are distinct from those of the recently reported, first CHD1 tCD inhibitor **UNC10142**, which is based on a dialkyloxyquinazoline scaffold.^22^ In comparison, our highest affinity compounds **2n** and **2s** are based on a 7-ethoxyquinoline scaffold. They exhibit aromatic cage binding with a *N*-benzylpiperidine moiety mimicking Kme interactions and forming cation−π and edge-to-face π-stacking interactions. Furthermore, possibly due to small geometric differences with quinazolines, the quinoline scaffold favors protein–ligand contacts in the peptide N-terminal region of the CHD1 tCD peptide binding pocket. In summary, our comprehensive SAR study describes high-affinity CHD1 tCD inhibitors with a unique set of binding features that have not been reported so far.

## Experimental Section

### Chemistry General Procedures

Room temperature (rt) refers to 22 °C unless otherwise specified. All reagents and anhydrous solvents were transferred using oven-dried syringes or cannulas. Reaction flasks were flame-dried under vacuum, and cooled under a constant stream of argon. Anhydrous solvents (DMF, DCM, and acetonitrile) were purchased from Sigma-Aldrich (stored over molecular sieves), while THF was dried over potassium. All other chemicals were obtained at the highest commercially available purity from ABCR, Acros, Alfa Aesar, BLDpharm, Fluorochem, Merck, Sigma-Aldrich, or TCI Europe and were used without further purification. Thin-layer chromatography (TLC) was performed on Merck Silica gel 60 F254 aluminum plates and visualized under UV light (254 nm) and/or by staining (ceric ammonium molybdate, potassium permanganate, ninhydrin) or using an iodine chamber. Flash column chromatography was carried out on MACHEREY-NAGEL Silica gel 60 (230–400 mesh) under forced flow (Still method). Yields refer to chromatographically purified and spectroscopically pure compounds. NMR (nuclear magnetic resonance) spectra were recorded on Bruker Avance 300, 400, or 500 MHz DRX spectrometers. Chemical shifts (δ) are reported in parts per million (ppm) relative to the solvent resonance as an internal standard (chloroform d: δ = 7.26 for ^1^H and δ = 77.16 for ^13^C; dimethyl sulfoxide d_6_: δ = 2.50 for ^1^H and δ = 39.52 for ^13^C; methanol d_4_: δ = 3.31 for ^1^H and δ = 49.00 for ^13^C; and dichloromethane d_2_: δ = 5.32 for ^1^H and δ = 53.84 for ^13^C). Multiplicities are given as s (singlet), d (doublet), t (triplet), q (quartet), quint (quintet), dd (doublet of doublet), dt (doublet of triplet), dq (doublet of quartet), m (multiplet), or br s (broad singlet). Coupling constants (*J*) are expressed in hertz (Hz). High-resolution mass spectra (HRMS) were measured on Thermo Scientific Advantage, Thermo Scientific Exactive, Thermo Scientific Q-Exactive UHMR instruments equipped with APCI or ESI sources. Microwave-assisted reactions were conducted using a CEM Discover SP microwave system (Buckingham, U.K.). HPLC analyses were performed to determine the purity of the final compounds on an Agilent Technologies 1260 Infinity II system or on a Hitachi Elite LaChrom system (L-2130 pump), or a Thermo Scientific UltiMate 3000 UHPLC system each equipped with a diode array detector (DAD). UV detection was carried out at 254 and 282 nm. The method for analytical HPLC employed an XBridge BEH Shield RP18 column (130 Å, 5 μm, 4.6 mm × 150 mm) or for preparative HPLC a BEH Shield RP18 OBD column (130 Å, 5 μm, 19 mm × 150 mm). EluentA was H_2_O containing 0.05% trifluoroacetic acid (TFA), and eluent B was MeCN. The linear gradient was as follows: 0–4 min, 90:10 (A/B); 4–19 min, 0:100 (A/B); 19–21 min, 0:100 (A/B); 21–21.5 min, 90:10 (A/B); and 21.5–25 min, 90:10 (A/B), all at a flow rate of 1.0 mL/min, 0.6 mL/min (analytical HPLC) or 17.1 mL/min (preparative HPLC). Alternatively, the method for analytical HPLC employed a Hypersil GOLD C18 Selectivity column (175 Å., 5 μm, 4.6 mm × 250 mm). Eluent A was H_2_O containing 0.1% TFA, and eluent B was MeCN containing 0.1% TFA. The linear gradient was as follows: 0–5 min, 90:10 (A/B); 5–30 min, 10:90 (A/B); 30–35 min, 10:90 (A/B); 35–37 min, 90:10 (A/B); and 37–45 min, 90:10 (A/B), all at a flow rate of 1.0 mL/min. All compounds assessed for biological activity showed >95% purity according to this HPLC method.

### Procedures for the Synthesis of Compounds 1e, 1g, 1i–1t and Related Intermediates

#### General Procedure for the Synthesis of Final Compounds 1e, 1g, 1i–1o, 1q, 1r, 1t, and Intermediates **25** and **26**

The intermediates **16**–**24** (0.176 mmol, 1 equiv) were mixed in a microwave glass vial and dissolved in isopropanol (1 mL). The appropriate amine (0.387 mmol, 2.2 equiv) was then added, and the resulting mixture was heated to 130 °C by microwave irradiation for 1 h. Upon completion of the reaction, the mixture was extracted with ethyl acetate (40 mL) and the organic phase was washed with 2 × 3 mL of a saturated sodium chloride solution, dried over anhydrous sodium sulfate, and finally concentrated under vacuum. The crude product was then purified by silica gel column chromatography using the appropriate chloroform/methanol/ammonia mixture as the mobile phase to afford final compounds **1e**, **1g**, **1i**–**1o**, **1q**, **1r**, **1t**, and intermediates **25** and **26**.

#### *N* ^4^-(1-Benzylpiperidine-4-yl)-*N* ^2^-(3-(dimethylamino)-propyl)-6-methoxyquinazoline-2,4-diamine (**1e**)

Colorless solid. Yield, 59%. ^1^H NMR (400 MHz, DMSO-*d*_6_) δ 7.47 (d, 1H, N*H*), 7.36–7.28 (m, 5H, 3 × C*H* benzene ring +2 x C*H* quinazoline ring), 7.27–7.23 (m, 1H, C*H* benzene ring), 7.18–7.11 (m, 2H, C*H* benzene ring + C*H* quinazoline ring), 6.22 (br s, 1H, N*H*-CH_2_), 4.17–4.09 (m, 1H, C*H* piperidine ring), 3.80 (s, 3H, OC*H*_3_), 3.52 (br s, 2H, NC*H*_2_-Ph), 3.26 (q, 2H, C*H*_2_NH), 2.89 (d, 2H, 2 x C*H* piperidine ring), 2.25 (t, 2H, C*H*_2_N­(CH_3_)_2_), 2.12 (s, 6H, N­(C*H*_3_)_2_), 2.05 (t, 2H, 2 x C*H* piperidine ring), 1.92 (d, 2H, 2 x C*H* piperidine ring), 1.70–1.60 (m, 4H, 2 x C*H* piperidine ring + CH_2_C*H*_2_CH_2_); ^13^C NMR (101 MHz, DMSO-*d*_6_) δ 158.71, 158.5, 153.0, 147.2, 138.6, 128.8 (2C), 128.2 (2C), 126.9, 126.1, 122.7, 110.5, 103.2, 62.2, 57.2, 55.7, 52.5 (2C), 47.6, 45.3 (2C), 38.5, 31.6 (2C), 27.7; ESI-HRMS (*m*/*z*): [M + H]^+^ calcd for C_26_H_37_N_6_O^+^, 449.3023; found, 449.3029.

#### *N* ^4^-(1-Benzylpiperidine-4-yl)-7-chloro-*N* ^2^-(3-(dimethylamino)­propyl)­quinazoline-2,4-diamine (**1g**)

Colorless solid. ^1^H NMR (400 MHz, DMSO *d*_6_) Yield, 57%. ^1^H NMR (400 MHz, DMSO-*d*_6_) δ 8.10 (d, 1H, C*H* quinazoline ring), 7.67 (br s, 1H, N*H*), 7.42–7.30 (m, 5H, 5 x C*H* benzene ring), 7.23 (br s, 1H, C*H* quinazoline ring), 7.06 (d, 1H, C*H* quinazoline ring), 6.71 (br s, 1H, N*H*-CH_2_), 4.19–4.07 (m, 1H, C*H* piperidine ring), 3.56 (s, 2H, NC*H*_2_-Ph), 3.35–3.31 (m, 2H, C*H*_2_NH), 2.92 (d, 2H, 2 x C*H* piperidine ring), 2.30 (t, 2H, C*H*_2_N­(CH_3_)_2_), 2.18 (s, 6H, N­(C*H*_3_)_2_), 2.09 (t, 2H, 2 x C*H* piperidine ring), 1.95 (br s, 2H, 2 x C*H* piperidine ring), 1.72–1.64 (m, 4H, 2 x C*H* piperidine ring + CH_2_C*H*_2_CH_2_); ^13^C NMR (101 MHz, DMSO-*d*_6_) δ 160.6, 159.4, 153.9, 139.0, 137.2, 129.3, 128.6 (2C), 127.4, 125.6, 123.9, 120.0, 102.3, 62.7, 57.6, 52.9 (2C), 47.8, 45.8 (2C), 39.1, 31.8 (2C), 27.7; ESI-HRMS (*m*/*z*): [M + H]^+^ calcd for C_25_H_34_ClN_6_^+^, 453.2528; found, 453.2532.

#### *N* ^4^-(1-Benzylazepan-4-yl)-*N* ^2^-(3-(dimethylamino)-propyl)-7-methoxyquinazoline-2,4-diamine (**1i**)

Colorless solid. Yield 57%. ^1^H NMR (400 MHz, DMSO-*d*_6_) δ 7.91 (d, 1H, C*H* quinazoline ring), 7.37–7.31 (m, 5H, N*H* + 4 x C*H* benzene ring), 7.26–7.22 (m, 1H, C*H* benzene ring), 7.91 (d, 1H, C*H* quinazoline ring), 6.61–6.57 (m, 2H, 2 x C*H* quinazoline ring), 6.36 (br s, 1H, N*H*-CH_2_), 4.42–4.37 (m, 1H, C*H* azepane ring), 3.79 (s, 3H, OC*H*_3_), 3.63 (s, 2H, NC*H*_2_-Ph), 3.33 – 3.27 (m, 2H, C*H*_2_NH), 2.74–2.55 (m, 4H, 4 x C*H* azepane ring), 2.25 (t, 2H, C*H*_2_N­(CH_3_)_2_), 2.12 (s, 6H, N­(C*H*_3_)_2_), 1.99–1.89 (m, 2H, 2 x C*H* azepane ring), 1.85–1.74 (m, 3H, 3 x C*H* azepane ring), 1.69–1.61 (m, 3H, C*H* azepane ring + CH_2_C*H*_2_CH_2_); ^13^C NMR (101 MHz, DMSO-*d*_6_) δ 164.7, 159.01, 158.97, 153.5, 141.8, 131.9 (2C), 131.8 (2C), 129.9, 129.3, 113.64, 113.57, 99.2, 67.5, 65.4, 56.4, 54.6, 53.29, 50.31, 42.5 (2C), 42.4, 25.6, 19.2, 15.6 (2C); ESI-HRMS (*m*/*z*): [M + H]^+^ calcd for C_27_H_39_N_6_O^+^, 463.3180; found, 463.3189.

#### *N* ^2^-(3-(Dimethylamino)­propyl)-7-methoxy-*N* ^4^-(1-(thiophen-3-ylmethyl)­piperidine-4-yl)­quinazoline-2,4-diamine (**1j**)

Colorless solid. Yield 51%.^1^H NMR (400 MHz, DMSO-*d*_6_) δ 7.91 (d, 1H, C*H* quinazoline ring), 7.49 (q, 1H, C*H* thiophene ring), 7.30 (d, 1H, C*H* thiophene ring), 7.25 (br s, 1H, N*H*), 7.06 (d, 1H, C*H* thiophene ring), 6.61–6.57 (m, 2H, 2 x C*H* quinazoline ring), 6.37 (br s, 1H, N*H*-CH_2_), 4.10–4.02 (m, 1H, C*H* piperidine ring), 3.80 (s, 3H, OC*H*_3_), 3.51 (s, 2H, C*H*_2_), 3.30–3.25 (m, 2H, C*H*_2_NH), 2.87 (d, 2H, 2 x C*H* piperidine ring), 2.34 (t, 2H, C*H*_2_N­(CH_3_)_2_), 2.12 (s, 6H, N­(C*H*_3_)_2_), 2.00 (t, 2H, 2 x C*H* piperidine ring), 1.87 (d, 2H, 2 x C*H* piperidine ring), 1.70–1.56 (m, 4H, 2 x C*H* piperidine ring + CH_2_C*H*_2_CH_2_); ^13^C NMR (101 MHz, DMSO-*d*_6_) δ 162.4, 159.9, 158. 9, 139.4, 128.6, 125.9, 124.4, 122.8, 110.3, 105.3, 104.2, 57.2, 56.9, 55.1, 52.4 (2C), 47.1, 45.3 (2C), 38.3, 31.4 (2C), 27.5; ESI-HRMS (*m*/*z*): [M + H]^+^ calcd for C_24_H_35_N_6_OS^+^, 455.2588; found, 455.2594.

#### *N* ^2^-(3-(Dimethylamino)­propyl)-7-methoxy-*N* ^4^-(1-(naphthalene-2-ylmethyl)­piperidine-4-yl)­quinazoline-2,4-diamine (**1k**)

Colorless solid. Yield 64%.^1^H NMR (400 MHz, DMSO-*d*_6_) δ 7.99–7.94 (m, 4H, C*H* quinazoline ring +3 x C*H* naphthalene ring), 7.87 (s, 1H, C*H* naphthalene ring), 7.59–7.53 (m, 3H, 3 x C*H* naphthalene ring), 7.34 (br s, 1H, N*H*), 6.68–6.64 (m, 2H, 2 x C*H* quinazoline ring), 6.44 (br s, 1H, N*H*-CH_2_), 4.20–4.10 (m, 1H, C*H* piperidine ring), 3.86 (s, 3H, OC*H*_3_), 3.74 (s, 2H, C*H*_2_), 3.34 (q, 2H, C*H*_2_NH), 2.98 (d, 2H, 2 x C*H* piperidine ring), 2.31 (t, 2H, C*H*_2_N­(CH_3_)_2_), 2.18–2.13 (m, 8H, 2 x C*H* piperidine ring + N­(C*H*_3_)_2_), 1.96 (d, 2H, 2 x C*H* piperidine ring), 1.75–1.67 (m, 4H, 2 x C*H* piperidine ring + CH_2_C*H*_2_CH_2_); ^13^C NMR (101 MHz, DMSO-*d*_6_) δ 162.8, 160.4, 159.4, 154.8, 136.8, 133.4, 132.7, 128.1, 128.0, 128.0, 127.8, 127.6, 126.5,126.1, 124.8, 110.8, 110.7, 105.7, 62.8, 57.7, 55.5, 53.0 (2C), 47.9, 45.7 (2C), 39.0, 32.0 (2C), 28.0; ESI-HRMS (*m*/*z*): [M + H]^+^ calcd for C_30_H_39_N_6_O^+^, 499.3180; found, 499.3184.

#### *N* ^2^-(3-(Dimethylamino)­propyl)-7-methoxy-*N* ^4^-(1-(pyridin-3-ylmethyl)­piperidine-4-yl)­quinazoline-2,4-diamine (**1l**)

Colorless solid. Yield 56%. ^1^H NMR (400 MHz, DMSO-*d*_6_) δ 8.57 (s, 1H, C*H* pyridine ring), 8.54 (d, 1H, C*H* pyridine ring), 7.96 (d, 1H, C*H* quinazoline ring), 7.78 (d, 1H, C*H* pyridine ring), 7.43 (q, 1H, C*H* pyridine ring), 7.32 (br s, 1H, N*H*), 6.67–6.63 (m, 2H, 2 x quinazoline ring), 6.43 (br s, 1H, N*H*-CH_2_), 4.15–4.06 (m, 1H, C*H* piperidine ring), 3.85 (s, 3H, OC*H*_3_), 3.59 (s, 2H, C*H*_2_), 3.35–3.31 (m, 2H, C*H*_2_NH), 2.91 (d, 2H, 2 x C*H* piperidine ring), 2.30 (t, 2H, C*H*_2_N­(CH_3_)_2_), 2.18 (s, 6H, N­(C*H*_3_)_2_), 2.12 (t, 2H, 2 x C*H* piperidine ring), 1.94 (d, 2H, 2 x C*H* piperidine ring), 1.74–1.64 (m, 4H, 2 x C*H* piperidine ring + CH_2_C*H*_2_CH_2_); ^13^C NMR (101 MHz, DMSO-*d*_6_) δ 162.8, 160.4, 159.4, 154.8, 150.5, 148.7, 137.0, 134.3, 124.8, 123.9, 110.7, 105.6, 105.1, 59.7, 57.7, 55.5, 52.8 (2C), 47.8, 45.8 (2C), 38.5, 31.9 (2C), 28.0; ESI-HRMS (*m*/*z*): [M + H]^+^ calcd for C_25_H_36_N_7_O^+^, 450.2976; found, 450.2980.

#### *N* ^2^-(3-(Dimethylamino)­propyl)-7-methoxy-*N* ^4^-(1-(3-methylbenzyl)­piperidine-4-yl)­quinazoline-2,4-diamine (**1m**)

Colorless solid. Yield 70%. ^1^H NMR (400 MHz, DMSO-*d*_6_) δ 7.96 (d, 1H, C*H* quinazoline ring), 7.33 (br s, 1H, N*H*), 7.27 (t, 1H, C*H* benzene ring), 7.18–7.11 (m, 3H, 3 x C*H* benzene ring), 6.67–6.64 (m, 2H, 2 x C*H* quinazoline ring), 6.45 (br s, 1H, N*H*-CH_2_), 4.18–4.09 (m, 1H, C*H* piperidine ring), 3.85 (s, 3H, OC*H*_3_), 3.67 (s, 2H, C*H*_2_), 3.33 (q, 2H, C*H*_2_NH), 2.91 (d, 2H, 2 x C*H* piperidine ring), 2.37 (s, 3H, Ph–C*H*_3_), 2.30 (t, 2H, C*H*_2_N­(CH_3_)_2_), 2.18 (s, 6H, N­(C*H*_3_)_2_), 2.11 (t, 2H, 2 x C*H* piperidine ring), 1.87 (d, 2H, 2 x C*H* piperidine ring), 1.75–1.63 (m, 4H, 2 x C*H* piperidine ring + CH_2_C*H*_2_CH_2_); ^13^C NMR (101 MHz, DMSO-*d*_6_) δ 162.8, 160.4, 159.4, 154.8, 138.9, 137.6, 129.9, 128.5, 128.0, 126.4, 124.8, 110.8, 105.7, 105.1, 62.7, 57.7, 55.5, 52.9 (2C), 47.9, 45.8 (2C), 39.2, 32.0 (2C), 28.0, 21.5; ESI-HRMS (*m*/*z*): [M + H]^+^ calcd for C_27_H_39_N_6_O^+^, 463.3180; found, 463.3185.

#### *N* ^4^-(1-(3-Chlorobenzyl)­piperidine-4-yl)-*N* ^2^-(3-(dimethylamino)­propyl)-7-methoxyquinazoline-2,4-diamine (**1n**)

Colorless solid. Yield 51%. ^1^H NMR (400 MHz, DMSO-*d*_6_) δ 7.90 (d, 1H, C*H* quinazoline ring), 7.42–7.30 (m, 5H, 4 x C*H* benzene ring + N*H*), 6.62–6.58 (m, 2H, 2 x C*H* quinazoline ring), 6.37 (br s, 1H, N*H*-CH_2_), 4.15–4.01 (m, 1H, C*H* piperidine ring), 3.80 (s, 3H, OC*H*_3_), 3.52 (s, 2H, C*H*_2_), 3.31–3.25 (m, 2H, C*H*_2_NH), 2.85 (d, 2H, 2 x C*H* piperidine ring), 2.25 (t, 2H, C*H*_2_N­(CH_3_)_2_), 2.12 (s, 6H, N­(C*H*_3_)_2_), 2.06 (t, 2H, 2 x C*H* piperidine ring), 1.89 (d, 2H, 2 x C*H* piperidine ring), 1.70–1.58 (m, 4H, 2 x C*H* piperidine ring + CH_2_C*H*_2_CH_2_); ^13^C NMR (101 MHz, DMSO-*d*_6_) δ 162.8, 160.4, 159.4, 154.8, 141.8, 133.4, 130.5, 128.8, 127.8, 127.3, 124.8, 110.7, 105.2, 105.1, 67.5, 61.7, 57.7, 55.5, 52.8 (2C), 45.8 (2C), 38.7, 32.0 (2C), 28.1; ESI-HRMS (*m*/*z*): [M + H]^+^ calcd for C_26_H_36_ClN_6_O^+^, 483.2634; found, 483.2643.

#### *N* ^4^-(1-Benzylpiperidine-4-yl)-*N* ^2^-(3-(dimethylamino)-propyl)-7-ethoxyquinazoline-2,4-diamine (**1o**)

Colorless solid. Yield 68%. ^1^H NMR (400 MHz, DMSO-*d*_6_) δ 7.90 (d, 1H, C*H* quinazoline ring), 7.42–7.30 (m, 6H, 5 x C*H* benzene ring + N*H*), 6.59–6.57 (m, 2H, 2 x C*H* quinazoline ring), 6.37 (br s, 1H, N*H*-CH_2_), 4.09–4.03 (m, 3H, OC*H*_2_+ C*H* piperidine ring), 3.50 (s, 2H, C*H*_2_), 3.26 (t, 2H, C*H*_2_NH), 2.86 (d, 2H, 2 x C*H* piperidine ring), 2.24 (t, 2H, C*H*_2_N­(CH_3_)_2_), 2.12 (s, 6H, N­(C*H*_3_)_2_), 2.03 (t, 2H, 2 x C*H* piperidine ring), 1.92 (d, 2H, 2 x C*H* piperidine ring), 1.68–1.56 (m, 4H, 2 x C*H* piperidine ring + CH_2_C*H*_2_CH_2_), 1.34 (t, 3H, C*H*_3_); ^13^C NMR (101 MHz, DMSO-*d*_6_) δ 162.1, 160.4, 159.4, 154.8, 139.0, 129.3 (2C), 128.6 (2C), 127.3, 124.8, 111.3, 111.0, 105.5, 63.4, 62.7, 57.7, 55.4, 52.9 (2C), 45.8 (2C), 39.0, 31.9 (2C), 28.1, 15.0; ESI-HRMS (*m*/*z*): [M + H]^+^ calcd for C_27_H_39_N_6_O^+^, 463.3180; found, 463.3189.

#### *N*-(1-Benzylpiperidine-4-yl)-2-(4-(dimethylamino)-piperidine-1-yl)-7-ethoxyquinazoline-4-amine (**1q**)

Colorless solid. Yield 82%. ^1^H NMR (400 MHz, DMSO-*d*_6_) δ 7.93 (d, 1H, C*H* quinazoline ring), 7.38–7.23 (m, 6H, 5 x C*H* benzene ring + N*H*), 6.64–6.60 (m, 2H, 2 x C*H* quinazoline ring), 4.75 (d, 2H, 2 x C*H* piperidine ring), 4.07 (q, 2H, OC*H*_2_), 4.03–3.97 (m, 1H, C*H* piperidine ring), 3.51 (s, 2H, C*H*_2_), 2.87–2.68 (m, 4H, 4 x C*H* piperidine ring), 2.33–2.27 (m, 1H, C*H* piperidine ring), 2.09 (s, 6H, N­(C*H*_3_)_2_), 2.06 (t, 2H, 2 x C*H* piperidine ring), 1.93 (d, 2H, 2 x C*H* piperidine ring), 1.77 (d, 2H, 2 x C*H* piperidine ring), 1.61 (qd, 2H, 2 x C*H* piperidine ring), 1.33 (t, 3H, C*H*_3_), 1.31–1.18 (m, 2H, 2 x C*H* piperidine ring); ^13^C NMR (101 MHz, DMSO-*d*_6_) δ 162.2, 159.4, 159.2, 154.7, 139.2, 129.2 (2C), 128.6 (2C), 127.3, 124.8, 111.7, 105.7, 105.0, 63.4, 62.7, 62.5, 52.9 (2C), 48.6, 43.2 (2C), 41.9 (2C), 31.7 (2C), 28.4 (2C), 15.0; ESI-HRMS (*m*/*z*): [M + H]^+^ calcd for C_29_H_41_N_6_O^+^, 489.3336; found, 489.3345.

#### *N*-(1-Benzylpiperidine-4-yl)-7-ethoxy-2-(4-(pyrrolidin-1-yl)­piperidine-1-yl)­quinazoline-4-amine (**1r**)

Colorless solid. Yield 49%. ^1^H NMR (400 MHz, methanol-d_4_) δ 7.81 (d, 1H, C*H* quinazoline ring), 7.39–7.33 (m, 4H, 4 x C*H* benzene ring), 7.32–7.28 (m, 1H, C*H* benzene ring), 6.80 (d, 1H, C*H* quinazoline ring), 6.69 (dd, 1H, C*H* quinazoline ring), 4.82 (br s, 2H, 2 x C*H* piperidine ring), 4.18–4.08 (m, 3H, OC*H*_2_ + C*H* piperidine ring), 3.59 (s, 2H, C*H*_2_), 3.00 (d, 2H 2 x C*H* piperidine ring), 2.89 (t, 2H 2 x C*H* piperidine ring), 2.69 (br s, 4H, 4 x C*H* pyrrolidine ring), 2.40–2.32 (m, 1H, C*H* piperidine ring), 2.21 (t, 2H, 2 x C*H* piperidine ring), 2.10–2.01 (m, 4H, 4 x C*H* piperidine ring), 1.84 (br s, 4H, 4 x C*H* pyrrolidine ring), 1.71 (qd, 2H, 2 x C*H* piperidine ring), 1.52–1.42 (m, 5H, C*H*_3_ + 2 x C*H* piperidine ring); ^13^C NMR (101 MHz, DMSO-*d*_6_) δ 162.2, 159.3, 154.7, 139.2, 129.2 (2C), 128.6 (2C), 127.3, 124.8, 111.7, 105.7, 105.0, 63.4, 62.7, 61.9, 52.9 (2C), 51.2 (2C), 48.6, 42.6 (2C), 31.7 (2C), 31.5 (2C), 23.4 (2C), 15.0; ESI-HRMS (*m*/*z*): [M + H]^+^ calcd for C_31_H_43_N_6_O^+^, 515.3493; found, 515.3499.

#### *N*-(1-Benzylpiperidine-4-yl)-7-ethoxy-2-(4-((4-methoxy-benzyl)­(methyl)­amino)­piperidine-1-yl)­quinazoline-4-amine (**1t**)

Colorless solid. Yield 82%. ^1^H NMR (400 MHz, DMSO-*d*_6_) δ 7.93 (d, 1H, C*H* quinazoline ring), 7.37–7.31 (m, 5H, 4 x C*H* benzene ring + N*H*), 7.29–7.23 (m, 1H, C*H* benzene ring), 7.21 (d, 2H, 2 x C*H* anisole ring), 6.86 (d, 2H, 2 x C*H* anisole ring), 6.65–6.59 (m, 2H, 2 x C*H* quinazoline ring), 4.82 (d, 2H, 2 x C*H* piperidine ring), 4.10–3.95 (m, 3H, OC*H*_2_ + C*H* piperidine ring), 3.73 (s, 3H, OC*H*_3_), 3.49 (s, 4H, 2 x N–C*H*_2_), 2.86 (d, 2H, 2 x C*H* piperidine ring), 2.75 (d, 2H, 2 x C*H* piperidine ring), 2.68–2.61 (m, 1H, C*H* piperidine ring), 2.10–2.03 (m, 5H, NC*H*_3_ + 2 x C*H* piperidine ring), 1.93 (d, 2H, 2 x C*H* piperidine ring), 1.79 (d, 2H, 2 x C*H* piperidine ring), 1.61 (qd, 2H, 2 x C*H* piperidine ring), 1.45–1.33 (m, 5H, C*H*_3_ + 2 x C*H* piperidine ring); ^13^C NMR (101 MHz, DMSO-*d*_6_) δ 162.2, 159.4, 159.2, 158.6, 154.6, 139.1, 132.1, 130.1 (2C), 129.3 (2C), 128.6 (2C), 127.3, 124.8, 113.9 (2C), 111.7, 105.6, 105.0, 63.4, 62.7, 61.1, 57.1, 55.4, 52.9 (2C), 48.6, 43.5 (2C), 37.4, 31.7 (2C), 28.0 (2C), 15.0; ESI-HRMS (*m*/*z*): [M + H]^+^ calcd for C_36_H_47_N_6_O_2_^+^, 595.3755; found, 595.3754.

#### *tert*-Butyl­(1-(4-((1-benzylpiperidine-4-yl)­amino)-7-ethoxyquinazoline-2-yl)­piperidine-4-yl)­(methyl)­carbamate (**25**)

Colorless solid. Yield 81%. ^1^H NMR (400 MHz, DMSO-*d*_6_) δ 8.03 (d, 1H, C*H* quinazoline ring), 7.48 (d, 1H, N*H*), 7.45–7.39 (m, 4H, 4 x C*H* benzene ring), 7.36–7.32 (m, 1H, C*H* benzene ring), 6.74–6.71 (m, 2H, 2 x C*H* quinazoline ring), 4.98 (d, 2H, 2 x C*H* piperidine ring), 4.17 (q, 2H, OC*H*_2_), 4.19–4.05 (m, 2H, 2 x C*H* piperidine ring), 3.58 (s, 2H, C*H*_2_), 2.95 (d, 2H, 2 x C*H* piperidine ring), 2.87 (t, 2H, 2 x C*H* piperidine ring), 2.72 (s, 3H, NC*H*_3_), 2.16 (t, 2H, 2 x C*H* piperidine ring), 2.02 (d, 2H, 2 x C*H* piperidine ring), 1.76–1.64 (m, 6H, 6 x C*H* piperidine ring), 1.50 (s, 9H, COO­(C*H*_3_)_3_), 1.44 (t, 3H, C*H*_3_).

#### *tert*-Butyl­(1-(4-((1-benzylpiperidine-4-yl)­amino)-7-ethoxyquinazoline-2-yl)­piperidine-4-yl)­carbamate (**26**)

Colorless solid. Yield 77%. ^1^H NMR (400 MHz, DMSO-*d*_6_) δ 7.93 (d, 1H, C*H* quinazoline ring), 7.37 (d, 1H, N*H*), 7.35–7.29 (m, 4H, 4 x C*H* benzene ring), 7.28–7.23 (m, 1H, C*H* benzene ring), 6.79 (d, 1H, N*H*), 6.64–6.60 (m, 2H, 2 x C*H* quinazoline ring), 4.65 (d, 2H, 2 x C*H* piperidine ring), 4.08 (q, 2H, OC*H*_2_), 4.03–3.95 (m, 1H, 1 x C*H* piperidine ring), 3.49 (s, 2H, C*H*_2_), 2.92–2.84 (m, 4H, 4 x C*H* piperidine ring), 2.06 (t, 2H, 2 x C*H* piperidine ring), 1.92 (d, 2H, 2 x C*H* piperidine ring), 1.74 (d, 2H, 2 x C*H* piperidine ring), 1.67–1.54 (m, 2H, 2 x C*H* piperidine ring), 1.39 (s, 9H, COO­(C*H*_3_)_3_), 1.35 (t, 3H, C*H*_3_), 1.30–1.24 (m, 3H, 3 x C*H* piperidine ring).

### General Procedure for the Synthesis of Final Compound 1p and Intermediate **27**

A solution of intermediates **25** or **26** (0.104 mmol, 1.0 equiv) in anhydrous methanol (2 mL) was cooled to 0 °C before adding triisopropylsilane (TIPS, 0.125 mmol, 1.2 equiv., 25.5 μL) and 4 N HCl in dioxane (0.912 mL). The reaction mixture was then allowed to warm to rt. After 5 h at rt, the reaction reached completion and was concentrated under reduced pressure. The crude product was triturated with a mixture of diethyl ether (2 mL) and tetrahydrofuran (1 mL) and filtered. The remaining solvent was then completely removed under reduced pressure to afford final compound **1p** and intermediate **27**.

#### *N*-(1-Benzylpiperidine-4-yl)-7-ethoxy-2-(4-(methylamino)­piperidine-1-yl)­quinazoline-4-amine (**1p**)

Colorless solid. Yield 93%. ^1^H NMR (400 MHz, methanol-*d*_4_) δ 8.17 (d, 1H, C*H* quinazoline ring), 7.60 (d, 2H, 2 x C*H* benzene ring), 7.55–7.52 (m, 3H, 3 x C*H* benzene ring), 7.17 (d, 1H, C*H* quinazoline ring), 7.05 (d, 1H, C*H* quinazoline ring), 4.82 (br s, 2H, 2 x C*H* piperidine ring), 4.65 (t, 1H, C*H* piperidine ring), 4.40 (br s, 2H, C*H*_2_), 4.22 (q, 2H, OC*H*_2_), 3.76 (t, 1H, C*H* piperidine ring), 3.69 (q, 1H, C*H* piperidine ring), 3.61–3.41 (m, 5H, 5 x C*H* piperidine ring), 2.79 (s, 3H, NC*H*_3_), 2.35 (d, 4H, 4 x C*H* piperidine ring), 2.09 (q, 2H, 2 x C*H* piperidine ring), 1.75 (qd, 2H, 2 x C*H* piperidine ring), 1.48 (t, 3H, C*H*_3_); ^13^C NMR (101 MHz, methanol-*d*_4_) δ 163.8, 158.9, 151.8, 142.5, 132.0 (2C), 130.4, 129.9, 129.2 (2C), 126.8, 114.3, 103.3, 100.8, 64.6, 60.6, 59.4, 50.8 (2C), 47.6, 43.8 (2C), 29.7 (2C), 28.1 (2C), 27.8, 14.8; ESI-HRMS (*m*/*z*): [M + H]^+^ calcd. for C_28_H_39_N_6_O^+^, 475.3180; found, 475.3181.

#### 2-(4r-Aminopiperidine-1-yl)-*N*-(1-benzylpiperidine-4-yl)-7-ethoxyquinazoline-4-amine trihydrochloride (**27**)

Colorless solid. Yield 85%. ^1^H NMR (400 MHz, DMSO-*d*_6_) δ 12.58 (s, 1H, HCl), 11.19 (s, 1H, HCl), 9.20 (d, 1H, N*H*), 8.59–8.28 (m, 4H, N*H*_3_^+^ + C*H* quinazoline ring), 7.67 (d, 2H, C*H* benzene ring), 7.59 (s, 1H, C*H* benzene ring), 7.48 (s, 3H, C*H* benzene ring + C*H* quinazoline ring), 7.02 (d, 1H, C*H* quinazoline ring), 4.75 (d, 2H, 2 x C*H* piperidine ring), 4.42–4.28 (m, 3H, 3 x C*H* piperidine ring), 4.14 (q, 2H, OC*H*_2_), 3.57 (s, 2H, C*H*_2_), 3.51–3.47 (m, 2H, 2 x C*H* piperidine ring), 3.29–3.15 (m, 3H, 3 x C*H* piperidine ring), 2.18–2.08 (m, 6H, 6 x C*H* piperidine ring), 1.62 (q, 2H, 2 x C*H* piperidine ring), 1.39 (t, 3H, C*H*_3_).

### Procedure for the Synthesis of Compound **1s**

A solution of intermediate **27** (0.14 mmol, 1 equiv) in anhydrous methanol (2.5 mL) and 4 Å molecular sieves was supplemented with 4-methoxybenzaldehyde (0.154 mmol, 1.1 equiv) and glacial acetic acid (8 mL) under constant nitrogen flow at 0 °C. The reaction mixture was then allowed to warm to rt. After stirring for 2.5 h at rt, sodium cyanoborohydride (0.28 mmol, 2 equiv) was added, and the reaction was left to proceed for 25 h. The mixture was then transferred into a solution containing tetrahydrofuran (2 mL), methanol (2 mL), ethyl acetate (15 mL), and a saturated sodium carbonate solution (7 mL). After stirring for 5 min, the organic and aqueous layers were separated, and the aqueous phase was extracted with ethyl acetate (4 × 7 mL). The combined organic extracts were washed with a saturated NaCl solution (2 × 3 mL), dried over anhydrous sodium sulfate, and concentrated under vacuum. The crude product was purified by silica gel column chromatography using a chloroform/methanol/ammonia (32:1:0.1) mixture as the mobile phase to afford final compound **1s**.

#### *N*-(1-Benzylpiperidine-4-yl)-7-ethoxy-2-(4-((4-methoxy-benzyl)­amino)­piperidine-1-yl)­quinazoline-4-amine (**1s**)

Colorless solid. Yield 44%. ^1^H NMR (400 MHz, DMSO-*d*_6_) δ 7.92 (d, 1H, C*H* quinazoline ring), 7.36–7.29 (m, 6H, 5 x C*H* benzene ring + N*H*), 7.26 (d, 2H, 2 x C*H* anisole ring), 6.86 (d, 2H, 2 x C*H* anisole ring), 6.64–6.59 (m, 2H, 2 x C*H* quinazoline ring), 4.57 (d, 2H, 2 x C*H* piperidine ring), 4.07 (q, 2H, OC*H*_2_), 4.02–3.95 (m, 2H, C*H* piperidine ring), 3.73 (s, 3H, OC*H*_3_), 3.68 (s, 2H, NHC*H*_2_), 3.55 (s, 2H, N–C*H*_2_-Ph), 2.95–2.84 (m, 4H, 3 x C*H* piperidine ring + N*H*CH_2_), 2.64–2.58 (m, 1H, C*H* piperidine ring), 2.06 (t, 2H, 2 x C*H* piperidine ring), 1.93 (d, 2H, 2 x C*H* piperidine ring), 1.84 (d, 2H, 2 x C*H* piperidine ring), 1.53 (qd, 2H, 2 x C*H* piperidine ring), 1.35 (t, 3H, C*H*_3_)- 1.36 – 1.22 (m, 3H, 3 x C*H* piperidine ring); ^13^C NMR (101 MHz, DMSO-*d*_6_) δ 162.2, 159.4, 159.3, 158.4, 154.7, 139.2, 133.7, 129.5 (2C), 129.2 (2C), 128.6 (2C), 127.3, 124.8, 113.9 (2C), 111.6, 105.6, 104.9, 63.4, 62.7, 55.5, 54.2, 52.9, 49.6 (2C), 48.6, 42.7 (2C), 32.5 (2C), 31.7 (2C), 15.1; ESI-HRMS (*m*/*z*): [M + H]^+^ calcd for C_35_H_45_N_6_O_2_^+^, 581.3599; found, 581.3605.

### General Procedure for the Synthesis of Intermediates **6** and **8**

The respective substituted quinazoline-2,4­(1*H*,3*H*)-dione (8.32 mmol, 1 equiv) was supplemented with POCl_3_ (138.9 mmol, 16.7 equiv, 12.94 mL) at 0 °C. Then, *N,N*-diethylaniline (9.15 mmol, 1.1 equiv) was added, and the mixture was heated at 150 °C. After 4 h, the crude reaction mixture was transferred dropwise into a flask containing an ice–water mixture (150 mL) and stirred for 30 min. The mixture was then filtered, and the solid was washed with water. The obtained product was transferred to a flask and the solvent was removed under reduced pressure. The crude material was finally purified by silica gel column chromatography with the appropriate acetate/hexane mixture as the mobile phase to afford the intermediates **6** ^43^ and **8**.

#### 2,4-Dichloro-7-ethoxyquinazoline (**8**)

Colorless solid. Yield, 81%. ^1^H NMR (400 MHz, DMSO-*d*_6_) δ 8.18 (d, 1H, C*H* quinazoline ring), 7.50–7.44 (m, 2H, 2 x C*H* quinazoline ring), 4.30 (q, 2H, C*H*_2_), 1.41 (t, 3H, C*H*_3_).

### General Procedure for the Synthesis of Intermediates **16**–**24**

A solution of the respective substituted quinazoline (2.88 mmol, 1 equiv) in anhydrous DMF (11 mL) was supplemented with DIPEA (14.4 mmol, 5 equiv, 2.5 mL) and the appropriate amine (4.61 mmol, 1.6 equiv). The mixture was stirred for 6 h at rt before being diluted with 160 mL of ethyl acetate. The organic phase was then washed with 6 × 15 mL of a saturated NaCl solution, dried over sodium sulfate, filtered, and concentrated under reduced pressure. The crude product was purified by silica gel column chromatography using appropriate mixtures of acetate/hexane as the mobile phase to yield intermediates **16**–**24**.

#### *N*-(1-Benzylpiperidine-4-yl)-2-chloro-6-methoxy-quinazoline-4-amine (**16**)

Colorless solid. Yield, 92%. ^1^H NMR (400 MHz, DMSO-*d*_6_) δ 8.23 (d, 1H, N*H*), 7.74 (d, 1H, C*H* quinazoline ring), 7.55 (d, 1H, C*H* quinazoline ring), 7.42 (dd, 1H, C*H* quinazoline ring), 7.34–7.27 (m, 5H, 5 x C*H* benzene ring), 4.16–4.08 (m, 1H, C*H* piperidine ring), 3.90 (s, 3H, OC*H*_3_), 3.53 (br s, 2H, C*H*_2_), 2.90 (br s, 2H, 2 x C*H* piperidine ring), 2.10 (br s, 2H, 2 x C*H* piperidine ring), 1.95–1.91 (m, 2H, 2 x C*H* piperidine ring), 1.75–1.69 (m, 2H, 2 x C*H* piperidine ring).

#### *N*-(1-Benzylpiperidine-4-yl)-2,7-dichloroquinazoline-4-amine (**17**)

Colorless solid. Yield, 85%. ^1^H NMR (400 MHz, DMSO-*d*_6_) δ 8.55 (br s, 1H, N*H*), 8.39 (d, 1H, C*H* quinazoline ring), 7.68 (d, 1H, C*H* quinazoline ring), 7.60 (dd, 1H, C*H* quinazoline ring), 7.36–7.24 (m, 5H, 5 x C*H* benzene ring), 4.10–4.00 (m, 1H, C*H* piperidine ring), 3.50 (br s, 2H, C*H*_2_), 2.87 (d, 2H, 2 x C*H* piperidine ring), 2.08 (t, 2H, 2 x C*H* piperidine ring), 1.89 (d, 2H, 2 x C*H* piperidine ring), 1.72–1.62 (m, 2H, 2 x C*H* piperidine ring).

#### *N*-(1-Benzylazepan-4-yl)-2-chloro-7-methoxy-quinazoline-4-amine (**18**)

Colorless solid. Yield, 82%. ^1^H NMR (400 MHz, DMSO-*d*_6_) δ 8.27–8.24 (m, 2H, C*H* quinazoline ring + N*H*), 7.37–7.24 (m, 5H, C*H* benzene ring), 7.13 (dd, 1H, C*H* quinazoline ring), 7.03 (d, 1H, C*H* quinazoline ring), 4.42–4.35 (m, 1H, C*H* azepane ring), 3.88 (s, 3H, OC*H*_3_), 3.64 (br s, 2H, C*H*_2_), 2.70–2.60 (m, 4H, 4 x C*H* azepane ring), 1.95–1.90 (m, 2H, 2 x C*H* azepane ring), 1.85–1.76 (m, 3H, 3 x C*H* piperidine ring), 1.64 (br s, 1H, C*H* azepane ring).

#### 2-Chloro-7-methoxy-*N*-(1-(thiophen-3-ylmethyl)-piperidine-4-yl)­quinazoline-4-amine (**19**)

Colorless solid. Yield, 78%. ^1^H NMR (400 MHz, DMSO-*d*_6_) δ 8.31 (d, 1H, C*H* quinazoline ring), 8.20 (d, 1H, N*H*), 7.50 (t, 1H, C*H* thiophene ring), 7.33 (br s, 1H, C*H* thiophene ring), 7.13 (dd, 1H, C*H* quinazoline ring), 7.07 (d, 1H, C*H* thiophene ring), 7.04 (d, 1H, C*H* quinazoline ring), 4.10–4.06 (m, 1H, C*H* piperidine ring), 3.88 (s, 3H, OC*H*_3_), 3.53 (br s, 2H, C*H*_2_), 2.91–2.88 (m, 2H, 2 x C*H* piperidine ring), 2.06 (br s, 2H, 2 x C*H* piperidine ring), 1.90–1.86 (m, 2H, 2 x C*H* piperidine ring), 1.71–1.62 (m, 2H, 2 x C*H* piperidine ring).

#### 2-Chloro-7-methoxy-*N*-(1-(naphthalene-2-ylmethyl)-piperidine-4-yl)­quinazoline-4-amine (**20**)

Colorless solid. Yield, 79%. ^1^H NMR (400 MHz, DMSO-*d*_6_) δ 8.25 (d, 1H, C*H* quinazoline ring), 8.20 (d, 1H, N*H*), 7.91–7.88 (m, 3H, 3 x C*H* naphthalene ring), 7.81 (s, 1H, C*H* naphthalene ring), 7.54–7.45 (m, 3H, 3 x C*H* naphthalene ring), 7.13 (dd, 1H, C*H* quinazoline ring), 7.04 (d, 1H, C*H* quinazoline ring), 4.13–4.07 (m, 1H, C*H* piperidine ring), 3.88 (s, 3H, OC*H*_3_), 3.67 (s, 2H, C*H*_2_), 2.92 (d, 2H, 2 x C*H* piperidine ring), 2.14 (t, 2H, 2 x C*H* piperidine ring), 1.90 (d, 2H, 2 x C*H* piperidine ring), 1.75–1.65 (m, 2H, 2 x C*H* piperidine ring).

#### 2-Chloro-7-methoxy-*N*-(1-(pyridin-3-ylmethyl)-piperidine-4-yl)­quinazoline-4-amine (**21**)

Colorless solid. Yield, 77%. ^1^H NMR (400 MHz, DMSO-*d*_6_) δ 8.52 (s, 1H, C*H* pyridine ring), 8.48 (d, 1H, C*H* pyridine ring), 8.24 (d, 1H, C*H* quinazoline ring), 8.21 (d, 1H, N*H*), 7.73 (d, 1H, C*H* pyridine ring), 7.38 (q, 1H, C*H* pyridine ring), 7.14 (dd, 1H, C*H* quinazoline ring), 7.04 (d, 1H, C*H* quinazoline ring), 4.13–4.07 (m, 1H, C*H* piperidine ring), 3.88 (s, 3H, OC*H*_3_), 3.69 (s, 2H, C*H*_2_), 2.86 (d, 2H, 2 x C*H* piperidine ring), 2.10 (t, 2H, 2 x C*H* piperidine ring), 1.88 (d, 2H, 2 x C*H* piperidine ring), 1.71–1.61 (m, 2H, 2 x C*H* piperidine ring).

#### 2-Chloro-7-methoxy-*N*-(1-(3-methylbenzyl)­piperidine-4-yl)­quinazoline-4-amine (**22**)

Colorless solid. Yield, 75%. ^1^H NMR (400 MHz, DMSO-*d*_6_) δ 8.30 (d, 1H, C*H* quinazoline ring), 8.25 (d, 1H, N*H*), 7.30 (t, 1H, C*H* benzene ring), 7.21–7.09 (m, 5H, 2 x C*H* quinazoline ring +3 x C*H* benzene ring), 4.18–4.07 (m, 1H, C*H* piperidine ring), 3.88 (s, 3H, OC*H*_3_), 3.69 (s, 2H, C*H*_2_), 2.92 (d, 2H, 2 x C*H* piperidine ring), 2.37 (s, 3H, C*H*_3_), 2.11 (t, 2H, 2 x C*H* piperidine ring), 1.93 (d, 2H, 2 x C*H* piperidine ring), 1.77–1.67 (m, 2H, 2 x C*H* piperidine ring).

#### 2-Chloro-*N*-(1-(3-chlorobenzyl)­piperidine-4-yl)-7-methoxyquinazoline-4-amine (**23**)

Colorless solid. Yield, 81%. ^1^H NMR (400 MHz, DMSO-*d*_6_) δ 8.24 (d, 1H, C*H* quinazoline ring), 8.19 (d, 1H, N*H*), 7.39–7.28 (m, 4H, 4 x C*H* benzene ring), 7.13 (dd, 1H, C*H* quinazoline ring), 7.04 (d, 1H, C*H* quinazoline ring), 4.15–4.06 (m, 1H, C*H* piperidine ring), 3.88 (s, 3H, OC*H*_3_), 3.52 (s, 2H, C*H*_2_), 2.85 (d, 2H, 2 x C*H* piperidine ring), 2.10 (t, 2H, 2 x C*H* piperidine ring), 1.89 (d, 2H, 2 x C*H* piperidine ring), 1.73–1.63 (m, 2H, 2 x C*H* piperidine ring).

#### *N*-(1-Benzylpiperidine-4-yl)-2-chloro-7-ethoxy-quinazoline-4-amine (**24**)

Colorless solid. Yield, 87%. ^1^H NMR (400 MHz, DMSO-*d*_6_) δ 8.30 (d, 1H, C*H* quinazoline ring), 8.24 (d, 1H, N*H*), 7.42–7.37 (m, 4H, 4 x C*H* benzene ring), 7.33–7.30 (m, 1H, C*H* benzene ring), 7.18 (dd, 1H, C*H* quinazoline ring), 7.07 (d, 1H, C*H* quinazoline ring), 4.21 (q, 2H, OC*H*_2_), 4.19–4.09 (m, 1H, C*H* piperidine ring), 3.56 (s, 2H, C*H*_2_), 2.92 (d, 2H, 2 x C*H* piperidine ring), 2.13 (t, 2H, 2 x C*H* piperidine ring), 1.93 (d, 2H, 2 x C*H* piperidine ring), 1.76–1.67 (m, 2H, 2 x C*H* piperidine ring), 1.43 (t, 3H, C*H*_3_).

### Procedure for the Synthesis of Intermediate **28**

4-hydroxy-2-nitrobenzoic acid (10.36 mol, 1 equiv) and potassium carbonate (31.07 mol, 3 equiv) were added to anhydrous DMF (12.65 mL), followed by iodoethane (31.07 mol, 3 equiv., 2.50 mL). The reaction mixture was stirred for 24 h at rt and then quenched with ethyl acetate (100 mL). The organic phase was washed with saturated solutions of sodium carbonate (2 × 10 mL) and sodium chloride (4 × 5 mL), then dried over sodium sulfate, filtered, and concentrated under reduced pressure. The crude product was purified by silica gel column chromatography using a 1:10 acetate/hexane mixture as the mobile phase to afford intermediate **28**.

#### Ethyl 4-Ethoxy-2-nitrobenzoate (**28**)

Colorless solid. Yield, 69%. ^1^H NMR (400 MHz, chloroform-d) δ ^1^H NMR (400 MHz, chloroform-d) δ 7.83 (d, 1H, C*H* benzene ring), 7.23 (d, 1H, C*H* benzene ring), 7.10 (dd, 1H, C*H* benzene ring), 4.34 (q, 2H, COOC*H*_2_CH_3_), 4.14 (q, 2H, Ph-OC*H*_2_CH_3_), 1.48 (t, 3H, Ph-OCH_2_C*H*_3_), 1.36 (t, 3H, COOCH_2_C*H*_3_).

### Procedure for the Synthesis of Intermediate **29**

Intermediate **28** (6.86 mol, 1 equiv) was dissolved in an EtOH/AcOH/H_2_O (5:1:1) mixture (34.4 mL). Finely powdered iron (68.64 mol, 10 equiv) was then gradually added, and the reaction mixture was stirred at rt for 50 min. The reaction mixture was then supplemented with ethyl acetate (60 mL) and filtered. The solid over was washed with ethyl acetate (10 × 25 mL), while the organic phase was washed with saturated solutions of sodium carbonate (8 × 10 mL) and sodium chloride (5 × 10 mL). The combined organic layers were dried over sodium sulfate, filtered, and concentrated under reduced pressure. The crude product was purified by silica gel column chromatography using a 1:12 acetate/hexane mixture as the mobile phase, finally yielding intermediate **29**.

#### Ethyl 2-Amino-4-ethoxybenzoate (**29**)

Colorless solid. Yield, 92%. ^1^H NMR (400 MHz, chloroform-d) δ 7.80 (d, 1H, C*H* benzene ring), 6.26 (dd, 1H, C*H* benzene ring), 6.15 (d, 1H, C*H* benzene ring), 5.90 (br s, 2H, NH_2_) 4.32 (q, 2H, COOC*H*_2_CH_3_), 4.04 (q, 2H, Ph-OC*H*_2_CH_3_), 1.44–1.30 (m, 6H, Ph-OCH_2_C*H*_3_ + COOCH_2_C*H*_3_).

### Procedure for the Synthesis of Intermediate **4**

Intermediate **4** was obtained through a two-step process. In the first step, an AcOH/H_2_O (2:1) (24 mL) mixture was added to a flask containing the intermediate **29** (6.29 mol, 1 equiv), and sodium cyanate (15.72 mol, 2.5 equiv) was then added at 0 °C. The reaction mixture was then allowed to warm to rt and stirred for 18 h. In the second step, after the addition of MeOH (24 mL), 8 M NaOH (∼70 mL) was added to reach pH 13. The mixture was then stirred under reflux for 5 h. The mixture was then vacuum filtered, the resulting solid was washed with water and the solvent was removed under reduced pressure to afford intermediate **4**.

#### 7-Ethoxyquinazoline-2,4­(1*H*,3*H*)-dione (**4**)

Colorless solid. Yield, 93%. ^1^H NMR (400 MHz, chloroform-d) δ 7.61 (d, 1H, C*H* benzene ring), 6.44–6.40 (m, 2H, 2 x C*H* benzene ring), 6.15 (d, 1H, C*H* benzene ring), 5.90 (br s, 2H, NH_2_),4.02 (q, 2H, C*H*_2_), 1.33 (t, 3H, C*H*_3_).

### General Procedure for the Synthesis of Intermediates **30**–**32**

A solution of *N*-*tert*-butoxycarbonyl-piperidine (2.0 mmol, 1 equiv) in anhydrous DCM (20 mL) was prepared under a nitrogen atmosphere. The appropriate aldehyde (2.8 mmol, 1.4 equiv) and 4 Å molecular sieves (1.43 g) were then added, and the mixture was stirred for 45 min at rt. Sodium triacetoxyborohydride (STAB) (3 mmol, 1.5 equiv) was then added at 0 °C using an ice bath. The reaction mixture was then allowed to warm to rt and stirred for 23 h. The reaction was quenched by adding ethyl acetate (25 mL), and the organic phase was washed with saturated solutions of sodium carbonate (3 × 3 mL) and sodium chloride (1 × 2 mL). The organic phase was dried over sodium sulfate, filtered, and concentrated under reduced pressure. The crude product was then purified by silica gel column chromatography using the appropriate mixtures of chloroform/methanol/ammonia as the mobile phase, yielding intermediates **30**–**32**.

#### *tert*-Butyl­(1-(thiophen-3-ylmethyl)­piperidine-4-yl)-carbamate (**30**)

Colorless solid. Yield, 72%. ^1^H NMR (400 MHz, DMSO-*d*_6_) δ 7.47 (dd, 1H, C*H* thiophene ring), 7.27 (d, 1H, thiophene ring), 7.02 (d, 1H, C*H* thiophene ring), 6.74 (d, 1H, N*H*), 3.42 (s, 2H, C*H*_2_), 3.25–3.16 (m, 1H, C*H* piperidine ring), 2.74 (d, 2H, 2 x C*H* piperidine ring), 1.90 (t, 2H, 2 x C*H* piperidine ring), 1.66 (d, 2H, 2 x C*H* piperidine ring), 1.37–1.30 (m, 11H, 2 x C*H* piperidine ring +3 x C*H*_3_).

#### *tert*-Butyl­(1-(naphthalene-2-ylmethyl)­piperidine-4-yl)-carbamate (**31**)

Colorless solid. Yield, 75%. ^1^H NMR (400 MHz, DMSO-*d*_6_) δ 7.90–7.85 (m, 3H, 3 x C*H* naphthalene ring), 7.77 (s, 1H, C*H* naphthalene ring), 7.49 (br s, 3H, 3 x C*H* naphthalene ring), 6.77 (d, 1H, N*H*), 3.59 (s, 2H, C*H*_2_), 3.29–3.19 (m, 1H, C*H* piperidine ring), 2.79 (d, 2H, 2 x C*H* piperidine ring), 2.03 (t, 2H, 2 x C*H* piperidine ring), 1.68 (d, 2H, 2 x C*H* piperidine ring), 1.45–1.30 (m, 11H, 2 x C*H* piperidine ring +3 x C*H*_3_).

#### *tert*-Butyl­(1-(pyridin-3-ylmethyl)­piperidine-4-yl)-carbamate (**32**)

Colorless solid. Yield, 79%. ^1^H NMR (400 MHz, DMSO-*d*_6_) δ 8.52 (s, 1H, C*H* pyridine ring), 8.51 (s, 1H, C*H* pyridine ring), 7.74 (d, 1H, C*H* pyridine ring), 7.41 (dd, 1H, C*H* pyridine ring), 6.81 (d, 1H, N*H*), 3.52 (s, 2H, C*H*_2_), 3.31–3.21 (m, 1H, C*H* piperidine ring), 2.78 (d, 2H, 2 x C*H* piperidine ring), 2.03 (t, 2H, 2 x C*H* piperidine ring), 1.73 (d, 2H, 2 x C*H* piperidine ring), 1.48–1.35 (m, 11H, 2 x C*H* piperidine ring +3 x C*H*_3_).

### General Procedure for the Synthesis of Intermediates **33**–**35**

A solution of intermediates **30**–**32** (1.324 mmol, 1 equiv) in anhydrous methanol (25 mL) was cooled to 0 °C before adding TIPS (1.588 mmol, 1.2 equiv, 323 μL) and HCl (4 M) in dioxane (9.93 mL). The reaction mixture was then allowed to warm to rt and stirred for 6 h. After completion, the solvent was evaporated under reduced pressure and washed with anhydrous diethyl ether (4 × 5 mL) until a solid was obtained. The suspension was then concentrated under reduced pressure and triturated with anhydrous diethyl ether (13 mL). The mixture was then vacuum filtered, and solvent traces were removed under reduced pressure to afford the hydrochloride salts **33**–**35**.

#### 1-(Thiophen-3-ylmethyl)­piperidine-4-amine Dihydrochloride (**33**)

Colorless solid. Yield, 92%. ^1^H NMR (400 MHz, DMSO-*d*_6_) δ 11.12 (br s, HCl), 8.48–8.35 (m, 3H, N*H*_2_ + HCl), 7.80 (s, 1H, C*H* thiophene ring), 7.65 (s, 1H, thiophene ring), 7.38 (d, 1H, C*H* thiophene ring), 4.33–4.24 (m, 2H, C*H*_2_), 3.29–3.24 (m, 3H, 3 x C*H* piperidine ring), 2.95 (d, 2H, 2 x C*H* piperidine ring), 2.10 (d, 2H, 2 x C*H* piperidine ring), 1.99–1.91 (m, 2H, 2 x C*H* piperidine ring).

#### 1-(Naphthalene-2-ylmethyl)­piperidine-4-amine Dihydrochloride (**34**)

Colorless solid. Yield, 87%. ^1^H NMR (400 MHz, DMSO-*d*_6_) δ 11.19 (br s, HCl), 8.51–8.37 (m, 3H, N*H*_2_ + HCl), 8.22–8.04 (m, 3H, 3 x C*H* naphthalene ring), 7.98 (s, 1H, C*H* naphthalene ring), 7.85 (br s, 3H, 3 x C*H* naphthalene ring), 4.39 (s, 2H, C*H*_2_), 3.55–3.44 (m, 2H, C*H* piperidine ring), 3.25–3.20 (d, 1H, 2 x C*H* piperidine ring), 2.78 (t, 2H, 2 x C*H* piperidine ring), 2.11 (d, 2H, 2 x C*H* piperidine ring), 1.99–1.88 (m, 2H, 2 x C*H* piperidine ring).

#### 1-(Pyridin-3-ylmethyl)­piperidine-4-amine Trihydrochloride (**35**)

Colorless solid. Yield, 91%. ^1^H NMR (400 MHz, DMSO-*d*_6_) δ 11.03 (br s, HCl), 8.50 (br s, 4H, N*H*_2_ + 2 x HCl), 8.46 (s, 1H, C*H* pyridine ring), 8.44 (s, 1H, C*H* pyridine ring), 7.88 (d, 1H, C*H* pyridine ring), 7.35 (dd, 1H, C*H* pyridine ring), 4.43 (s, 2H, C*H*_2_), 3.57–3.48 (m, 2H, 2 x C*H* piperidine ring), 3.36–3.28 (d, 1H, C*H* piperidine ring), 3.17 (t, 2H, 2 x C*H* piperidine ring), 2.13 (d, 2H, 2 x C*H* piperidine ring), 2.07–1.98 (m, 2H, 2 x C*H* piperidine ring).

### General Procedure for the Synthesis of Intermediates **11**–**13**

The hydrochloride salts **33**–**35** (0.638 mmol) were dissolved in a saturated solution of sodium carbonate (11.65 mL) at 0 °C, and the reaction was stirred at rt for 10 min. This was followed by the addition of a chloroform/isopropanol (4:1) mixture (3 mL). After 5 min, the reaction mixture was extracted with chloroform/isopropanol (4:1) (10 × 3 mL), and the organic phase was dried over sodium sulfate, filtered, and concentrated under reduced pressure to afford intermediates **11**–**13** as free amines, which were immediately used for the following reactions.

### Procedure for the Synthesis of Intermediate **37**

*N*-*tert*-butoxycarbonyl-4-(methylamino)­piperidine (2.33 mmol, 1 equiv) was dissolved in anhydrous DCM (10 mL) with 4 Å molecular sieves (1.67 g). 4-methoxybenzaldehyde (3.27 mmol, 1.4 equiv) was then added under a nitrogen atmosphere at rt. After 1 h, STAB (3.50 mmol, 1.5 equiv) was added at 0 °C, and the reaction mixture was allowed to warm to rt. After stirring for 21 h at rt, ethyl acetate (50 mL) was added to the reaction mixture, and the organic phase was washed with saturated sodium carbonate (3 × 4 mL) and sodium chloride (2 × 3 mL) solutions. The organic phase was dried over sodium sulfate, filtered, and concentrated under reduced pressure. The crude product was purified by silica gel column chromatography using a chloroform/methanol (99:1) mixture as the mobile phase to afford intermediate **37**.

#### *tert*-Butyl-4-((4-methoxybenzyl)­(methyl)­amino)-piperidine-1-carboxylate (**37**)

Colorless solid. Yield, 78%. ^1^H NMR (400 MHz, chloroform-d) δ 7.15 (d, 2H, 2 x C*H* benzene ring), 6.78 (d, 2H, 2 x C*H* benzene ring), 4.09 (br s, 2H, C*H* piperidine ring), 3.73 (s, 3H, OC*H*_3_), 3.45 (s, 2H, C*H*_2_), 2.61 (t, 2H, 2 x C*H* piperidine ring), 2.50 (t, 1H, C*H* piperidine ring), 2.11 (s, 3H, NC*H*_3_), 1.72 (d, 2H, 2 x C*H* piperidine ring), 1.48–1.38 (m, 11H, 2 x C*H* piperidine ring +3 x C*H*_3_).

### Procedure for the Synthesis of Intermediate **38**

Intermediate **37** (1.79 mmol, 1 equiv), TIPS (2.15 mmol, 1.2 equiv, 440 μL), and 4 N HCl in dioxane (13.5 mL) were added to anhydrous methanol (11 mL) at 0 °C, and the mixture was allowed to reach rt. After stirring for 6 h at rt, the solvent was evaporated under reduced pressure, and the crude product was washed with anhydrous diethyl ether (4 × 5 mL) until a solid was obtained. The suspension was then concentrated under reduced pressure and triturated with anhydrous diethyl ether (10 mL). The mixture was then vacuum filtered, and solvent traces were removed under reduced pressure to afford *N*-(4-methoxybenzyl)-*N*-methylpiperidine-4-amine hydrochloride salt (**38**).

#### N-(4-Methoxybenzyl)-*N*-methylpiperidine-4-amine Dihydrochloride (**38**)

Colorless solid. Yield, 85%. ^1^H NMR (400 MHz, DMSO-*d*_6_) δ 10.99 (br s, HCl), 9.13 (d, 2H, N*H* + HCl), 7.58 (d, 2H, 2 x C*H* benzene ring), 7.00 (d, 2H, 2 x C*H* benzene ring), 4.36–4.10 (m, 2H, 2 x C*H* piperidine ring), 3.80 (s, 3H, OC*H*_3_), 3.42–3.38 (m, 3H, C*H*_2_ + C*H* piperidine ring), 2.90 (br s, 2H, 2 x C*H* piperidine ring), 2.53 (s, 3H, NC*H*_3_), 2.31 (dd, 2H, 2 x C*H* piperidine ring), 2.09–2.01 (m, 2H, 2 x C*H* piperidine ring).

### Procedure for the Synthesis of Intermediate **36**

Intermediate **38** (1.622 mmol) was dissolved in a saturated solution of sodium carbonate (29 mL) at 0 °C, and the reaction was stirred at rt for 10 min. This was followed by the addition of a chloroform/isopropanol (4:1) mixture (7 mL). After 5 min, the reaction mixture was extracted with chloroform/isopropanol (4:1) (10 × 7 mL), and the organic phase was dried over sodium sulfate, filtered, and concentrated under reduced pressure to afford intermediate **36**, which was immediately used for the following reaction.

#### Procedures for the Synthesis of Compounds 1c, 1d, 2a–2s, and Related Intermediates

##### General Procedure A

The dichloro derivative (1.0 mmol, 1.0 equiv) was dissolved in *N*-methyl-2-pyrrolidone (NMP, 4 mL), and the corresponding amine (1.2 mmol, 1.2 equiv) and *N*,*N*-diisopropylethylamine (DIPEA, 5.0 mmol, 5.0 equiv) were added at rt. The reaction mixture was stirred overnight at 150 °C in an oil bath. The resulting solution was heated to 150 °C in an oil bath and stirred at this temperature overnight. After completion of the reaction (monitored by TLC), the mixture was cooled down to rt and water was added to the reaction mixture. The product was extracted three times using diethyl ether, and the combined organic layers were dried over sodium sulfate. After filtration, the solvents were evaporated under vacuum. The resulting crude product was purified by flash column chromatography (DCM:MeOH, 0 to 3%) to give the corresponding products.

##### General Procedure B

The corresponding amine (1.5 mmol, 1.5 equiv) and aqueous solution (4 M) of HCl (0.26 mL, 1.0 equiv) were added at rt to a stirring solution of the chloride (1.0 mmol, 1.0 equiv) in isopropanol (*i*-PrOH) (4 mL). The resulting mixture was heated to 160 °C under microwave irradiation for 3 h. Upon completion of the reaction, the solvents were evaporated under vacuum, and the resulting crude product was purified by flash column chromatography (DCM/MeOH/NH_4_OH (aqueous, 25%), 100:0:1 to 100:5:1) to give the corresponding products.

##### General Procedure C

Malonic acid (8.45 g, 81.2 mmol, 1.0 equiv) and the corresponding aniline (81.2 mmol, 1.0 equiv) were added to a two-neck flask fitted with a reflux condenser. Phosphorus oxychloride (11.4 mL, 122 mmol, 1.5 equiv) was then added in portions at rt. After gas evolution ceased, the slurry was slowly heated to 95 °C and stirred for 30 min. The resultant foam was then cooled to rt and phosphorus oxychloride (22.8 mL, 244 mmol, 3.0 equiv) was added. The mixture was heated to 120 °C and stirred for 3 h at this temperature. After cooling in an ice bath, the reaction mixture was slowly added to an ice–water mixture to quench the remaining phosphorus oxychloride, followed by 5 N NaOH solution, until the solution reached pH = 8. The mixture was diluted with ethyl acetate, and the organic layer was collected. The organic phase was washed with water and brine. After drying over sodium sulfate and being concentrated, the crude residue was purified by flash column chromatography (DCM) to provide the corresponding dichloro quinoline derivatives. General procedure **C** is based on a reported synthesis.^40^

##### General Procedure D

Potassium carbonate (5.0 mmol, 5.0 equiv) was added to a stirring solution of 4-(*tert*-butoxycarbonylamino)­piperidine (1.0 mmol, 1.0 equiv) and alkyl bromide (1.1 mmol, 1.1 equiv) in dimethylformamide (DMF) (2.0 mL), and the reaction mixture was stirred overnight at rt. After completion of the reaction, water was added, and the product was extracted using DCM. After the organic layer was washed three times with water, it was dried over sodium sulfate, and filtered. After evaporation of the solvent under reduced pressure, the residue was purified by flash column chromatography (DCM/MeOH, 0 to 2%) to afford the corresponding products.

##### General Procedure E

Trifluoroacetic acid (TFA) (4 mL) was added to a solution of the corresponding amine (1.0 mmol, 1.0 equiv) in DCM (4 mL) at rt, and the reaction mixture was stirred overnight. After completion of the reaction, the solution was removed under reduced pressure to afford a crude product. A saturated sodium hydrogen carbonate solution was added to the crude product, which was then extracted with DCM. The organic layer was dried over sodium sulfate, and filtered. The solvent was removed by evaporation under vacuum to afford the products as TFA salts in quantitative yields.

#### *N*-(1-Benzylpiperidine-4-yl)-2-chloroquinazoline-4-amine (**1d**)

Compound **1d** was prepared following the general synthetic procedure **A** and was isolated as a colorless solid (80% yield). ^1^H NMR (500 MHz, chloroform-d) δ 7.77–7.70 (m, 2H), 7.66 (d, *J =* 8.1 Hz, 1H), 7.44 (ddd, *J =* 8.2, 6.7, 1.5 Hz, 1H), 7.33 (d, *J =* 4.4 Hz, 4H), 7.28 (dd, *J =* 8.3, 4.1 Hz, 1H), 5.79 (d, *J =* 7.2 Hz, 1H), 4.31 (tdt, *J =* 11.6, 8.4, 4.3 Hz, 1H), 3.57 (s, 2H), 2.91 (d, *J =* 12.0 Hz, 2H), 2.27 (td, *J =* 11.7, 2.2 Hz, 2H), 2.16–2.10 (m, 2H), 1.66 (qd, *J =* 11.4, 3.8 Hz, 2H); ^13^C NMR (126 MHz, chloroform-d) δ 160.3, 157.9, 151.0, 138.0, 133.5, 129.3, 128.4, 128.0, 127.3, 126.2, 120.7, 113.3, 63.1, 52.2, 48.3, 32.0; ESI-HRMS (*m*/*z*): [M + H]^+^ calcd for C_20_H_22_ClN_4_^+^, 353.1528; found, 353.1530. The analytical data are in accordance with previously reported literature.^38^

#### *N* ^4^-(1-Benzylpiperidine-4-yl)-*N* ^2^-(3-(dimethylamino)-propyl)­quinazoline-2,4-diamine (**1c**)

Compound **1c** was prepared following the general synthetic procedure **B** and was isolated as a colorless solid (68% yield). ^1^H NMR (500 MHz, methanol-*d*_4_) δ 8.16 (d, *J =* 8.0 Hz, 1H), 7.72 (t, *J =* 7.7 Hz, 1H), 7.46 (d, *J =* 7.6 Hz, 3H), 7.43 – 7.31 (m, 4H), 4.40 (s, 1H), 3.87 (s, 2H), 3.63 (t, *J =* 6.4 Hz, 2H), 3.20 (d, *J =* 12.3 Hz, 2H), 3.11–3.06 (m, 2H), 2.79 (s, 6H), 2.61 (t, *J =* 11.6 Hz, 2H), 2.14 (s, 2H), 2.08 (p, *J =* 6.8 Hz, 2H), 2.00–1.89 (m, 2H); ^13^C NMR (126 MHz, methanol-d_4_) δ 161.4, 136.0, 135.6, 131.3, 129.6, 129.3, 124.6, 119.9, 63.0, 56.8, 53.1, 49.9, 43.8, 43.6, 39.3, 35.9, 35.4, 31.0, 26.6, 26.3; ESI-HRMS (*m*/*z*): [M + H]^+^ calcd for C_25_H_35_N_6_^+^, 419.2918; found, 419.2923. The analytical data are in accordance with previously reported literature.^38^

#### *N* ^4^-(1-Benzylpiperidine-4-yl)-*N* ^2^-(3-(dimethylamino)-propyl)-7-methoxyquinoline-2,4-diamine (**2a**)

Intermediate **48** was prepared following the general synthetic procedure **C** and was isolated as a colorless solid (58% yield). ^1^H NMR (300 MHz, chloroform-d) δ 8.07 (d, *J =* 9.2 Hz, 1H), 7.41–7.35 (m, 2H), 7.30–7.27 (m, 1H), 3.95 (s, 3H). The analytical data are in accordance with previously reported literature.^40^

Intermediate **50** was prepared following the general synthetic procedure **A** and was isolated as a colorless solid (40% yield). ^1^H NMR (400 MHz, chloroform-d) δ 7.51 (d, *J =* 9.2 Hz, 1H), 7.34 (d, *J =* 4.4 Hz, 4H), 7.30–7.27 (m, 1H), 7.24 (d, *J =* 2.6 Hz, 1H), 7.05 (dd, *J =* 9.1, 2.6 Hz, 1H), 6.32 (s, 1H), 4.88 (d, *J =* 7.4 Hz, 1H), 3.90 (s, 3H), 3.57 (s, 2H), 3.50 (qd, *J =* 10.6, 5.4 Hz, 1H), 2.90 (dd, *J =* 12.1, 3.3 Hz, 2H), 2.25 (td, *J =* 11.7, 2.3 Hz, 2H), 2.18–2.08 (m, 2H)-, 1.68 (dd, *J =* 10.5, 3.3 Hz, 2H); ESI-HRMS (*m*/*z*): [M + H]^+^ calcd. for C_22_H_25_ClN_3_O^+^, 382.1681; found, 382.1682.

Compound **2a** was prepared following the general synthetic procedure **B** and was isolated as a colorless solid (79% yield). ^1^H NMR (400 MHz, methanol-*d*_4_) δ 7.73 (d, *J =* 9.1 Hz, 1H), 7.35–7.23 (m, 5H), 6.93 (d, *J =* 2.6 Hz, 1H), 6.72 (dd, *J =* 9.1, 2.6 Hz, 1H), 5.67 (s, 1H), 3.84 (s, 3H), 3.56 (s, 2H), 3.45 (td, *J =* 10.7, 5.5 Hz, 1H), 3.39 (t, *J =* 6.9 Hz, 2H), 2.94 (d, *J =* 12.2 Hz, 2H), 2.47–2.40 (m, 2H), 2.25 (s, 6H), 2.24–2.16 (m, 2H), 2.06 (d, *J =* 11.5 Hz, 2H), 1.81 (p, *J =* 6.9 Hz, 2H), 1.73–1.60 (m, 2H); ^13^C NMR (101 MHz, methanol-d_4_) δ 162.3, 160.7, 151.4, 150.8, 138.5, 130.8, 129.3, 128.4, 123.1, 112.2, 111.6, 106.0, 85.5, 64.0, 58.3, 55.6, 53.5, 50.8, 45.5, 40.8, 32.2, 28.5; ESI-HRMS (*m*/*z*): [M + H]^+^ calcd for C_27_H_38_N_5_O^+^, 448.3071; found, 448.3070.

#### *N* ^4^-(1-Benzylpiperidine-4-yl)-*N* ^2^-(3-(dimethylamino)-propyl)-7-ethoxyquinoline-2,4-diamine (**2b**)

Intermediate **48** was prepared by following the synthetic procedure for compound **2a**.

Intermediate **39** was prepared following a reported procedure.^40^ 2,4-Dichloro-7-methoxyquinoline (**48**) (342 mg, 1.50 mmol) was dissolved in sulfuric acid (3.4 mL) and heated in a 160 °C oil bath for 2 h. The mixture was cooled to rt, poured into ice-cold water to form a precipitate and extracted with ethyl acetate. The organic phase was washed with water, and then with saturated aqueous NaHCO_3_ solution. The organic layer was dried (MgSO_4_), filtered and concentrated. The crude mixture was purified by column chromatography (DCM/MeOH/NH_4_OH = 15:0.1:0.1) to afford the title compound as a colorless solid (94% yield). ^1^H NMR (300 MHz, dichloromethane-d_2_) δ 8.10–8.01 (m, 1H), 7.33 (s, 1H), 7.25 (dd, *J =* 9.0, 2.4 Hz, 1H), 7.19–7.17 (m, 1H); ESI-HRMS (*m*/*z*): [M + H]^+^ calcd for C_9_H_6_ONCl_2_^+^, 213.9821; found, 213.9819. The analytical data are in accordance with previously reported literature.^40^

Intermediate **40** was prepared following the general synthetic procedure **D** and was isolated as a colorless solid (93% yield). ^1^H NMR (400 MHz, chloroform-d) δ 8.06 (dd, *J =* 9.2, 0.4 Hz, 1H), 7.35 (s, 1H), 7.33 (d, *J =* 2.7 Hz, 1H), 7.26 (dd, *J =* 9.2, 2.5 Hz, 1H), 4.17 (q, *J =* 7.0 Hz, 2H), 1.49 (t, *J =* 7.0 Hz, 3H). ESI-HRMS (*m*/*z*): [M + H]^+^ calcd for C_11_H_10_ONCl_2_^+^, 242.0134; found, 242.0137.

Intermediate **44** was prepared following the general synthetic procedure **A** and was isolated as a colorless solid (43% yield). ^1^H NMR (400 MHz, chloroform-d) δ 7.51 (d, *J =* 9.2 Hz, 1H), 7.33 (d, *J =* 4.4 Hz, 4H), 7.27 (d, *J =* 4.2 Hz, 1H), 7.22 (d, *J =* 2.6 Hz, 1H), 7.05 (dd, *J =* 9.1, 2.6 Hz, 1H), 6.32 (s, 1H), 4.87 (d, *J =* 7.4 Hz, 1H), 4.13 (q, *J =* 7.0 Hz, 2H), 3.56 (s, 2H), 3.49 (dq, *J =* 10.3, 6.8, 5.0 Hz, 1H), 2.95–2.85 (m, 2H), 2.29–2.20 (m, 2H), 2.17–2.07 (m, 2H), 1.65 (s, 2H), 1.46 (t, *J =* 7.0 Hz, 3H); ESI-HRMS (*m*/*z*): [M + H]^+^ calcd. for C_23_H_27_ClN_3_O^+^, 396.1837; found, 396.1839.

Compound **2b** was prepared following the general synthetic procedure **B** and was isolated as a colorless solid (65% yield). ^1^H NMR (500 MHz, methanol-d_4_) δ 7.73 (d, *J =* 9.0 Hz, 1H), 7.36–7.25 (m, 5H), 6.91 (d, *J =* 2.5 Hz, 1H), 6.71 (dd, *J =* 9.0, 2.5 Hz, 1H), 5.66 (s, 1H), 4.08 (q, *J =* 7.0 Hz, 2H), 3.57 (s, 2H), 3.44 (dd, *J =* 9.5, 5.5 Hz, 1H), 3.38 (t, *J =* 6.9 Hz, 2H), 2.95 (d, *J =* 12.0 Hz, 2H), 2.47–2.41 (m, 2H), 2.25 (s, 6H), 2.21 (dd, *J =* 11.7, 2.4 Hz, 2H), 2.07 (d, *J =* 11.9 Hz, 2H), 1.81 (dt, *J =* 14.4, 7.1 Hz, 2H), 1.68 (qd, *J =* 11.7, 3.7 Hz, 2H), 1.41 (t, *J =* 7.0 Hz, 3H); ^13^C NMR (126 MHz, methanol-d_4_) δ 161.6, 160.7, 151.4, 138.5, 130.8, 129.3, 128.5, 123.1, 112.6, 106.6, 85.5, 64.4, 64.0, 58.3, 53.5, 50.8, 45.5, 40.8, 32.2, 28.5, 15.1; ESI-HRMS (*m*/*z*): [M + H]^+^ calcd. for C_28_H_40_N_5_O^+^, 462.3227; found, 462.3228.

#### *N* ^4^-(1-Benzylpiperidine-4-yl)-*N* ^2^-(3-(dimethylamino)-propyl)-7-isopropoxyquinoline-2,4-diamine (**2c**)

Intermediate **39** was prepared by following the synthetic procedure for compound **2b**.

Intermediate **41** was prepared starting from intermediate **39** following the general synthetic procedure **D** and was isolated as a colorless solid (86% yield). ^1^H NMR (400 MHz, chloroform-d) δ 7.97 (d, *J =* 9.2 Hz, 1H), 7.26 (d, *J =* 1.8 Hz, 2H), 7.16 (dd, *J =* 9.2, 2.5 Hz, 1H), 4.65 (pd, *J =* 6.1, 0.6 Hz, 1H), 1.35 (d, *J =* 6.1 Hz, 6H); APCI-HRMS (*m*/*z*): [M + H]^+^ calcd for C_12_H_12_Cl_2_NO^+^, 256.0290; found, 256.0293.

Intermediate **45** was prepared following the general synthetic procedure **A** and was isolated as a colorless solid (42% yield). ^1^H NMR (500 MHz, chloroform-d) δ 7.50 (d, *J =* 9.2 Hz, 1H), 7.36–7.31 (m, 4H), 7.30–7.26 (m, 1H), 7.22 (d, *J =* 2.5 Hz, 1H), 7.01 (dd, *J =* 9.1, 2.6 Hz, 1H), 6.30 (s, 1H), 4.87 (d, *J =* 7.5 Hz, 1H), 4.67 (hept, *J =* 6.0 Hz, 1H), 3.56 (s, 2H), 3.54–3.45 (m, 1H), 2.94–2.87 (m, 2H), 2.24 (td, *J =* 11.4, 2.6 Hz, 2H), 2.15–2.08 (m, 2H), 1.63 (dtd, *J =* 14.0, 10.7, 3.7 Hz, 2H), 1.39 (d, *J =* 6.1 Hz, 6H); ESI-HRMS (*m*/*z*): [M + H]^+^ calcd for C_24_H_29_ClN_3_O^+^, 410.1994; found, 410.1998.

Compound **2c** was prepared following the general synthetic procedure **B** and was isolated as a colorless solid (56% yield). ^1^H NMR (500 MHz, methanol-*d*_4_) δ 7.72 (d, *J =* 9.1 Hz, 1H), 7.35–7.30 (m, 4H), 7.28–7.24 (m, 1H), 6.92 (d, *J =* 2.5 Hz, 1H), 6.69 (dd, *J =* 9.0, 2.5 Hz, 1H), 5.66 (s, 1H), 4.65 (p, *J =* 6.0 Hz, 1H), 3.56 (s, 2H), 3.47–3.41 (m, 1H), 3.38 (t, *J =* 6.9 Hz, 2H), 2.95 (d, *J =* 12.1 Hz, 2H), 2.47–2.40 (m, 2H), 2.25 (s, 6H), 2.21 (t, *J =* 11.8 Hz, 2H), 2.07 (d, *J =* 11.8 Hz, 3H), 1.81 (dt, *J =* 14.5, 7.1 Hz, 2H), 1.72–1.60 (m, 2H), 1.34 (d, *J =* 6.0 Hz, 6H); ^13^C NMR (126 MHz, methanol-*d*_4_) δ 160.8, 160.4, 151.3, 138.5, 130.8, 129.3, 128.5, 123.1, 113.4, 111.6, 108.1, 85.5, 70.8, 64.0, 58.4, 53.5, 50.8, 49.5, 45.5, 40.8, 32.2, 28.5, 22.4; ESI-HRMS (*m*/*z*): [M + H]^+^ calcd for C_29_H_42_N_5_O^+^, 476.3384; found, 476.3391.

#### *N* ^4^-(1-Benzylpiperidine-4-yl)-7-cyclobutoxy-*N* ^2^-(3-(dimethylamino)­propyl)­quinoline-2,4-diamine (**2d**)

Intermediate **39** was prepared following the synthetic procedure for compound **2b**.

Intermediate **42** was prepared starting from intermediate **39** following general synthetic procedure **D** and was directly subjected to the next synthesis step without further purification.

Intermediate **46** was prepared following the general synthetic procedure **A** and was isolated as a colorless oil (32% yield). ^1^H NMR (500 MHz, chloroform-d) δ 7.51 (d, *J =* 9.2 Hz, 1H), 7.37–7.27 (m, 5H), 7.09 (d, *J =* 2.5 Hz, 1H), 7.02 (dd, *J =* 9.1, 2.6 Hz, 1H), 6.31 (s, 1H), 4.86 (d, *J =* 7.4 Hz, 1H), 4.74 (p, *J =* 7.1 Hz, 1H), 3.56 (s, 2H), 3.49 (dp, *J =* 14.4, 5.4, 4.1 Hz, 1H), 2.90 (d, *J =* 12.0 Hz, 2H), 2.59–2.50 (m, 2H), 2.27–2.17 (m, 4H), 2.15–2.09 (m, 2H), 1.94–1.84 (m, 1H), 1.77–1.69 (m, 1H), 1.66–1.60 (m, 2H); ESI-HRMS (*m*/*z*): [M + H]^+^ calcd for C_25_H_29_ClN_3_O^+^, 422.1994; found, 422.1997.

Compound **2d** was prepared following the general synthetic procedure **B** and was isolated as a colorless solid (31% yield). ^1^H NMR (500 MHz, methanol-*d*_4_) δ 7.75 (d, *J =* 9.0 Hz, 1H), 7.35–7.31 (m, 4H), 7.26 (td, *J =* 6.3, 5.9, 2.3 Hz, 1H), 6.83 (d, *J =* 2.5 Hz, 1H), 6.69 (dd, *J =* 9.0, 2.5 Hz, 1H), 5.66 (s, 1H), 4.72 (q, *J =* 7.1 Hz, 1H), 3.56 (s, 2H), 3.44 (dd, *J =* 9.5, 5.3 Hz, 1H), 3.39 (t, *J =* 6.9 Hz, 2H), 2.95 (d, *J =* 12.1 Hz, 2H), 2.55–2.47 (m, 2H), 2.44 (d, *J =* 7.7 Hz, 2H), 2.26 (s, 6H), 2.24–2.18 (m, 2H), 2.14 (td, *J =* 7.3, 3.7 Hz, 2H), 2.06 (d, *J =* 12.3 Hz, 2H), 1.88–1.77 (m, 3H), 1.75–1.64 (m, 3H); ^13^C NMR (126 MHz, methanol-*d*_4_) δ 160.3, 160.1, 151.7, 149.5, 138.5, 130.8, 129.3, 128.5, 123.4, 113.0, 111.4, 106.8, 85.1, 72.9, 64.0, 58.1, 53.5, 50.9, 45.4, 40.8, 32.1, 31.6, 28.4, 14.1; ESI-HRMS (*m*/*z*): [M + H]^+^ calcd for C_30_H_42_N_5_O^+^, 488.3384; found, 488.3390.

#### *N* ^4^-(1-Benzylpiperidine-4-yl)-7-(cyclopentyloxy)-*N* ^2^-(3-(dimethylamino)­propyl)­quinoline-2,4-diamine (**2e**)

Intermediate **39** was prepared by following the synthetic procedure for compound **2b**.

Intermediate **43** was prepared starting from intermediate **39** following the general synthetic procedure **D** and was isolated as a colorless oil (61% yield). ^1^H NMR (500 MHz, chloroform-d) δ 8.04 (d, *J =* 9.1 Hz, 1H), 7.34 (s, 1H), 7.32 (d, *J =* 2.5 Hz, 1H), 7.22 (dd, *J =* 9.2, 2.5 Hz, 1H), 4.90 (tt, *J =* 5.8, 2.7 Hz, 1H), 2.03–1.96 (m, 2H), 1.96–1.89 (m, 2H), 1.87–1.78 (m, 2H), 1.71–1.62 (m, 2H); APCI-HRMS (*m*/*z*): [M + H]^+^ calcd for C_14_H_14_Cl_2_NO^+^, 282.0447; found, 282.0448.

Intermediate **47** was prepared following the general synthetic procedure **A** and was isolated as a colorless oil (31% yield). ^1^H NMR (500 MHz, chloroform-d) δ 7.49 (d, *J =* 9.2 Hz, 1H), 7.33 (d, *J =* 4.4 Hz, 4H), 7.29 (d, *J =* 4.3 Hz, 1H), 7.21 (d, *J =* 2.5 Hz, 1H), 7.01 (dd, *J =* 9.1, 2.6 Hz, 1H), 6.31 (s, 1H), 4.86 (dd, *J =* 5.7, 2.5 Hz, 1H), 3.56 (s, 2H), 3.49 (dh, *J =* 14.3, 4.0 Hz, 1H), 2.90 (d, *J =* 11.9 Hz, 2H), 2.24 (t, *J =* 10.5 Hz, 2H), 2.17–2.06 (m, 2H), 1.98 (dq, *J =* 13.9, 7.8, 7.0 Hz, 2H), 1.93–1.86 (m, 2H), 1.83–1.76 (m, 2H), 1.66–1.61 (m, 4H); ESI-HRMS (*m*/*z*): [M + H]^+^ calcd. for C_26_H_31_ClN_3_O^+^, 436.2150; found, 436.2150.

Compound **2e** was prepared following the general synthetic procedure **B** and was isolated as a colorless solid (75% yield). ^1^H NMR (400 MHz, methanol-*d*_4_) δ 7.71 (d, *J =* 9.0 Hz, 1H), 7.37–7.22 (m, 5H), 6.91 (d, *J =* 2.5 Hz, 1H), 6.67 (dd, *J =* 9.0, 2.5 Hz, 1H), 5.66 (s, 1H), 4.85 (tt, *J =* 6.1, 2.6 Hz, 1H), 3.57 (s, 2H), 3.49–3.41 (m, 1H), 3.38 (t, *J =* 6.9 Hz, 2H), 2.95 (d, *J =* 12.2 Hz, 2H), 2.47–2.40 (m, 2H), 2.25 (s, 6H), 2.20 (dd, *J =* 12.0, 2.2 Hz, 2H), 2.07 (d, *J =* 10.5 Hz, 2H), 2.00–1.92 (m, 2H), 1.83 (tdd, *J =* 14.6, 7.5, 3.9 Hz, 6H), 1.73–1.61 (m, 4H); ^13^C NMR (101 MHz, methanol-*d*_4_) δ 160.8, 160.6, 151.4, 150.8, 138.5, 130.8, 129.3, 128.4, 123.0, 113.4, 111.4, 108.0, 85.5, 80.4, 64.0, 58.4, 53.5, 50.8, 45.5, 40.8, 33.9, 32.2, 28.5, 25.0; ESI-HRMS (*m*/*z*): [M + H]^+^ calcd for C_31_H_44_N_5_O^+^, 502.3540; found, 502.3538.

#### *N* ^4^-(1-Benzylpiperidine-4-yl)-7-(cyclohexyloxy)-*N* ^4^-(3-(dimethylamino)­propyl)­quinoline-2,4-diamine (**2f**)

Intermediate **39** was prepared following the synthetic procedure for compound **2b**.

Intermediate **39** (300 mg, 1.41 mmol, 1 equiv), cyclohexanol (0.150 mL, 1.41 mmol, 1.0 equiv), triphenylphosphine (481 mg, 1.83 mmol, 1.3 equiv), and THF (9 mL) were added to a round-bottom flask. Diisopropylazodicarboxylate (DIAD) (0.300 mL, 1.55 mmol, 1.1 equiv) was added dropwise to the reaction mixture over the course of 2 min at rt. Then, the reaction mixture was warmed up to 80 °C and stirred at this temperature for 36 h. Upon reaction completion, the reaction solvent was evaporated under reduced pressure, and the residue was purified by flash column chromatography (cyclohexane:ethyl acetate, 0 to 1%) to give the corresponding intermediate **53** as a colorless oil (253 mg, 61% yield). ^1^H NMR (500 MHz, chloroform-d) δ 8.05 (d, *J =* 9.2 Hz, 1H), 7.35–7.32 (m, 2H), 7.25 (s, 1H), 4.43 (td, *J =* 9.1, 4.5 Hz, 1H), 2.09 (dd, *J =* 13.1, 3.8 Hz, 2H), 1.88–1.79 (m, 2H), 1.66–1.56 (m, 3H), 1.46–1.31 (m, 3H); ESI-HRMS (*m*/*z*): [M + H]^+^ calcd for C_15_H_16_Cl_2_NO^+^, 296.0603; found, 296.0604.

Intermediate **55** was prepared following the general synthetic procedure **A** and was isolated as a colorless oil (37% yield). ^1^H NMR (500 MHz, chloroform-d) δ 7.72–7.63 (m, 1H), 7.56–7.46 (m, 2H), 7.34 (d, *J =* 4.4 Hz, 3H), 7.24 (d, *J =* 2.6 Hz, 1H), 7.03 (dd, *J =* 9.1, 2.6 Hz, 1H), 6.30 (s, 1H), 4.86 (d, *J =* 7.4 Hz, 1H), 4.37 (ddd, *J =* 12.9, 9.1, 3.7 Hz, 1H), 3.56 (s, 2H), 3.49 (ddt, *J =* 14.3, 10.8, 5.3 Hz, 1H), 2.90 (d, *J =* 12.0 Hz, 2H), 2.24 (t, *J =* 11.3 Hz, 2H), 2.16–2.04 (m, 4H), 1.85–1.76 (m, 2H), 1.67–1.60 (m, 3H), 1.52 (dd, *J =* 16.3, 7.0 Hz, 2H), 1.42–1.26 (m, 3H); ESI-HRMS (*m*/*z*): [M + H]^+^ calcd for C_27_H_33_ClN_3_O^+^, 450.2307; found, 450.2308.

Compound **2f** was prepared following the general synthetic procedure **B** and was isolated as a colorless solid (42% yield). ^1^H NMR (500 MHz, methanol-*d*_4_) δ 7.73 (d, *J =* 9.0 Hz, 1H), 7.35–7.31 (m, 4H), 7.28–7.24 (m, 1H), 6.94 (d, *J =* 2.5 Hz, 1H), 6.71 (dd, *J =* 9.0, 2.5 Hz, 1H), 5.66 (s, 1H), 4.36 (tt, *J =* 8.7, 3.6 Hz, 1H), 3.56 (s, 2H), 3.45 (dq, *J =* 10.7, 6.7, 5.3 Hz, 1H), 3.38 (t, *J =* 6.9 Hz, 2H), 2.94 (d, *J =* 12.1 Hz, 2H), 2.49–2.41 (m, 2H), 2.25 (s, 6H), 2.23–2.15 (m, 2H), 2.09–2.01 (m, 4H), 1.84–1.75 (m, 4H), 1.71–1.64 (m, 2H), 1.63–1.57 (m, 1H), 1.56–1.49 (m, 2H), 1.43 (dtd, *J =* 13.4, 7.1, 3.5 Hz, 2H), 1.35 (ddt, *J =* 13.6, 6.2, 3.1 Hz, 1H); ^13^C NMR (126 MHz, methanol-*d*_4_) δ 160.5, 160.3, 151.4, 150.4, 138.5, 130.8, 129.3, 128.5, 123.2, 113.6, 111.5, 107.9, 85.4, 76.3, 64.0, 58.3, 53.5, 50.8, 45.5, 40.8, 32.9, 32.2, 28.5, 26.8, 24.8; ESI-HRMS (*m*/*z*): [M + H]^+^ calcd for C_32_H_46_N_5_O^+^, 516.3697; found, 516.3699.

#### *N* ^4^-(1-Benzylpiperidine-4-yl)-*N* ^2^-(3-(dimethylamino)-propyl)-7-phenoxyquinoline-2,4-diamine (**2g**)

Intermediate **49** was prepared following the general synthetic procedure **C** and was isolated as a colorless oil (45% yield). ^1^H NMR (500 MHz, chloroform-d) δ 8.16 (d, *J =* 9.2 Hz, 1H), 7.47–7.41 (m, 3H), 7.39 (s, 1H), 7.32 (d, *J =* 2.5 Hz, 1H), 7.26–7.22 (m, 1H), 7.15–7.11 (m, 2H); ESI-HRMS (*m*/*z*): [M + H]^+^ calcd for C_15_H_10_Cl_2_NO^+^, 290.0134; found, 290.0141.

Intermediate **51** was prepared following the general synthetic procedure **A** and was isolated as a colorless solid (28% yield). ^1^H NMR (500 MHz, chloroform-d) δ 7.60 (d, *J =* 9.1 Hz, 1H), 7.41–7.36 (m, 2H), 7.34 (d, *J =* 4.4 Hz, 4H), 7.30–7.26 (m, 2H), 7.22–7.15 (m, 2H), 7.10 (d, *J =* 7.7 Hz, 2H), 6.34 (s, 1H), 4.90 (d, *J =* 7.3 Hz, 1H), 3.58 (s, 2H), 3.55 – 3.46 (m, 1H), 2.91 (d, *J =* 12.0 Hz, 2H), 2.31–2.22 (m, 2H), 2.18–2.06 (m, 2H), 1.73–1.60 (m, 2H); ESI-HRMS (*m*/*z*): [M + H]^+^ calcd for C_27_H_27_ClN_3_O^+^, 444.1837; found, 444.1837.

Compound **2g** was prepared following the general synthetic procedure **B** and was isolated as a colorless solid (67% yield). ^1^H NMR (500 MHz, methanol-*d*_4_) δ 7.83 (d, *J =* 9.0 Hz, 1H), 7.40–7.23 (m, 7H), 7.13 (t, *J =* 7.4 Hz, 1H), 7.06–7.02 (m, 2H), 6.92 (d, *J =* 2.5 Hz, 1H), 6.80 (dd, *J =* 9.0, 2.5 Hz, 1H), 5.70 (s, 1H), 3.57 (s, 2H), 3.47–3.42 (m, 1H), 3.36–3.33 (m, 2H), 2.95 (d, *J =* 12.2 Hz, 2H), 2.45–2.35 (m, 2H), 2.23 (s, 8H), 2.08 (d, *J =* 13.5 Hz, 2H), 1.81–1.75 (m, 2H), 1.69 (tt, *J =* 11.5, 6.5 Hz, 2H); ^13^C NMR (126 MHz, methanol-*d*_4_) δ 161.0, 160.2, 158.1, 151.1, 150.9, 138.5, 130.9, 130.8, 129.3, 128.5, 124.8, 123.6, 120.7, 113.6, 113.5, 112.4, 86.4, 64.0, 58.3, 53.5, 50.8, 45.4, 40.7, 32.1, 28.5; ESI-HRMS (*m*/*z*): [M + H]^+^ calcd for C_32_H_40_N_5_O^+^, 510.3227; found, 510.3230.

#### 7-(Benzyloxy)-*N* ^4^-(1-benzylpiperidine-4-yl)-*N* ^2^-(3-(dimethylamino)­propyl)­quinoline-2,4-diamine (**2h**)

Intermediate **39** was prepared following the synthetic procedure for compound **2b**.

A suspension of intermediate **39** (400 mg, 1.88 mmol, 1.0 equiv) and NaH (60% dispersion in mineral oil) (189 mg, 4.70 mmol, 2.5 equiv) in anhydrous DMF (5 mL) was stirred for 1 h at 0 °C under argon. Benzyl bromide (0.830 mL, 7.00 mmol, 3.7 equiv) was added, and the reaction was warmed up to rt and stirred at this temperature until complete consumption of the starting material (monitored by TLC). The mixture was extracted with ethyl acetate and washed with water and brine. The organic solution was dried over sodium sulfate, filtered, and concentrated under reduced pressure. The residue was purified by flash column chromatography (cyclohexane/ethyl acetate, 0 to 1%) to afford intermediate **52** as a colorless solid (427 mg, 75% yield). ^1^H NMR (500 MHz, chloroform-d) δ 8.07 (d, *J =* 9.2 Hz, 1H), 7.47 (d, *J =* 7.3 Hz, 2H), 7.43–7.38 (m, 3H), 7.35 (td, *J =* 6.1, 5.5, 3.2 Hz, 3H), 5.20 (s, 2H); ESI-HRMS (*m*/*z*): [M + H]^+^ calcd for C_16_H_12_Cl_2_NO^+^, 304.0290; found, 304.0292. The analytical data are in accordance with previously reported literature.^44^

Intermediate **54** was prepared following the general synthetic procedure **A** and was isolated as a colorless solid (37% yield). ^1^H NMR (500 MHz, chloroform-d) δ 7.53 (d, *J =* 9.2 Hz, 1H), 7.46 (d, *J =* 7.2 Hz, 2H), 7.39 (t, *J =* 7.4 Hz, 2H), 7.36–7.31 (m, 5H), 7.28 (dt, *J =* 8.9, 4.3 Hz, 1H), 7.14 (dd, *J =* 9.1, 2.6 Hz, 1H), 6.32 (s, 1H), 5.16 (s, 2H), 4.88 (d, *J =* 7.4 Hz, 1H), 3.57 (s, 2H), 3.50 (d, *J =* 7.1 Hz, 1H), 2.91 (d, *J =* 11.9 Hz, 2H), 2.24 (t, *J =* 11.3 Hz, 2H), 2.13 (d, *J =* 14.1 Hz, 2H), 1.69–1.61 (m, 2H); ESI-HRMS (*m*/*z*): [M + H]^+^ calcd for C_28_H_29_ClN_3_O^+^, 458.1994; found, 458.1992.

Compound **2h** was prepared following the general synthetic procedure **B** and was isolated as a colorless solid (80% yield). ^1^H NMR (400 MHz, methanol-*d*_4_) δ 8.07 (d, *J =* 9.2 Hz, 1H), 7.48–7.31 (m, 10H), 7.21 (d, *J =* 2.4 Hz, 1H), 7.07 (dd, *J =* 9.2, 2.5 Hz, 1H), 5.88 (s, 1H), 5.22 (s, 2H), 3.85 (s, 2H), 3.76 (t, *J =* 10.5 Hz, 1H), 3.56 (t, *J =* 6.7 Hz, 2H), 3.22–3.12 (m, 3H), 3.05–2.98 (m, 2H), 2.69 (s, 6H), 2.63 (t, *J =* 9.9 Hz, 1H), 2.14 (d, *J =* 13.4 Hz, 2H), 2.08–2.00 (m, 2H), 1.92–1.81 (m, 2H); ^13^C NMR (101 MHz, methanol-*d*_4_) δ 163.2, 155.6, 153.9, 141.0, 137.7, 131.3, 129.7, 129.7, 129.4, 129.2, 128.6, 125.1, 115.3, 110.0, 102.6, 82.9, 71.5, 63.1, 56.4, 52.9, 50.7, 44.1, 40.4, 30.9, 26.3; ESI-HRMS (*m*/*z*): [M + H]^+^ calcd for C_33_H_42_N_2_O^+^, 524.3384; found, 524.3388.

#### *N* ^4^-(1-Benzylpiperidine-4-yl)-7-ethoxy-*N* ^2^-(1-methylpiperidine-4-yl)­quinoline-2,4-diamine (**2i**)

Intermediate **44** was prepared by following the synthetic procedure for compound **2b**.

Compound **2i** was prepared starting from intermediate **44** following the general synthetic procedure **B** and was isolated as a colorless solid (84% yield). ^1^H NMR (400 MHz, methanol-*d*_4_) δ 7.70 (d, *J =* 9.0 Hz, 1H), 7.36–7.22 (m, 5H), 6.93 (d, *J =* 2.5 Hz, 1H), 6.70 (dd, *J =* 9.0, 2.6 Hz, 1H), 5.67 (s, 1H), 4.09 (q, *J =* 7.0 Hz, 2H), 3.86 (td, *J =* 10.4, 5.2 Hz, 1H), 3.56 (s, 2H), 3.42 (ddd, *J =* 14.2, 10.3, 3.8 Hz, 1H), 2.94 (d, *J =* 12.2 Hz, 2H), 2.85 (d, *J =* 11.9 Hz, 2H), 2.29 (s, 3H), 2.25–2.14 (m, 4H), 2.03 (d, *J =* 13.7 Hz, 4H), 1.74–1.60 (m, 2H), 1.54 (q, *J =* 10.5 Hz, 2H), 1.41 (t, *J =* 7.0 Hz, 3H); ^13^C NMR (101 MHz, methanol-d_4_) δ 161.5, 160.1, 151.2, 151.1, 138.6, 130.7, 129.3, 128.4, 122.9, 112.6, 111.7, 106.9, 86.5, 64.4, 64.0, 55.6, 53.5, 50.8, 46.3, 33.3, 32.2, 15.1; ESI-HRMS (*m*/*z*): [M + H]^+^ calcd for C_29_H_40_N_5_O^+^, 474.3227; found, 474.3227.

#### *N*-(1-Benzylpiperidine-4-yl)-7-ethoxy-2-(1*H*-imidazol-1-yl)­quinoline-4-amine (**2j**)

Intermediate **44** was prepared by following the synthetic procedure for compound **2b**.

Compound **2j** was prepared starting from intermediate **44** following the general synthetic procedure **B** and was isolated as a yellow/green oil (85% yield). ^1^H NMR (500 MHz, dichloromethane-*d*_2_) δ 8.36 (t, *J =* 1.1 Hz, 1H), 7.75 (t, *J =* 1.4 Hz, 1H), 7.63 (d, *J =* 9.2 Hz, 1H), 7.36–7.31 (m, 4H), 7.28–7.24 (m, 1H), 7.22 (d, *J =* 2.6 Hz, 1H), 7.15–7.14 (m, 1H), 7.05 (dd, *J =* 9.1, 2.6 Hz, 1H), 6.39 (s, 1H), 5.13 (d, *J =* 7.5 Hz, 1H), 4.17 (q, *J =* 7.0 Hz, 2H), 3.65–3.57 (m, 1H), 3.55 (s, 2H), 2.93–2.87 (m, 2H), 2.29–2.22 (m, 2H), 2.18–2.13 (m, 2H), 1.72–1.63 (m, 2H), 1.47 (t, *J =* 7.0 Hz, 3H); ^13^C NMR (126 MHz, dichloromethane-d_2_) δ 161.1, 151.5, 150.0, 150.0, 135.6, 130.3, 129.4, 121.1, 117.1, 117.0, 112.5, 109.1, 64.2, 63.3, 52.5, 50.3, 32.4; ESI-HRMS (*m*/*z*): [M + H]^+^ calcd for C_26_H_30_N_5_O^+^, 428.2445; found, 428.2437.

#### *N*-(1-Benzylpiperidine-4-yl)-7-ethoxy-2-(4-(methylamino)­piperidine-1-yl)­quinoline-4-amine (**2k**)

Intermediate **44** was prepared following the synthetic procedure for compound **2b**.

Compound **2k** was prepared starting from intermediate **44** following the general synthetic procedure **B** and was isolated as a colorless solid (43% yield). ^1^H NMR (500 MHz, methanol-*d*_4_) δ 7.78 (d, *J =* 9.1 Hz, 1H), 7.36–7.30 (m, 4H), 7.28–7.24 (m, 1H), 6.95 (d, *J =* 2.5 Hz, 1H), 6.77 (dd, *J =* 9.1, 2.5 Hz, 1H), 5.90 (s, 1H), 4.34 (d, *J =* 13.3 Hz, 2H), 4.10 (q, *J =* 7.0 Hz, 2H), 3.60–3.51 (m, 3H), 2.99–2.88 (m, 4H), 2.64 (ddd, *J =* 10.9, 6.8, 4.1 Hz, 1H), 2.41 (s, 3H), 2.26 (td, *J =* 11.8, 2.0 Hz, 2H), 2.10–1.96 (m, 4H), 1.70 (qd, *J =* 13.4, 3.6 Hz, 2H), 1.41 (t, *J =* 7.0 Hz, 4H); ^13^C NMR (126 MHz, methanol-d_4_) δ 161.8, 160.5, 152.5, 138.5, 130.8, 129.3, 128.5, 123.2, 113.7, 111.0, 106.5, 84.9, 64.5, 64.0, 58.1, 53.4, 50.6, 49.6, 46.3, 32.8, 32.3, 32.1, 15.1; ESI-HRMS (*m*/*z*): [M + H]^+^ calcd for C_29_H_40_N_5_O^+^, 474.3227; found, 474.3223.

#### *N*-(1-Benzylpiperidine-4-yl)-2-(4-(dimethylamino)-piperidine-1-yl)-7-ethoxyquinoline-4-amine (**2l**)

Intermediate **44** was prepared following the synthetic procedure for compound **2b**.

Compound **2l** was prepared starting from intermediate **44** following the general synthetic procedure **B** and was isolated as a colorless solid (87% yield). ^1^H NMR (500 MHz, methanol-*d*_4_) δ 7.96 (d, *J =* 9.1 Hz, 1H), 7.37–7.31 (m, 4H), 7.27 (ddd, *J =* 8.5, 5.5, 2.2 Hz, 1H), 7.01 (d, *J =* 2.4 Hz, 1H), 6.91 (dd, *J =* 9.1, 2.4 Hz, 1H), 5.72 (s, 1H), 4.13 (q, *J =* 7.0 Hz, 2H), 3.60 (s, 2H), 3.53 (t, *J =* 6.6 Hz, 3H), 2.98 (dq, *J =* 14.5, 7.0 Hz, 6H), 2.24 (t, *J =* 11.8 Hz, 2H), 2.06 (d, *J =* 12.4 Hz, 2H), 1.99 (p, *J =* 6.8 Hz, 2H), 1.75 (qd, *J =* 12.8, 3.7 Hz, 2H), 1.43 (t, *J =* 7.0 Hz, 3H), 1.26 (t, *J =* 7.3 Hz, 3H); ^13^C NMR (126 MHz, methanol-*d*_4_) δ 163.1, 153.3, 138.3, 130.8, 129.4, 128.5, 124.5, 114.4, 110.3, 103.0, 83.2, 65.1, 63.9, 53.3, 51.4, 49.6, 45.7, 43.9, 39.6, 31.8, 28.1, 15.0, 12.2; ESI-HRMS (*m*/*z*): [M + H]^+^ calcd for C_28_H_40_N_5_O^+^, 462.3227; found, 462.3219.

#### *N*-(1-Benzylpiperidine-4-yl)-7-ethoxy-2-(4-(pyrrolidin-1-yl)­piperidine-1-yl)­quinoline-4-amine (**2m**)

Intermediate **44** was prepared following the synthetic procedure for compound **2b**.

Compound **2m** was prepared starting from intermediate **44** following the general synthetic procedure **B** and was isolated as a colorless solid (83% yield). ^1^H NMR (400 MHz, methanol-*d*_4_) δ 7.78 (d, *J =* 9.1 Hz, 1H), 7.34–7.29 (m, 4H), 7.28–7.22 (m, 1H), 6.95 (d, *J =* 2.5 Hz, 1H), 6.77 (dd, *J =* 9.1, 2.5 Hz, 1H), 5.89 (s, 1H), 4.34 (d, *J =* 13.3 Hz, 2H), 4.09 (q, *J =* 7.0 Hz, 2H), 3.55 (s, 3H), 2.98–2.85 (m, 4H), 2.64 (s, 4H), 2.31–2.18 (m, 3H), 2.03 (t, *J =* 13.1 Hz, 4H), 1.80 (t, *J =* 3.2 Hz, 4H), 1.73–1.63 (m, 2H), 1.60–1.47 (m, 2H), 1.40 (t, *J =* 7.0 Hz, 3H); ^13^C NMR (101 MHz, methanol-d_4_) δ 161.8, 160.6, 152.3, 150.1, 138.5, 130.8, 129.3, 128.4, 123.1, 113.7, 111.1, 106.8, 85.0, 64.5, 64.0, 63.8, 53.3, 52.4, 50.6, 46.3, 32.2, 32.1, 24.0, 15.1; ESI-HRMS (*m*/*z*): [M + H]^+^ calcd for C_32_H_44_N_5_O^+^, 514.3540; found, 514.3544.

#### *N*-(1-Benzylpiperidine-4-yl)-7-ethoxy-2-(4-((4-methoxy-benzyl)­amino)­piperidine-1-yl)­quinoline-4-amine (**2n**)

Intermediate **44** was prepared following the synthetic procedure for compound **2b**.

(4-Methoxyphenyl)­methanamine (5.5 mmol, 1.1 equiv) and acetic acid (0.75 mL) were added to a solution of the *N*-(*tert*-butoxycarbonyl)-4-piperidone (1.0 g, 5.0 mmol, 1.0 equiv) in methanol (25 mL). Sodium cyanoborohydride (0.63 g, 10 mmol, 2.0 equiv) was added portionwise to the reaction mixture. After stirring overnight at rt, the solvent was evaporated under a vacuum, and NaOH solution (2 M) and DCM were added to the residue. The organic phase was washed with brine, dried over sodium sulfate, and filtered, and the resulting crude mixture was purified by flash column chromatography (DCM:MeOH, 0 to 3%) to give intermediate **56** as a colorless oil (1.5 g, 92% yield). The analytical data are in accordance with the previously reported literature.^45^

Intermediate **57** was prepared following the general synthetic procedure **E** and was isolated as the TFA salt as a colorless oil (quantitative yield). ^1^H NMR (400 MHz, chloroform-d) δ 7.23 (d, *J =* 8.6 Hz, 2H), 6.87–6.84 (m, 2H), 3.80 (d, *J =* 1.6 Hz, 4H), 3.75 (s, 2H), 3.11 (dt, *J =* 12.9, 3.7 Hz, 2H), 2.65–2.58 (m, 3H), 1.91 (d, *J =* 4.1 Hz, 4H), 1.35–1.25 (m, 2H); ESI-HRMS (*m*/*z*): [M + H]^+^ calcd for C_13_H_21_N_2_O^+^, 221.1648; found, 221.1648.

Compound **2n** was prepared starting from intermediates **44** and **57** following the general synthetic procedure **B** and was isolated as a colorless solid (76% yield). ^1^H NMR (500 MHz, methanol-*d*_4_) δ 7.74 (d, *J =* 9.1 Hz, 1H), 7.33 (q, *J =* 7.9, 7.3 Hz, 4H), 7.28–7.21 (m, 3H), 6.92 (d, *J =* 2.5 Hz, 1H), 6.89–6.85 (m, 2H), 6.73 (dd, *J =* 9.1, 2.6 Hz, 1H), 5.89 (s, 1H), 4.34 (d, *J =* 13.2 Hz, 2H), 4.08 (q, *J =* 7.0 Hz, 2H), 3.77 (s, 3H), 3.73 (s, 2H), 3.56 (s, 2H), 3.54–3.49 (m, 1H), 2.96–2.85 (m, 4H), 2.71 (tt, *J =* 10.8, 3.9 Hz, 1H), 2.24 (td, *J =* 11.7, 1.9 Hz, 2H), 2.10–1.94 (m, 4H), 1.72–1.62 (m, 2H), 1.41 (q, *J =* 6.8 Hz, 5H); ^13^C NMR (126 MHz, methanol-d_4_) δ 161.5, 161.4, 160.4, 152.0, 151.2, 138.5, 132.8, 130.8, 130.7, 129.7, 129.3, 128.5, 122.9, 114.9, 114.9, 113.4, 111.3, 107.3, 85.3, 64.4, 64.0, 55.7, 53.4, 50.6, 50.5, 46.3, 46.1, 32.8, 32.2, 15.1; ESI-HRMS (*m*/*z*): [M + H]^+^ calcd for C_36_H_46_N_5_O_2_^+^, 580.3646; found, 580.3652.

#### *N*-(1-Benzylpiperidine-4-yl)-7-ethoxy-2-(4-((4-methoxy-benzyl)­(methyl)­amino)­piperidine-1-yl)­quinoline-4-amine (**2o**)

Intermediate **44** was prepared following the synthetic procedure for compound **2b**.

Intermediate **56** was prepared by following the synthetic procedure for compound **2n**.

Paraformaldehyde (290 mg, 9.60 mmol, 10 equiv) and acetic acid (3 mL) were added to a solution of the intermediate **56** (300 mg, 0.940 mmol, 1.0 equiv) in acetonitrile (3 mL). Sodium cyanoborohydride (304 mg, 4.80 mmol, 5.0 equiv) was added portionwise to the reaction mixture. After stirring overnight at rt, the solvent was evaporated under vacuum, and NaOH solution (2 M) and DCM were added to the residue. The organic phase was washed with brine, dried over sodium sulfate, and filtered, and the resulting crude mixture was purified by flash column chromatography (DCM/MeOH, 0 to 2%) to give intermediate **58** as a colorless oil (274 mg, 87% yield). ^1^H NMR (400 MHz, chloroform-d) δ 7.24–7.18 (m, 2H), 6.87–6.81 (m, 2H), 4.15 (d, *J =* 12.9 Hz, 2H), 3.80 (s, 3H), 3.52 (s, 2H), 2.68 (t, *J =* 12.3 Hz, 2H), 2.56 (tt, *J =* 11.4, 3.5 Hz, 1H), 2.18 (s, 3H), 1.79 (d, *J =* 12.7 Hz, 2H), 1.51 (td, *J =* 12.1, 4.4 Hz, 2H), 1.46 (s, 9H); ESI-HRMS (*m*/*z*): [M + H]^+^ calcd for C_19_H_31_N_2_O_3_^+^, 335.2329; found, 335.2335.

Intermediate **59** was prepared following the general synthetic procedure **E** and was isolated as the TFA salt as a colorless oil (quantitative yield). ^1^H NMR (500 MHz, methanol-*d*_4_) δ 7.46–7.41 (m, 2H), 7.05–6.99 (m, 2H), 4.32 (s, 2H), 3.82 (s, 3H), 3.71–3.56 (m, 3H), 3.11 (td, *J =* 13.1, 2.7 Hz, 2H), 2.73 (s, 3H), 2.39 (d, *J =* 13.5 Hz, 2H), 2.09 (qd, *J =* 13.2, 4.2 Hz, 2H); ESI-HRMS (*m*/*z*): [M + H]^+^ calcd for C_14_H_23_N_2_O^+^, 235.1805; found, 235.1809.

Compound **2o** was prepared starting from intermediates **44** and **59** following the general synthetic procedure **B** and was isolated as a colorless solid (80% yield). ^1^H NMR (400 MHz, methanol-*d*_4_) δ 7.76 (d, *J =* 9.1 Hz, 1H), 7.35–7.31 (m, 4H), 7.25 (ddd, *J =* 8.8, 5.3, 2.8 Hz, 1H), 7.21 (d, *J =* 8.6 Hz, 2H), 6.95 (d, *J =* 2.5 Hz, 1H), 6.85 (d, *J =* 8.6 Hz, 2H), 6.75 (dd, *J =* 9.1, 2.5 Hz, 1H), 5.89 (s, 1H), 4.41 (d, *J =* 13.1 Hz, 2H), 4.08 (q, *J =* 7.0 Hz, 2H), 3.76 (s, 3H), 3.57–3.48 (m, 6H), 2.92 (d, *J =* 12.1 Hz, 2H), 2.82 (d, *J =* 11.6 Hz, 2H), 2.65 (tt, *J =* 11.5, 3.5 Hz, 1H), 2.29–2.19 (m, 2H), 2.18 (s, 3H), 2.05 (d, *J =* 11.7 Hz, 2H), 1.93 (d, *J =* 11.2 Hz, 2H), 1.64 (dtd, *J =* 24.2, 13.3, 12.2, 3.7 Hz, 5H), 1.40 (t, *J =* 7.0 Hz, 3H); ^13^C NMR (101 MHz, methanol-*d*_4_) δ 161.7, 161.0, 160.4, 152.2, 150.7, 138.5, 131.7, 131.7, 130.8, 130.7, 129.3, 129.3, 128.4, 123.0, 114.7, 113.6, 111.2, 107.1, 85.2, 64.5, 64.0, 62.0, 58.3, 55.7, 53.4, 50.6, 46.9, 37.9, 32.2, 28.9, 15.1; ESI-HRMS (*m*/*z*): [M + H]^+^ calcd for C_37_H_48_N_5_O_2_^+^, 594.3803; found, 594.3785.

#### *N*-(1-Benzylpiperidine-4-yl)-7-ethoxy-2-(4-((4-nitro-benzyl)­amino)­piperidine-1-yl)­quinoline-4-amine (**2p**)

Intermediate **44** was prepared following the synthetic procedure for compound **2b**.

Potassium carbonate (830 mg, 6.00 mmol, 2.4 equiv) and potassium iodide (41.0 mg, 0.250 mmol, 0.1 equiv) were added to a stirring solution of *tert*-butyl 4-aminopiperidine-1-carboxylate (500 mg, 2.50 mmol, 1.0 equiv) and 1-(bromomethyl)-4-nitrobenzene (3.00 mmol, 1.2 equiv) in acetonitrile (10 mL), and the reaction mixture was refluxed overnight. After completion of the reaction, the solvent was evaporated, water was added, and the product was extracted using DCM. After washing the organic layer three times with water, it was dried over sodium sulfate, and filtered. After evaporation of the solvent under reduced pressure, the residue was purified by flash column chromatography (DCM:MeOH, 0 to 2%) to afford the intermediate **60** as a pale yellow solid (65% yield). ^1^H NMR (500 MHz, chloroform-d) δ 8.17 (d, *J =* 8.7 Hz, 2H), 7.51 (d, *J =* 8.7 Hz, 2H), 4.01 (s, 2H), 3.93 (s, 2H), 2.80 (t, *J =* 11.7 Hz, 2H), 2.65 (tt, *J =* 10.1, 3.9 Hz, 1H), 1.86 (d, *J =* 11.6 Hz, 2H), 1.45 (s, 9H), 1.29 (td, *J =* 14.7, 4.7 Hz, 2H); ESI-HRMS (*m*/*z*): [M + H]^+^ calcd for C_17_H_26_N_3_O_4_^+^, 336.1918; found, 336.1919. The analytical data are in accordance with previously reported literature.^46^

Intermediate **62** was prepared following the general synthetic procedure **E** and was isolated as the TFA salt as a pale yellow solid (quantitative yield). ^1^H NMR (500 MHz, methanol-*d*_4_) δ 8.31 (d, *J =* 8.7 Hz, 2H), 7.76 (d, *J =* 8.7 Hz, 2H), 4.41 (s, 2H), 3.64–3.51 (m, 3H), 3.11 (td, *J =* 13.2, 2.8 Hz, 2H), 2.43 (d, *J =* 13.8 Hz, 2H), 1.95 (qd, *J =* 13.4, 4.1 Hz, 2H); ESI-HRMS (*m*/*z*): [M + H]^+^ calcd for C_12_H_18_N_3_O_2_^+^, 236.1394; found, 236.1398.

Compound **2p** was prepared starting from intermediate **44** and **62** following the general synthetic procedure **B** and was isolated as a pale yellow solid (63% yield). ^1^H NMR (400 MHz, methanol-*d*_4_) δ 8.22–8.14 (m, 2H), 7.74 (d, *J =* 9.1 Hz, 1H), 7.60 (dd, *J =* 8.9, 2.1 Hz, 2H), 7.35–7.30 (m, 4H), 7.29–7.23 (m, 1H), 6.93 (d, *J =* 2.5 Hz, 1H), 6.74 (dd, *J =* 9.1, 2.6 Hz, 1H), 5.89 (s, 1H), 4.33 (d, *J =* 13.2 Hz, 2H), 4.09 (q, *J =* 7.0 Hz, 2H), 3.93 (s, 2H), 3.56 (s, 2H), 3.55–3.47 (m, 1H), 2.91 (t, *J =* 12.7 Hz, 4H), 2.73 (tt, *J =* 10.6, 4.0 Hz, 1H), 2.24 (td, *J =* 11.8, 2.1 Hz, 2H), 2.09–1.96 (m, 4H), 1.72–1.62 (m, 2H), 1.51–1.43 (m, 2H), 1.40 (t, *J =* 7.0 Hz, 3H); ^13^C NMR (101 MHz, methanol-d_4_) δ 161.6, 161.3, 152.1, 151.1, 149.3, 148.5, 138.5, 131.4, 130.8, 130.3, 129.3, 128.4, 124.5, 122.9, 113.4, 111.3, 107.3, 85.3, 64.4, 64.0, 56.0, 53.4, 50.6, 46.2, 33.0, 32.2, 15.1; ESI-HRMS (*m*/*z*): [M + H]^+^ calcd for C_35_H_43_N_6_O_3_^+^, 595.3391; found, 595.3396.

#### *N*-(1-Benzylpiperidine-4-yl)-7-ethoxy-2-(4-((4-methyl-benzyl)­amino)­piperidine-1-yl)­quinoline-4-amine (**2q**)

Intermediate **44** was prepared following the synthetic procedure for compound **2b**.

Potassium carbonate (830 mg, 6.00 mmol, 2.4 equiv) and potassium iodide (41.0 mg, 0.250 mmol, 0.1 equiv) were added to a stirring solution of *tert*-butyl 4-aminopiperidine-1-carboxylate (500 mg, 2.50 mmol, 1.0 equiv) and 1-(bromomethyl)-4-methylbenzene (3.00 mmol, 1.2 equiv) in acetonitrile (10 mL), and the reaction mixture was refluxed overnight. After completion of the reaction, the solvent was evaporated, water was added, and the product was extracted using DCM. After washing the organic layer three times with water, it was dried over sodium sulfate, and filtered. After evaporation of the solvent under reduced pressure, the residue was purified by flash column chromatography (DCM/MeOH, 0 to 2%) to afford the intermediate **61** as a colorless solid (39% yield). ^1^H NMR (400 MHz, chloroform-d) δ 7.20 (d, *J =* 8.0 Hz, 2H), 7.13 (d, *J =* 7.9 Hz, 2H), 4.01 (d, *J =* 10.1 Hz, 2H), 3.78 (s, 2H), 2.80 (t, *J =* 11.5 Hz, 2H), 2.67 (ddd, *J =* 10.2, 6.3, 3.9 Hz, 1H), 2.33 (s, 3H), 1.90–1.80 (m, 2H), 1.53 (dd, *J =* 10.2, 5.3 Hz, 1H), 1.45 (s, 9H), 1.30 (qd, *J =* 11.5, 3.4 Hz, 2H); ESI-HRMS (*m*/*z*): [M + H]^+^ calcd for C_18_H_29_N_2_O_2_^+^, 305.2224; found, 305.2228.

Intermediate **63** was prepared following the general synthetic procedure **E** and was isolated as the TFA salt as a colorless solid (quantitative yield). ^1^H NMR (500 MHz, methanol-*d*_4_) δ 7.38 (d, *J =* 8.1 Hz, 2H), 7.27 (d, *J =* 8.0 Hz, 2H), 4.22 (s, 2H), 3.58–3.46 (m, 3H), 3.09 (td, *J =* 13.3, 2.9 Hz, 2H), 2.40 (d, *J =* 14.0 Hz, 2H), 2.36 (s, 3H), 1.92 (qd, *J =* 13.4, 4.2 Hz, 2H); ESI-HRMS (*m*/*z*): [M + H]^+^ calcd for C_13_H_21_N_2_^+^, 205.1699; found, 205.1698.

Compound **2q** was prepared starting from intermediates **44** and **63** following the general synthetic procedure **B** and was isolated as a colorless solid (64% yield). ^1^H NMR (400 MHz, methanol-*d*_4_) δ 7.74 (d, *J =* 9.1 Hz, 1H), 7.34–7.30 (m, 4H), 7.27 (dd, *J =* 5.7, 3.0 Hz, 1H), 7.22 (d, *J =* 8.0 Hz, 2H), 7.13 (d, *J =* 7.9 Hz, 2H), 6.93 (d, *J =* 2.5 Hz, 1H), 6.74 (dd, *J =* 9.1, 2.5 Hz, 1H), 5.88 (s, 1H), 4.33 (d, *J =* 13.2 Hz, 2H), 4.08 (q, *J =* 7.0 Hz, 2H), 3.75 (s, 2H), 3.55 (s, 2H), 3.54–3.45 (m, 1H), 2.95–2.83 (m, 4H), 2.70 (ddt, *J =* 10.8, 8.3, 4.0 Hz, 1H), 2.30 (s, 3H), 2.27–2.18 (m, 2H), 2.05 (d, *J =* 11.7 Hz, 2H), 2.01–1.95 (m, 2H), 1.73–1.61 (m, 2H), 1.47–1.42 (m, 1H), 1.40 (t, *J =* 7.0 Hz, 4H); ^13^C NMR (101 MHz, methanol-*d*_4_) δ 161.6, 161.3, 152.0, 151.1, 138.6, 137.8, 137.8, 130.8, 130.1, 129.5, 129.3, 128.4, 122.9, 113.4, 111.3, 107.3, 85.3, 64.4, 64.0, 55.7, 53.4, 51.0, 50.5, 46.2, 32.9, 32.2, 21.1, 15.1; ESI-HRMS (*m*/*z*): [M + H]^+^ calcd for C_36_H_46_N_5_O^+^, 564.3697; found, 564.3694.

#### *N*-(1-Benzylpiperidine-4-yl)-7-ethoxy-2-(4-((4-(prop-2-yn-1-yloxy)­benzyl)­amino)­piperidine-1-yl)­quinoline-4-amine (**2r**)

Intermediate **44** was prepared following the synthetic procedure for compound **2b**.

To a solution of the *N*-(*tert*-butoxycarbonyl)-4-piperidone (1.0 g, 5.0 mmol, 1.0 equiv) in methanol (25 mL) was added (4-(prop-2-yn-1-yloxy)­phenyl)­methanamine (5.5 mmol, 1.1 equiv) and acetic acid (0.75 mL). Sodium cyanoborohydride (0.63 g, 10 mmol, 2.0 equiv) was added portionwise to the reaction mixture. After stirring overnight at rt, the solvent was evaporated under vacuum, and to the residue was added NaOH solution (2 M) and DCM. The organic phase was washed with brine, dried over sodium sulfate, and filtered, and the resulting crude mixture was purified by flash column chromatography (DCM:MeOH, 0 to 3%) to give intermediate **64** as a colorless oil (677 mg, 39% yield). ^1^H NMR (400 MHz, chloroform-d) δ 7.25 (d, *J =* 9.0 Hz, 2H), 6.98–6.88 (m, 2H), 4.68 (d, *J =* 2.4 Hz, 2H), 4.01 (d, *J =* 9.9 Hz, 2H), 3.76 (s, 2H), 2.80 (t, *J =* 11.5 Hz, 2H), 2.66 (ddd, *J =* 10.1, 6.3, 3.9 Hz, 1H), 2.50 (t, *J =* 2.4 Hz, 1H), 1.89–1.80 (m, 2H), 1.45 (s, 9H), 1.34–1.24 (m, 2H); ESI-HRMS (*m*/*z*): [M + H]^+^ calcd for C_20_H_29_N_2_O_3_^+^, 345.2173; found, 345.2163.

Intermediate **65** was prepared following the general synthetic procedure **E** and was isolated as the TFA salt of intermediate **65** as a colorless solid (quantitative yield). ^1^H NMR (500 MHz, methanol-d_4_) δ 7.47–7.42 (m, 2H), 7.10–7.04 (m, 2H), 4.76 (d, *J =* 2.4 Hz, 2H), 4.21 (s, 2H), 3.57–3.52 (m, 2H), 3.48 (td, *J =* 7.8, 3.9 Hz, 1H), 3.10 (td, *J =* 13.2, 2.8 Hz, 2H), 2.95 (t, *J =* 2.4 Hz, 1H), 2.40 (d, *J =* 13.9 Hz, 2H), 1.90 (qd, *J =* 13.4, 4.2 Hz, 2H); ESI-HRMS (*m*/*z*): [M + H]^+^ calcd for C_15_H_21_N_2_O^+^, 245.1648; found, 245.1649.

Compound **2r** was prepared starting from intermediates **44** and **65**, following the general synthetic procedure **B**, and was isolated as a colorless solid (69% yield). ^1^H NMR (500 MHz, methanol-*d*_4_) δ 8.13 (d, *J =* 9.2 Hz, 1H), 7.56 (dd, *J =* 7.1, 2.1 Hz, 2H), 7.52–7.45 (m, 5H), 7.31 (d, *J =* 2.4 Hz, 1H), 7.07 (d, *J =* 8.7 Hz, 2H), 7.02 (dd, *J =* 9.2, 2.5 Hz, 1H), 6.08 (s, 1H), 4.76 (d, *J =* 2.3 Hz, 2H), 4.49 (d, *J =* 13.8 Hz, 2H), 4.24 (s, 5H), 4.17 (q, *J =* 7.0 Hz, 2H), 3.57 (ddd, *J =* 15.8, 11.5, 4.2 Hz, 1H), 3.45 (d, *J =* 11.9 Hz, 2H), 3.34 (s, 2H), 3.21 (s, 2H), 2.96 (t, *J =* 2.4 Hz, 1H), 2.38 (d, *J =* 10.2 Hz, 2H), 2.25 (d, *J =* 12.5 Hz, 2H), 2.07 (q, *J =* 10.8 Hz, 2H), 1.85 (qd, *J =* 12.6, 3.9 Hz, 2H), 1.45 (t, *J =* 7.0 Hz, 3H); ^13^C NMR (126 MHz, methanol-*d*_4_) δ 163.9, 160.0, 155.1, 154.7, 141.1, 132.5, 132.2, 130.7, 130.2, 125.5, 125.0, 116.7, 115.9, 109.2, 101.6, 83.5, 79.4, 77.1, 65.4, 56.7, 55.7, 52.4, 49.9, 49.2, 46.7, 29.5, 14.9; ESI-HRMS (*m*/*z*): [M + H]^+^ calcd for C_38_H_46_N_5_O_2_^+^, 604.3646; found, 604.3661.

#### *N*-(1-Benzylpiperidine-4-yl)-7-ethoxy-2-(4-((4-((1-(2-methoxyethyl)-1*H*-1,2,3-triazol-4-yl)­methoxy)­benzyl)-amino)-piperidine-1-yl)­quinoline-4-amine (**2s**)

Compound **2r**, obtained as described above, was used as the starting material for the next transformation. A reaction tube was charged with compound **2r** (36.2 mg, 60.0 μmol, 1.0 equiv) and 1-azido-2-methoxyethane (4 mL of a solution of azide in DMF-DCM (2 mg/mL); 7.90 mg, 78.0 μmol, 1.3 equiv) and *t*-BuOH (2 mL) at rt. Then, a solution of CuSO_4_ pentahydrate (3.00 mg, 20 mol %) and sodium ascorbate (4.80 mg, 40 mol %) in water (2 mL) were added to the reaction mixture. The reaction mixture was stirred at 40 °C for 5 h. After completion, the reaction mixture was extracted with DCM. The organic phase was washed with brine, dried over sodium sulfate, and filtered to give a crude product, which was purified by flash column chromatography (DCM/MeOH, 0 to 3%) to obtain compound **2s** as a colorless solid (35.6 mg, 84% yield). ^1^H NMR (500 MHz, methanol-*d*_4_) δ 8.00 (s, 1H), 7.75 (d, *J =* 9.1 Hz, 1H), 7.33–7.25 (m, 7H), 6.99–6.96 (m, 2H), 6.94 (d, *J =* 2.6 Hz, 1H), 6.74 (dd, *J =* 9.1, 2.6 Hz, 1H), 5.89 (s, 1H), 5.13 (s, 2H), 4.56–4.54 (m, 2H), 4.35 (d, *J =* 13.0 Hz, 2H), 4.08 (q, *J =* 7.0 Hz, 2H), 3.77–3.74 (m, 2H), 3.73 (s, 2H), 3.55 (s, 2H), 3.54–3.48 (m, 1H), 3.31 (s, 3H), 2.94–2.84 (m, 4H), 2.70 (tt, *J =* 10.9, 4.0 Hz, 1H), 2.22 (td, *J =* 11.9, 2.5 Hz, 2H), 2.08–2.03 (m, 2H), 2.01–1.96 (m, 2H), 1.71–1.63 (m, 2H), 1.47–1.41 (m, 2H), 1.41 (t, *J =* 7.1 Hz, 3H); ^13^C NMR (126 MHz, methanol-*d*_4_) δ 161.5, 161.4, 158.9, 152.0, 151.2, 144.9, 138.5, 133.6, 130.8, 130.8, 129.3, 128.4, 125.9, 122.9, 115.9, 113.4, 111.3, 107.3, 85.3, 71.7, 64.4, 64.0, 62.4, 59.0, 55.7, 53.4, 51.3, 50.6, 46.2, 32.8, 32.2, 15.1; ESI-HRMS (*m*/*z*): [M + H]^+^ calcd for C_41_H_53_N_8_O_3_^+^, 705.4235; found, 705.4231.

#### *N*-(2-(2-(2-(2-(4-((4-(((1-(4-((1-Benzylpiperidine-4-yl)­amino)-7-ethoxyquinoline-2-yl)­piperidine-4-yl)­amino)-methyl)­phenoxy)­methyl)-1*H*-1,2,3-triazol-1-yl)­ethoxy)-ethoxy)­ethoxy)­ethyl)-3′,6′-bis­(dimethylamino)-3-oxo-3*H*-spiro­[isobenzofuran-1,9′-xanthene]-6-carboxamide (**2s-TAMRA**)

Compound **2r**, obtained as described above, was used as the starting material for the next transformation. A reaction tube was charged with compound **2r** (17.1 mg, 12.9 μmol, 1.2 equiv), *N*-(2-(2-(2-(2-azidoethoxy)­ethoxy)­ethoxy)­ethyl)-3′,6′-bis­(dimethylamino)-3-oxo-3H-spiro­[isobenzofuran-1,9′-xanthene]-6-carboxamide (N_3_–PEG_3_-TAMRA, 10.0 mg, 15.9 μmol, 1.0 equiv), and tris­[(1-benzyl-1*H*-1,2,3-triazol-4-yl)­methyl]­amine (TBTA, 0.91 mg, 1.71 μmol, 10 mol %) in DMF (0.17 mL). First, a mixture of H_2_O/*t*-BuOH (1:1, 0.30 mL), then aqueous solutions of CuSO_4_ (1 M, 16.4 mL) and sodium ascorbate (0.1 M, 32.8 mL) were added to the reaction mixture. The reaction mixture was stirred at rt for 12 h. After completion, the reaction mixture was extracted with DCM. The organic phase was washed with brine, dried over sodium sulfate, and filtered to give a crude product, which was purified by preparative reversed-phase HPLC (H_2_O (0.05% TFA)/MeCN = 95:5 to 5:95) to obtain compound **2s-TAMRA** as a red solid (6.50 mg, 40% yield). ^1^H NMR (500 MHz, methanol-*d*_4_) δ 8.76 (d, *J* = 1.8 Hz, 1H), 8.24 (dd, *J* = 7.9, 1.9 Hz, 1H), 8.15 (s, 1H), 8.11 (d, *J* = 9.3 Hz, 1H), 7.55–7.49 (m, 6H), 7.48–7.45 (m, 2H), 7.20 (d, *J* = 2.5 Hz, 1H), 7.15–7.09 (m, 4H), 7.07–7.02 (m, 3H), 6.97 (d, *J* = 2.4 Hz, 2H), 6.02 (s, 1H), 5.16 (s, 2H), 4.59–4.55 (m, 2H), 4.42 (d, *J* = 14.1 Hz, 2H), 4.37 (s, 2H), 4.25 (s, 2H), 4.16 (q, *J* = 7.0 Hz, 2H), 3.90–3.87 (m, 2H), 3.71–3.67 (m, 2H), 3.67–3.61 (m, 12H), 3.61–3.53 (m, 2H), 3.30 (s, 12H), 3.29–3.26 (m, 2H), 2.40–2.28 (m, 5H), 2.14–1.93 (m, 3H), 1.88–1.79 (m, 2H), 1.45 (t, *J* = 7.0 Hz, 3H); ^13^C NMR (126 MHz, methanol-d_4_) δ 168.2, 167.5, 164.1, 160.8, 160.7, 159.0, 159.0, 155.3, 154.7, 144.4, 141.0, 138.1, 137.6, 133.1, 132.6, 132.5, 132.3, 132.0, 131.9, 131.4, 131.3, 130.4, 126.3, 125.0, 125.0, 116.6, 116.1, 115.6, 114.7, 109.2, 101.4, 97.5, 83.4, 71.6, 71.5, 71.4, 71.4, 70.5, 70.3, 65.4, 62.5, 61.7, 55.6, 51.5, 49.9, 49.6, 49.5, 49.5, 49.3, 49.3, 49.2, 49.1, 49.0, 48.8, 48.7, 48.5, 46.5, 41.2, 40.9, 29.6, 29.3, 14.8.

### Cloning of Expression Plasmids, Expression of Recombinant Proteins, and Protein Purification

Plasmids for expression of the human CHD1 tCD in fusion with a hexahistidine tag (His), enhanced green fluorescent protein (GFP), and/or NanoLuc luciferase (NLuc) [pET15b_hCHD1_260–443_His, pET15b_His-hCHD1_270–443_, pET15b_His-GFP-hCHD1_260–443_, and pNLF1_NanoLuc-hCHD1_260–443_] were generated by PCR cloning using standard techniques or have been previously described.^5^ The plasmid pET15b_CHD1_260–443_(D425A)­His encoding mutant CHD1 was generated by standard PCR cloning. The cDNA of human METTL21A_1–226_ was cloned into pET28a in fusion with an N-terminal His-SUMO tag. The cDNA of human HSPA8_1–641_ was cloned into pGEX-6P-1. The cDNA of human METTL21B_1–226_ was cloned into a pFastBac-HTb. Cloning vectors pET15b and pET18a were obtained from Novagen, pNLF1-N [CMV/Hygro], pGEX-6P-1, and pFastBac-HTb were obtained from Promega and Sigma-Aldrich. Detailed information on cloning procedures will be provided upon request. Proteins were expressed in *Escherichia coli* BL21-CodonPlus­(DE3)-RIPL. Cultures were grown in Terrific Broth medium (Sigma-Aldrich) and induced with 0.5 mM IPTG overnight at 18 °C. Bacterial pellets for expression of CHD1 proteins were resuspended in buffer 1 [20 mM Tris-HCl (pH 8.0), 200 mM NaCl] and cells were disrupted in an EmulsiFlex high-pressure homogenizer (Avestin). Proteins were affinity-purified in batch using TALON Superflow affinity resin (GE Healthcare). The resin was washed with buffer 1, and proteins were eluted with buffer 2 [20 mM Tris-HCl (pH 8.0), 50 mM NaCl, 50 mM imidazole (pH 8.0)]. Proteins were further purified by ion exchange chromatography (MonoQ HR 5/50 or Capto HiResQ 5/50 column, GE Healthcare) in 20 mM Tris-HCl (pH 8.0), 50–500 mM NaCl buffer followed by gel filtration (HiLoad 16/600 Superdex 75 pg column, GE Healthcare) in buffer 3 [50 mM HEPES (pH 7.5) 200 mM NaCl] using an ÄKTA pure HPLC system (GE Healthcare).

His-SUMO-METTL21A was first purified using a HisTrap column (GE Healthcare), then the His-SUMO tag was removed by Ulp1 cleavage. GST-HSPA8 was purified with glutathione sepharose 4B (GE Healthcare), then the GST tag was removed by Prescission protease cleavage. The proteins were further purified by gel filtration using a HiLoad 16/600 Superdex 75 pg column (GE Healthcare) in buffer containing 10 mM Tris/HCl (pH 8.0), 100 mM NaCl, and 1 mM dithiothreitol. Purified proteins were aliquoted, flash frozen in liquid nitrogen, and stored at −80 °C. His-METTL21B_1–226_ was expressed in Sf9 cells, affinity-purified using a HisTrap column, and further purified by gel filtration on a HiLoad 16/600 Superdex 75 pg column (GE Healthcare) in buffer containing 10 mM Tris/HCl (pH 8.0), 100 mM NaCl, and 1 mM dithiothreitol.

Expression plasmids, protein expression and purification procedures have been described for hSPIN1_49–262_,^15^ hKMT9 (KMT9α_1–214_-His/KMT9β_2–125_),^47^ glutathione-S-transferase (GST)-tagged mTaf3_857–924_ (GST-mTaf3-PHD),^48^ GST-hPHF8_37–102_,^48^ and GST-LSD1_2–852_.^49^

### Isothermal Titration Calorimetry (ITC)

ITC experiments were performed with a Microcal VP-ITC instrument (Malvern). Ligand (200 μM to 1.2 mM in the syringe) was titrated to purified hCHD1_260–443_His protein (20 μM in the sample cell) in ITC buffer [25 mM HEPES (pH 7.5) 100 mM NaCl, and 0.2–0.6% DMSO depending on ligand concentration] at 20 °C. Instrument settings were: 3 μL initial injection followed by 28 injections of 10 μL at an injection rate of 0.5 μL/s, 240 s spacing between injections, and a stirring speed of 307 rpm. Data were analyzed with manual baseline corrections using the Origin 7 software for VP-ITC instruments (Malvern). (Number of experiments: *n* = 1 for **1b**, **1c**, **1f**, **1g**, **1h**, **1j**, **1k**, **1m**, **1n**, **1o**, **1p**, **1r**, **1t**, **2a**, **2b**, **2c**, **2d**, **2e**, **2g**, **2i**, **2j**, **2k**, **2m**, **2o**, **2p**, **2q**, **2r**, and **2s-TAMRA**; *n* = 2 for **1a**, **1i**, **1l**, **1q**, **1s**, **2f**, **2h**, **2l**, **2n**, and **2s**.)

### Förster Resonance Energy Transfer (FRET) Assay

Compounds were initially diluted from 100 mM or 200 mM DMSO stocks in assay buffer [25 mM HEPES (pH 7.5), 50 mM NaCl, 1 mg/mL BSA, 0.5 mg/mL Tween-20, 2 mM DTT] followed by serial dilutions in the same buffer containing 2% DMSO. A protein-peptide mixture was prepared in assay buffer containing 0.2 μM purified His-GFP–CHD1_260–443_ protein and 2 μM TAMRA-H3_1–14_K4me3 peptide (Table S3). For the 100% inhibition control, unlabeled H3_1–12_K4me3 peptide (Table S3) was diluted to 400 μM in assay buffer containing 2% DMSO. In a 384-well black OptiPlate (PerkinElmer), 10 μL of compound dilution were added to 10 μL of protein-peptide mixture (total assay volume of 20 μL and 1% DMSO per well). For the 0% inhibition control, assay buffer with 2% DMSO was used instead of the compound; for the 100% inhibition control, the H3_1–12_K4me3 solution was added. For the blank control, assay buffer with 2% DMSO was mixed with DMSO-free assay buffer in equal volumes. All conditions were tested in at least duplicate. Plates were sealed with Black TopSeal-A film (PerkinElmer), centrifuged for 1 min at 700 rpm, incubated at 25 °C for 30 min at 500 rpm, then centrifuged again for 1 min at 700 rpm. Fluorescence was measured using an EnVision 2102 multilabel plate reader (PerkinElmer) with excitation at 450 nm and emission at 535 and 590 nm. After blank subtraction, the FRET ratio (FR) was calculated for each well as

$$\mathrm{FR}=\frac{I_{590}}{I_{535}}$$

where *I*_590_ and *I*_535_ are the blank-corrected fluorescence intensities. Inhibition (%) was calculated using

$$\mathrm{Inh}\;\left(\%\right)=\frac{{\mathrm{FR}}_{i}-{\mathrm{FR}}_{0\%}}{{\mathrm{FR}}_{100\%}-{\mathrm{FR}}_{0\%}}\cdot 100\%$$

where FR_i_, FR_0%_, and FR_100%_ represent the FRET ratios for the sample, 0% inhibition control, and 100% inhibition control, respectively. Inhibition values were plotted against compound concentration in GraphPad Prism 9 or OriginPro2019. Dose–response curves were fitted using the equation

$$Y=\mathrm{bottom}+\left(\frac{\mathrm{top}-\mathrm{bottom}}{{\left(1+\frac{{\mathrm{IC}}_{50}}{X}\right)}^{\mathrm{HillSlope}}}\right)$$

### Fluorescent Thermal Shift Assay (FTSA)

Compounds were diluted from 10 mM DMSO stock solutions into assay buffer (25 mM HEPES, pH 7.5, 50 mM NaCl, 2 mM DTT) to the desired concentrations, keeping a final DMSO concentration of 1.6% (v/v). Then, 10 μL of a protein-dye mix containing 0.2 mg/mL CHD1_260–443_His and 10× SYPRO Orange was added to 10 μL of each compound solution in a 96-well Hard-Shell PCR plate (Bio-Rad). Control wells received buffer with 1.6% DMSO. All measurements were performed in duplicate. Plates were sealed with an adhesive PCR seal (Biozym), centrifuged (700 rpm, 1 min), and incubated at 25 °C for 15 min (shaking at 400 rpm) followed by a second centrifugation step. Fluorescence (excitation: 485 nm; emission: 530 nm) was recorded during sample heating in a temperature gradient from 20 to 100 °C at 1 °C/min using a CFX96 Touch Real-Time PCR Detection System (Bio-Rad). Melting temperatures (*T*_m_) were determined using Bio-Rad CFX Manager (v3.1), Microsoft Excel, and GraphPad Prism 7, applying the DSF analysis tool described by Niesen et al.^50^

### Protein Crystallization and Crystal Soaking

Crystallization was performed using the vapor diffusion sitting-drop method with an Oryx Nano pipetting robot (Douglas Instruments, U.K.) in MRC 2 Well UVP Plates (SWISSCI, U.K.) at 4 °C. CHD1_270–443_/LSD1K114me3 crystals were obtained as previously described.^5^ Briefly, purified His-CHD1_270–443_ protein (11–15 mg/mL) was incubated with 2 mM LSD1K114me3 peptide (Table S3) on ice for 1 h, followed by centrifugation at 4 °C for 10 min to remove precipitates. Crystals formed within 1–2 days from 0.30 μL protein solution and 0.30 μL reservoir solution containing 6–16% (w/v) PEG 3,350, 0.2 M l-proline, and 0.1 M HEPES at pH 6.5–8.0. CHD1_270–443_/inhibitor complexes were obtained by soaking preformed CHD1_270–443_/LSD1K114me3 crystals in a mixture of reservoir solution and 10% (v/v) DMSO containing inhibitor (final inhibitor concentration: **2b** and **2l** = 20 mM; **1q**, **2n** and **2s** = 10 mM) for 24 h. Crystals were cryoprotected with reservoir solution supplemented with 15% (v/v) 2R,3R-(−)-butanediol, mounted on nylon loops, and flash-cooled in liquid nitrogen.

### Data Collection and Structure Determination

X-ray diffraction data for CHD1_270–443_/**2b** (PDB code 9T9E), CHD1_270–443_/**2l** (PDB code 9T9F), and CHD1_270–443_/**2n** (PDB code 9T9H) were collected on beamline BM07 at the European Synchrotron Radiation Facility (ESRF, Grenoble, France) using a Pilatus 6 M detector. X-ray diffraction data for CHD1_270–443_/**2s** (PDB code 9T9I) and CHD1_270–443_/**1q** (PDB code 9T9G) were collected on beamline ID30A-3 at the ESRF using an Eiger X 4 M detector. The data sets were processed with autoPROC^51^ and scaled using Aimless.^52^ The structures were solved by molecular replacement with Phaser^53^ using the CHD1_270–443_/LSD1K114me2 complex (PDB 5AFW)^5^ as the search model. Model building and refinement were performed iteratively with COOT^54^ and either REFMAC^55^ or Phenix.refine.^56^ Ligand restraints were generated using the grade Web Server (Global Phasing Ltd., U.K.). The electron density for all ligands was well resolved. Final structures were validated using MolProbity.^57^ Data collection and refinement statistics are summarized in Table S1.

### Methyltransferase Inhibition Assays

Inhibition of KMT9 methyltransferase activity was performed as previously described.^47^ Briefly, the assay was carried out in duplicates in assay buffer [50 mM BTP (pH 8.5) 1 mM MgCl_2_, 1 mM DTT, and 0.01% Triton-X100] in the presence of varying inhibitor concentrations, 25 nM purified KMT9, 0.3 μM 3H-SAM, 0.7 μM SAM, and 5 μM His-ETF1_140–275_ in a final volume of 20 μL. Reactions were incubated at 30 °C for 2 h with shaking and then stopped by adding 5 μL of a 50% trichloroacetic acid (TCA) solution. Then, 22 μL reaction mixtures were transferred into 96-well MultiScreenHTS FB filter plates (Merck) and subsequently washed with 10% TCA and 100% ethanol. After drying overnight, filters were transferred into Pony Vials (PerkinElmer Inc.) and incubated in 3 mL of Ultima Gold scintillation cocktail (PerkinElmer Inc.) for 30 min. The scintillation signal was measured 3 times for 1 min using a TriCarb 2910 TR (PerkinElmer Inc.) scintillation counter set to 3H CPM mode (LL: 0, UL: 18.6).

Inhibition of METTL21A methyltransferase activity was determined in a white 384-well OptiPlate instrument (PerkinElmer Inc.) in duplicates. For IC_50_ determination, 10-point 3-fold dilution series of inhibitor was preincubated with 1 μM HSPA8, 40 μM SAM, 1x MTaseGlo Reagent in assay buffer [20 mM Tris (pH 8.0) 50 mM NaCl, 1 mM EDTA, 3 mM MgCl_2_, 0.1 mg/mL BSA] for 15 min. Then the reaction was started by the addition of 0.4 μM METTL21A and incubated for 4 h at 30 °C. After incubation, 2x MTaseGlo Detection solution was added, and the mixture was incubated for 1 h at 25 °C. Luminescence was measured using an EnVision 2102 multilabel plate reader (PerkinElmer). Inhibition was calculated using the following formula

$$\mathrm{inhibition}\left[\%\right]=\left(1-\frac{x_{c}-x_{\mathrm{pos}}}{x_{\mathrm{pos}}-x_{\mathrm{neg}}}\right)\times 100$$

with *x*_c_: signal of compound, *x*_pos_: mean signal of positive control, *x*_neg_: mean signal of negative control. Data fitting was carried out in GraphPad 7.0 by using nonlinear fit ([inhibitor] vs the response–variable slope (four parameters)).

### Fluorescent Thermal Shift Assay (FTSA)

FTSA assays were carried out in 96-well hard-shell PCR plates (Bio-Rad Laboratories Inc.) in duplicates. Desired concentrations of inhibitors in DMSO were mixed with 2 μM METTL21B and 5x SyproOrange in assay buffer [25 mM HEPES (pH 7.5), 100 mM NaCl, 1 μM DTT] and incubated at 25 °C for 15 min. Controls contained DMSO instead of the inhibitor. Measurements were conducted using a CFX96 Touch Real-Time PCR Detection System (Bio-Rad Laboratories Inc.). The plate was equilibrated at 20 °C for 4 min and then heated stepwise at a rate of 1 °C per 15 s until 95 °C was reached. After every step, fluorescence was measured (λ_ex_ = 485 nm, λ_em_ = 530 nm) in “FRET mode.” Calculation of melting points was conducted using a Boltzmann sigmoidal model in GraphPad Prism 7.0.

### Fluorescence Polarization Assay

Ten μL of protein solution containing either 0.3 μM GST–mTaf3–PHD and 0.06 μM Cy5–H3K4me3 (), 0.2 μM His-hSPIN1_49–262_ and 0.2 μM full-length (FL)-H3K4me3 peptide^15^ or 1 μM GST–hPHF8_37–102_ ^48^ and 0.02 μM FL–H3K4me3 () were mixed with 10 μL of compound dilution in a 384-well black nonbinding plate (Greiner), resulting in a total assay volume of 20 μL per well. CHD1 tCD inhibitors were serially diluted from 10 mM DMSO stocks in TAF3 assay buffer [50 mM Bis-Tris (pH 6.5) 200 mM NaCl, 10 μM ZnCl_2_, 1 mg/mL BSA, 0.5 mg/mL Tween-20, 2 mM DTT], SPIN1 assay buffer [25 mM HEPES (pH 7.5) 100 mM NaCl, 1 mg/mL BSA, 0.5 mg/mL CHAPS], or PHF8 assay buffer [25 mM HEPES (pH 7.5) 100 mM NaCl, 10 μM ZnCl_2_, 1 mg/mL BSA, 0.5 mg/mL CHAPS], respectively, containing 10% DMSO at each dilution step. The final DMSO concentration in all wells was 5%. For the 0% inhibition control, assay buffer with 10% DMSO was used instead of the compound. For the 100% inhibition control, assay buffer with 10% DMSO was mixed with 0.06 μM Cy5–H3K4me3 peptide in the absence of TAF3, 0.02 μM FL-H3K4me3 in the absence of SPIN1, or 0.02 μM FL–H3K4me3 in the absence of PHF8. For the blank control, assay buffer with 10% DMSO was combined with DMSO-free assay buffer. All conditions were tested at least in duplicate. Plates were sealed with Black TopSeal-A film (PerkinElmer), centrifuged for 1 min at 700 rpm, incubated at 25 °C for 30 min with shaking at 500 rpm, then centrifuged again for 1 min at 700 rpm. Fluorescence polarization was measured on an EnVision 2102 multilabel plate reader (PerkinElmer) with excitation at 480 nm and emission at 535 nm, detecting fluorescence parallel (S-plane) and perpendicular (P-plane) to the excitation plane. After blank subtraction, polarization (*P*, in mP) was calculated as

$$P(\mathrm{mP})=1000\cdot \frac{I_{S}-G\cdot I_{P}}{I_{S}+G\cdot I_{P}}$$

where *I*_S_ and *I*_P_ are the fluorescence intensities of the S- and P-plane, and *G* is a device-specific factor (0.93). Inhibition (%) was calculated using the equation

$$I\left(\%\right)=100\%\cdot \left(1-\frac{P_{I}-P_{100\%}}{P_{0\%}-P_{100\%}}\right)$$

where *P*_I_, *P*_0%_, and *P*_100%_ are the polarization values for the sample, 0% inhibition control, and 100% inhibition control, respectively. Inhibition values were plotted against compound concentration in GraphPad Prism 9 or OriginPro2019. Dose–response curves were fitted using the equation

$$Y=\mathrm{bottom}+\left(\frac{\mathrm{top}-\mathrm{bottom}}{{\left(1+\frac{{\mathrm{IC}}_{50}}{X}\right)}^{\mathrm{HillSlope}}}\right)$$

### Peroxidase Assay

A standard peroxidase-coupled assay was used to determine IC_50_ values for LSD1 inhibition as described previously.^58^ Briefly, LSD1 enzyme at a final concentration of 0.035 μM in buffer (45 mM HEPES, 40 mM NaCl, pH 8.5) was incubated with the inhibitor for 20 min in an OptiPlate-384 microtiter plate (*PerkinElmer*). H3_1–20_K4me2 peptide (*Peptide Specialty Laboratories GmbH*) at a final concentration of 20 μM was used as substrate. After 1 h of incubation, Amplex Red reagent/horseradish peroxidase (HRP) mixture was added (final concentration: 50 μM AmplexRed and 1 U/mL HRP; *Sigma-Aldrich*, *P8125*) in the reaction buffer. Immediately after addition, fluorescence intensity corresponding to the resorufin product was measured at λ_ex_ = 510 nm and λ_em_ = 615 nm using a BMG FLUOstar Omega microplate reader. Percent inhibition was calculated relative to the compound-free DMSO control (positive control) and the no-substrate negative control. Inhibition curves were analyzed by sigmoidal curve fitting using GraphPad Prism 9.0.2. IC_50_ values are reported as the mean of two independent experiments.

### Cell Culture and Proliferation Assay

Cell lines used in this study were obtained from the American Type Culture Collection (ATCC), the European Collection of Cell Cultures (ECACC), or Caliper Life Sciences and cultured as recommended by the suppliers. Media were supplemented with 10% fetal calf serum and penicillin/streptomycin. Cell proliferation was determined using the X-Celligence RTCA system (Roche). For real-time recording of cell proliferation, PC-3M-Luc (3000 cells/well), LNCaPLuc (20,000 cells/well), or 22Rv1 (20,000 cells/well) cells were seeded into 16-well E-plates (Roche). Cell indices were automatically recorded every 15 min. Twenty-four h prior to seeding, PC-3M-Luc and LNCaP-Luc cells were transfected with siRNA at a final concentration of 80 nM using DharmaFECT 2 Transfection Reagent (Thermo Fisher Scientific), and 22Rv1 cells were transfected with Lipofectamine RNAiMax (Thermo Fisher Scientific). The following stealth siRNAs (Invitrogen) were used: 5′-GAAAGTCCTAGATCCACACGCAAAT-3′ (siCtrl), 5′-AAUGAGAGCUCCAUCUUCCCAGCUG-3′ (siCHD1–1), 5′-GCUACCUCAUUAAACCACCAGAUAA-3′ (siCHD1–2). Knockdown efficiencies were verified by Western blot. Proliferation curves are presented as mean ± standard deviation (*n* = 4 for each condition). Statistical analyses were performed using the Student′s *t* test with two-tailed distribution. Statistical significance is presented as * *p* < 0.05, ** *p* < 0.01, *** *p* < 0.001.

### Transient Transfection of HEK293T Cells and Bioluminescence Resonance Energy Transfer (BRET) Measurements

NanoBRET experiments were performed with HEK293T cells plated in 6-well plates (Sarstedt, cat. #83.1839.300) at a density of 8 × 10^5^ cells per well and incubated for 2–4 h at 37 °C and 5% CO_2_ before transfection. Plasmid encoding NLuc-CHD1_260–443_ fusion protein was transfected using Fugene HD Transfection reagent (Promega) according to the manufacturer’s protocol. Briefly, 2 μg expression plasmid were dissolved in 100 μL medium without serum and phenol red to obtain a concentration of 0.02 μg DNA per μL. Fugene reagent was added, the sample vortexed for a short time, and incubated for 15 min at rt. The mix was added dropwise to the HEK-293T cells, followed by incubation for 24 h at 37 °C and 5% CO_2_. Cells were trypsinized, resuspended in medium without serum and phenol red, and adjusted to a concentration of 2 × 10^5^ cells per mL. To determine affinities of the inhibitors, a final tracer concentration of 8 μM was used. Serially diluted inhibitor and tracer were added to the cell suspension, and 100 μL were seeded in 96-well white, sterile nonbinding surface plates. Plates were incubated at 37 °C and 5% CO_2_ for 2 h. For BRET measurements, NanoBRET NanoGlo Substrate (Promega cat. #N1571) was added to the wells according to the manufacturer’s protocol. For all measurements, the 2102 EnVisionTM Multilabel reader (PerkinElmer) was used, equipped with 460 nm (donor) and 590LP nm (acceptor) filter. Data analysis was performed with Prism (GraphPad Software, San Diego, CA, USA). Milli-BRET units (mBU) are BRET values multiplied with 1000. Tracer affinities were calculated using the following equation

$$Y=\frac{B_{\max}\times X}{K_{d}+X}$$

with *B*_max_ representing the maximal response upon saturation, *X* the tracer concentration, and *K*_d_ the equilibrium dissociation constant. Apparent *K_i_* values were calculated using the Cheng–Prusoff equation.

$$K_{i}=\frac{{\mathrm{IC}}_{50}}{1+\frac{\left[\mathrm{Tracer}\right]}{K_{d,\mathrm{app}}}}$$

with *K*_d,app_ as the apparent *K*_d_ value of the fluorescent ligand (tracer).

### Cell Lysate Pull-Down Assay and Western Blotting

Pull-down assays were essentially performed as described by Johnson et al.^22^ with minor modifications. Briefly, HEK293T cells were lysed in Cell Lysis Buffer (Cell Signaling) supplemented with cOmplete, EDTA-free protease inhibitor cocktail (Roche). The protein concentration was calculated by Bradford assay with a BSA standard curve. Streptavidin sepharose beads (Merck) were washed (2 × 500 μL) with assay buffer [20 mM Tris/HCl (pH8.0), 200 mM NaCl, 0.1% Tween 20] and then incubated with 30 μg of a biotinylated LSD1-K114me3 peptide [104-TPEGRRTSRR­(Kme3)­RAKVEYREMDESL-127-K-biotin (Peptide Specialty Laboratories GmbH)] in 1 mL assay buffer for 1.5 h at 4 °C on a rotator. Meanwhile, 500 μg of cell lysate was incubated with compound (0–400 μM) in 250 μL assay buffer on ice. The beads with bound peptide were briefly washed (2 × 1 mL, 1 min) with assay buffer and then incubated with the cell lysates [250 μL + 750 μL assay buffer (reducing the ligand concentration to 0–100 μM)] on a rotator at 4 °C overnight. After overnight incubation, beads were washed (3 × 1 mL, 10 min) with assay buffer, resuspended in 1x SDS-PAGE sample buffer, and heated at 95 °C for 5 min. Bound proteins were resolved by SDS-PAGE on 8% polyacrylamide gels. 100 μg (20%) of HEK293T lysate served as an input control. Western blotting was performed according to standard procedures. Membranes (Immobilon-P, Millipore) were blocked for 1 h in PBS buffer supplemented with 0.1% Tween 20 and 5% (w/v) skim milk (1h rt), decorated with anti-CHD1 antibody (BETHYL, A301–218A, 1:2500) at 4 °C overnight, and then decorated with mouse anti-rabbit IgG-HRP secondary antibody (Cell Signaling, 5127, 1:10,000) for 45 min at rt. Finally, membranes were incubated with ECL Select Western Blotting Detection Reagent (Cytiva) and signals recorded on an Amersham Imager 600 (GE Healthcare). Signal intensities were quantified using the build-in software provided by the manufacturer.

### 3-(4,5-Dimethylthiazol-2-yl)-2,5-diphenyl-2*H*-tetrazolium Bromide (MTT) Assay

Cell proliferation was determined using the CellTiter 96 Non-Radioactive Cell Proliferation Assay (MTT) kit (Promega) essentially as described by the manufacturer. PC-3M-Luc (2500 cells/well) or LNCaP (5000 cells/well) were seeded in 96-well plates in the presence of compound or vehicle with a final concentration of 0.1% DMSO. After 72 h, MTT solution was added. The absorbance was measured with a BMG LABTECH FLUOstar OMEGA plate reader (BMG Labtechnologies, Germany). Experiments were performed in triplicate, and EC_50_ values were calculated using Prism (GraphPad Software, San Diego, CA, USA).

### Kinase Selectivity Assay

The kinase selectivity profile of KMI169 at 10 μM was validated by the KINOMEscan^50^ Profiling Service performed at Eurofins DiscoverX Corporation, San Diego, USA. Compound-kinase interactions were tested with 97 representative kinases belonging to the AGC, CAMK, CMGC, CK1, STE, TK, TKL, lipid, and atypical kinase families including important mutant forms (scanEDGE Kinase Panel).

### Molecular Modeling

Protein–ligand contacts in solved X-ray structures were analyzed with the Protein–Ligand Interaction Profiler (PLIP) program^39^ and visualized in PyMOL (Schrödinger LLC, NY, USA). Structure-based lead optimization was performed with the tools of the Schrödinger Suite 2019–1 (Schrödinger LLC, NY, USA), as previously described.^59^ Briefly, the crystal structure of the CHD1 tCD/**2b** complex was prepared with the program Protein PrepWizard.^60^ The center of mass of the ligand (**2b**) was considered as the docking grid centroid. Designed analogs were docked with a core constraint docking using the program Glide,^61^ with the Standard Precision (SP) scoring function.

## Supplementary Material





## Data Availability

Additional tables and figures can be found in the Supporting Information. Atomic coordinates and structure factors for CHD1 tCD/**2b** (PDB 9T9E), CHD1 tCD/**2l** (PDB 9T9F), CHD1 tCD/**1q** (PDB 9T9G), CHD1 tCD/**2n** (PDB 9T9H), and CHD1 tCD/**2s** (PDB 9T9I) have been deposited in the Protein Data Bank (www.rcsb.org). The authors will release the atomic coordinates upon article publication.

## Notes

Chemical structures of compound **29**, **UNC0379**, **BIX01294** (Figures S1A-C), and 2**s- TAMRA** (Figure S8D); design and validation of the FRET assay (Figure S1D-I); original ITC data (Figures S2, S3, S4, S5, S6D, S6E, S7A, S8A, and S8E); structure comparison for **2l** vs **2b** (Figures S6A), 2**l** vs H3K4me3 (Figures S6B), 2**l** vs LSD1-K114me2 depicting interactions of D425 with the ligand or the N-terminus of an α-helix (Figures S6C), 2**n** vs **1q** (Figures S7B), and 2**n** vs **2s** (Figure S8C); FTSA for CHD1 tCD and **2n** and **2s** (Figure S8B); FTSA for METTL21B and **2n** and **2s** (Figure S8F); MTT assay for the control compound **2j** (Figure S8G); IC_50_ determination for MTT assays with PC-3M-Luc and 22Rv1 cells (Figure S8H, I); validation of CHD1 knockdown efficiencies in PC-3M-Luc, LNCaP-Luc, and 22Rv1 cells (Figure S8J); synthesis of compound intermediates (Schemes S1–4); tables with crystallographic data collection and refinement statistics (Table S1), results of kinase selectivity screening (Table S2), list of peptides used in this study (Table S3), and compound SMILES (Table S4); supplementary references; HPLC chromatograms of compounds (Figure S9–44); ^1^H- and ^13^C NMR spectra of compounds (Figure S45–116) (PDF)
Molecular formula strings for the tested compounds (CSV)

## Abbreviations Used

BPA: *N*-(benzylpiperidin-4-yl)­amine
BRET: bioluminescence resonance energy transfer
calcd.: calculated
CHD: chromo and helicase domain
CETSA: cellular thermal shift assay
DPD: *N* ^3^ *,N* ^3^-dimethylpropane-1,3-diamine
equiv.: equivalent
FRET: Förster resonance energy transfer
FTSA: fluorescent thermal shift assay
GST: glutathione-S-transferase
GFP: green fluorescent protein
H3K4me3: histone H4 lysine 4 trimethyl
His: hexahistidine tag
ITC: isothermal titration calorimetry
LSD1: lysine-specific demethylase 1
LSD1-K114me2: LSD1 lysine 114 dimethyl
MBT: malignant brain tumor
MTT: 3-(4,5-dimethylthiazol-2-yl)-2,5-diphenyl-2H-tetrazolium bromide
no bd.: no binding
n.d.: not determined
PCa: prostate cancer
PHD: plant homeodomain
PTEN: phosphatase and tensin homologue
PWWP: proline–tryptophan–tryptophan–proline
rt: room temperature
TAMRA: 5-carboxytetramethylrhodamine
tCD: tandem chromodomain
WD40: tryptophan-aspartate 40

## References

[1] Marfella C. G. A., Imbalzano A. N. (2007). The Chd family of chromatin remodelers. Mutat. Res., Fundam. Mol. Mech. Mutagen..

[2] Liu C., Kang N., Guo Y., Gong P. (2021). Advances in Chromodomain
Helicase DNA-Binding (CHD) Proteins Regulating Stem Cell Differentiation
and Human Diseases. Front. Cell Dev. Biol..

[3] Alendar, Anton A. B. (2021). Sentinels
of chromatin:
chromodomain helicase DNA-binding proteins in development and disease. Genes Dev..

[4] Flanagan J. F., Mi L.-Z., Chruszcz M., Cymborowski M., Clines K. L., Kim Y., Minor W., Rastinejad F., Khorasanizadeh S. (2005). Double chromodomains cooperate to
recognize the methylated
histone H3 tail. Nature.

[5] Metzger E., Willmann D., McMillan J., Forne I., Metzger P., Gerhardt S., Petroll K., von Maessenhausen A., Urban S., Schott A.-K. (2016). Assembly
of methylated
KDM1A and CHD1 drives androgen receptor–dependent transcription
and translocation. Nat. Struct. Mol. Biol..

[6] Qin S., Liu Y., Tempel W., Eram M. S., Bian C., Liu K., Senisterra G., Crombet L., Vedadi M., Min J. (2014). Structural
basis for histone mimicry and hijacking of host proteins by influenza
virus protein NS1. Nat. Commun..

[7] Taverna S. D., Li H., Ruthenburg A. J., Allis C. D., Patel D. J. (2007). How chromatin-binding
modules interpret histone modifications: lessons from professional
pocket pickers. Nat. Struct. Mol. Biol..

[8] Cipriano A., Sbardella G., Ciulli A. (2020). Targeting epigenetic reader domains
by chemical biology. Curr. Opin. Chem. Biol..

[9] Arrowsmith C. H., Schapira M. (2019). Targeting non-bromodomain
chromatin readers. Nat. Struct. Mol. Biol..

[10] Ortiz G., Kutateladze T. G., Fujimori D. G. (2023). Chemical tools targeting readers
of lysine methylation. Curr. Opin. Chem. Biol..

[11] Huang X., Chen Y., Xiao Q., Shang X., Liu Y. (2024). Chemical inhibitors
targeting histone methylation readers. Pharmacol.
Ther..

[12] James L. I., Barsyte-Lovejoy D., Zhong N., Krichevsky L., Korboukh V. K., Herold J. M., MacNevin C. J., Norris J. L., Sagum C. A., Tempel W. (2013). Discovery of a chemical
probe for the L3MBTL3 methyllysine reader domain. Nat. Chem. Biol..

[13] Stuckey J. I., Dickson B. M., Cheng N., Liu Y., Norris J. L., Cholensky S. H., Tempel W., Qin S., Huber K. G., Sagum C. (2016). A cellular chemical probe targeting the chromodomains
of Polycomb repressive complex 1. Nat. Chem.
Biol..

[14] Ren C., Smith S. G., Yap K., Li S., Li J., Mezei M., Rodriguez Y., Vincek A., Aguilo F., Walsh M. J., Zhou M. M. (2016). Structure-Guided Discovery of Selective
Antagonists for the Chromodomain of Polycomb Repressive Protein CBX7. ACS Med. Chem. Lett..

[15] Wagner T., Greschik H., Burgahn T., Schmidtkunz K., Schott A.-K., McMillan J., Baranauskienė L., Xiong Y., Fedorov O., Jin J. (2016). Identification
of a small-molecule ligand of the epigenetic reader protein Spindlin1
via a versatile screening platform. Nucleic
Acids Res..

[16] Fagan V., Johansson C., Gileadi C., Monteiro O., Dunford J. E., Nibhani R., Philpott M., Malzahn J., Wells G., Faram R. (2019). A Chemical Probe for Tudor Domain Protein Spindlin1
to Investigate Chromatin Function. J. Med. Chem..

[17] Böttcher J., Dilworth D., Reiser U., Neumüller R. A., Schleicher M., Petronczki M., Zeeb M., Mischerikow N., Allali-Hassani A., Szewczyk M. M. (2019). Fragment-based discovery
of a chemical probe for the PWWP1 domain of NSD3. Nat. Chem. Biol..

[18] Dilworth D., Hanley R. P., Ferreira de Freitas R., Allali-Hassani A., Zhou M., Mehta N., Marunde M. R., Ackloo S., Machado R. A. C., Yazdi A. K. (2022). A chemical
probe targeting
the PWWP domain alters NSD2 nucleolar localization. Nat. Chem. Biol..

[19] Guo Y., Mao X., Xiong L., Xia A., You J., Lin G., Wu C., Huang L., Wang Y., Yang S. (2021). Structure-Guided Discovery
of a Potent and Selective Cell-Active Inhibitor of SETDB1 Tudor Domain. Angew. Chem., Int. Ed..

[20] Bae N., Viviano M., Su X., Lv J., Cheng D., Sagum C., Castellano S., Bai X., Johnson C., Khalil M. I. (2017). Developing Spindlin1
small-molecule inhibitors
by using protein microarrays. Nat. Chem. Biol..

[21] Xiong Y., Greschik H., Johansson C., Seifert L., Gamble V., Park K.-s., Fagan V., Li F., Chau I., Vedadi M. (2024). Discovery of a Potent, Selective, and Cell-Active SPIN1
Inhibitor. J. Med. Chem..

[22] Johnson R. L., Graboski A. L., Li F., Norris-Drouin J. L., Walton W. G., Arrowsmith C. H., Redinbo M. R., Frye S. V., James L. I. (2024). Discovery of CHD1
Antagonists for PTEN-Deficient Prostate
Cancer. J. Med. Chem..

[23] Li H., Gigi L., Zhao D. (2023). CHD1, a multifaceted
epigenetic remodeler
in prostate cancer. Front. Oncol..

[24] Kumar K. S. P., Jyothi M. N., Prashant A. (2025). CHD1 dysregulation in cancer: bridging
chromatin instability, therapy resistance, and immune evasion. Mol. Biol. Rep..

[25] Arora K., Barbieri C. E. (2018). Molecular Subtypes
of Prostate Cancer. Curr. Oncol. Rep..

[26] Boysen G., Rodrigues D. N., Rescigno P., Seed G., Dolling D., Riisnaes R., Crespo M., Zafeiriou Z., Sumanasuriya S., Bianchini D. (2018). SPOP-Mutated/CHD1-Deleted
Lethal Prostate Cancer and Abiraterone Sensitivity. Clin. Cancer Res..

[27] Rodrigues L. U., Rider L., Nieto C., Romero L., Karimpour-Fard A., Loda M., Lucia M. S., Wu M., Shi L., Cimic A. (2015). Coordinate Loss of MAP3K7
and CHD1 Promotes Aggressive
Prostate Cancer. Cancer Res..

[28] Orme J. J., Antonarakis E. S., Dehm S. M. (2025). CHD1 status drives divergent metabolic
pathways in SPOP-mutant prostate cancer. Nat.
Cancer.

[29] Chen F., Li H., Wang Y., Tang X., Lin K., Li Q., Meng C., Shi W., Leo J., Liang X. (2025). CHD1 loss reprograms SREBP2-driven cholesterol synthesis to fuel
androgen-responsive growth and castration resistance in SPOP-mutated
prostate tumors. Nat. Cancer.

[30] Zhao D., Lu X., Wang G., Lan Z., Liao W., Li J., Liang X., Chen J. R., Shah S., Shang X. (2017). Synthetic essentiality
of chromatin remodelling factor CHD1 in PTEN-deficient
cancer. Nature.

[31] Zhao D., Cai L., Lu X., Liang X., Li J., Chen P., Ittmann M., Shang X., Jiang S., Li H. (2020). Chromatin
Regulator CHD1 Remodels the Immunosuppressive Tumor Microenvironment
in PTEN-Deficient Prostate Cancer. Cancer Discovery.

[32] Li H., Wang Y., Lin K., Venkadakrishnan V. B., Bakht M., Shi W., Meng C., Zhang J., Tremble K., Liang X. (2022). CHD1 Promotes Sensitivity
to Aurora Kinase Inhibitors by Suppressing Interaction of AURKA with
Its Coactivator TPX2. Cancer Res..

[33] Shenoy T. R., Boysen G., Wang M. Y., Xu Q. Z., Guo W., Koh F. M., Wang C., Zhang L. Z., Wang Y., Gil V. (2017). CHD1 loss sensitizes prostate cancer to DNA damaging
therapy by promoting error-prone double-strand break repair. Ann. Oncol..

[34] Kari V., Mansour W. Y., Raul S. K., Baumgart S. J., Mund A., Grade M., Sirma H., Simon R., Will H., Dobbelstein M. (2016). Loss of CHD1 causes DNA repair defects and
enhances prostate cancer therapeutic responsiveness. EMBO Rep..

[35] Santiago C., Nguyen K., Schapira M. (2011). Druggability
of methyl-lysine binding
sites. J. Comput.-Aided Mol. Des..

[36] Menna M., Fiorentino F., Marrocco B., Lucidi A., Tomassi S., Cilli D., Romanenghi M., Cassandri M., Pomella S., Pezzella M. (2022). Novel non-covalent LSD1
inhibitors endowed with anticancer effects in leukemia and solid tumor
cellular models. Eur. J. Med. Chem..

[37] Ma A., Yu W., Li F., Bleich R. M., Herold J. M., Butler K. V., Norris J. L., Korboukh V., Tripathy A., Janzen W. P. (2014). Discovery
of a Selective, Substrate-Competitive Inhibitor of the
Lysine Methyltransferase SETD8. J. Med. Chem..

[38] Kubicek S., O’Sullivan R. J., August E. M., Hickey E. R., Zhang Q., Teodoro M. L., Rea S., Mechtler K., Kowalski J. A., Homon C. A. (2007). Reversal
of H3K9me2 by a Small-Molecule Inhibitor
for the G9a Histone Methyltransferase. Mol.
Cell.

[39] Schake P., Bolz S. N., Linnemann K., Schroeder M. (2025). PLIP 2025:
introducing protein–protein interactions to the protein–ligand
interaction profiler. Nucleic Acids Res..

[40] Chang Y., Ganesh T., Horton J. R., Spannhoff A., Liu J., Sun A., Zhang X., Bedford M. T., Shinkai Y., Snyder J. P., Cheng X. (2010). Adding a lysine
mimic in the design
of potent inhibitors of histone lysine methyltransferases. J. Mol. Biol..

[41] Speranzini V. R. D., Rotili D., Ciossani G., Pilotto S., Marrocco B., Forgione M., Lucidi A., Forneris F., Mehdipour P., Velankar S., Mai A., Mattevi A. (2016). Polymyxins and quinazolines
are LSD1/KDM1A inhibitors with unusual structural features. Sci. Adv..

[42] Rotili D., Tarantino D., Marrocco B., Gros C., Masson V., Poughon V., Ausseil F., Chang Y., Labella D., Cosconati S. (2014). Properly Substituted Analogues of BIX-01294
Lose Inhibition of G9a Histone Methyltransferase and Gain Selective
Anti-DNA Methyltransferase 3A Activity. PLoS
One.

[43] Smits R. A., de Esch I. J. P., Zuiderveld O. P., Broeker J., Sansuk K., Guaita E., Coruzzi E., Adami M., Haaksma E., Leurs R. (2008). Discovery of Quinazolines
as Histamine H4 Receptor Inverse Agonists
Using a Scaffold Hopping Approach. J. Med. Chem..

[44] Wynn, T. A. ; Hodous, B. L. ; Boriack-Sjodin, P. A. ; Sickmier, E. A. ; Mills, J. E. J. ; Tasker, A. S. ; Copeland, R. A. METTL3Modulators. WO Patent WO2021081211.2021.

[45] Fleury-Brégeot N., Raushel J., Sandrock D. L., Dreher S. D., Molander G. A. (2012). Rapid and
Efficient Access to Secondary Arylmethylamines. Chem. - Eur. J..

[46] De
Simone A., Georgiou C., Ioannidis H., Gupta A. A., Juárez-Jiménez J., Doughty-Shenton D., Blackburn E. A., Wear M. A., Richards J. P., Barlow P. N. (2019). A computationally designed binding mode flip
leads to a novel class of potent tri-vector cyclophilin inhibitors. Chem. Sci..

[47] Wang S., Klein S. O., Urban S., Staudt M., Barthes N. P. F., Willmann D., Bacher J., Sum M., Bauer H., Peng L. (2024). Structure-guided design of a selective inhibitor of
the methyltransferase KMT9 with cellular activity. Nat. Commun..

[48] Pulido-Cortés L., Gielingh H., Thijssen V., Liu M., Yoshisada R., Soares L. R., Nizamuddin S., Friedrich F., Greschik H., Peng L. (2025). Molecular
determinants
for recognition of serotonylated chromatin. Nucelic Acids Res..

[49] Willmann D., Lim S., Wetzel S., Metzger E., Jandausch A., Wilk W., Jung M., Forne I., Imhof A., Janzer A. (2012). Impairment of prostate cancer cell growth by a selective
and reversible lysine-specific demethylase 1 inhibitor. Int. J. Cancer.

[50] Niesen F. H., Berglund H., Vedadi M. (2007). The use of differential scanning
fluorimetry to detect ligand interactions that promote protein stability. Nat. Protoc..

[51] Vonrhein C., Flensburg C., Keller P., Sharff A., Smart O., Paciorek W., Womack T., Bricogne G. (2011). Data processing and
analysis with theautoPROCtoolbox. Acta Crystallogr.,
Sect. D:Biol. Crystallogr..

[52] Winn M. D., Ballard C. C., Cowtan K. D., Dodson E. J., Emsley P., Evans P. R., Keegan R. M., Krissinel E. B., Leslie A. G. W., McCoy A. (2011). Overview
of theCCP4
suite and current developments. Acta Crystallogr.,
Sect. D:Biol. Crystallogr..

[53] McCoy A. J., Grosse-Kunstleve R. W., Adams P. D., Winn M. D., Storoni L. C., Read R. J. (2007). Phasercrystallographic
software. J. Appl. Crystallogr..

[54] Emsley P., Lohkamp B., Scott W. G., Cowtan K. (2010). Features and development
of Coot. Acta Crystallogr., Sect. D:Biol. Crystallogr..

[55] Murshudov G. N., Skubák P., Lebedev A. A., Pannu N. S., Steiner R. A., Nicholls R. A., Winn M. D., Long F., Vagin A. A. (2011). REFMAC5
for the refinement of macromolecular crystal structures. Acta Crystallogr., Sect. D:Biol. Crystallogr..

[56] Adams P. D., Afonine P. V., Bunkóczi G., Chen V. B., Davis I. W., Echols N., Headd J. J., Hung L.-W., Kapral G. J., Grosse-Kunstleve R. W. (2010). PHENIX: a comprehensive Python-based system
for macromolecular structure solution. Acta
Crystallogr., Sect. D:Biol. Crystallogr..

[57] Chen V. B., Arendall W. B., Headd J. J., Keedy D. A., Immormino R. M., Kapral G. J., Murray L. W., Richardson J. S., Richardson D. C. (2010). MolProbity: all-atom structure validation
for macromolecular
crystallography. Acta Crystallogr., Sect. D:Biol.
Crystallogr..

[58] Seitz J., Auth M., Prinz T., Hau M., Tzortzoglou P., Schulz-Fincke J., Schmidtkunz K., Baniahmad A. A., Willmann D., Metzger E. (2024). Soft drug
inhibitors
for the epigenetic targets lysine-specific demethylase 1 and histone
deacetylases. Arch. Pharm..

[59] Wang S., Barthes N. P. F., Urban S., Hazai V. I., Klein S. O., Pappert T., Kummel P., Heller N., Bacher J., Staudt M. (2025). Structure-Guided Design
of a KMT9 Inhibitor Prodrug
with Cellular Activity. J. Med. Chem..

[60] Sastry G. M., Adzhigirey M., Day T., Annabhimoju R., Sherman W. (2013). Protein and ligand preparation: parameters, protocols,
and influence on virtual screening enrichments. J. Comput.-Aided Mol. Des..

[61] Friesner R. A. B., Banks J. L., Murphy R. B., Halgren T. A., Klicic J. J., Mainz D. T., Repasky M. P., Knoll E. H., Shelley M., Perry J. K., Shaw D. E., Francis P., Shenkin P. S. (2004). Glide:
A New Approach for Rapid, Accurate Docking and Scoring. 1. Method
and Assessment of Docking Accuracy. J. Med.
Chem..

