# Supplementary material for: Development of High-Affinity CHD1 Chromodomain Inhibitors
Source: J Med Chem. 2026 May 5;69(10):12020–47. doi: 10.1021/acs.jmedchem.5c03690 (PMC13224165; doi:10.1021/acs.jmedchem.5c03690)
Supplement: Supplementary file 1 [file jm5c03690_si_001.pdf]

Supporting Information

Development of high-affinity CHD1 chromodomain inhibitors

Holger Greschik[1], Florian Friedrich[2], Ludwig Seifert[2], Farnoush Mousavizadeh[3], Francesco Fiorentino[4], Johannes Walz[3], Lin Zhang[5], Jianyu Li[6], Emanuele Fabbrizi[7], Stefano Tomassi[8], Farhad Panahi[3], Niklas Papenkordt[2], Silas L. Wurnig[2], Johannes Osterroth[2], Anna M. Strasser[2], Jan Ruprecht[2], Aurélien F.A. Moumbock[6], Martin Hügle[6], Manuela Sum[1], Ling Peng[1], Sheng Wang[1], Adina A. Baniahmad[2], Laura Pulido-Cortés[9], H. Th. Marc Timmers[9], Ralf Flaig[10,11], Eric Metzger[1,9], Bernhard Breit[3], Oliver Einsle[5], Stefan Günther[6], Dante Rotili[12,*], Antonello Mai[7,*], Roland Schüle[1,9,*], and Manfred Jung[2,9,*]

[1]Department of Urology and Center for Clinical Research, University Freiburg Medical Center, Breisacher Str. 66, 79106 Freiburg, Germany.

[2]Institute of Pharmaceutical Sciences, University of Freiburg, Albertstr. 25, 79104 Freiburg, Germany.

[3]Institute of Organic Chemistry, University of Freiburg, Albertstr. 21, 70104 Freiburg, Germany.

[4]Department of Biochemical Sciences, Sapienza University of Rome, P.le Aldo Moro 5, 00185 Rome, Italy.

[5]Institute of Biochemistry, University of Freiburg, 79104 Freiburg, Germany.

[6]Institute of Pharmaceutical Sciences, University of Freiburg, Hermann-Herder-Str. 9, 79104 Freiburg, Germany.

[7]Department of Drug Chemistry and Technologies, Sapienza University of Rome, P.le Aldo Moro 5, 00185 Rome, Italy.

[8]Department of Life Science, Health and Health Professions, LINK Campus University, Via del Casale di San Pio V, 44, CAP 00165 Rome, Italy.

[9]German Cancer Consortium (DKTK), partner site Freiburg; University Medical Center Freiburg, Breisacher Str. 66, 79106 Freiburg, Germany.

[10]Diamond Light Source Ltd, Harwell Science & Innovation Campus, Didcot, Oxfordshire OX11 0DE, United Kingdom.

[11]Research Complex at Harwell, Rutherford Appleton Laboratory, Didcot OX11 0FA, United Kingdom.

[12]Department of Science, Roma Tre University of Rome, Viale Guglielmo Marconi 446, 00146 Rome, Italy.

* Corresponding authors:

Prof. Dr. Manfred Jung, Institute of Pharmaceutical Sciences, University of Freiburg, Albertstr. 25, 79104 Freiburg, Germany.
E-mail: manfred.jung@pharmazie.uni-freiburg.de

Prof. Dr. Roland Schüle, Department of Urology and Center for Clinical Research, University Freiburg Medical Center, Breisacher Str. 66, 79106 Freiburg, Germany.
E-mail: roland.schuele@uniklinik-freiburg.de

Prof. Dr. Antonello Mai, Department of Drug Chemistry and Technologies, Sapienza University of Rome, P.le Aldo Moro 5, 00185 Rome, Italy.
E-mail: antonello.mai@uniroma1.it

Prof. Dr. Dante Rotili, Department of Science, Roma Tre University, Viale Guglielmo Marconi 446, 00146 Rome, Italy.
E-mail: dante.rotili@uniroma3.it

TABLE OF CONTENTS

**S3**	**Figure S1**
**S4**	**Figure S2, Figure S3**
**S5**	**Figure S4, Figure S5**
**S6**	**Figure S6**
**S7**	**Figure S7**
**S8**	**Figure S8**
**S9**	**Supplementary Synthesis Information**
**S10**	**Supplementary Synthesis Schemes S1–S4**
**S11**	**Table S1 (Xray Statistics)**
**S12**	**Table S2 (Kinase Selectivity Screen)**
**S13**	**Table S3 (Peptides)**
**S14**	**Table S4 (Compound SMILES)**
**S15**	**Supplementary References**
**S16–S34**	**Figure S9–S44 (Compound HPLC Spectra)**
**S35–S70**	**Figure S45–S116 (Compound ^{1}H NMR and ^{13}C NMR Spectra)**

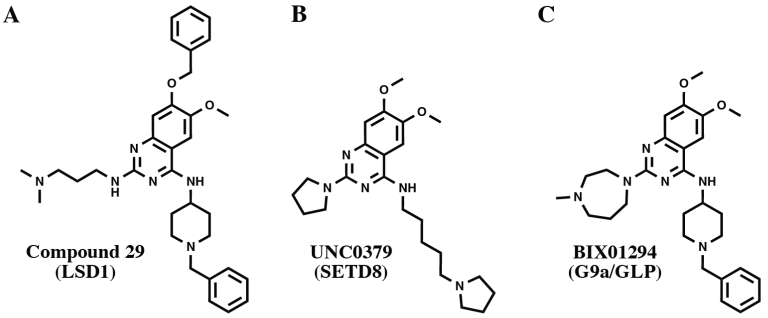

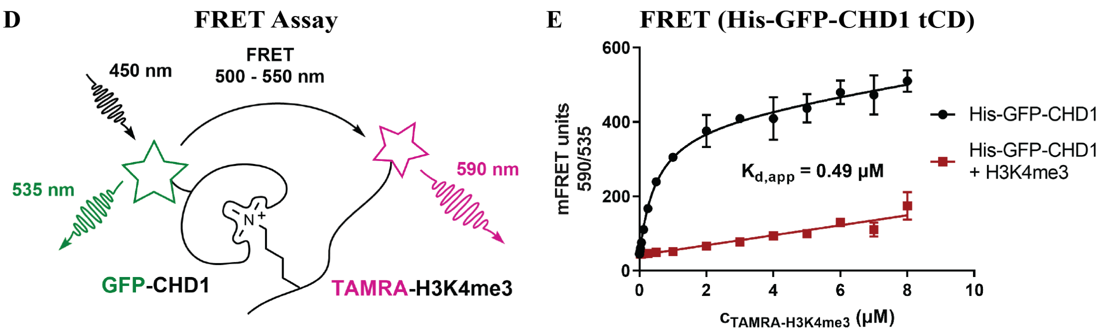

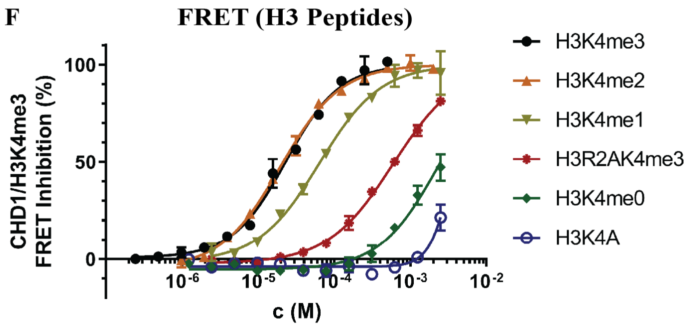

G

Peptide	FRET IC_{50} [µM]
H3K4me3	23 ± 1
H3K4me2	20 ± 1
H3K4me1	64 ± 6
H3R2AK4me3	600 ± 20
H3K4me0	2300 ± 100
H3K4A	4000 ± 1000

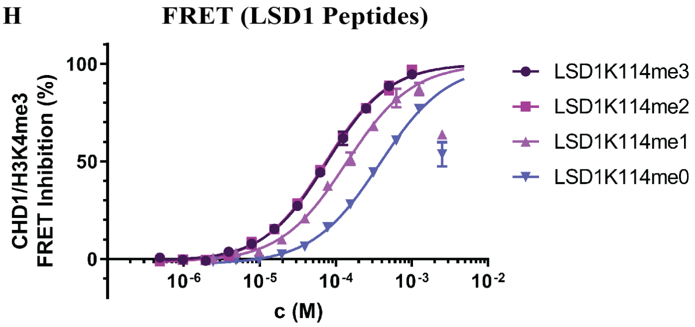

I

Peptide	FRET IC_{50} [µM]
LSD1-K114me3	76 ± 2
LSD1-K114me2	72 ± 2
LSD1-K114me1	137 ± 6
LSD1-K114me0	377 ± 7

Figure S1: Selected quinazoline-based inhibitors of epigenetic regulators (**A**–**C**) and CHD1 FRET assay (**D**–**I**). (**A**) Compound **29** (LSD1),[1] (**B**) **UNC0379** (SETD8),[2] (**C**) **BIX01294** [G9a (EHMT2) and GLP (EHMT1)].[3] (**D**) Schematic representation of the FRET assay used for CHD1 tCD ligand screening. (**E**) Determination of the apparent K_d ($K_{d,\ app}$) for the binding of TAMRA-labelled H3K4me3 peptide (TAMRA-H3K4me3) to GFP-CHD1 tCD in FRET assay. (**F**–**I**) Structure-activity relationship (SAR) of the indicated H3 (**F**, **G**) or LSD1 peptides (**H**, **I**) inhibiting the GFP-CHD1 tCD/TAMRA-H3K4me3 interaction in FRET assays.

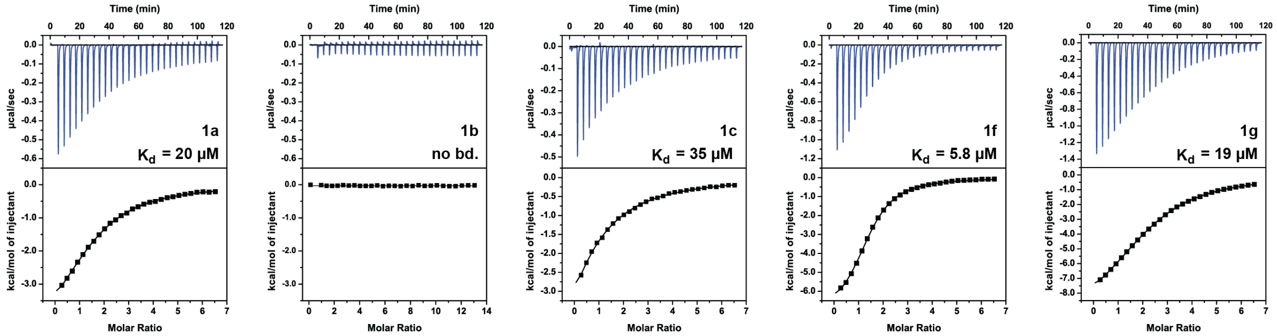

Figure S2: Identification of an initial hit and truncation at C2, C4, C6, and C7 of the quinazoline scaffold. ITC data for compounds **1a**–**1c**, **1f**, and **1g** listed in Figure 2. ITC was performed with 20 µM CHD1 tCD in the sample cell and 600–1200 µM of indicated compound in the syringe.

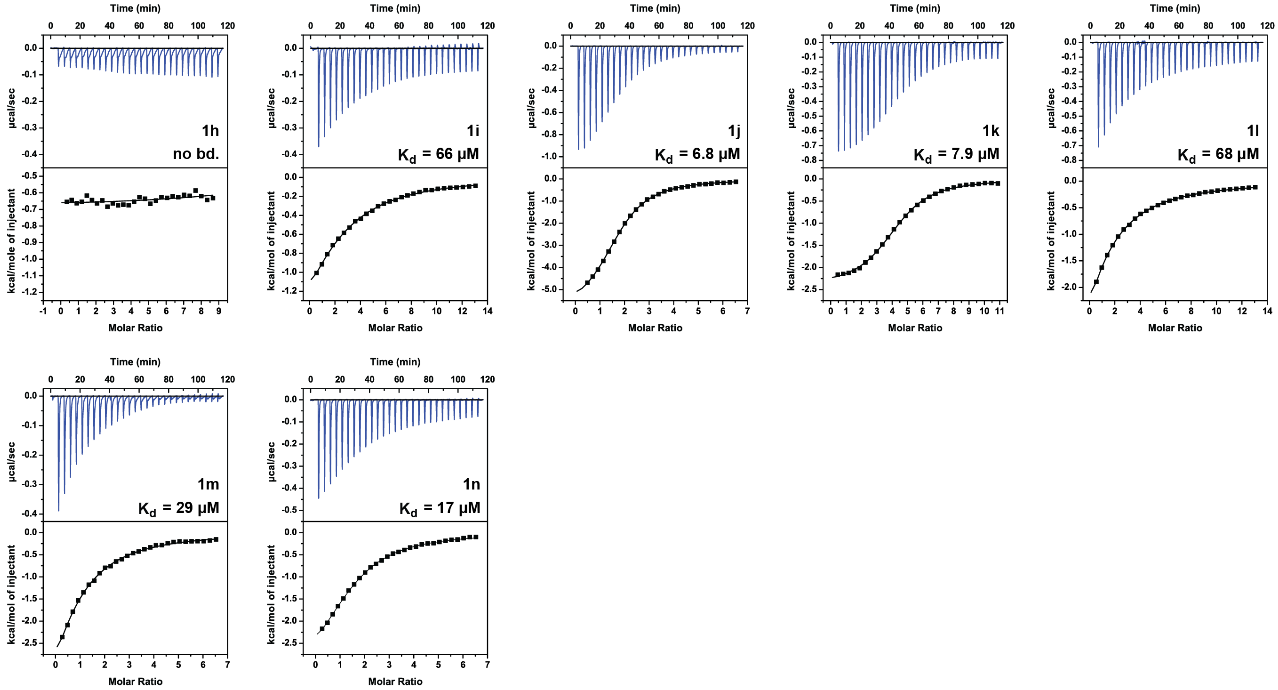

Figure S3: Focused SAR study at C4 of the quinazoline scaffold. ITC data for compounds **1h**–**1n** listed in Figure 3. ITC was performed with 20 µM CHD1 tCD in the sample cell and 600–1200 µM of indicated compound in the syringe.

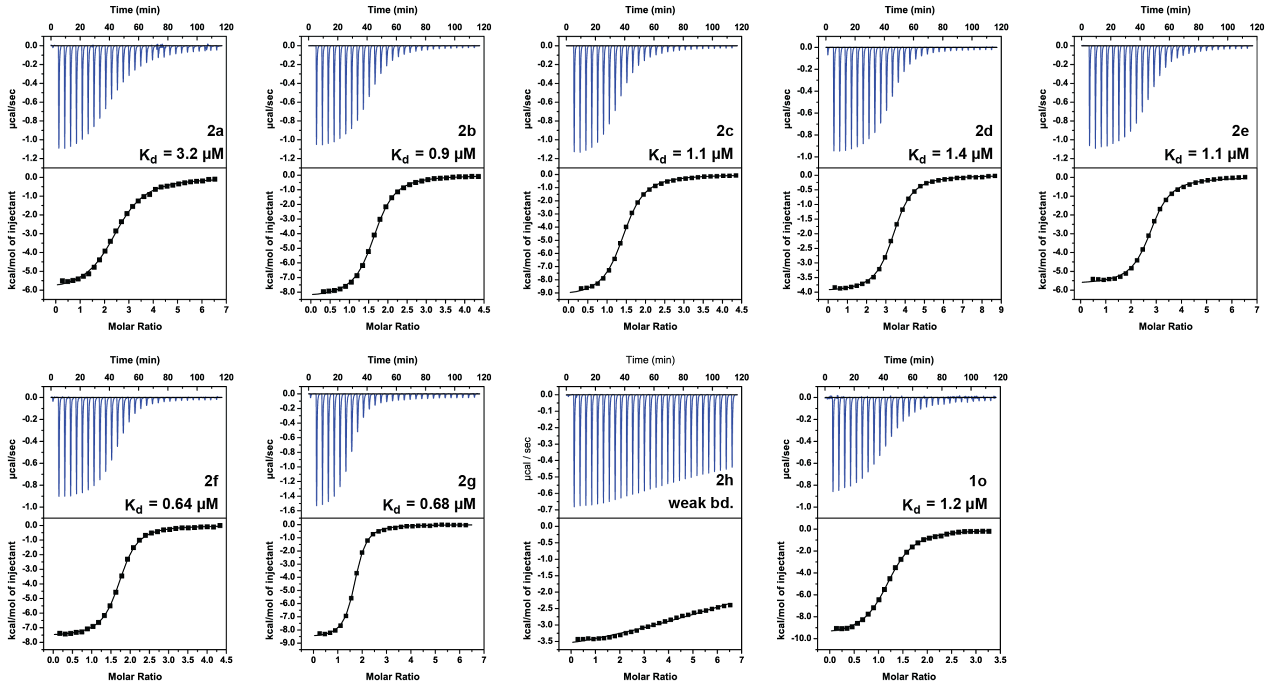

Figure S4: Quinazoline-to-quinoline switch and focused SAR study at C7 of the quinoline or quinazoline scaffold. ITC data for compounds **2a**–**2h** and **1o** listed in Figure 4. ITC was performed with 20 µM CHD1 tCD in the sample cell and 300–800 µM of indicated compound in the syringe.

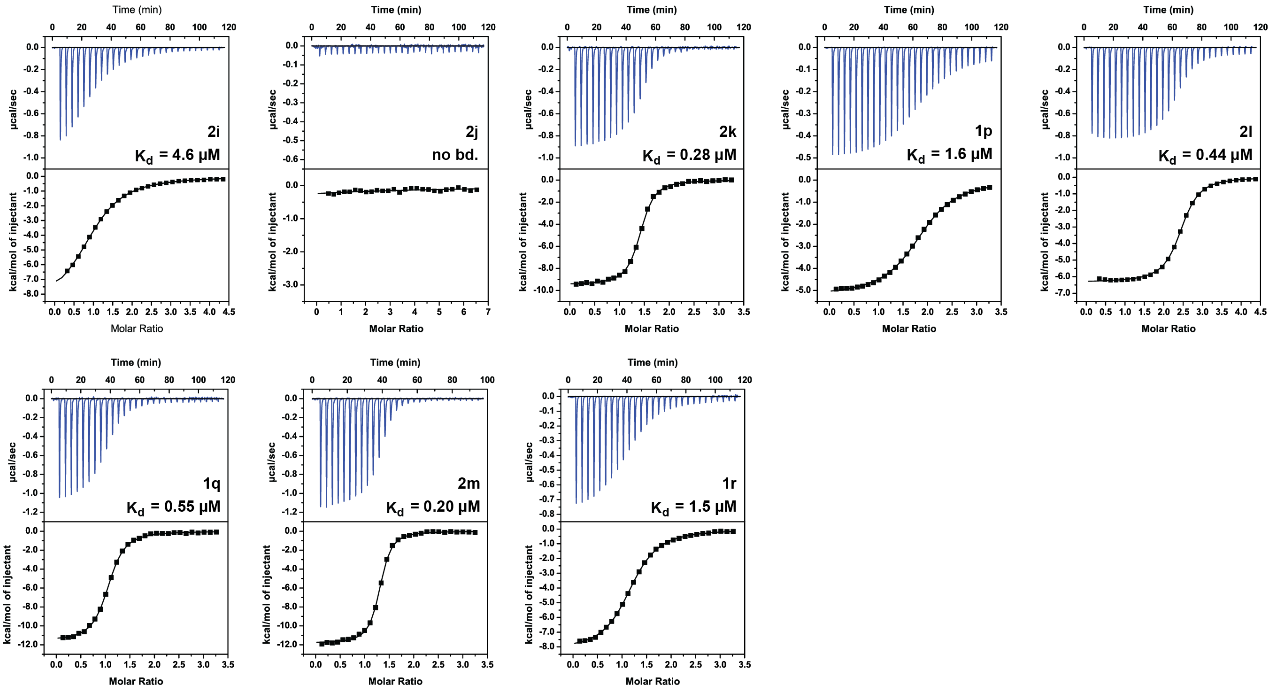

Figure S5: Focused SAR study at C2 of the quinoline or quinazoline scaffold. ITC data for compounds **2i**–**2m** and **1p**–**1r** listed in Figure 5. ITC was performed with 20 µM CHD1 tCD in the sample cell and 300–400 µM of indicated compound in the syringe.

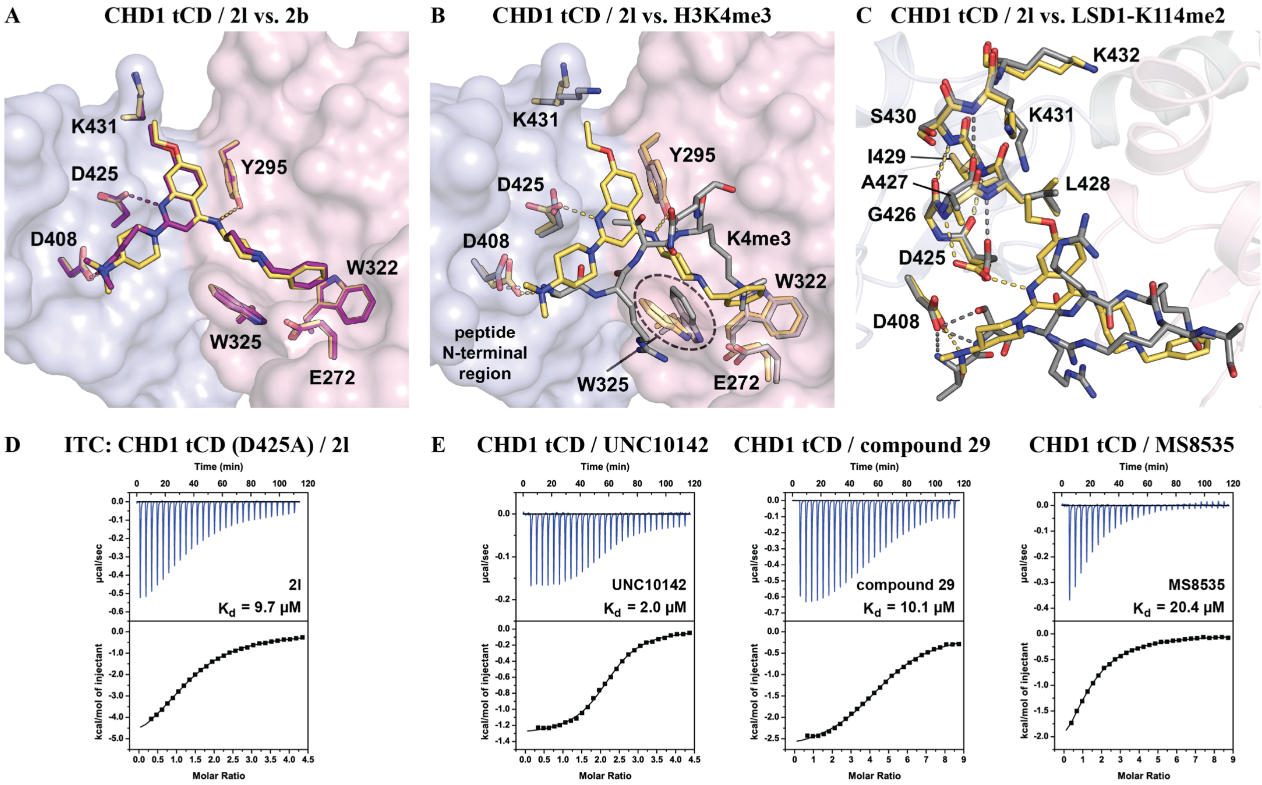

Figure S6: Crystallographic analysis of compound binding to the CHD1 tCD. (**A**) Superimposition of the CHD1 tCD in complex with **2l** (yellow) or **2b** (dark violet). (**B**) Superimposition of the CHD1 tCD in complex with a H3K4me3 peptide (grey; PDB code 2B2W) or **2l** (yellow). The side chain of W325 is slightly tilted in the CHD1 tCD/**2l** complex relative to its position in the CHD1 tCD/H3K4me3 peptide complex thereby accommodating the bulkier benzyl-piperidine moiety of **2l**. (**C**) Superimposition of the CHD1 tCD in complex with **2l** (yellow) or LSD1-K114me2 peptide (grey; PDB code 5AFW). Note the rotation of the side chain of D425 to form alternative contacts with a main chain nitrogen atom of the α-helix and N2 of the quinoline scaffold of **2l**. (**D**, **E**) ITC data for binding of **2l** to the mutated CHD1 tCD (D425A) (**D**) or of **UNC10142**, compound **29**, and **MS8535** to the wild-type CHD1 tCD (**E**). ITC was performed with 20 µM of wild-type or mutant CHD1 tCD in the sample cell and 400 µM (**2l**, **UNC10142**) or 800 µM (compound **29**, **MS8535**) of ligand in the syringe.

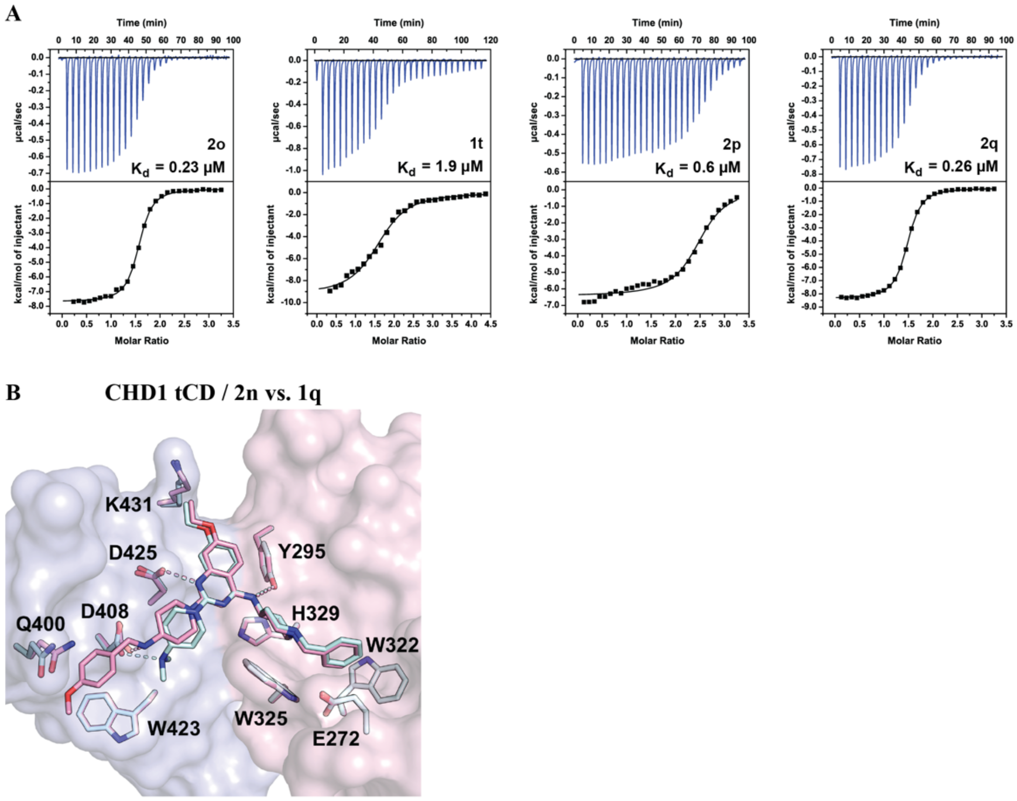

Figure S7: Further ligand extension. (**A**) ITC data for compounds listed in Figure 4. ITC was performed with 20 µM CHD1 tCD in the sample cell and 200 to 400 µM of indicated compound in the syringe. (**B**) Superimposition of the CHD1 tCD co-crystal structures with **2n** (pink) or **1q** (pale cyan). Note the small rotation of both ligands relative to one another and the stacking of **2n** with the side chain of Q400 requiring a slight conformational adaption, which is not observed in the CHD1 tCD/**1q** complex.

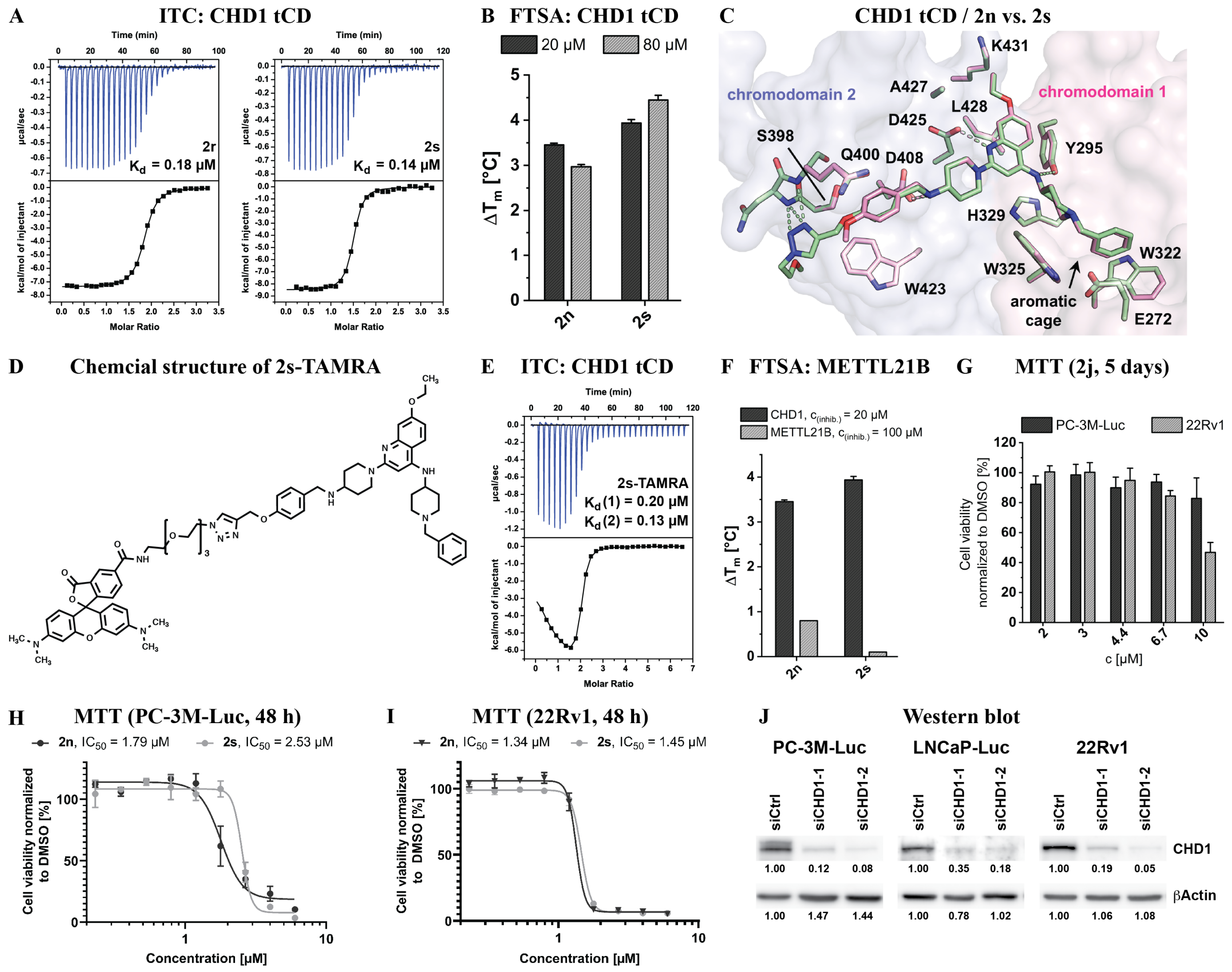

Figure S8: Target engagement and selectivity of CHD1 tCD ligands. (**A**) ITC data for **2r** and **2s**. ITC was performed with 20 µM CHD1 tCD in the sample cell and 300 µM compound in the syringe. (**B**) FTSA assay depicting the increase in melting temperature (ΔT_m) for the CHD1 tCD upon binding of **2n** or **2s** relative to the unliganded tCD. (**C**) Superimposition of the CHD1 tCD in complex with **2n** (pink) or **2s** (green). (**D**) Chemical structure of **2s-TAMRA**. (**E**) Binding of **2s-TAMRA** (600 µM in the syringe) to the CHD1 tCD (20 µM in the sample cell) in ITC assay. (**F**) FTSA assay depicting ΔT_m for METTL21B upon binding of **2n** or **2s** relative to the unliganded protein. Data for the CHD1 tCD were included as a positive control. (**G**) Cell viability of PC-3M-Luc or 22Rv1 cells upon treatment with the indicated concentrations of **2j** for 5 days determined by MTT assay. (**H, I**) IC_{50} values for concentration-dependent effects of **2n** and **2s** on the viability of PC-3M-Luc (**H**) and 22Rv1 (**I**) cells after 48 h of treatment (same MTT assays as shown in Figure 5G, H). (**J**) Western blot analysis of CHD1 expression in PC-3M-Luc, LNCaP-Luc, and 22Rv1 cells upon treatment with siCtrl, siCHD1-1, or siCHD1-2 as indicated. βActin served as a loading control. Relative, normalized signal intensities are indicated below bands.

Synthesis. The first step of the synthetic route for the preparation of intermediate **4** involves the methylation of 2-hydroxy-4-nitrobenzoic acid using iodomethane in the presence of potassium carbonate in anhydrous DMF at rt affording intermediate **28** (Scheme S1). Next, reduction of the nitro group was performed using finely powdered iron in a mixture of ethanol, acetic acid, and water (5:1:1) at rt yielding the corresponding amine **29**. Finally, cyclization to form the quinazoline-2,4(1*H*,3*H*)-dione core was achieved by treating intermediate **29** with sodium cyanate in a mixture of acetic acid and water (2:1) at rt, followed by heating under reflux with 8 M sodium hydroxide leading to the formation of **4** (Scheme S1).

Amines **9**, **10**, **14** and **15** were commercially available, while amines **11**–**13** were prepared through a synthetic route which begins with a reductive amination reaction involving piperidine-4-amine, protected with a Boc group on the exocyclic nitrogen and the appropriate aldehyde (Scheme S2). Formation of the imine was achieved by stirring the two reactants in anhydrous DCM at rt. This was followed by reduction with sodium triacetoxyborohydride (STAB) at rt yielding intermediates **30**–**32**. The tert-butoxycarbonyl protective group was then removed through acidic treatment (4 M hydrochloric acid in 1,4-dioxane) in anhydrous methanol in the presence of TIPS at rt leading to the formation of the hydrochloride salts **33**–**35**. Finally, neutralization with a saturated solution of sodium carbonate at rt afforded the free-base amines **11**, **12** and **13** (Scheme S2).

The preparation of intermediate **36** begins with a reductive amination reaction in which *N*-methylpiperidine-4-amine, protected with a Boc group on the endocyclic nitrogen, was reacted with 4-methoxybenzaldehyde in anhydrous DCM at rt. This was followed by reduction with STAB at rt to afford intermediate **37**. The tert-butoxycarbonyl protective group was then removed through acidic treatment (4 M hydrochloric acid in 1,4-dioxane) in anhydrous methanol in the presence of TIPS at rt leading to the formation of the hydrochloride salt **38**. Finally, neutralization with a saturated solution of sodium carbonate at rt yielded the free-base amine **36** (Scheme S3).

The synthesis of compounds **39** and **48** is based on a previous report (Scheme S4).[4] Briefly, 3-methoxyaniline was treated with malonic acid and $POCl_3$ at 95 °C for 30 min, followed by the addition of an additional portion of $POCl_3$. The mixture was subsequently heated to 120 °C, at which point intermediate **48** was formed. Further addition of sulfuric acid and subsequent heating at 160 °C provided quinoline derivative **39**, which was then used as a precursor for further modifications.

Scheme S1. Synthesis of intermediate 4.[a]

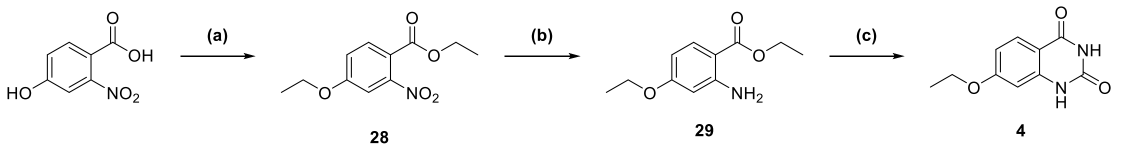

[a]Reagents and conditions: (a) iodoethane, K_2CO_3, anhydrous DMF, rt, 24 h, 69%; (b) EtOH/AcOH/H_2O (5:1:1), Fe, rt, 50 min, 92%; (c) AcOH/H_2O (2:1), NaOCN, 0 °C to rt, 18 h, then NaOH (8 M), reflux, 5 h, 93%.

Scheme S2. Synthesis of intermediates 11–13.[a]

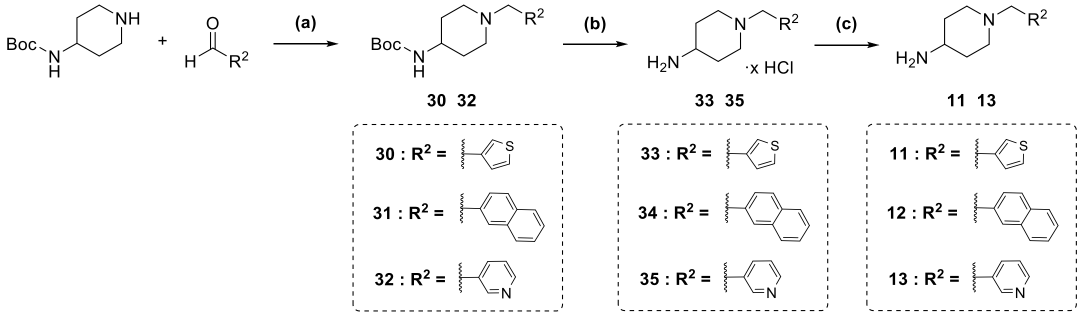

[a]Reagents and conditions: (a) anhydrous DCM, molecular sieves (4 Å), N_2, rt, 45 min, then STAB, 0 °C to rt, 23 h, 72–79%; (b) TIPS, HCl (4 M), anhydrous MeOH, 0 °C to rt, 6 h,87–92%; (c) Na_2CO_3, 0 °C to rt, 10 min, 91–95%.

Scheme S3. Synthesis of intermediate 36.[a]

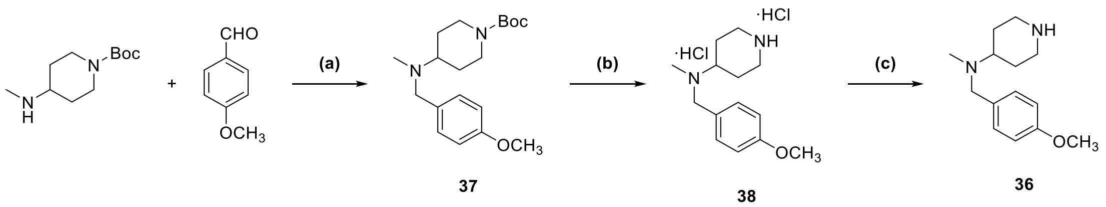

[a]Reagents and conditions: (a) anhydrous DCM, molecular sieves (4 Å), N_2, rt, 1 h, then STAB, 0 °C to rt, 21 h, 78%.; (b) TIPS, HCl (4 N), anhydrous MeOH, 0 °C to rt, 6 h, 90%; (c) $Na_2CO_{3(ss)}$, 0 °C to rt, 10 min, 85%.

Scheme S4. Synthesis of intermediates 39 and **48.**[a]

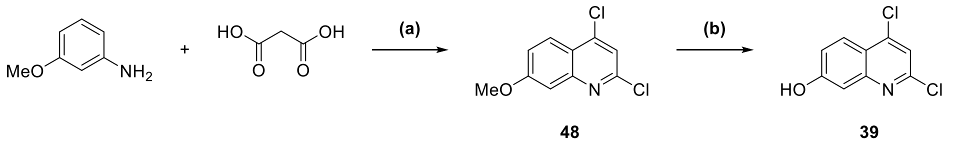

[a]Reagent and conditions: (a) $POCl_3$, rt to 95 °C, 30 min, then add more $POCl_3$, rt to 120 °C, 3 h, 58%; (b) H_2SO_4, 160 °C, 2 h, 82%.

Table S1: Crystallographic data collection and refinement statistics for CHD1 tCD/inhibitor complexes.

Structure	CHD1–**2b**	CHD1–**2l**	CHD1–**1q**	CHD1–**2n**	CHD1–**2s**
Beamline	BM07	BM07	ID30A-3	BM07	ID30A-3
**Data Collection**					
Space group	$P2_1\,2\,2_1$	$P2_1\,2\,2_1$	$P2_1\,2\,2_1$	$P2_1\,2\,2_1$	$P2_1\,2\,2_1$
a, b, c (Å)	44.73, 46.10, 110.01	45.15, 46.22, 110.03	44.80, 46.09, 110.26	44.96, 46.24, 110.17	44.95, 46.16, 110.04
α, β, γ (deg)	90.00, 90.00, 90.00	90.00, 90.00, 90.00	90.00, 90.00, 90.00	90.00, 90.00, 90.00	90.00, 90.00, 90.00
Wavelength (Å)	0.9795	0.9795	0.9677	0.9795	0.9677
Resolution (Å)	55.01 – 1.70 (1.73 – 1.70)	110.03 – 1.35 (1.37 – 1.35)	46.09 – 1.70 (1.73 – 1.70)	110.17 – 1.45 (1.47 – 1.45)	55.02 – 1.55 (1.58 – 1.55)
Total/unique no. of reflections	331,177 (25,755)	663,031 (51,348)	344,118 (25,905)	540,232 (41,483)	455,097 (33,668)
$R_{\mathrm{merge}}{}^{a,b}$	0.051 (2.687)	0.033 (1.214)	0.060 (1.754)	0.030 (1.416)	0.064 (1.341)
$R_{\mathrm{meas}}{}^{a,c}$	0.053 (2.795)	0.034 (1.277)	0.063 (1.821)	0.031 (1.472)	0.067 (1.390)
$R_{\mathrm{pim}}{}^{a,d}$	0.015 (0.763)	0.009 (0.386)	0.017 (0.487)	0.012 (0.554)	0.018 (0.363)
Wilson *B*-Factors ($Å^2$)	32.7	21.2	30.2	25.3	23.9
$CC_{1/2}{}^{a,e}$	1.00 (0.638)	1.00 (0.747)	0.999 (0.701)	1.00 (0.753)	0.999 (0.800)
$I/\sigma(I)\,^{a}$	24.5 (1.1)	32.5 (1.9)	21.6 (1.6)	35.5 (1.9)	19.5 (2.0)
Redundancy[a]	12.9 (13.0)	12.9 (10.5)	13.3 (13.9)	13.0 (13.5)	13.5 (14.2)
Completeness (%)[a]	99.7 (100)	99.7 (96.8)	99.9 (100)	99.8 (99.9)	99.0 (100)
**Refinement**					
No. of reflections used in refinement/test set	25,691 (2,509)	51,260 (4,973)	25,857 (2,513)	41,404 (4,067)	33,624 (3,297)
$R_{\mathrm{work}}{}^{f}$	0.203 (0.330)	0.185 (0.272)	0.198 (0.342)	0.201 (0.282)	0.187 (0.264)
$R_{\mathrm{free}}{}^{g}$	0.221 (0.323)	0.208 (0.290)	0.212 (0.362)	0.208 (0.299)	0.217 (0.331)
Number of Atoms[h]	1617	1792	1629	1722	1720
protein	1430	1490	1444	1455	1461
ligands	38	56	50	55	64
solvent	149	246	135	212	195
Average *B*-Factors ($Å^2$)	51.1	33.5	44.2	40.4	37.0
protein	51.4	32.4	44.3	40.0	36.6
ligands	51.4	36.5	45.1	40.5	36.3
solvent	48.6	39.4	43.5	43.0	39.8
RMS Deviations					
bonds (Å)	0.0058	0.0054	0.0060	0.0054	0.0063
angles (deg)	0.73	0.81	0.79	0.78	0.82
Ramachandran plot (%)[i]					
favored	99.40	100	99.42	99.42	99.42
allowed	0.60	0	0.58	0.58	0.58
outliers	0	0	0	0	0
PDB accession code	9T9E	9T9F	9T9G	9T9H	9T9I

[a] Values in parentheses refer to the highest-resolution shell of the data.

[b] $R_{merge} = \sum|I_h - \langle I_h\rangle|/\sum\langle I_h\rangle$; I_h = intensity measure for reflection *h;* $\langle I_h\rangle$ = average intensity for reflection *h* calculated from replicate data.

[c] $R_{meas} = \sum(n/(n-1)^{1/2}\sum|I_h - \langle I_h\rangle|/\sum\langle I_h\rangle$

[d] $R_{pim} = \sum(1/(n-1)^{1/2}|I_h - \langle I_h\rangle|/\sum\langle I_h\rangle$; n = number of observations (redundancy).

[e] $CC_{1/2} = \sigma_\tau^2/(\sigma_\tau^2 + \sigma_\varepsilon^2)$, where σ_τ^2 is the true measurement error variance and σ_ε^2 is the independent measurement error variance.

[f] $R_{work} = \sum||F_o| - |F_c||/\sum|F_o|$ for reflections contained in the working set. $|F_o|$ and $|F_c|$ are the observed and calculated structure factor amplitudes, respectively.

[g] $R_{free} = \sum||F_o| - |F_c||/\sum|F_o|$ for reflections contained in the test set held aside during refinement.

[h] Per asymmetric unit.

[i] Assessed by MolProbity

Table S2: Selectivity screening of **2s** for different kinases.

**Target**	**2s**	**2s**
Gene Symbol	Activity (%) vs. Ctrl at 10 µM	Inhibition (%) at 10 µM
ABL1(E255K)-phosphorylated	84	16
ABL1(T315I)-phosphorylated	92	8
ABL1-nonphosphorylated	77	23
ABL1-phosphorylated	79	21
ACVR1B	97	3
ADCK3	100	0
AKT1	95	5
AKT2	92	8
ALK	97	3
AURKA	76	24
AURKB	64	36
AXL	83	17
BMPR2	70	30
BRAF	96	4
BRAF(V600E)	65	35
BTK	99	1
CDK11	89	11
CDK2	86	14
CDK3	93	7
CDK7	100	0
CDK9	95	5
CHEK1	81	19
CSF1R	85	15
CSNK1D	84	16
CSNK1G2	78	22
DCAMKL1	69	31
DYRK1B	100	0
EGFR	90	10
EGFR (L858R)	86	14
EPHA2	91	9
ERBB2	71	29
ERBB4	93	7
ERK1	81	19
FAK	99	1
FGFR2	97	3
FGFR3	95	5
FLT3	86	14
GSK3B	80	20
IGF1R	94	6
IKK-alpha	80	20
IKK-beta	91	9
INSR	66	34
JAK2 (JH1domain-catalytic)	96	4
JAK3 (JH1domain-catalytic)	100	0
JNK1	67	33
JNK2	100	0
JNK3	71	29
KIT	90	10
KIT(D816V)	100	0
KIT(V559D,T670I)	87	13
LKB1	86	14
MAP3K4	81	19
MAPKAPK2	91	9
MARK3	84	16
MEK1	86	14
MEK2	93	7
MET	94	6
MKNK1	100	0
MKNK2	71	29
MLK1	95	5
p38-alpha	93	7
p38-beta	95	5
PAK1	96	4
PAK2	89	11
PAK4	92	8
PCTK1	84	16
PDGFRA	80	20
PDGFRB	91	9
PDPK1	98	2
PIK3C2B	50	50
PIK3CA	94	6
PIK3CG	72	28
PIM1	92	8
PIM2	91	9
PIM3	93	7
PKAC-alpha	94	6
PLK1	79	21
PLK3	92	8
PLK4	99	1
PRKCE	99	1
RAF1	85	15
RET	97	3
RIOK2	32	68
ROCK2	82	18
RSK2 (Kin.Dom.1-N-terminal)	94	6
SNARK	73	27
SRC	99	1
SRPK3	100	0
TGFBR1	77	23
TIE2	99	1
TRKA	100	0
TSSK1B	75	25
TYK2 (JH1domain-catalytic)	78	22
ULK2	80	20
VEGFR2	80	20
YANK3	84	16
ZAP70	100	0

Table S3: Peptides used in this study were synthesized by PSL Peptide Specialty Laboratories GmbH (Heidelberg, Germany). Peptides were delivered as TFA salts and dissolved in deionized water. For fluorescent probes, fluorophores were attached via their carboxyl function forming an amide bond with K14 (H3) or V118 (LSD1) side chain.

Name	Sequence	Used as
**$H3_{1-12}$K4me3**	H_2N-ARTKme3QTARKSTG-COOH	Reference inhibitor
**$H3_{1-12}$K4me2**	H_2N-ARTKme2QTARKSTG-COOH	Reference inhibitor
**$H3_{1-12}$K4me1**	H_2N-ARTKme1QTARKSTG-COOH	Reference inhibitor
**$H3_{1-12}$K4A**	H_2N-ARTAQTARKSTG-COOH	Negative control
**$H3_{1-21}$K4A**	H_2N-ARTAQTARKSTGGKAPRKQLA-COOH	Negative control
**FL-H3K4me3**	H_2N-ARTKme3QTARKSTGGK(-Fluorescein)-COOH	Fluorescent probe
**TAMRA-$H3_{1-14}$K4me3**	H_2N-ARTKme3QTARKSTGGK(-TAMRA)-COOH	Fluorescent probe
**Cy5-H3K4me3**	H_2N-ARTKme3QTARKSTGGK(-Cy5)-COOH	Fluorescent probe
**$LSD1_{104-120}$K114me3**	H_2N-TPEGRRTSRRKme3RAKVEY-COOH	Reference inhibitor
**$LSD1_{104-120}$K114me2**	H_2N-TPEGRRTSRRKme2RAKVEY-COOH	Reference inhibitor
**$LSD1_{104-120}$K114me1**	H_2N-TPEGRRTSRRKme1RAKVEY-COOH	Reference inhibitor
**$LSD1_{104-120}$**	H_2N-TPEGRRTSRRKRAKVEY-COOH	Negative control
**TAMRA-LSD1K114me2**	H_2N-TPEGRRTSRRKme2RAKV-COOH	Fluorescent probe

Table S4: Compound SMILES

Compound	SMILE
**1a**	COC1=CC2=C(C=C1OC)C(NC3CCN(CC4=CC=CC=C4)CC3)=NC(NCCCN(C)C)=N2
**1b**	COC1=CC2=C(C=C1OC)C(N(C)C)=NC(NCCCN(C)C)=N2
**1c**	CN(C)CCCNC1=NC2=C(C=CC=C2)C(NC3CCN(CC4=CC=CC=C4)CC3)=N1
**1d**	ClC1=NC2=C(C=CC=C2)C(NC3CCN(CC4=CC=CC=C4)CC3)=N1
**1e**	COC(C=C1)=CC2=C1N=C(NCCCN(C)C)N=C2NC3CCN(CC4=CC=CC=C4)CC3
**1f**	COC1=CC2=C(C=C1)C(NC3CCN(CC4=CC=CC=C4)CC3)=NC(NCCCN(C)C)=N2
**1g**	ClC1=CC2=C(C=C1)C(NC3CCN(CC4=CC=CC=C4)CC3)=NC(NCCCN(C)C)=N2
**1h**	COC1=CC2=C(C=C1)C(NC3=CC=C(Cl)C=C3)=NC(NCCCN(C)C)=N2
**1i**	COC1=CC2=C(C=C1)C(NC3CCN(CC4=CC=CC=C4)CCC3)=NC(NCCCN(C)C)=N2
**1j**	COC1=CC2=C(C=C1)C(NC3CCN(CC4=CNC=C4)CC3)=NC(NCCCN(C)C)=N2
**1k**	COC1=CC2=C(C=C1)C(NC3CCN(CC4=CC(C=CC=C5)=C5C=C4)CC3)=NC(NCCCN(C)C)=N2
**1l**	COC1=CC2=C(C=C1)C(NC3CCN(CC4=CC=CN=C4)CC3)=NC(NCCCN(C)C)=N2
**1m**	COC1=CC2=C(C=C1)C(NC3CCN(CC4=CC=CC(C)=C4)CC3)=NC(NCCCN(C)C)=N2
**1n**	COC1=CC2=C(C=C1)C(NC3CCN(CC4=CC=CC(Cl)=C4)CC3)=NC(NCCCN(C)C)=N2
**2a**	COC1=CC2=C(C=C1)C(NC3CCN(CC4=CC=CC=C4)CC3)=CC(NCCCN(C)C)=N2
**2b**	CN(C)CCCNC1=NC2=C(C=CC(OCC)=C2)C(NC3CCN(CC4=CC=CC=C4)CC3)=C1
**2c**	CN(C)CCCNC1=NC2=C(C=CC(OC(C)C)=C2)C(NC3CCN(CC4=CC=CC=C4)CC3)=C1
**2d**	CN(C)CCCNC1=NC2=C(C=CC(OC3CCC3)=C2)C(NC4CCN(CC5=CC=CC=C5)CC4)=C1
**2e**	CN(C)CCCNC1=NC2=C(C=CC(OC3CCCC3)=C2)C(NC4CCN(CC5=CC=CC=C5)CC4)=C1
**2f**	CN(C)CCCNC1=NC2=C(C=CC(OC3CCCCC3)=C2)C(NC4CCN(CC5=CC=CC=C5)CC4)=C1
**2g**	CN(C)CCCNC1=NC2=C(C=CC(OC3=CC=CC=C3)=C2)C(NC4CCN(CC5=CC=CC=C5)CC4)=C1
**2h**	CN(C)CCCNC1=NC2=C(C=CC(OCC3=CC=CC=C3)=C2)C(NC4CCN(CC5=CC=CC=C5)CC4)=C1
**1o**	CN(C)CCCNC1=NC2=C(C=CC(OCC)=C2)C(NC3CCN(CC4=CC=CC=C4)CC3)=N1
**2i**	CCOC1=CC2=C(C=C1)C(NC3CCN(CC4=CC=CC=C4)CC3)=CC(NC5CCN(C)CC5)=N2
**2j**	CCOC1=CC2=C(C=C1)C(NC3CCN(CC4=CC=CC=C4)CC3)=CC(N5C=NC=C5)=N2
**2k**	CCOC1=CC2=C(C=C1)C(NC3CCN(CC4=CC=CC=C4)CC3)=CC(N5CCC(NC)CC5)=N2
**1p**	CCOC1=CC2=C(C=C1)C(NC3CCN(CC4=CC=CC=C4)CC3)=NC(N5CCC(NC)CC5)=N2
**2l**	CCOC1=CC2=C(C=C1)C(NC3CCN(CC4=CC=CC=C4)CC3)=CC(N5CCC(N(C)C)CC5)=N2
**1q**	CCOC1=CC2=C(C=C1)C(NC3CCN(CC4=CC=CC=C4)CC3)=NC(N5CCC(N(C)C)CC5)=N2
**2m**	CCOC1=CC2=C(C=C1)C(NC3CCN(CC4=CC=CC=C4)CC3)=CC(N5CCC(N6CCCC6)CC5)=N2
**1r**	CCOC1=CC2=C(C=C1)C(NC3CCN(CC4=CC=CC=C4)CC3)=NC(N5CCC(N6CCCC6)CC5)=N2
**2n**	CCOC1=CC2=C(C=C1)C(NC3CCN(CC4=CC=CC=C4)CC3)=CC(N5CCC(NCC6=CC=C(OC)C=C6)CC5)=N2
**2o**	CCOC1=CC2=C(C=C1)C(NC3CCN(CC4=CC=CC=C4)CC3)=CC(N5CCC(N(C)CC6=CC=C(OC)C=C6)CC5)=N2
**1s**	CCOC1=CC2=C(C=C1)C(NC3CCN(CC4=CC=CC=C4)CC3)=NC(N5CCC(NCC6=CC=C(OC)C=C6)CC5)=N2
**1t**	CCOC1=CC2=C(C=C1)C(NC3CCN(CC4=CC=CC=C4)CC3)=NC(N5CCC(N(C)CC6=CC=C(OC)C=C6)CC5)=N2
**2p**	CCOC1=CC2=C(C=C1)C(NC3CCN(CC4=CC=CC=C4)CC3)=CC(N5CCC(NCC6=CC=C([N+]([O-])=O)C=C6)CC5)=N2
**2q**	CCOC1=CC2=C(C=C1)C(NC3CCN(CC4=CC=CC=C4)CC3)=CC(N5CCC(NCC6=CC=C(C)C=C6)CC5)=N2
**2r**	CCOC1=CC2=C(C=C1)C(NC3CCN(CC4=CC=CC=C4)CC3)=CC(N5CCC(NCC6=CC=C(OCC#C)C=C6)CC5)=N2
**2s**	CCOC1=CC2=C(C=C1)C(NC3CCN(CC4=CC=CC=C4)CC3)=CC(N5CCC(NCC6=CC=C(OCC7=CN(CCOC)N=N7)C=C6)CC5)=N2
**2s-TAMRA**	O=C(C1=CC=C(C(OC23C(C=CC(N(C)C)=C4)=C4OC5=C3C=CC(N(C)C)=C5)=O)C2=C1)NCCOCCOCCCOCCN6N=NC(COC(C=C7)=CC=C7CNC(CC8)CCN8C9=NC%10=CC(OCC)=CC=C%10C(NC%11CCN(CC%12=CC=CC=C%12)CC%11)=C9)=C6

Supplementary References

(1) Menna, M.; Fiorentino, F.; Marrocco, B.; Lucidi, A.; Tomassi, S.; Cilli, D.; Romanenghi, M.; Cassandri, M.; Pomella, S.; Pezzella, M.; et al. Novel non-covalent LSD1 inhibitors endowed with anticancer effects in leukemia and solid tumor cellular models. *European Journal of Medicinal Chemistry* **2022**, *237*, 114410. DOI: 10.1016/j.ejmech.2022.114410.

(2) Ma, A.; Yu, W.; Li, F.; Bleich, R. M.; Herold, J. M.; Butler, K. V.; Norris, J. L.; Korboukh, V.; Tripathy, A.; Janzen, W. P.; et al. Discovery of a Selective, Substrate-Competitive Inhibitor of the Lysine Methyltransferase SETD8. *Journal of Medicinal Chemistry* **2014**, *57* (15), 6822-6833. DOI: 10.1021/jm500871s.

(3) Kubicek, S.; O'Sullivan, R. J.; August, E. M.; Hickey, E. R.; Zhang, Q.; Teodoro, Miguel L.; Rea, S.; Mechtler, K.; Kowalski, J. A.; Homon, C. A.; et al. Reversal of H3K9me2 by a Small-Molecule Inhibitor for the G9a Histone Methyltransferase. *Molecular Cell* **2007**, *25* (3), 473-481. DOI: 10.1016/j.molcel.2007.01.017.

(4) Cho, A.; Clarke, M.; Kim, C.; Link, J.; OPyun, H.; Sheng, X.; Wu, Q. Antiviral Compounds. *WO2009005676A2* **2009**.

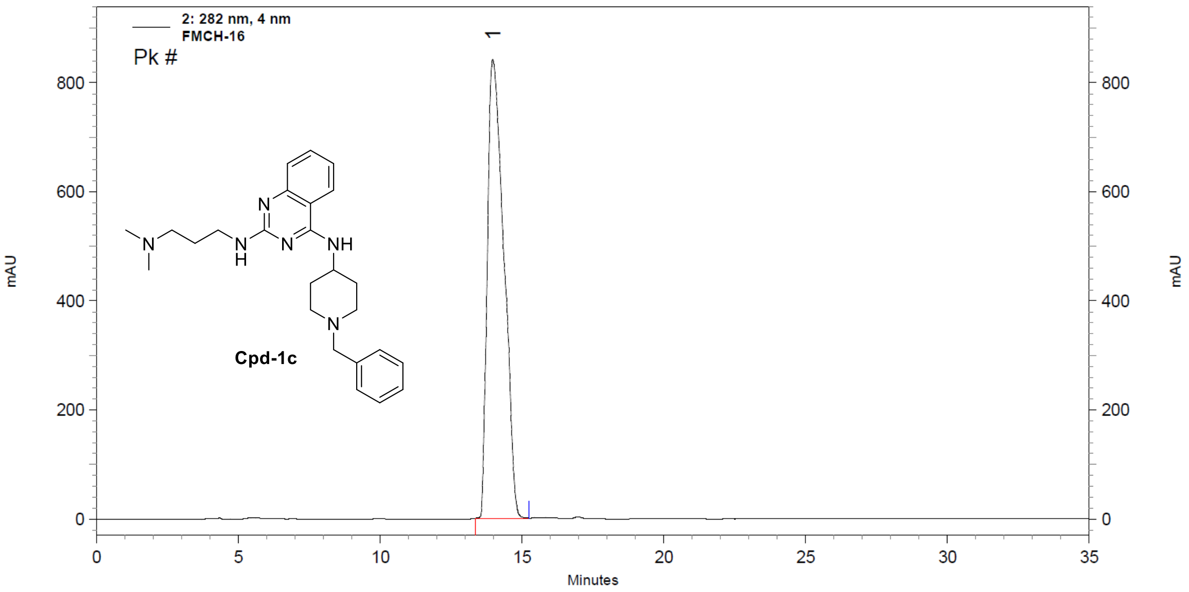

2: 282 nm, 4 nm Results

Pk #	Retention Time	Area Percent	Lambda Max
1	13,967	100,000	256

Figure S9: HPLC chromatogram of Compound **1c** (282 nm).

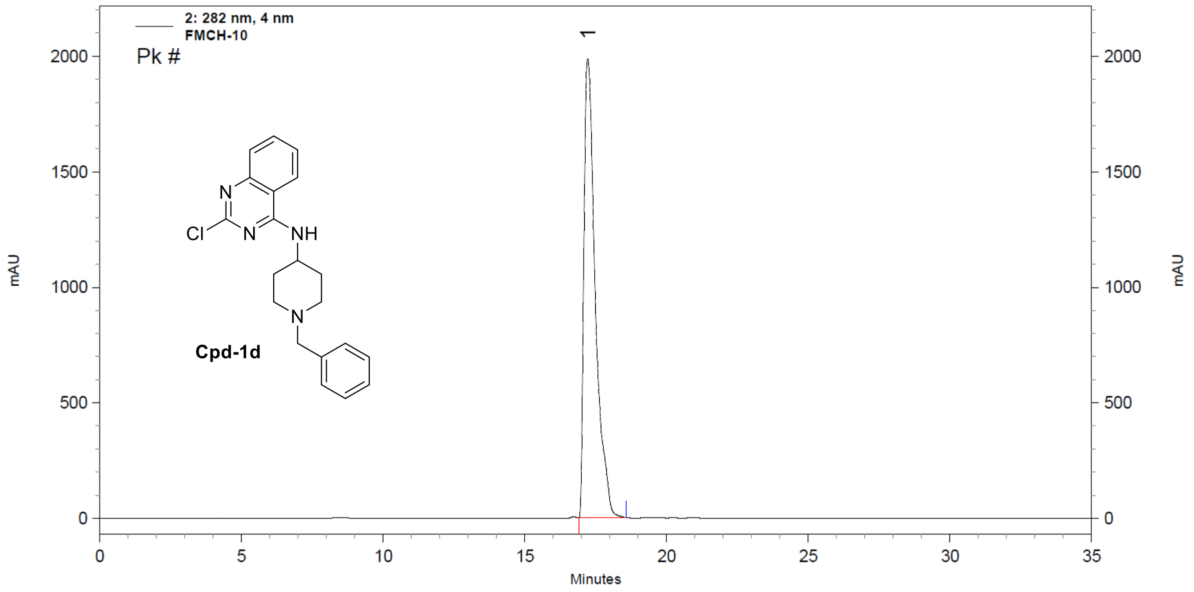

2: 282 nm, 4 nm Results

Pk #	Retention Time	Area Percent	Lambda Max
1	17,227	100,000	314

Figure S10: HPLC chromatogram of Compound **1d** (282 nm).

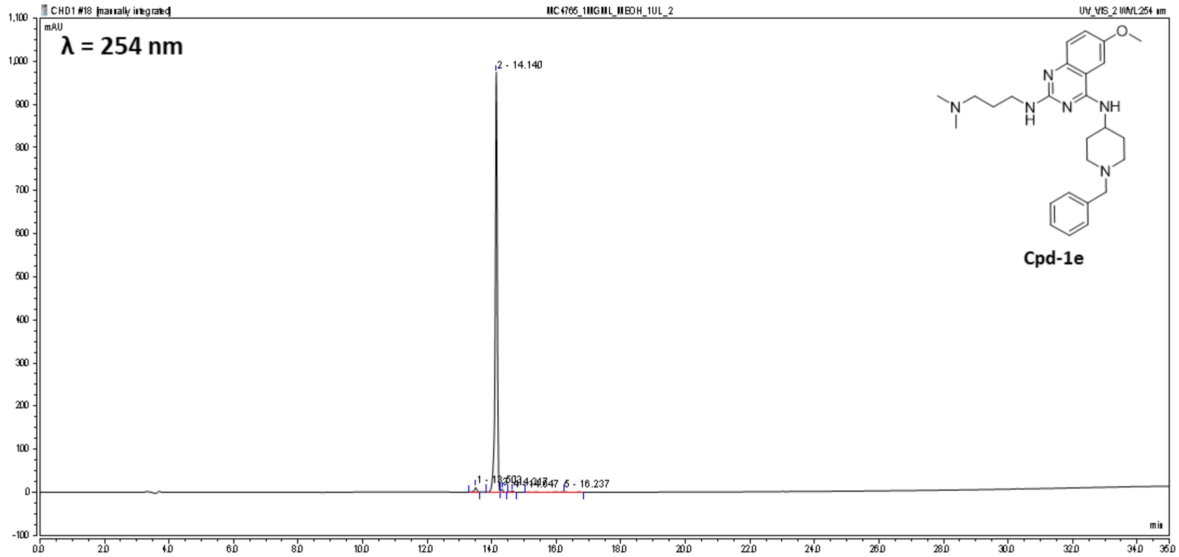

Peak n.	Ret. Time (min)	Rel. area (%)	Area (mAU * min)	Height (mAU)
1	13.503	1.22	0.8953	10.9
2	14.14	97.29	71.5972	974.19
3	14.317	0.67	0.4942	7.16
4	14.647	0.30	0.2238	2.57
5	16.237	0.52	0.3826	0.93

Figure S11: HPLC chromatogram of Compound **1e** (254 nm).

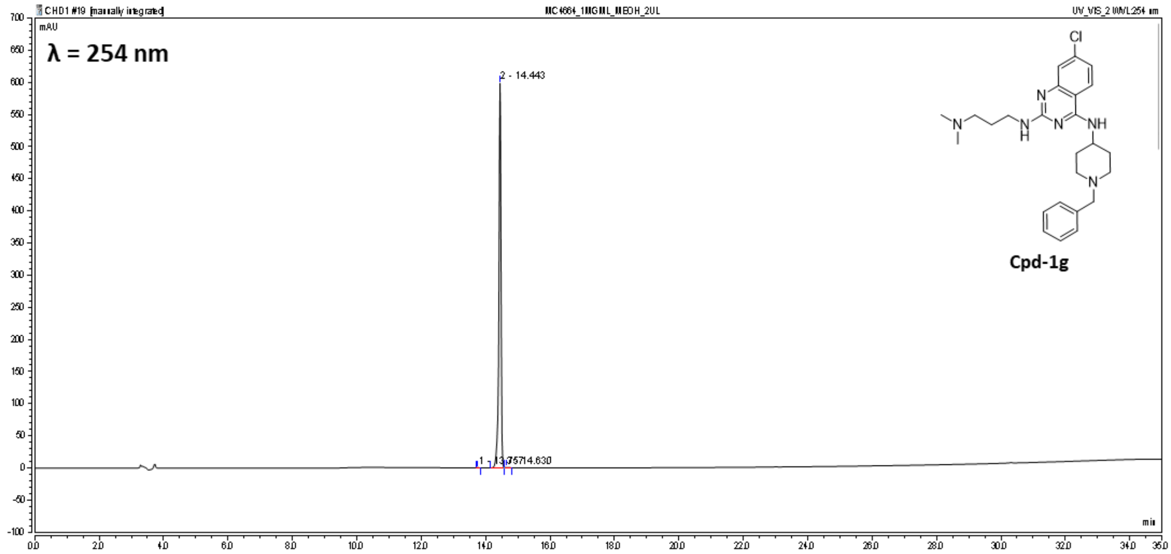

Peak n.	Ret. Time (min)	Rel. area (%)	Area (mAU * min)	Height (mAU)
1	13.757	0.05	0.0217	0.31
2	14.443	99.81	44.8589	598.28
3	14.63	0.14	0.0615	0.58

Figure S12: HPLC chromatogram of Compound **1g** (254 nm).

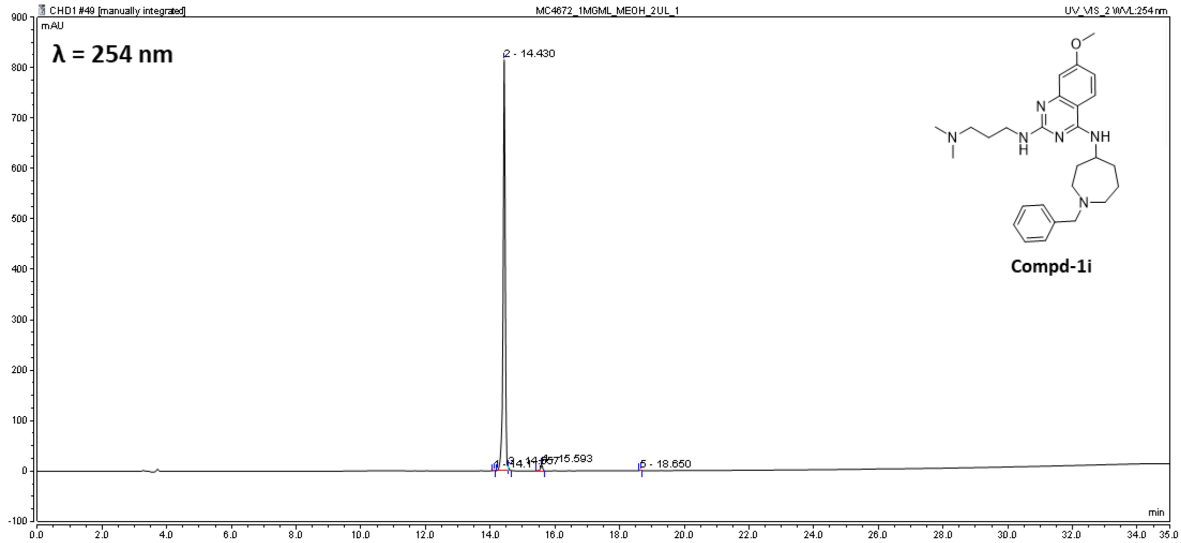

Peak n.	Ret. Time (min)	Rel. area (%)	Area (mAU * min)	Height (mAU)
1	14.12	0.08	0.05	1.03
2	14.43	97.84	61.72	814.70
3	14.56	0.63	0.40	7.62
4	15.59	1.37	0.86	11.81
5	18.65	0.07	0.04	0.72

Figure S13: HPLC chromatogram of Compound **1i** (254 nm).

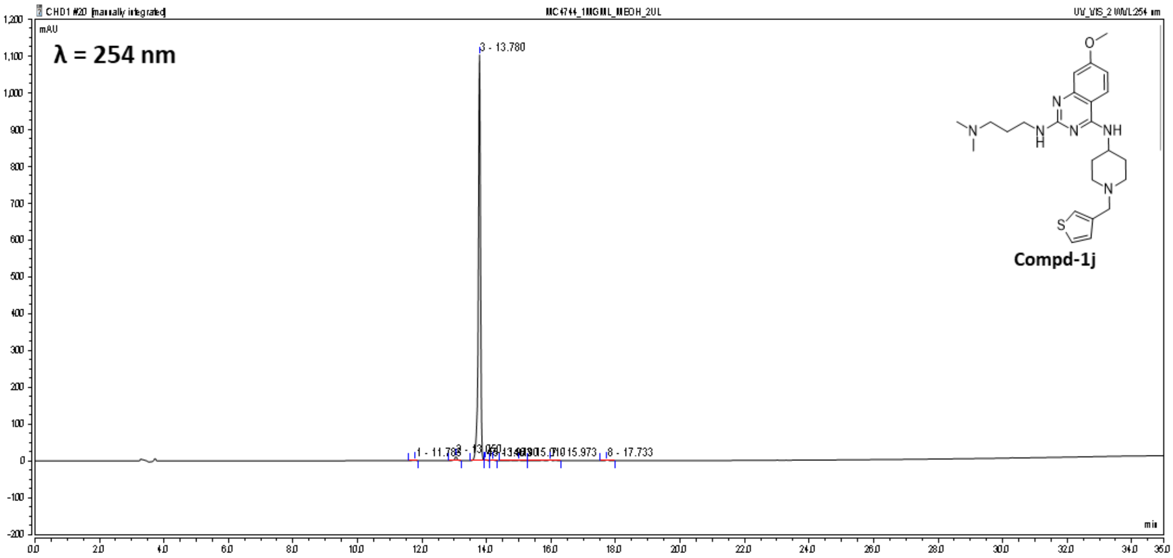

Peak n.	Ret. Time (min)	Rel. area (%)	Area (mAU * min)	Height (mAU)
1	11.783	0.12	0.1054	1.09
2	13.05	1.11	0.9699	11.26
3	13.78	96.95	84.5202	1102.21
4	13.973	0.28	0.2455	2.76
5	14.19	0.33	0.2882	2.54
6	15.01	0.31	0.2736	1.13
7	15.973	0.63	0.5516	2.98
8	17.733	0.26	0.228	2.31

Figure S14: HPLC chromatogram of Compound **1j** (254 nm).

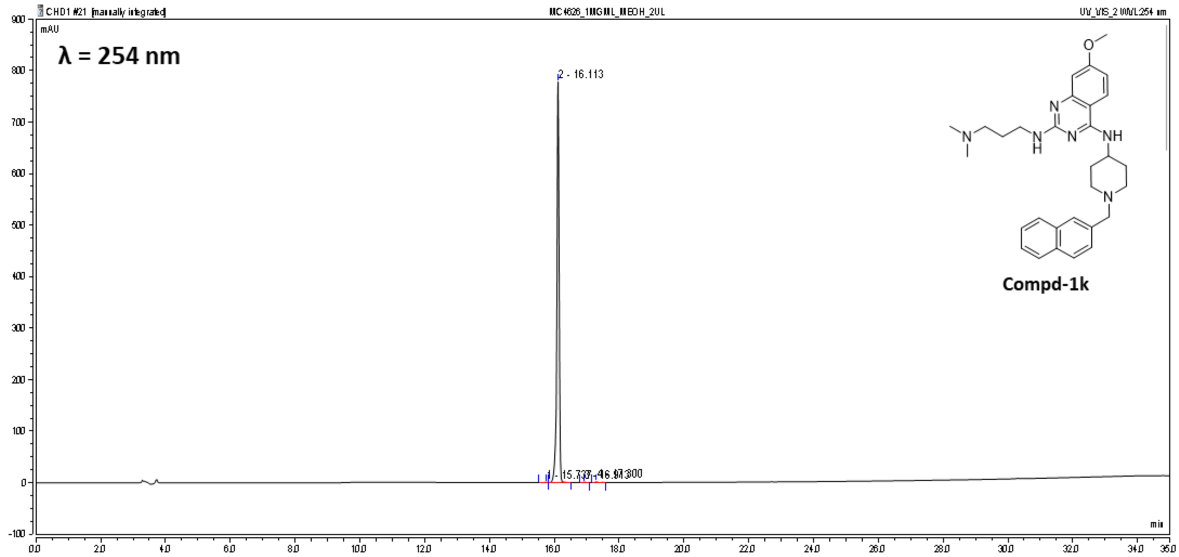

Peak n.	Ret. Time (min)	Rel. area (%)	Area (mAU * min)	Height (mAU)
1	15.737	0.09	0.0552	0.62
2	16.113	99.62	61.6471	777.79
3	16.913	0.08	0.0508	0.44
4	17.3	0.21	0.1276	1.18

Figure S15: HPLC chromatogram of Compound **1k** (254 nm).

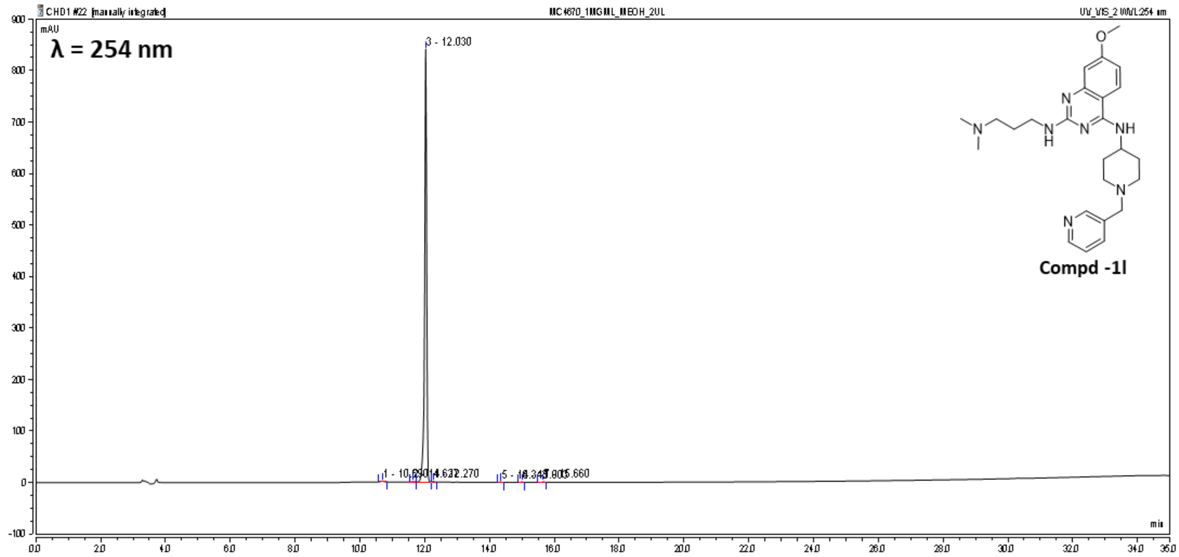

Peak n.	Ret. Time (min)	Rel. area (%)	Area (mAU * min)	Height (mAU)
1.00	10.69	0.30	0.19	1.82
2.00	11.63	0.10	0.06	0.63
3.00	12.03	99.12	61.71	841.14
4.00	12.27	0.14	0.09	1.14
5.00	14.34	0.10	0.06	0.97
6.00	15.00	0.00	0.06	0.94
7.00	15.66	0.13	0.08	1.16

Figure S16: HPLC chromatogram of Compound **1l** (254 nm).

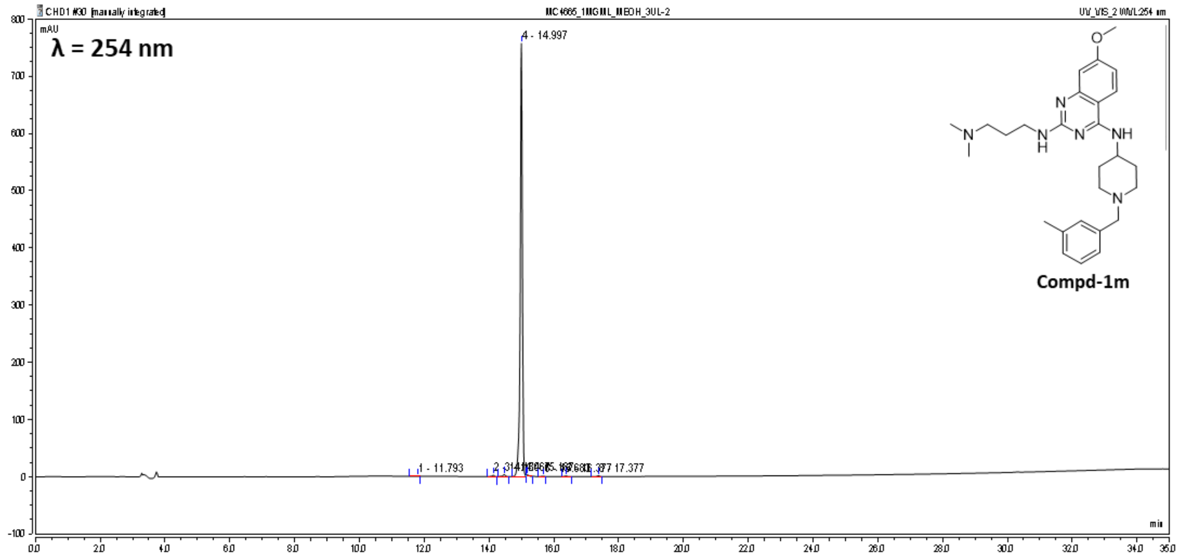

Peak n.	Ret. Time (min)	Rel. area (%)	Area (mAU * min)	Height (mAU)
1.00	11.79	0.04	0.02	0.21
2.00	14.13	0.30	0.18	2.50
3.00	14.47	0.48	0.29	3.57
4.00	15.00	98.32	58.47	757.18
5.00	15.19	0.44	0.26	3.07
6.00	15.68	0.09	0.05	0.52
7.00	16.38	0.15	0.09	1.17
8.00	17.38	0.17	0.10	1.35

Figure S17: HPLC chromatogram of Compound **1m** (254 nm).

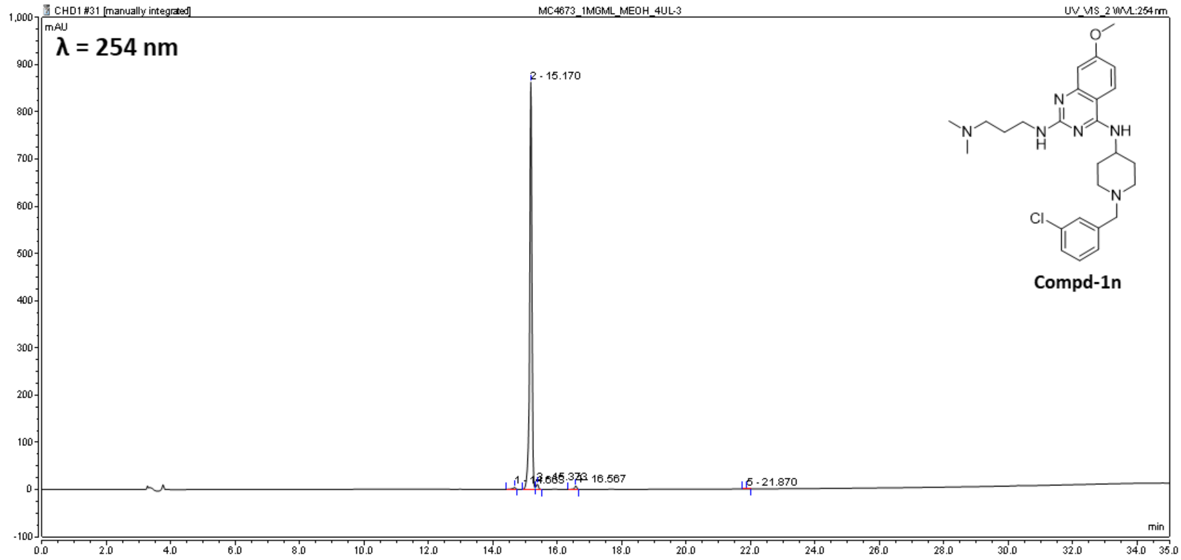

Peak n.	Ret. Time (min)	Rel. area (%)	Area (mAU * min)	Height (mAU)
1	14.66	0.59	0.42	4.50
2	15.17	97.19	69.02	862.62
3	15.37	1.30	0.92	11.40
4	16.57	0.86	0.61	7.57
5	21.87	0.06	0.05	0.51

Figure S18: HPLC chromatogram of Compound **1n** (254 nm).

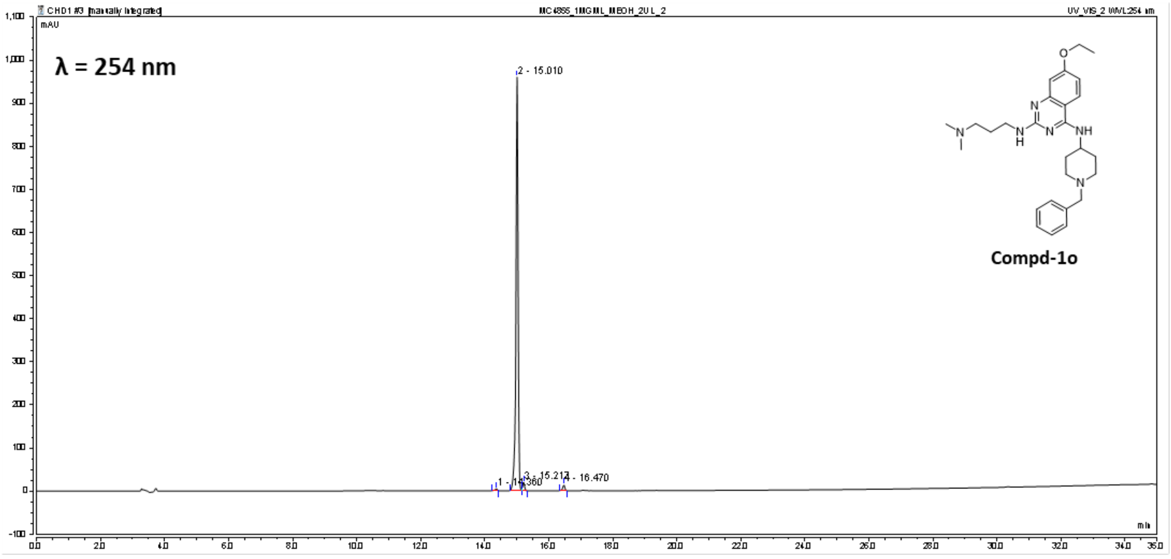

Peak n.	Ret. Time (min)	Rel. area (%)	Area (mAU * min)	Height (mAU)
1	14.36	0.45	0.3501	4.83
2	15.01	96.58	75.0866	959.66
3	15.217	1.69	1.3109	19.22
4	16.47	1.28	0.9974	13.33

Figure S19: HPLC chromatogram of Compound **1o** (254 nm).

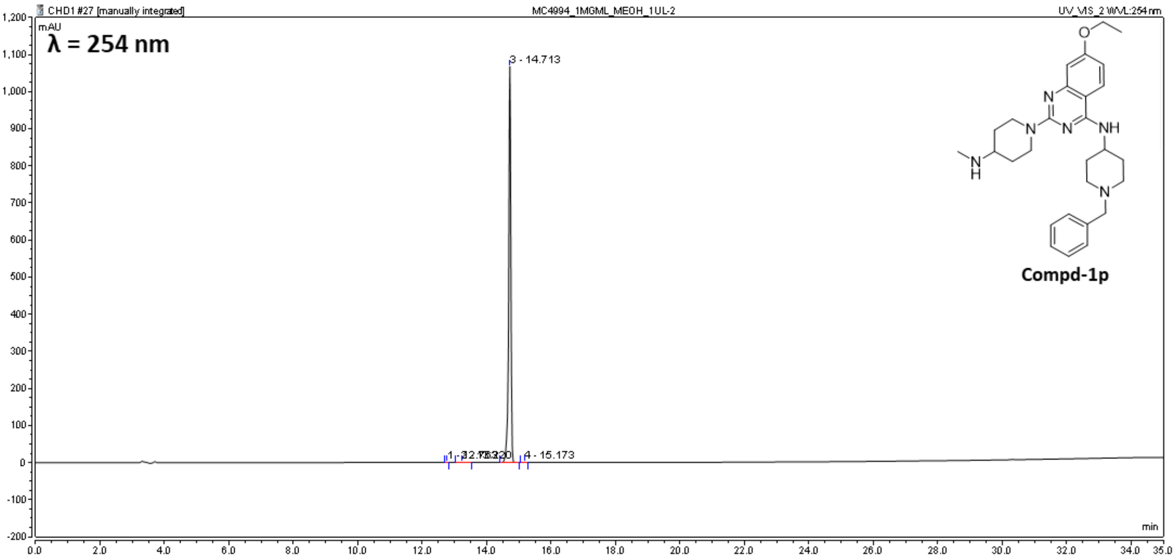

Peak n.	Ret. Time (min)	Rel. area (%)	Area (mAU * min)	Height (mAU)
1	12.76	0.02	0.02	0.31
2	13.22	0.06	0.05	0.43
3	14.71	99.87	81.90	1067.88
4	15.17	0.05	0.04	0.68

Figure S20: HPLC chromatogram of Compound **1p** (254 nm).

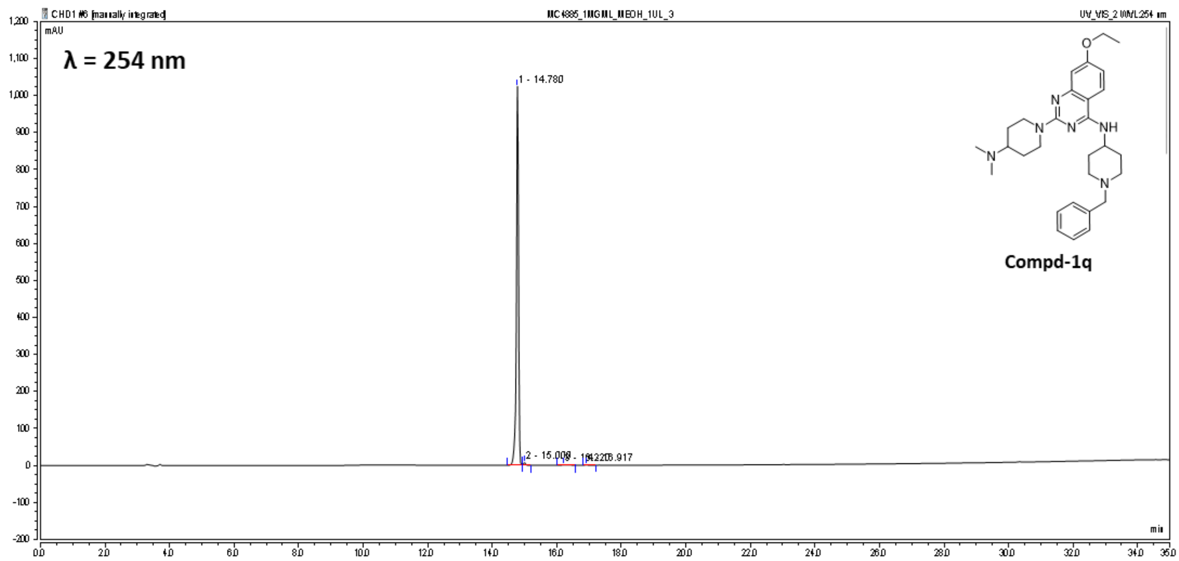

Peak n.	Ret. Time (min)	Rel. area (%)	Area (mAU * min)	Height (mAU)
1	14.78	98.99	77.7548	1024.45
2	15	0.65	0.5091	7
3	16.22	0.29	0.2244	1
4	16.917	0.07	0.0576	1.03

Figure S21: HPLC chromatogram of Compound **1q** (254 nm).

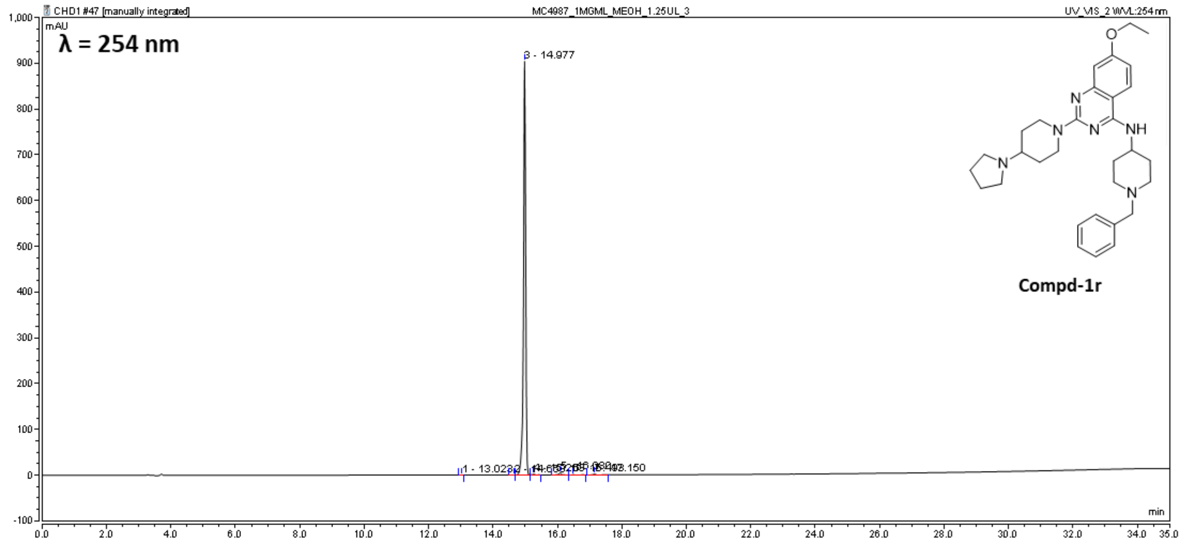

Peak n.	Ret. Time (min)	Rel. area (%)	Area (mAU * min)	Height (mAU)
1	13.02	0.01	0.01	0.16
2	14.66	0.03	0.02	0.23
3	14.98	98.77	69.90	903.80
4	15.26	0.22	0.15	1.69
5	16.08	0.63	0.45	5.03
6	16.49	0.08	0.06	0.41
7	17.15	0.25	0.18	2.06

Figure S22: HPLC chromatogram of Compound **1r** (254 nm).

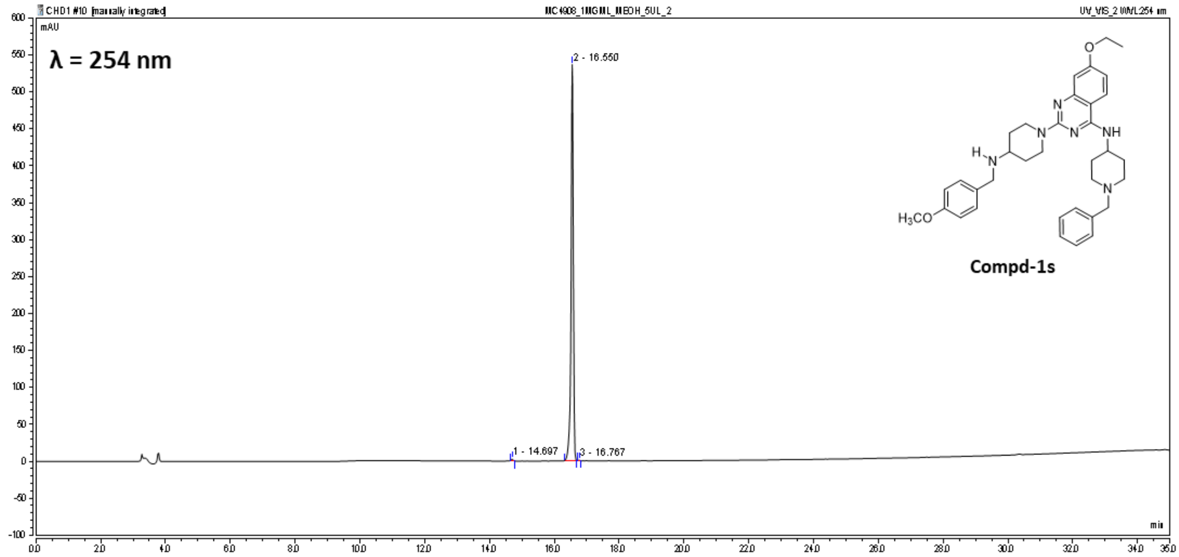

Peak n.	Ret. Time (min)	Rel. area (%)	Area (mAU * min)	Height (mAU)
1	14.697	0.34	0.1482	2.4
2	16.55	99.58	43.1051	536.01
3	16.767	0.08	0.0338	0.6

Figure S23: HPLC chromatogram of Compound **1s** (254 nm).

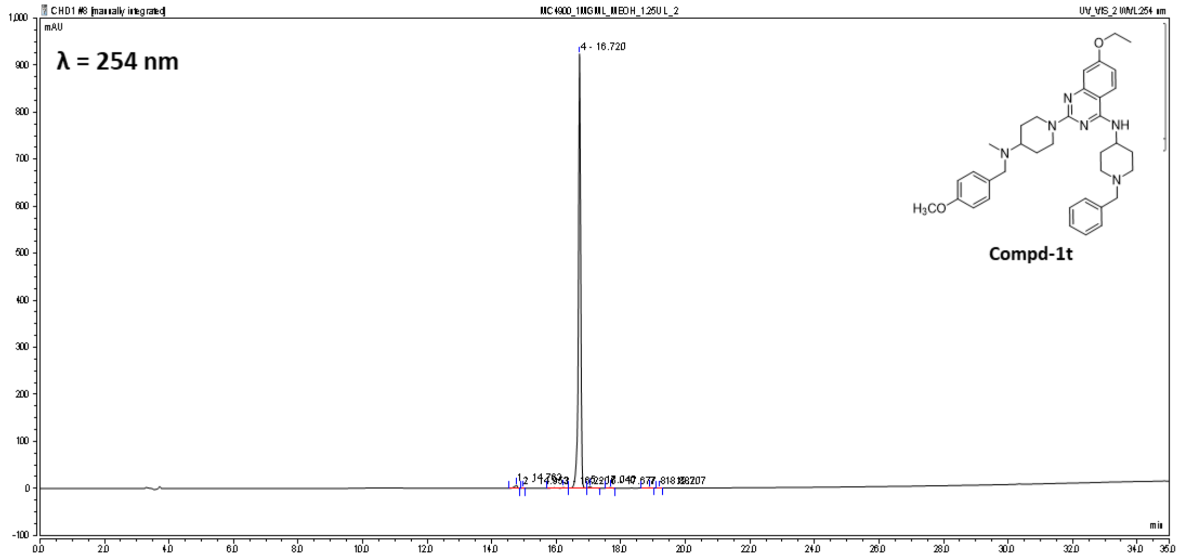

Peak n.	Ret. Time (min)	Rel. area (%)	Area (mAU * min)	Height (mAU)
1	14.763	0.90	0.7018	6.81
2	14.953	0.04	0.0327	0.56
3	16.22	0.49	0.3812	1.45
4	16.72	97.73	75.8403	923.22
5	17.04	0.49	0.3769	3.63
6	17.677	0.19	0.1472	1.22
7	18.887	0.12	0.0954	1.1
8	19.207	0.04	0.0285	0.36

Figure S24: HPLC chromatogram of Compound **1t** (254 nm).

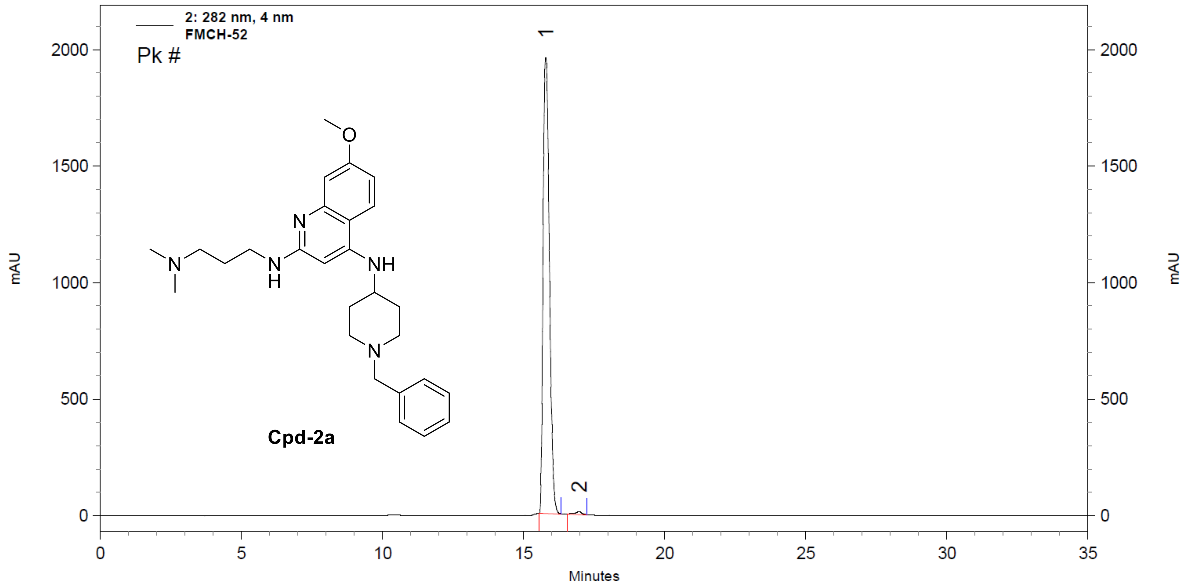

2: 282 nm, 4 nm Results

Pk #	Retention Time	Area Percent	Lambda Max
1	15,787	99,333	317
2	16,967	0,667	239

Figure S25: HPLC chromatogram of Compound **2a** (282 nm).

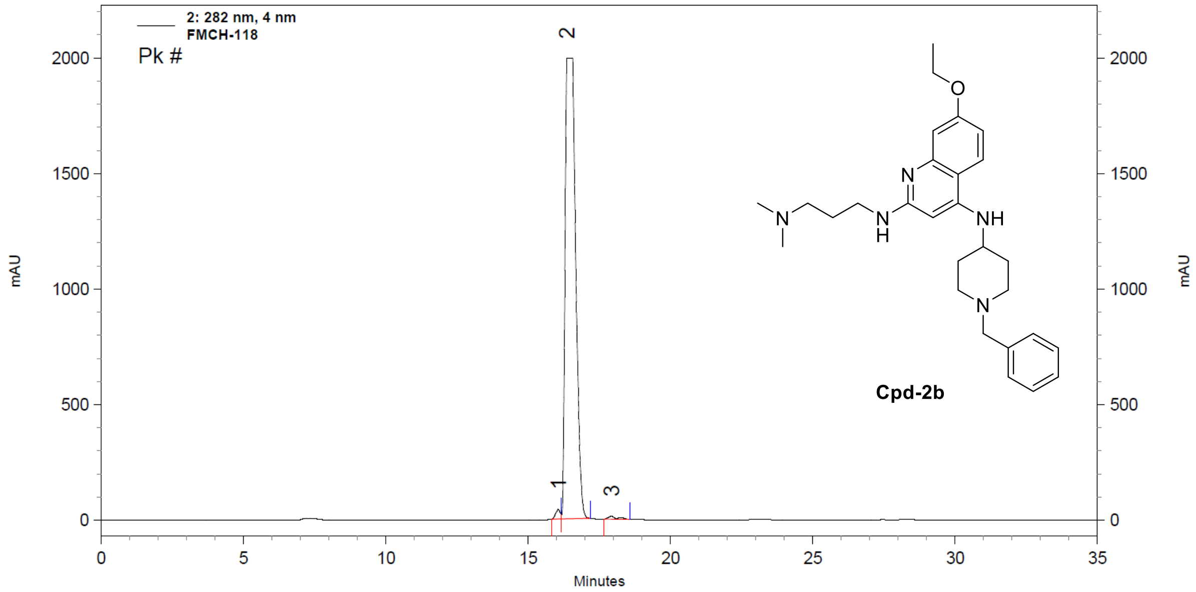

2: 282 nm, 4 nm Results

Pk #	Retention Time	Area Percent	Lambda Max
1	16,060	0,936	237
2	16,360	98,477	319
3	17,927	0,587	239

Figure S26: HPLC chromatogram of Compound **2b** (282 nm).

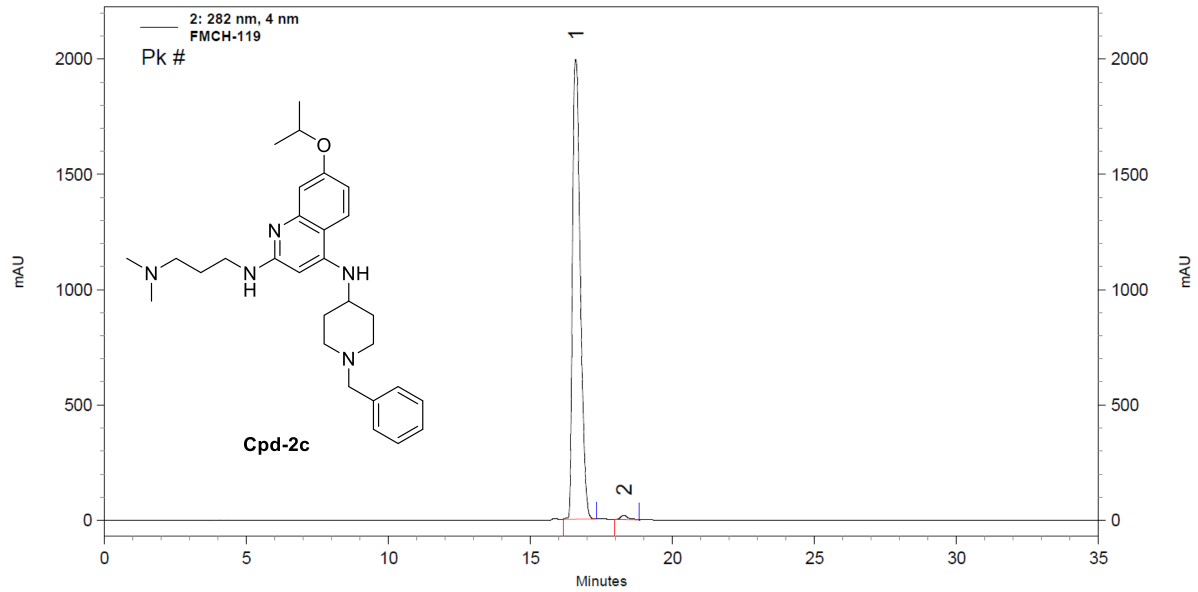

2: 282 nm, 4 nm Results

Pk #	Retention Time	Area Percent	Lambda Max
1	16,593	99,162	319
2	18,293	0,838	240

Figure S27: HPLC chromatogram of Compound **2c** (282 nm).

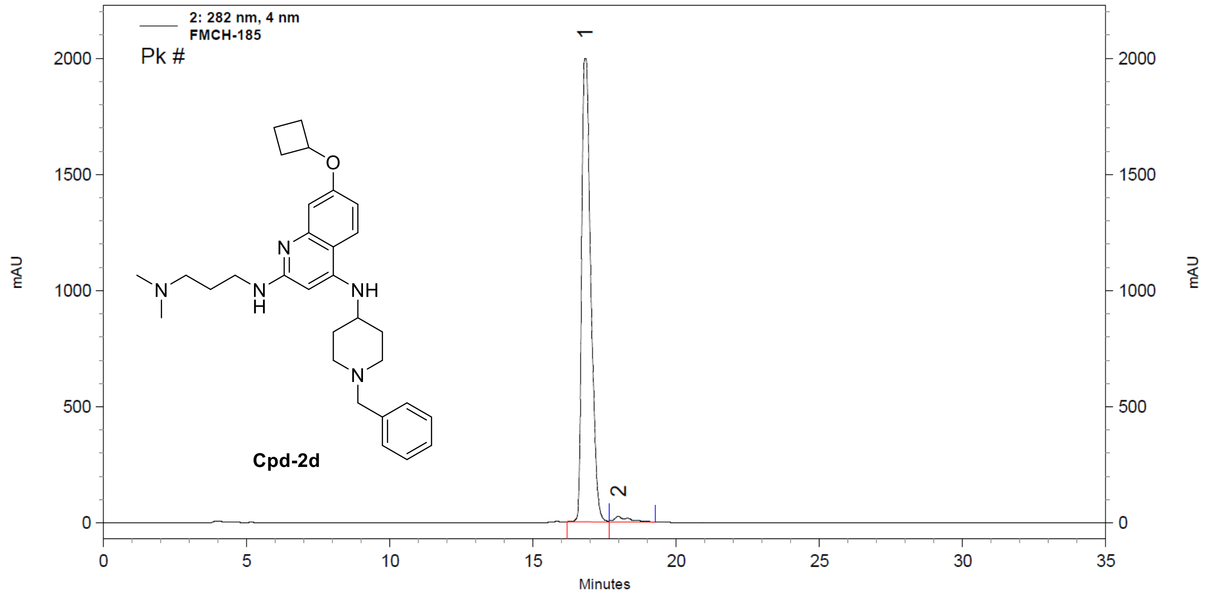

2: 282 nm, 4 nm Results

Pk #	Retention Time	Area Percent	Lambda Max
1	16,807	98,036	318
2	17,980	1,964	241

Figure S28: HPLC chromatogram of Compound **2d** (282 nm).

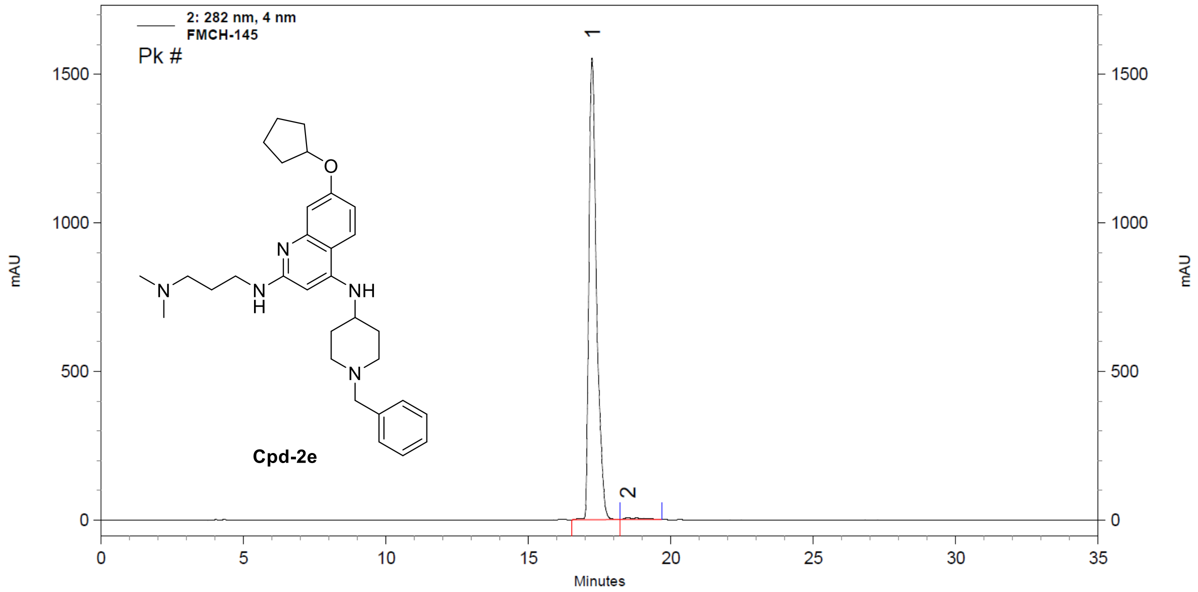

2: 282 nm, 4 nm Results

Pk #	Retention Time	Area Percent	Lambda Max
1	17,240	99,067	317
2	18,493	0,933	243

Figure S29: HPLC chromatogram of Compound **2e** (282 nm).

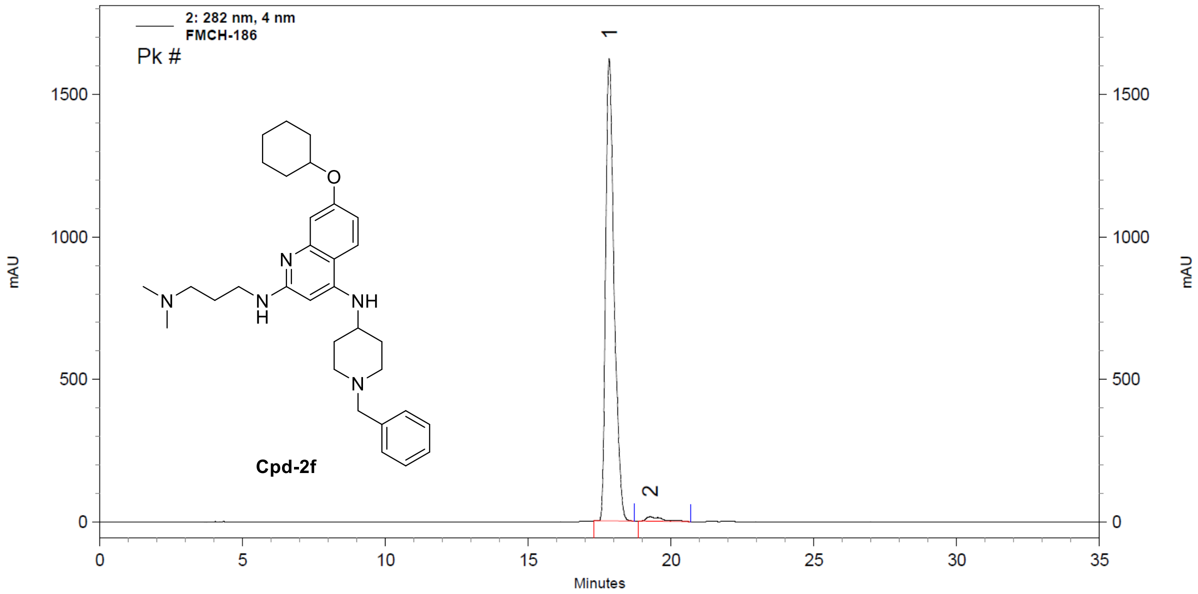

2: 282 nm, 4 nm Results

Pk #	Retention Time	Area Percent	Lambda Max
1	17,840	98,193	318
2	19,267	1,807	243

Figure S30: HPLC chromatogram of Compound **2f** (282 nm).

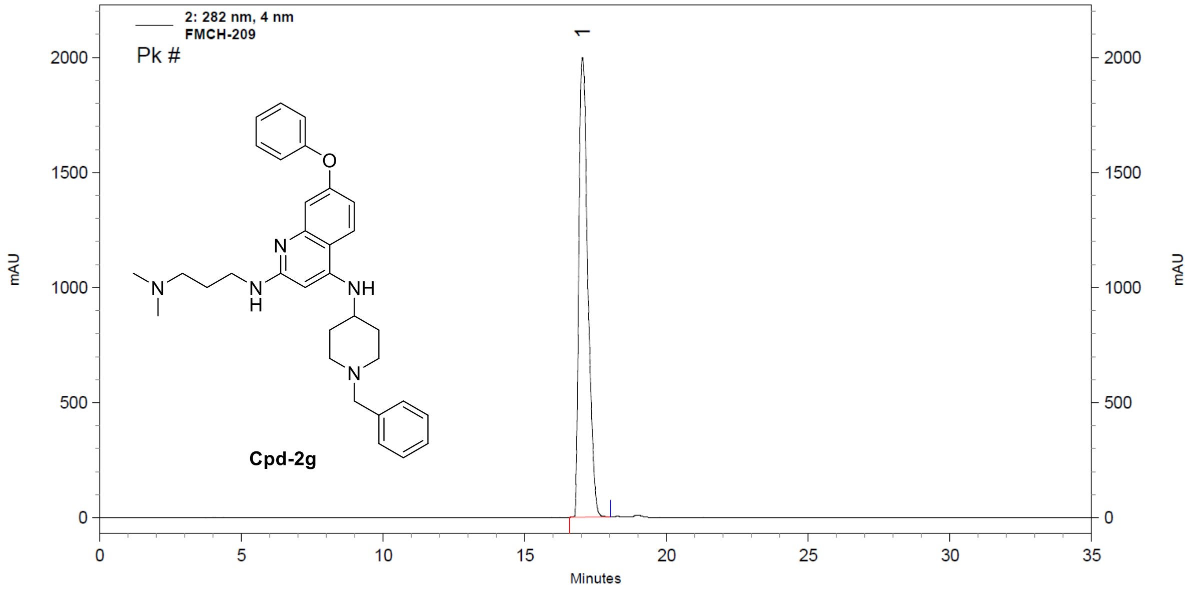

2: 282 nm, 4 nm Results			
Pk #	Retention Time	Area Percent	Lambda Max
1	17,027	100,000	318

Figure S31: HPLC chromatogram of Compound **2g** (282 nm).

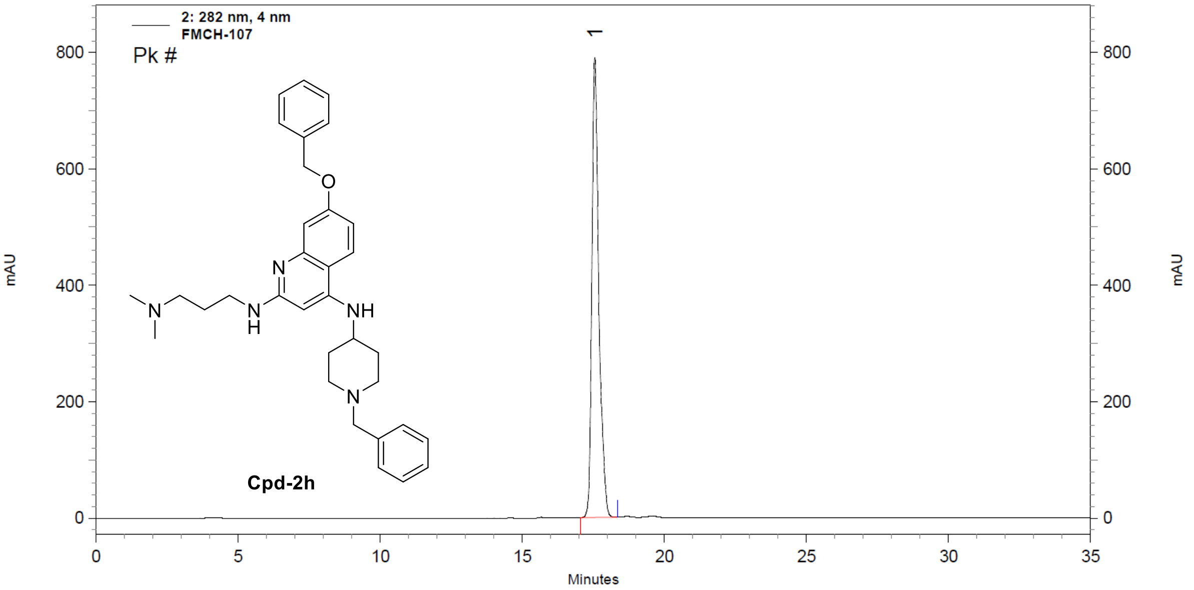

2: 282 nm, 4 nm Results			
Pk #	Retention Time	Area Percent	Lambda Max
1	17,553	100,000	253

Figure S32: HPLC chromatogram of Compound **2h** (282 nm).

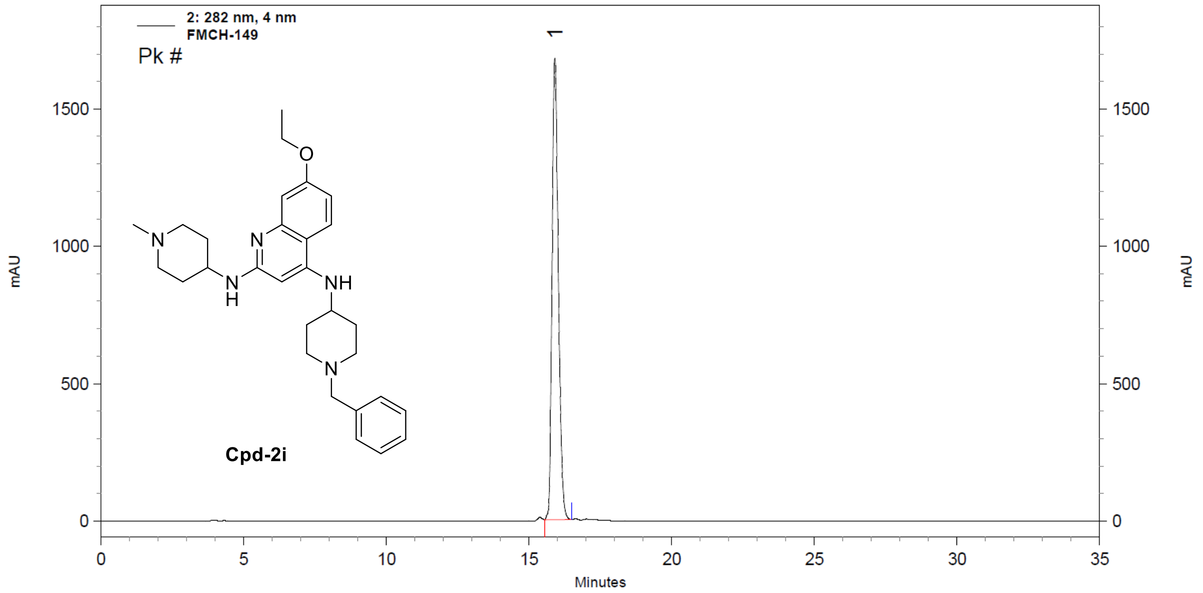

2: 282 nm, 4 nm Results

Pk #	Retention Time	Area Percent	Lambda Max
1	15,907	100,000	316

Figure S33: HPLC chromatogram of Compound **2i** (282 nm).

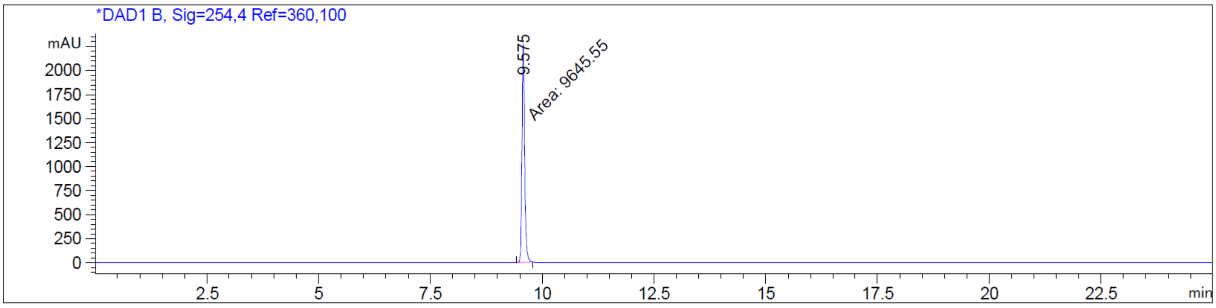

Signal 2: DAD1 B, Sig=254,4 Ref=360,100

Peak #	RetTime [min]	Type	Width [min]	Area [mAU*s]	Height [mAU]	Area %
1	9.575	MM	0.0704	9645.55078	2283.61694	100.0000
Totals :				9645.55078	2283.61694	

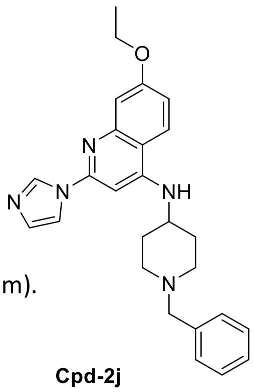

Cpd-2j

Figure S34: HPLC chromatogram of Compound **2j** (254 nm).

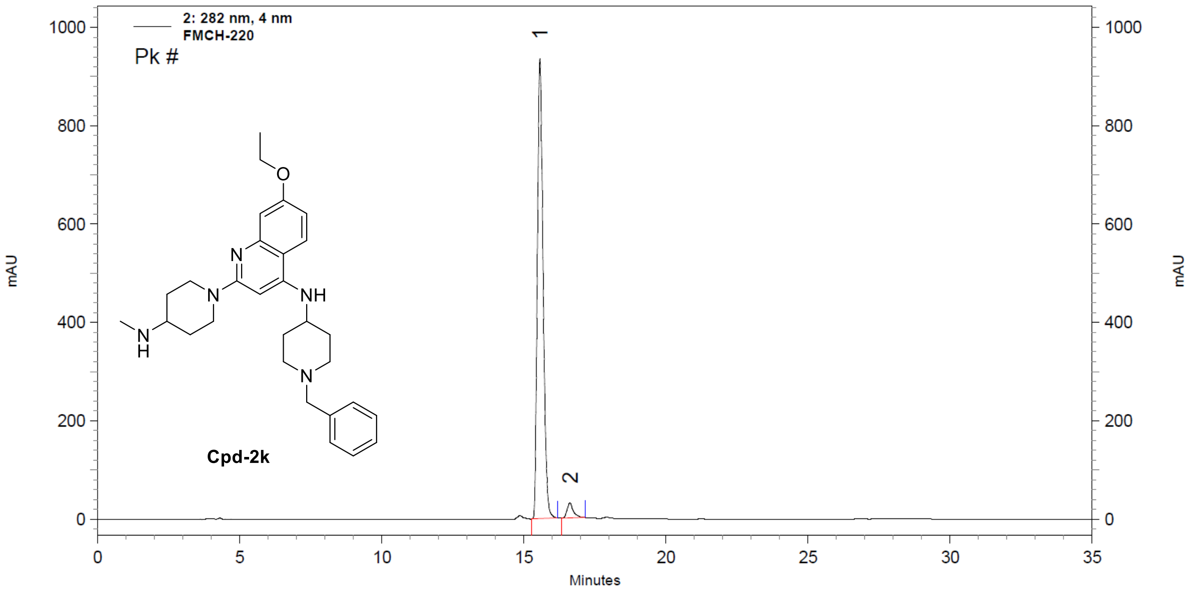

2: 282 nm, 4 nm Results

Pk #	Retention Time	Area Percent	Lambda Max
1	15,567	96,601	318
2	16,627	3,399	244

Figure S35: HPLC chromatogram of Compound **2k** (282 nm).

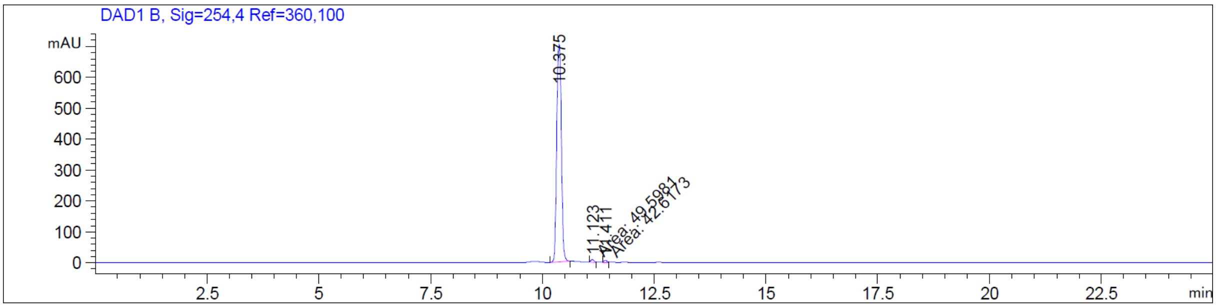

Signal 2: DAD1 B, Sig=254,4 Ref=360,100

Peak #	RetTime [min]	Type	Width [min]	Area [mAU*s]	Height [mAU]	Area %
1	10.375	BB	0.1161	5071.67578	703.44751	98.2142
2	11.123	MM	0.0933	49.59809	8.86044	0.9605
3	11.411	MM	0.0926	42.61728	7.66941	0.8253
Totals :				5163.89116	719.97736	

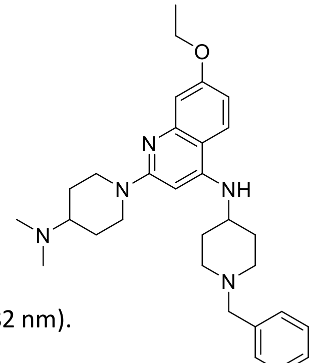

Cpd-2l

Figure S36: HPLC chromatogram of Compound **2l** (282 nm).

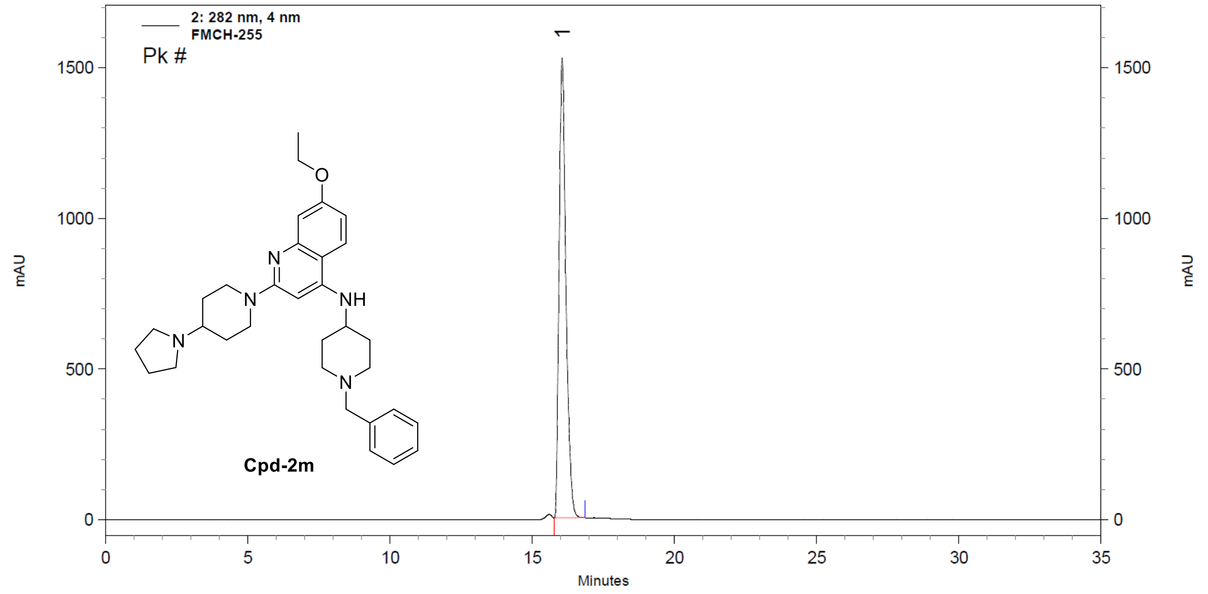

2: 282 nm, 4 nm Results			
Pk #	Retention Time	Area Percent	Lambda Max
1	16,053	100,000	321

Figure S37: HPLC chromatogram of Compound **2m** (282 nm).

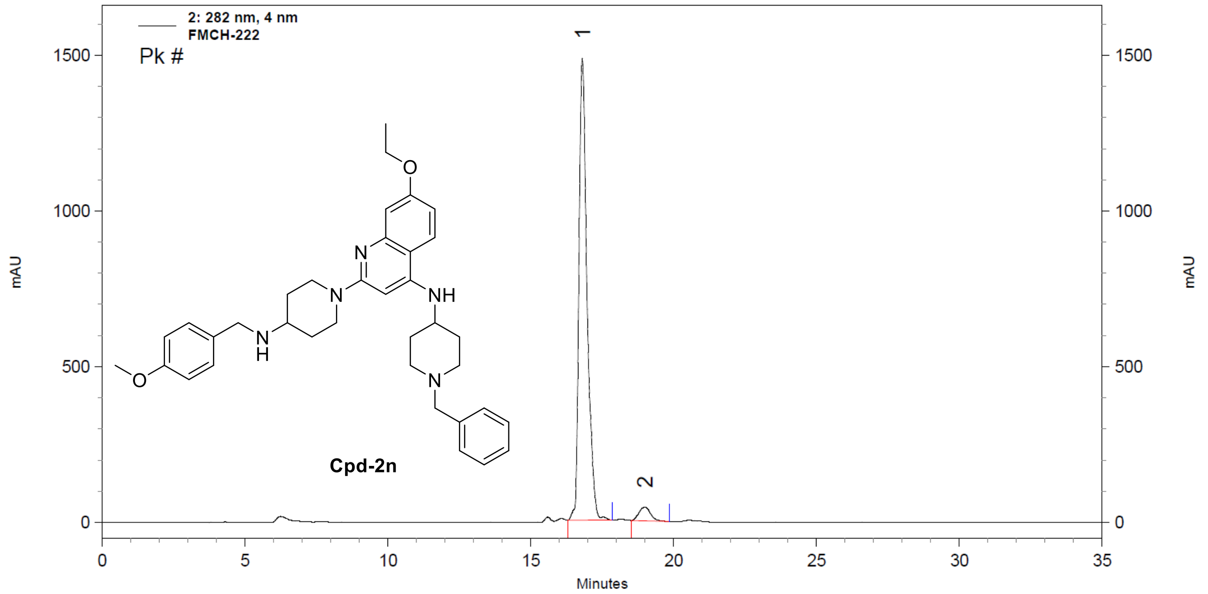

2: 282 nm, 4 nm Results			
Pk #	Retention Time	Area Percent	Lambda Max
1	16,807	95,923	322
2	19,000	4,077	282

Figure S38: HPLC chromatogram of Compound **2n** (282 nm).

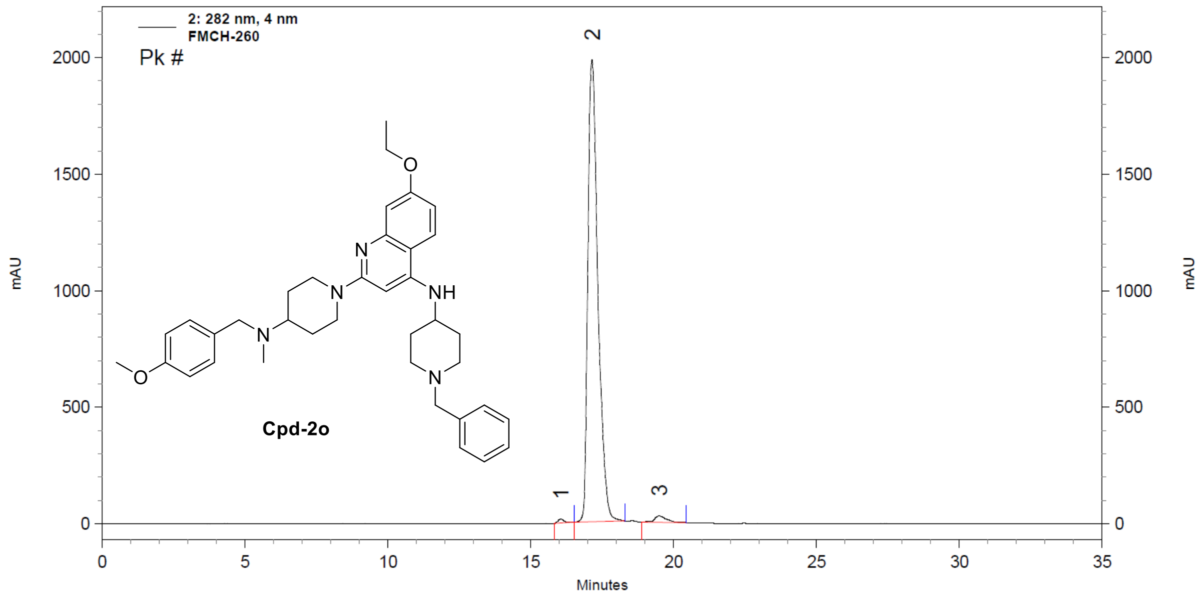

2: 282 nm, 4 nm Results

Pk #	Retention Time	Area Percent	Lambda Max
1	16,053	0,496	249
2	17,147	97,873	323
3	19,500	1,631	256

Figure S39: HPLC chromatogram of Compound **2o** (282 nm).

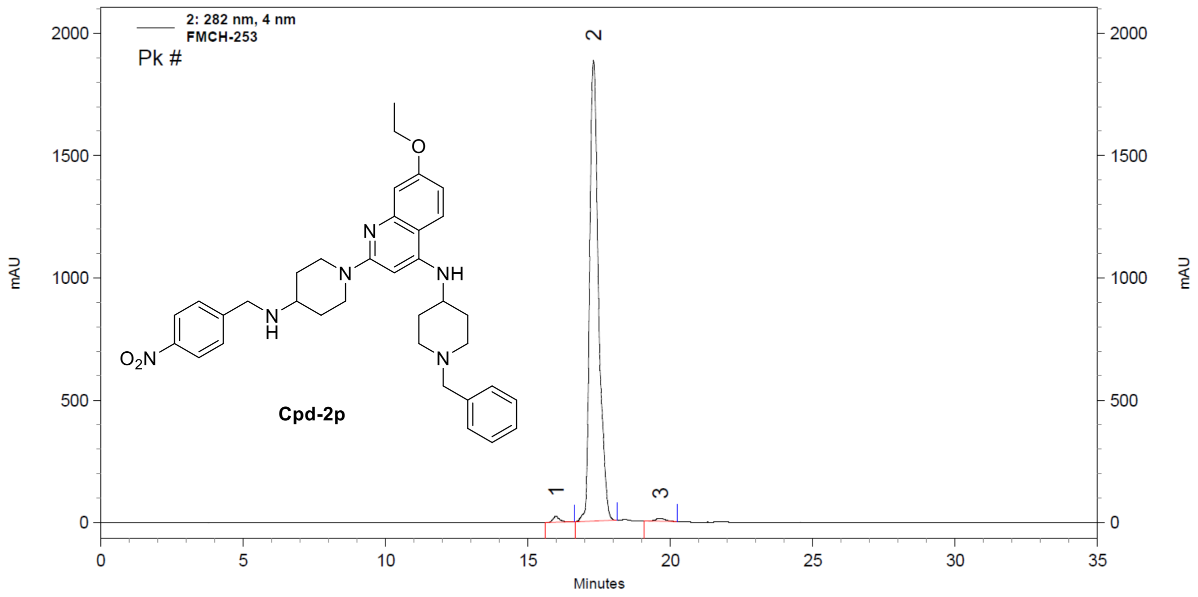

2: 282 nm, 4 nm Results

Pk #	Retention Time	Area Percent	Lambda Max
1	15,987	0,940	250
2	17,307	98,348	321
3	19,647	0,711	257

Figure S40: HPLC chromatogram of Compound **2p** (282 nm).

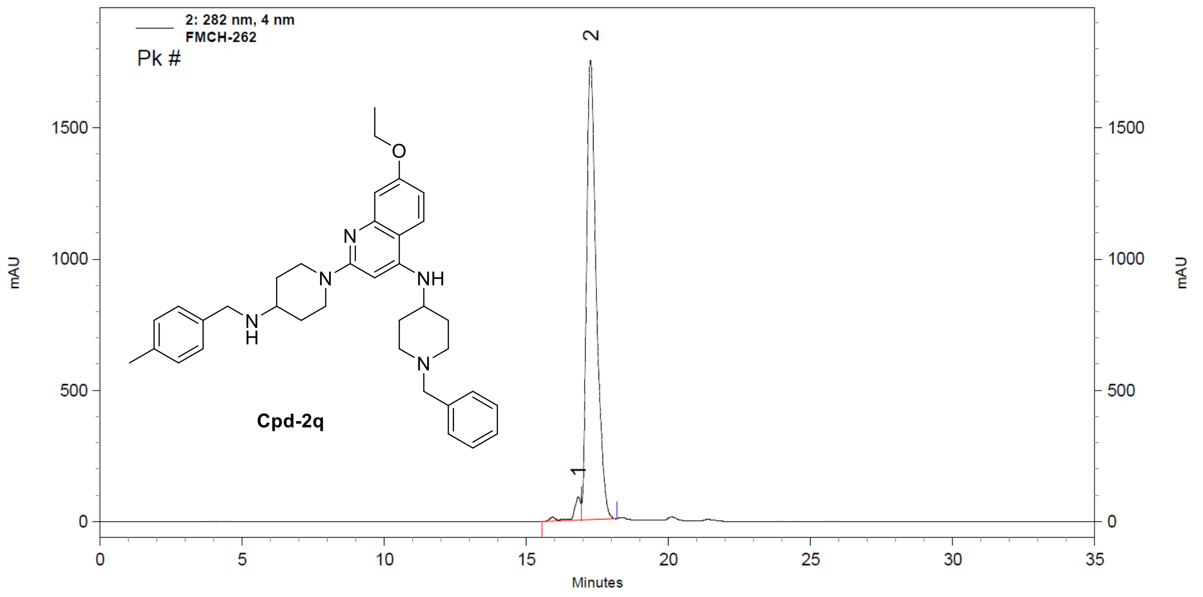

2: 282 nm, 4 nm Results

Pk #	Retention Time	Area Percent	Lambda Max
1	16,827	3,616	242
2	17,260	96,384	323

Figure S41: HPLC chromatogram of Compound **2q** (282 nm).

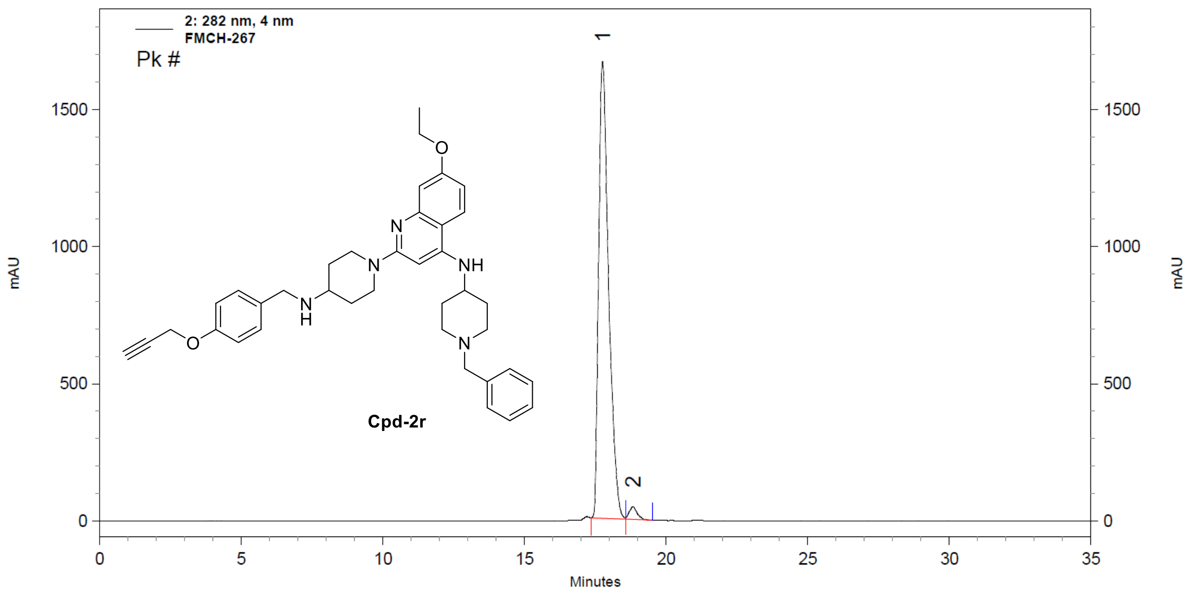

2: 282 nm, 4 nm Results

Pk #	Retention Time	Area Percent	Lambda Max
1	17,760	97,850	323
2	18,833	2,150	249

Figure S42: HPLC chromatogram of Compound **2r** (282 nm).

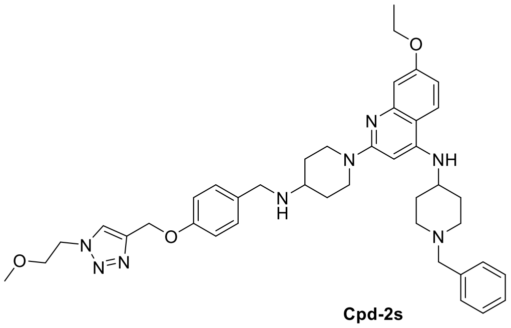

Cpd-2s

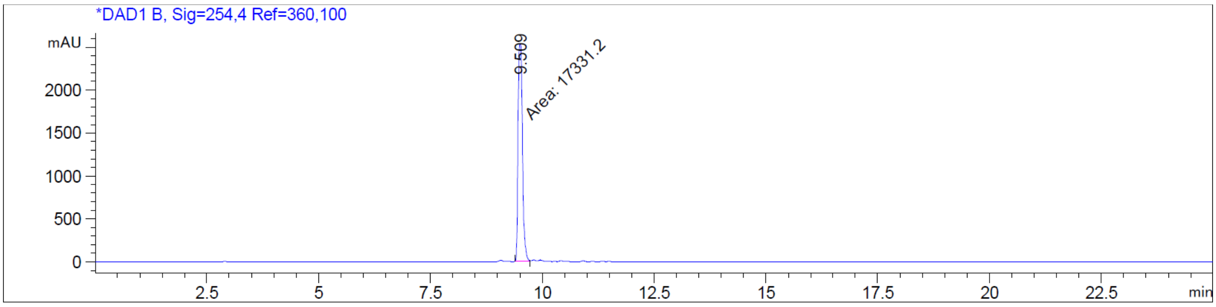

Signal 2: DAD1 B, Sig=254,4 Ref=360,100

Peak #	RetTime [min]	Type	Width [min]	Area [mAU*s]	Height [mAU]	Area %
1	9.509	MM	0.1140	1.73312e4	2534.77979	100.0000

Totals : 1.73312e4 2534.77979

Figure S43: HPLC chromatogram of Compound **2s** (254 nm).

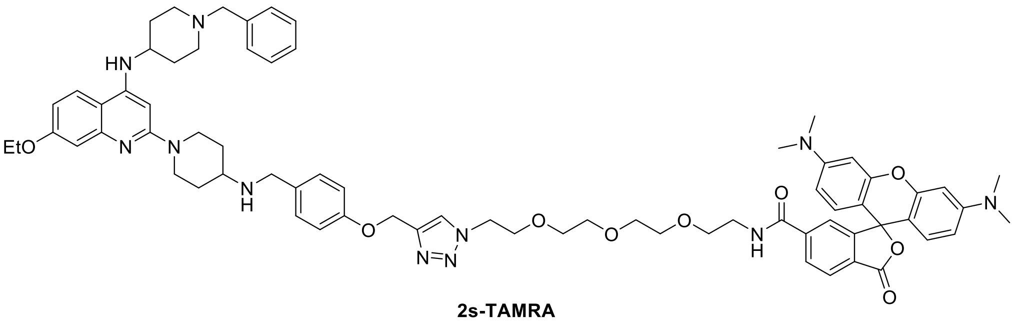

2s-TAMRA

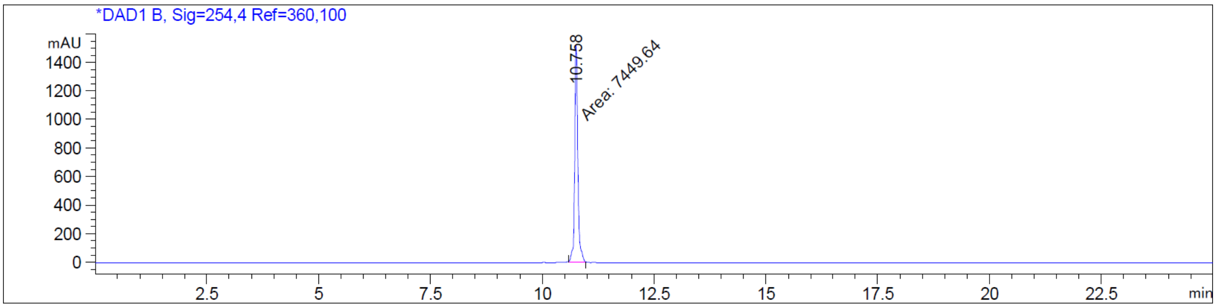

Signal 2: DAD1 B, Sig=254,4 Ref=360,100

Peak #	RetTime [min]	Type	Width [min]	Area [mAU*s]	Height [mAU]	Area %
1	10.758	MM	0.0813	7449.64209	1527.25317	100.0000
Totals :				7449.64209	1527.25317	

Figure S44: HPLC chromatogram of Compound **2s-TAMRA** (254 nm).

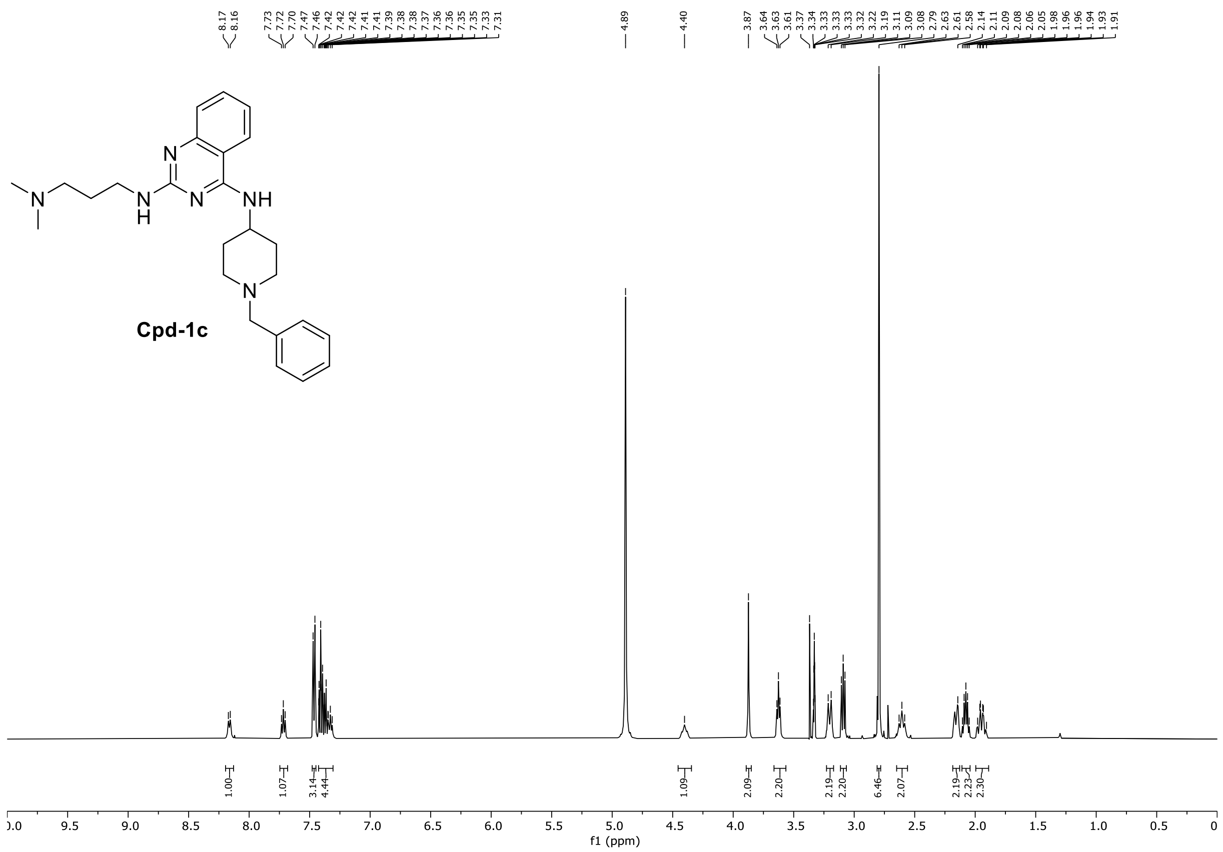

Figure S45: ^{1}H NMR spectrum of Compound **1c**.

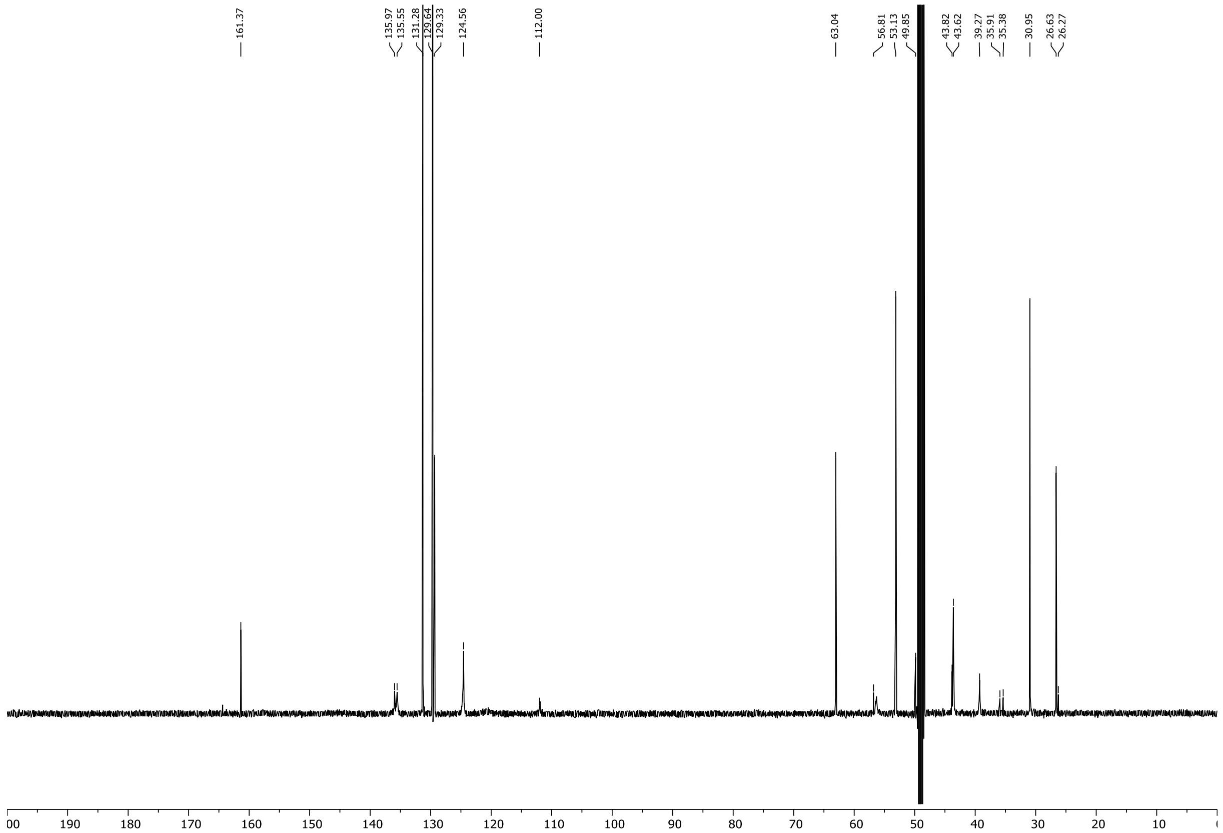

Figure S46: ^{13}C NMR spectrum of Compound **1c**.

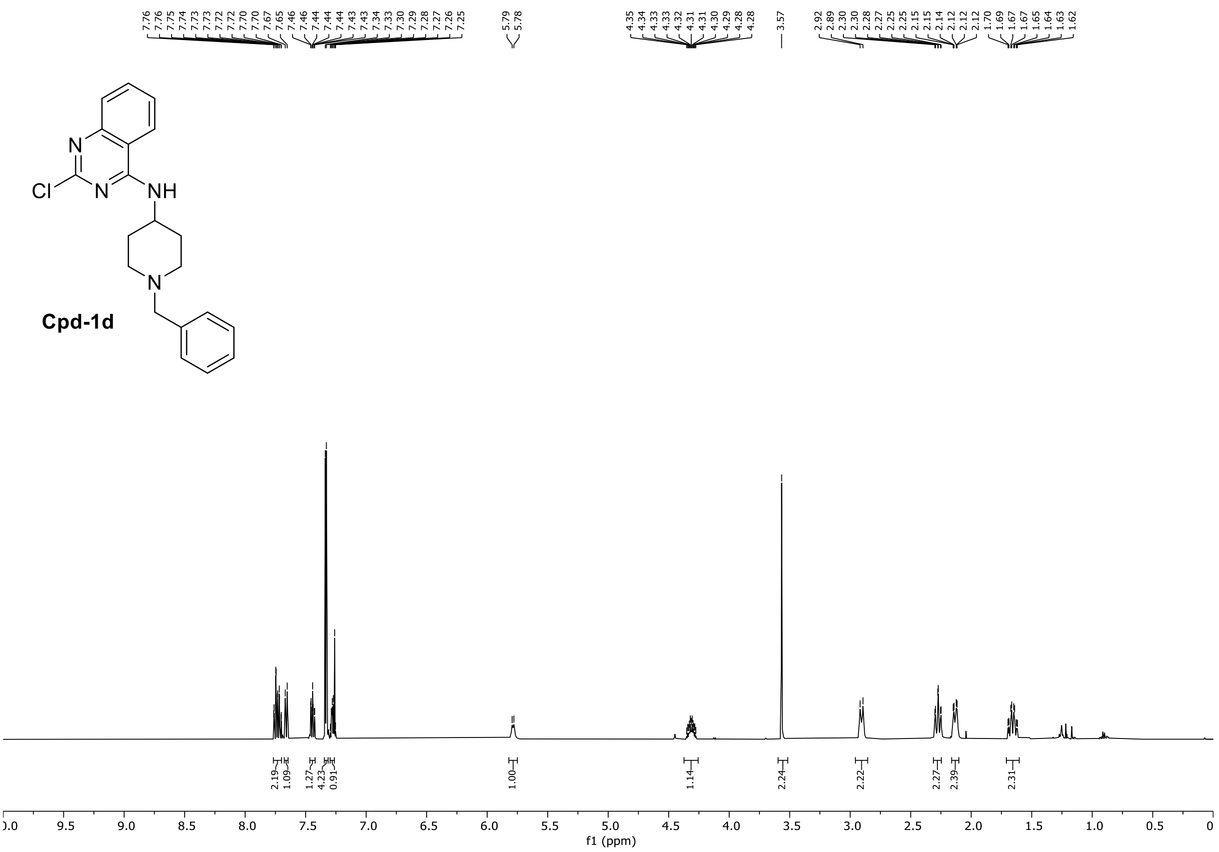

Figure S47: ^{1}H NMR spectrum of Compound **1d**.

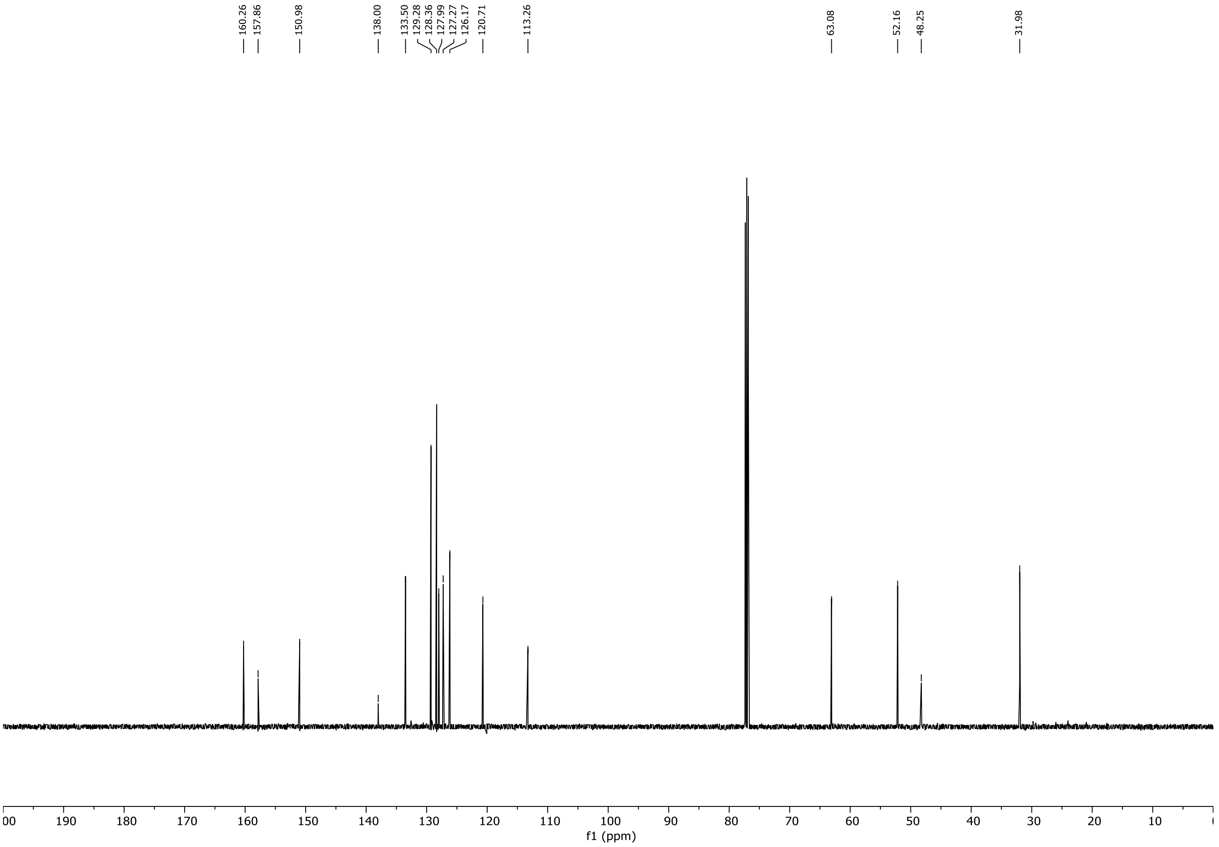

Figure S48: ^{13}C NMR spectrum of Compound **1d**.

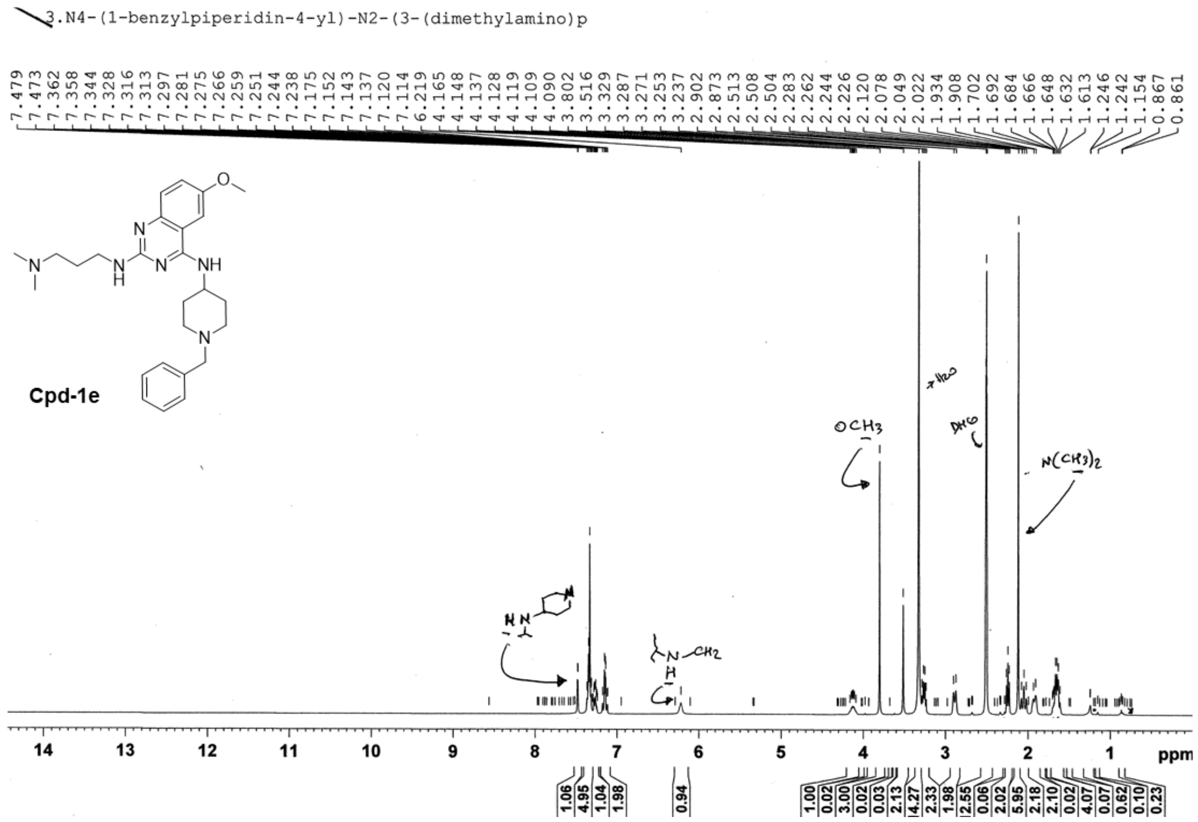

Figure S49: ^{1}H NMR spectrum of Compound **1e**.

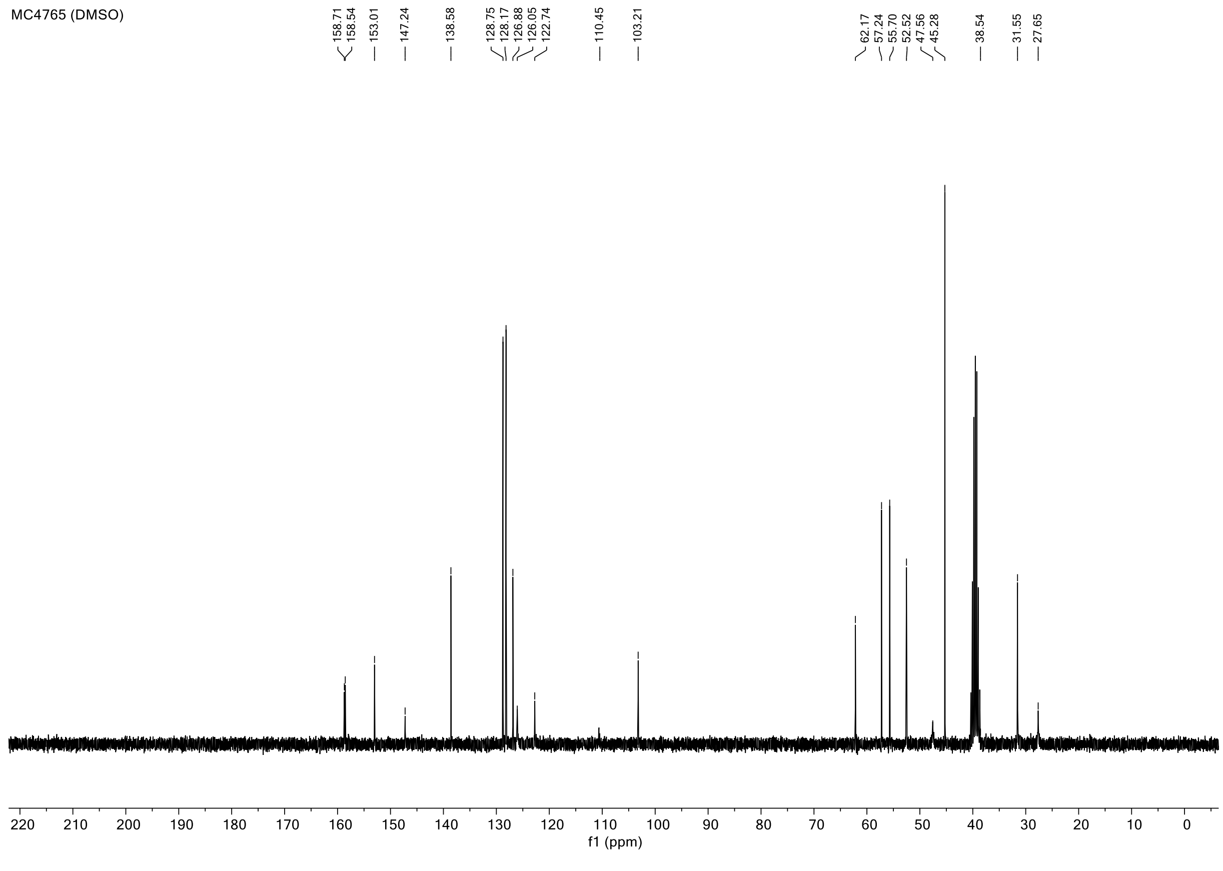

Figure S50: ^{13}C NMR spectrum of Compound **1e**.

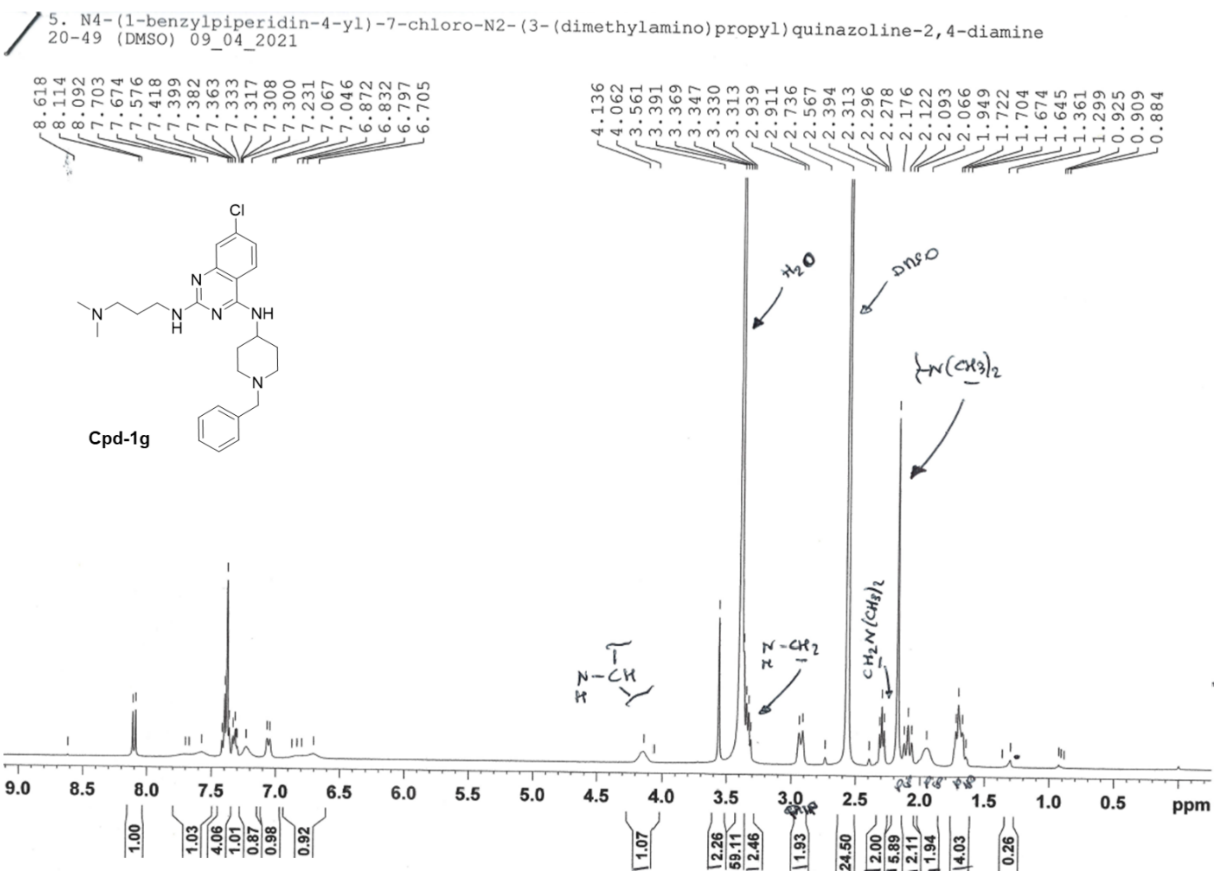

Figure S51: ^{1}H NMR spectrum of Compound **1g**.

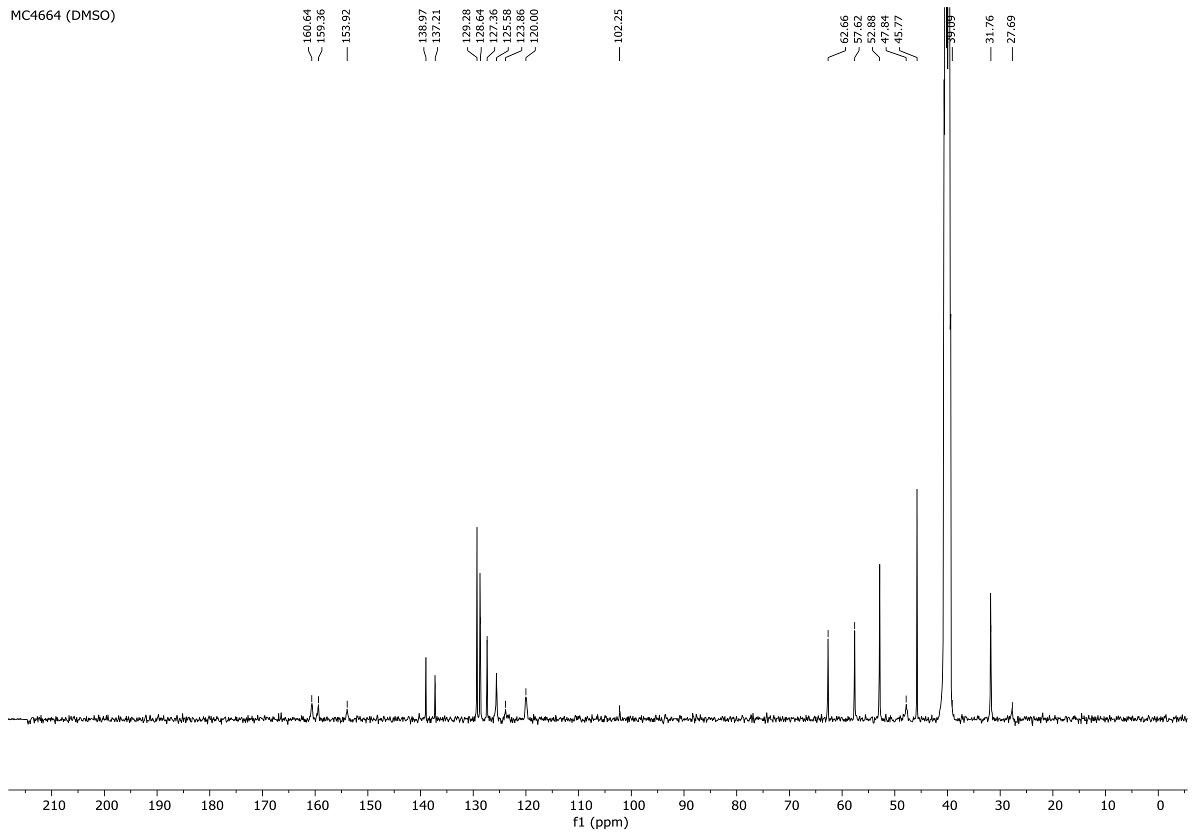

Figure S52: ^{13}C NMR spectrum of Compound **1g**.

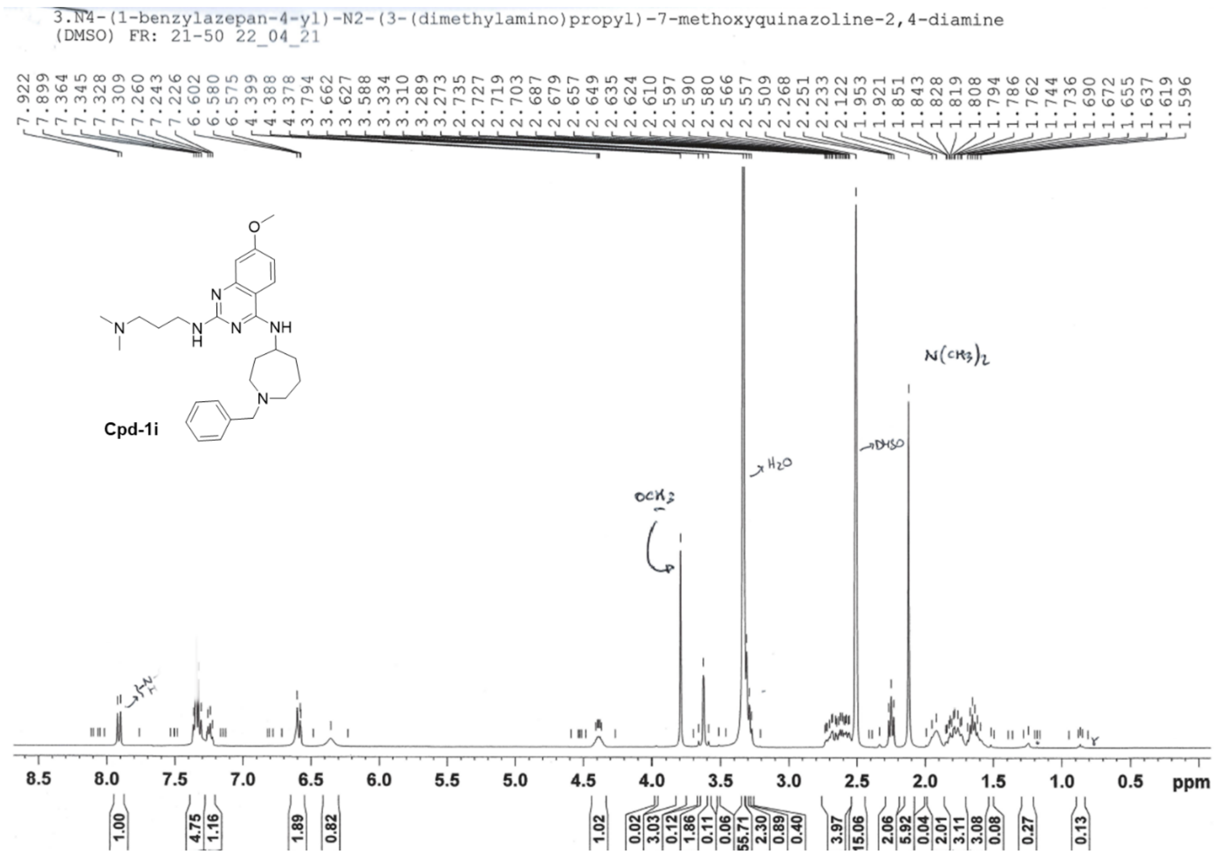

Figure S53: ^{1}H NMR spectrum of Compound **1i**.

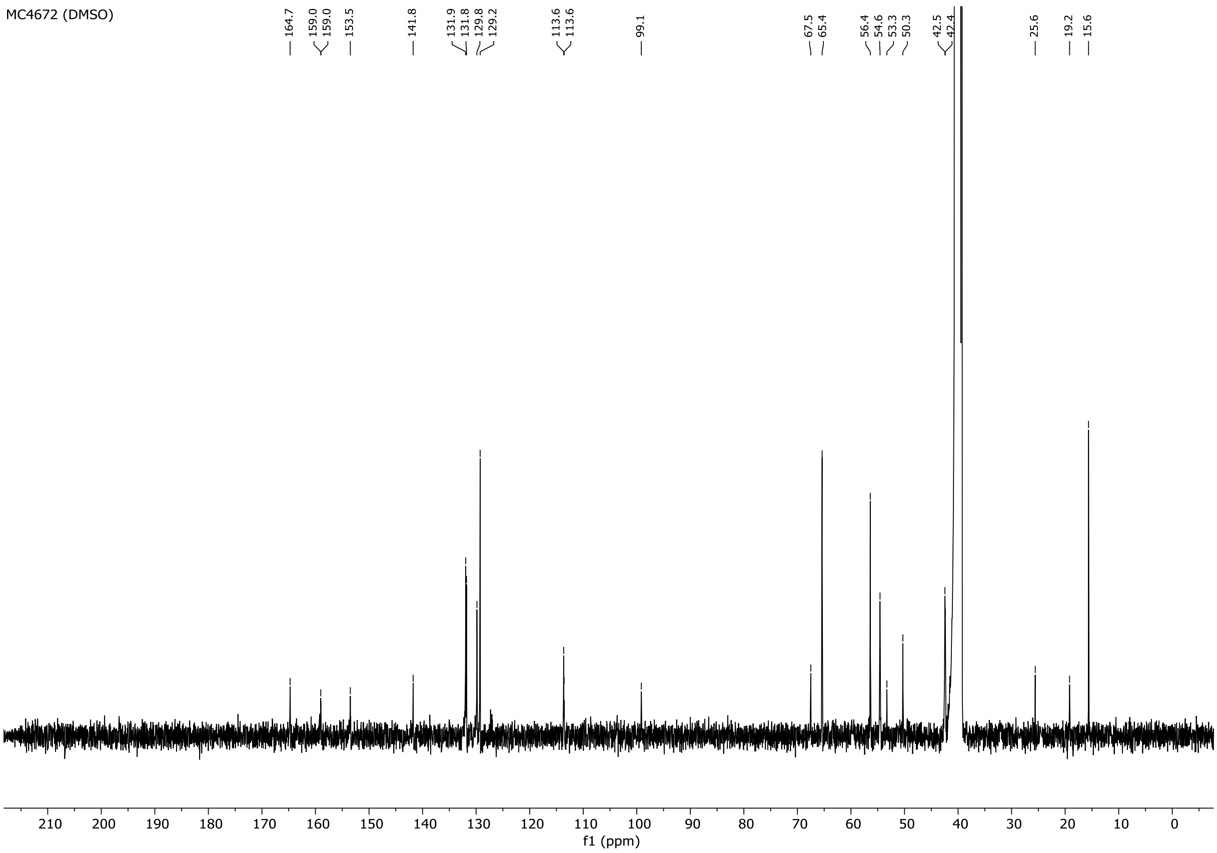

Figure S54: ^{13}C NMR spectrum of Compound **1i**.

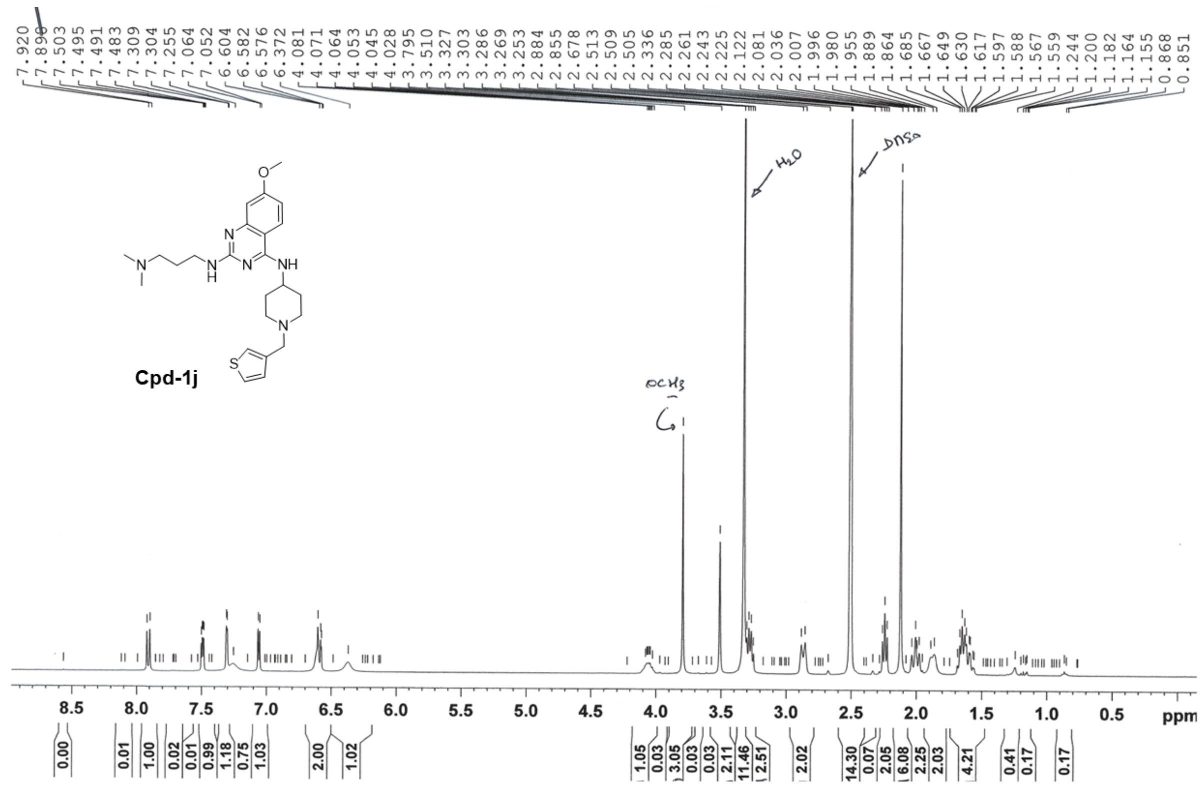

Figure S55: ^{1}H NMR spectrum of Compound **1j**.

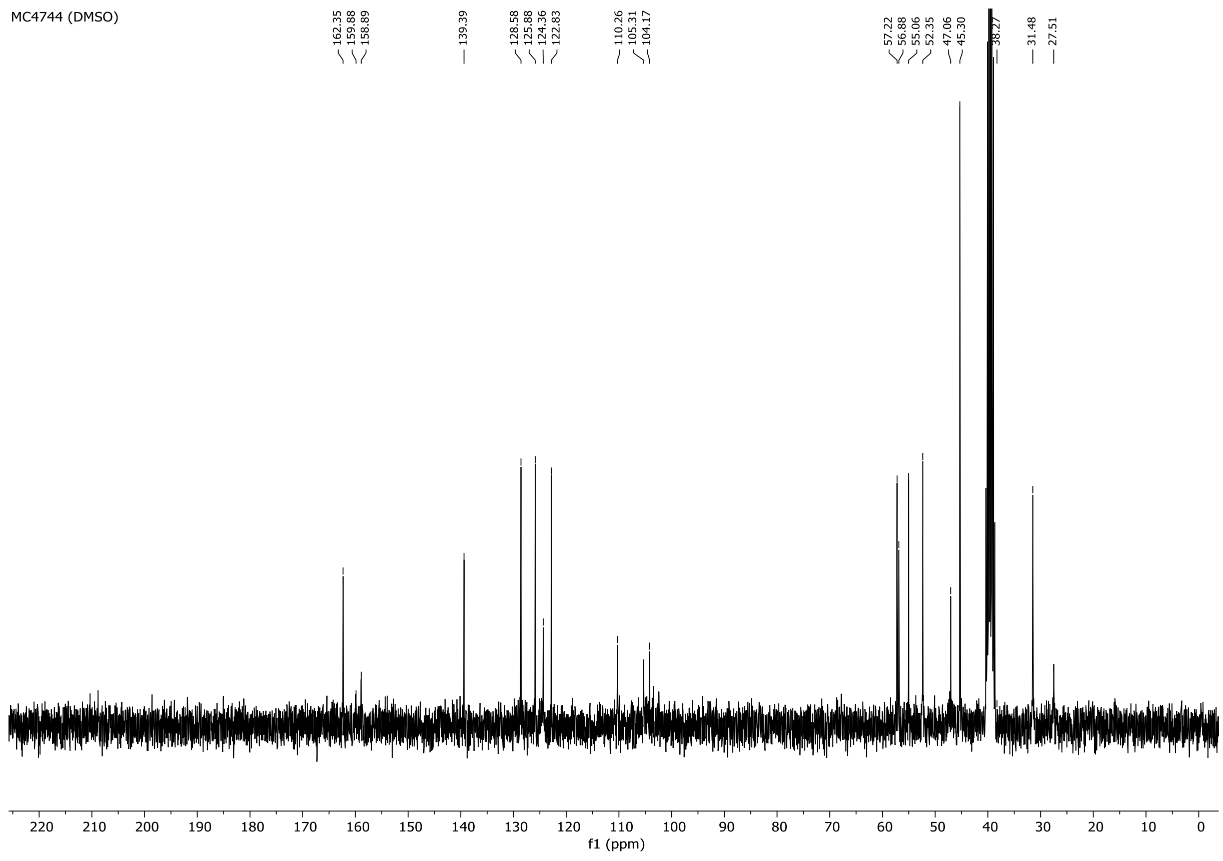

Figure S56: ^{13}C NMR spectrum of Compound **1j**.

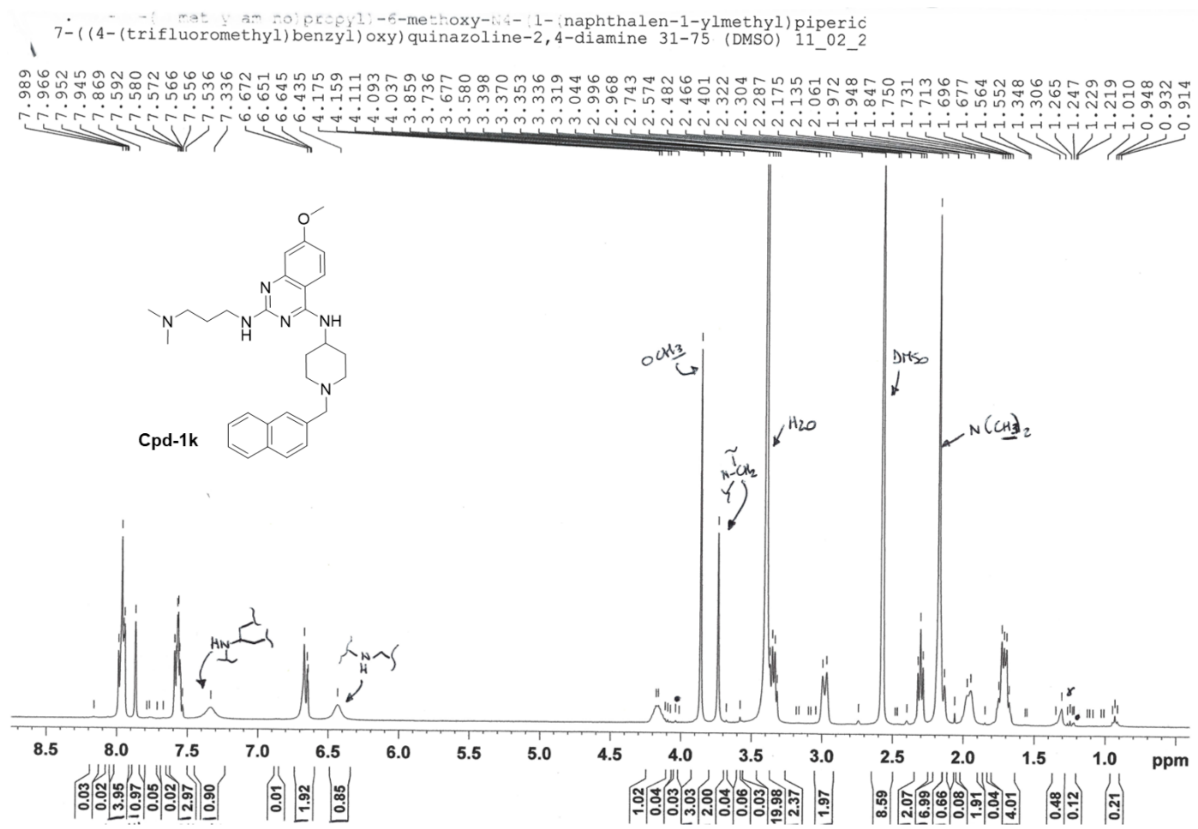

Figure S57: ^{1}H NMR spectrum of Compound **1k**.

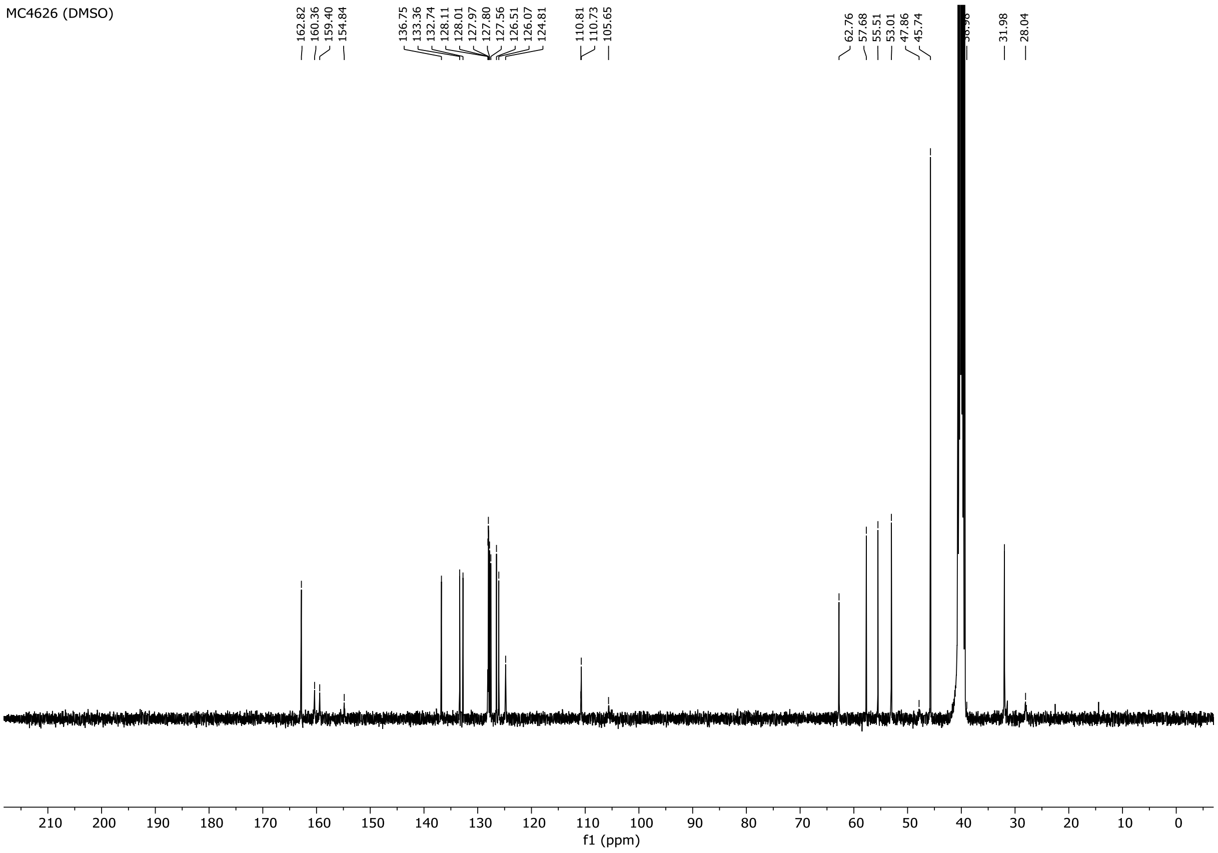

Figure S58: ^{13}C NMR spectrum of Compound **1k**.

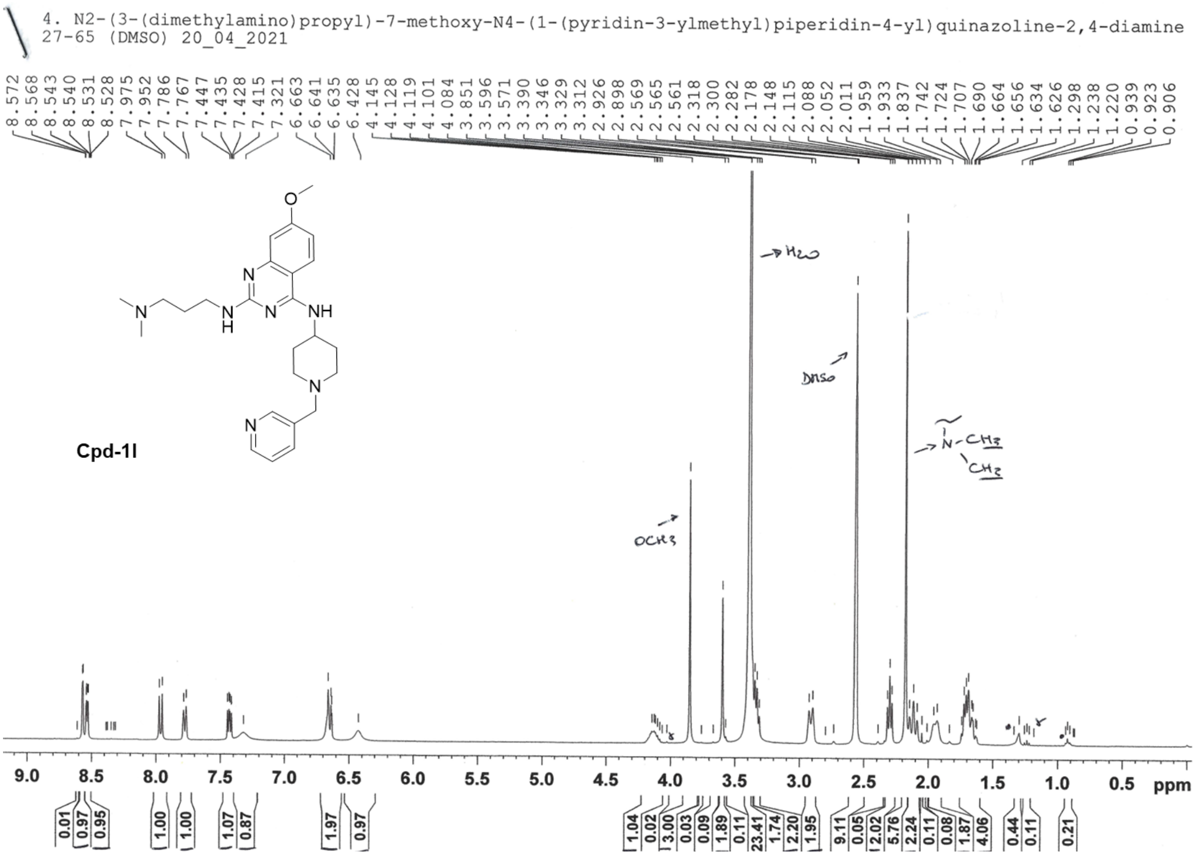

Figure S59: ^{1}H NMR spectrum of Compound **1l**.

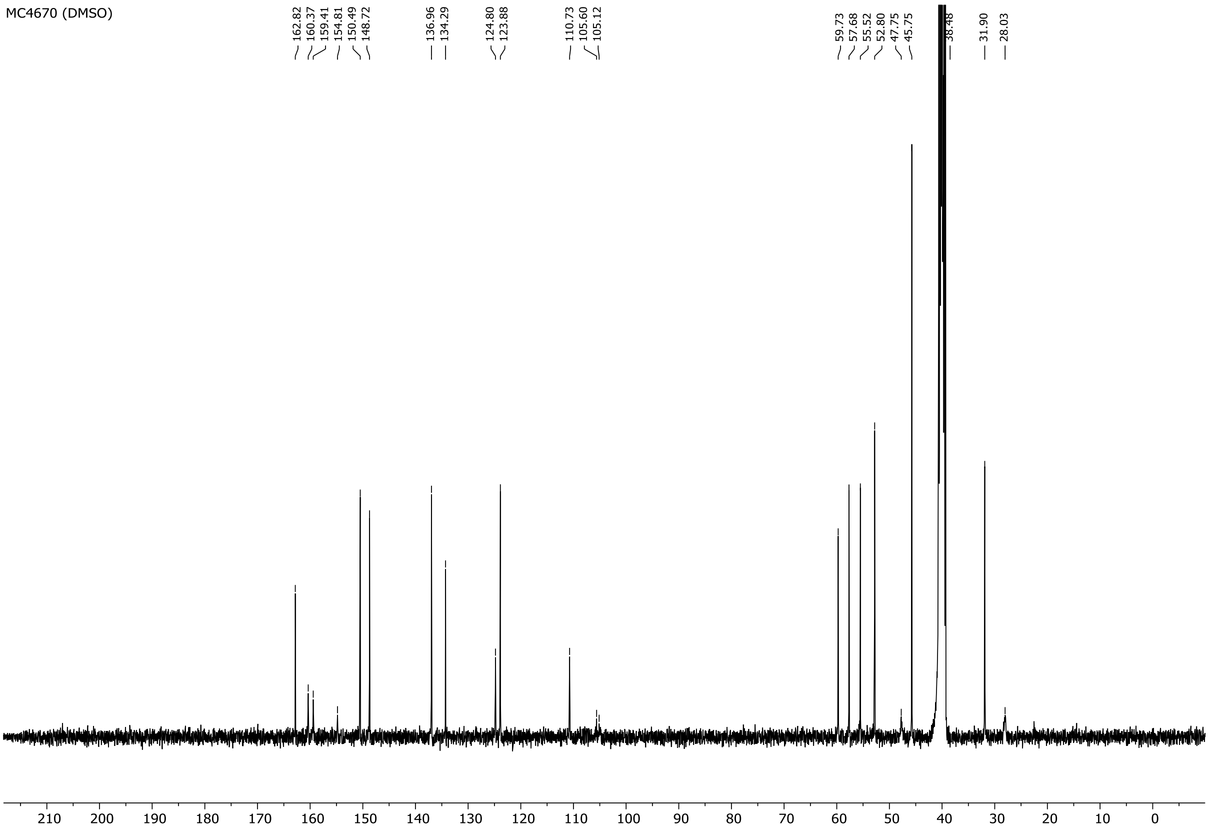

Figure S60: ^{13}C NMR spectrum of Compound **1l**.

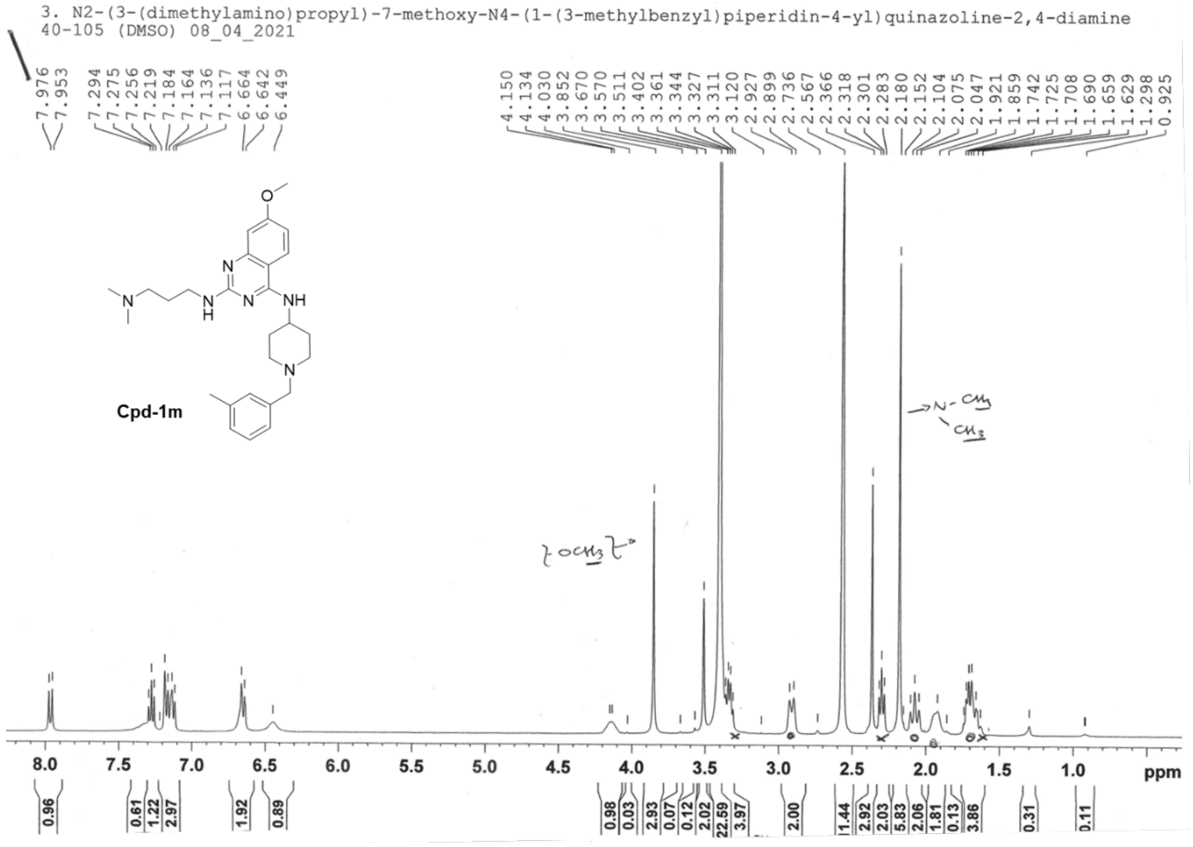

Figure S61: ^{1}H NMR spectrum of Compound **1m**.

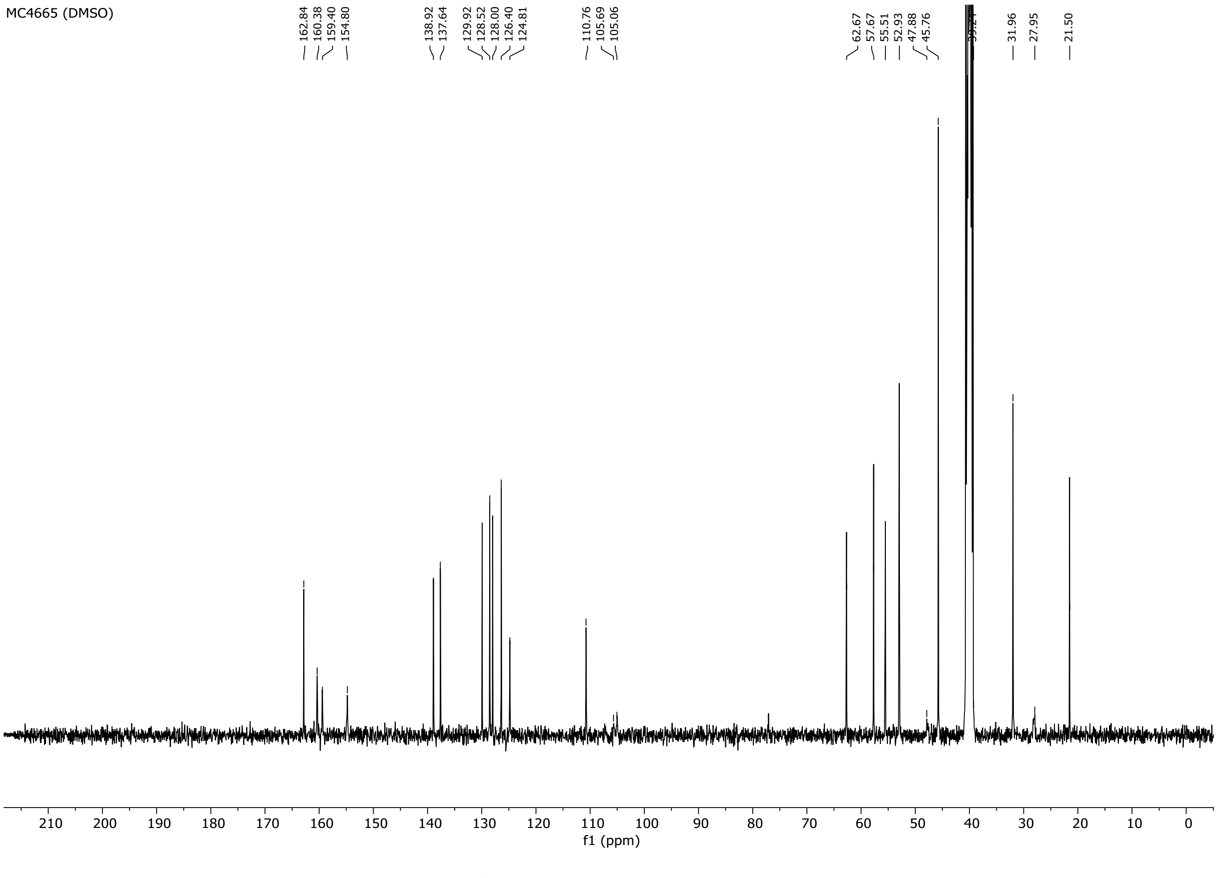

Figure S62: ^{13}C NMR spectrum of Compound **1m**.

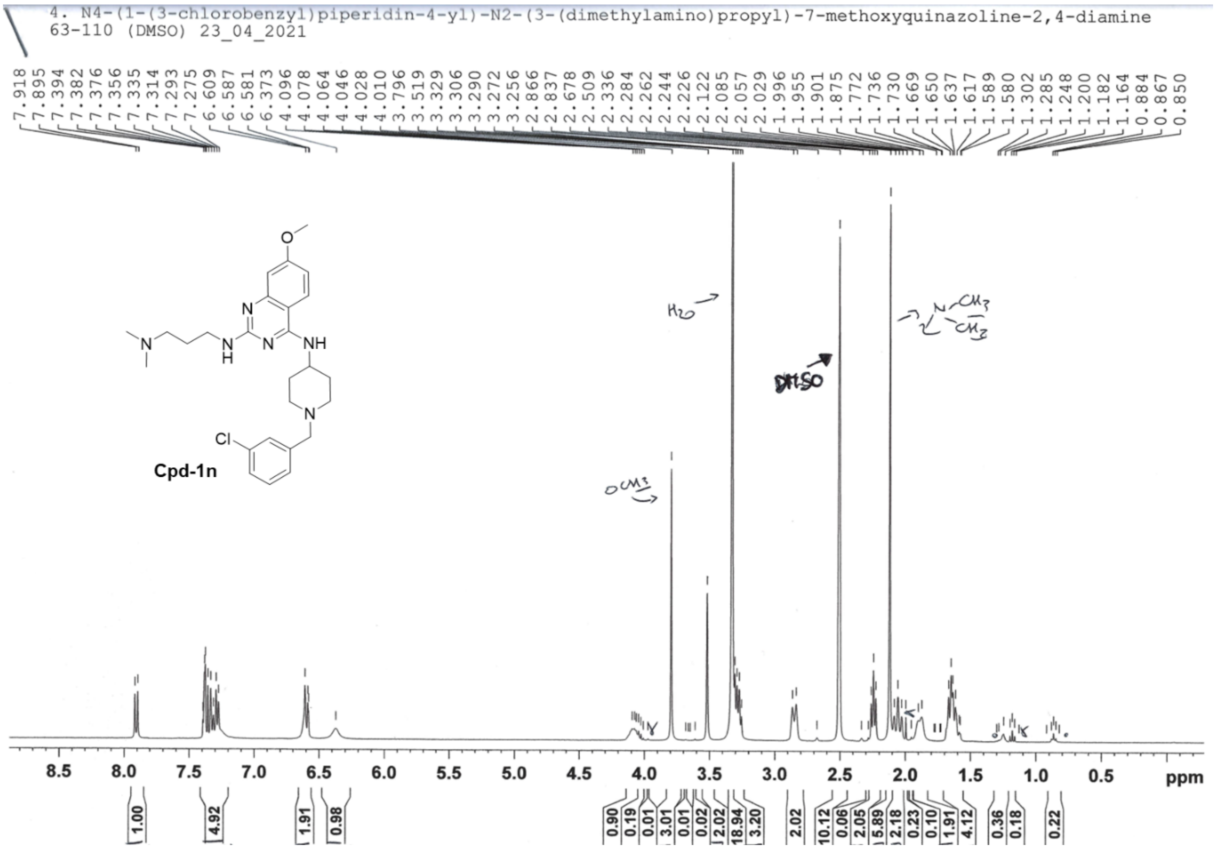

Figure S63: ^{1}H NMR spectrum of Compound **1n**.

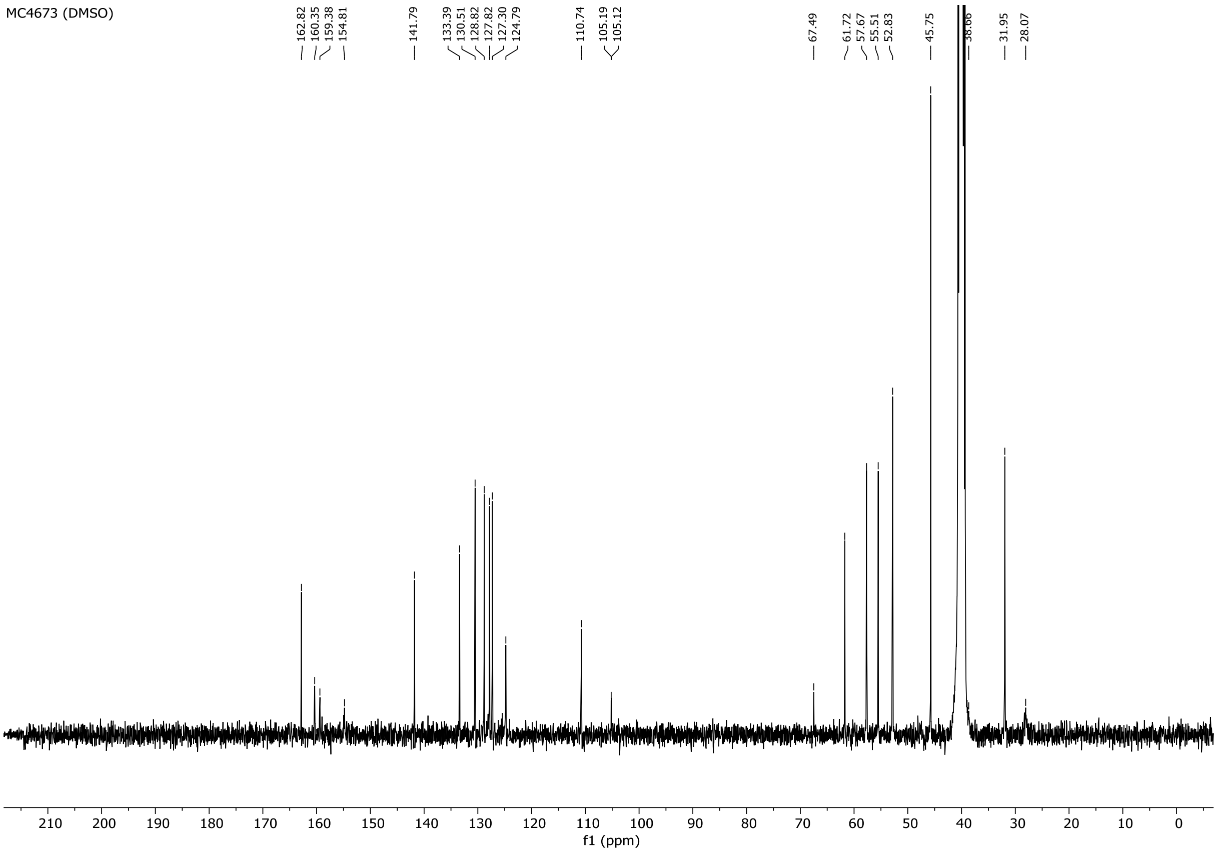

Figure S64: ^{13}C NMR spectrum of Compound **1n**.

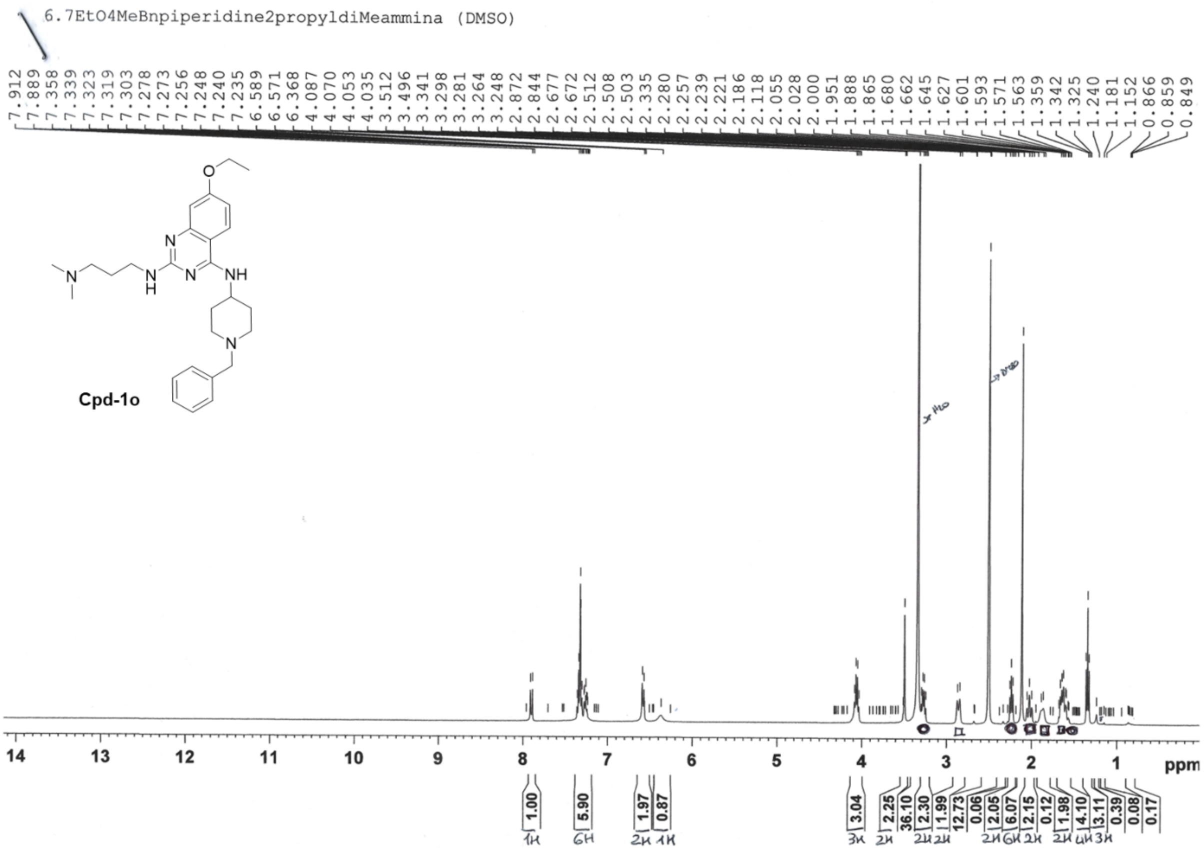

Figure S65: ^{1}H NMR spectrum of Compound **1o**.

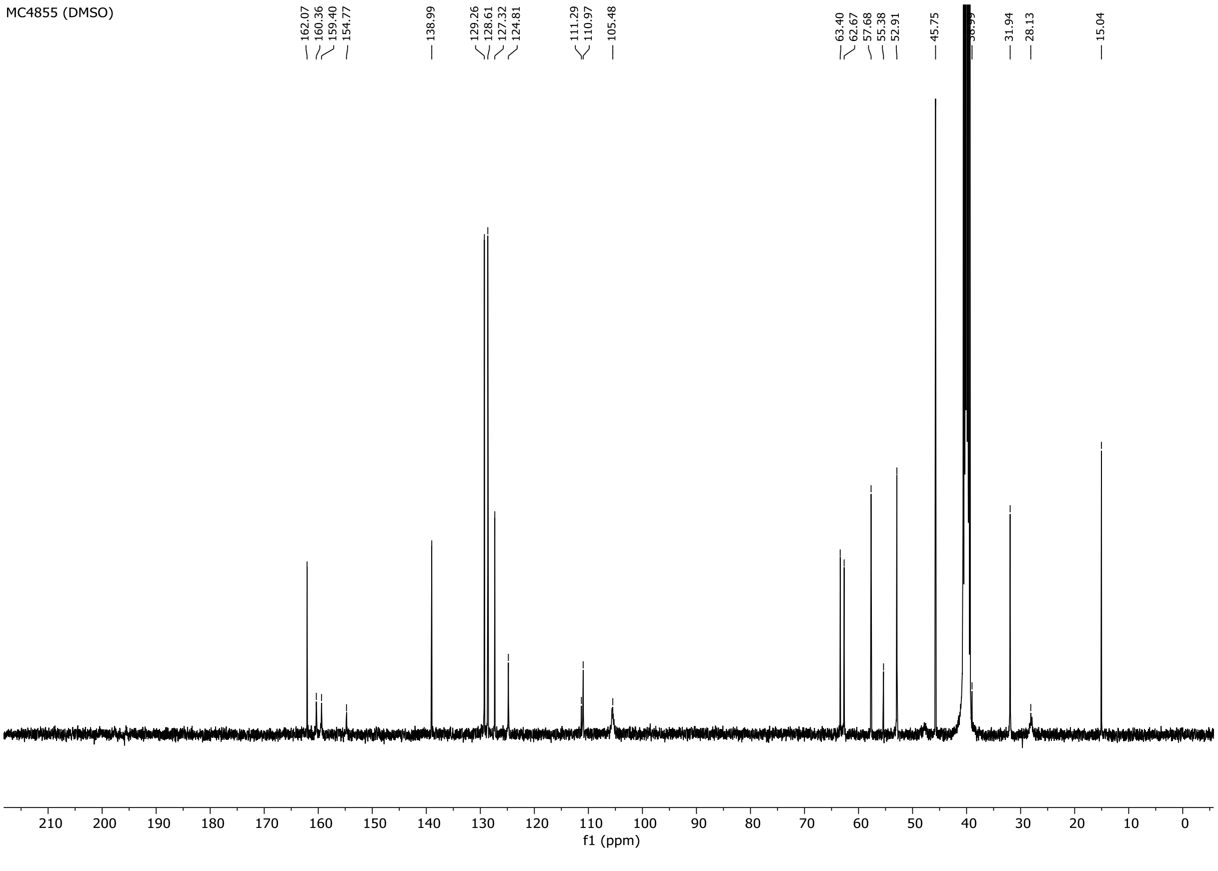

Figure S66: ^{13}C NMR spectrum of Compound **1o**.

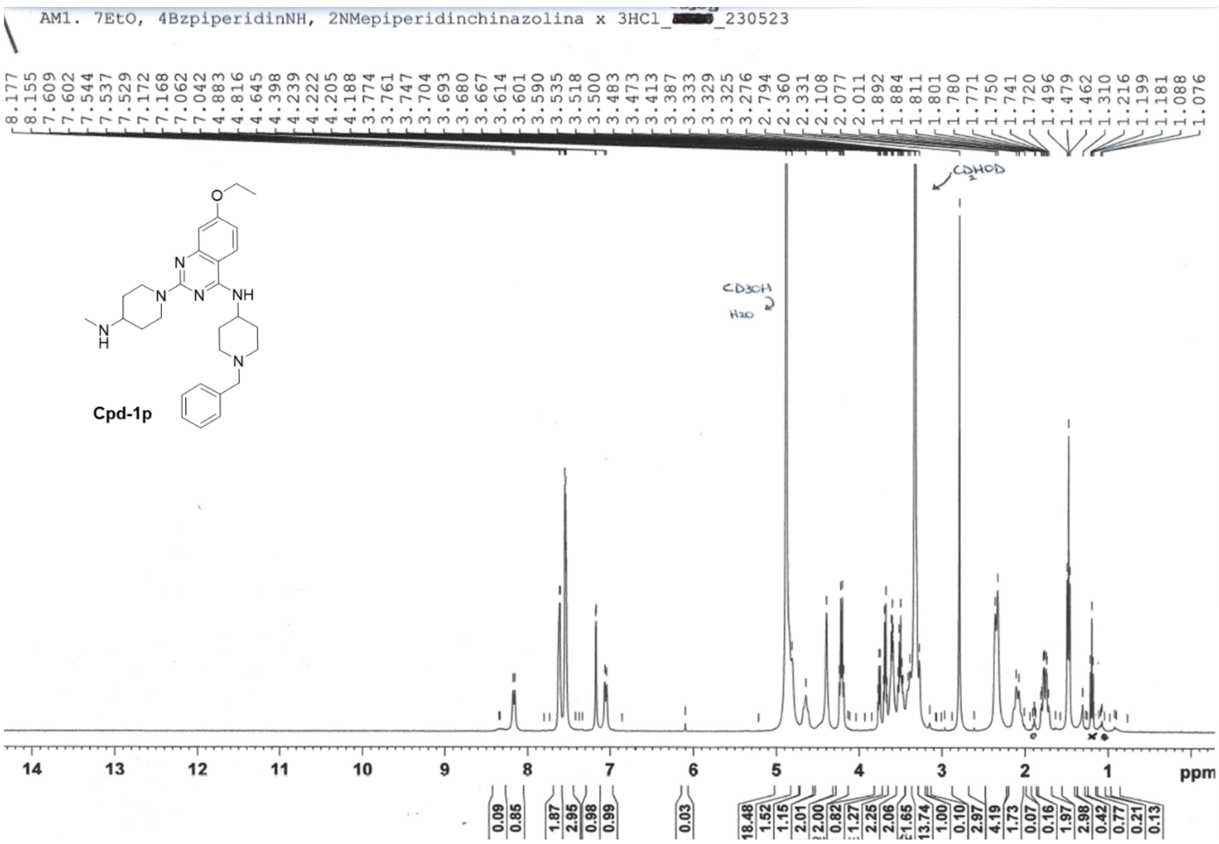

Figure S67: ^{1}H NMR spectrum of Compound **1p**.

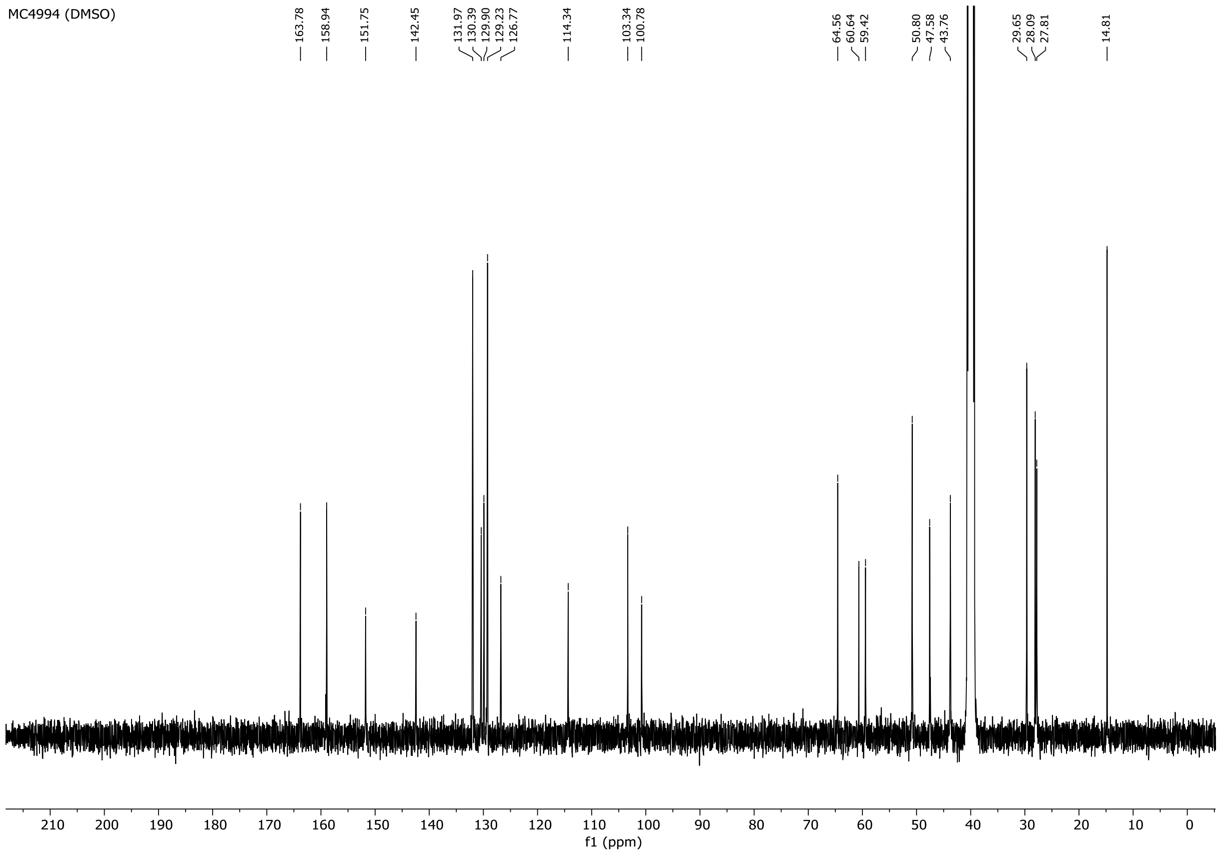

Figure S68: ^{13}C NMR spectrum of Compound **1p**.

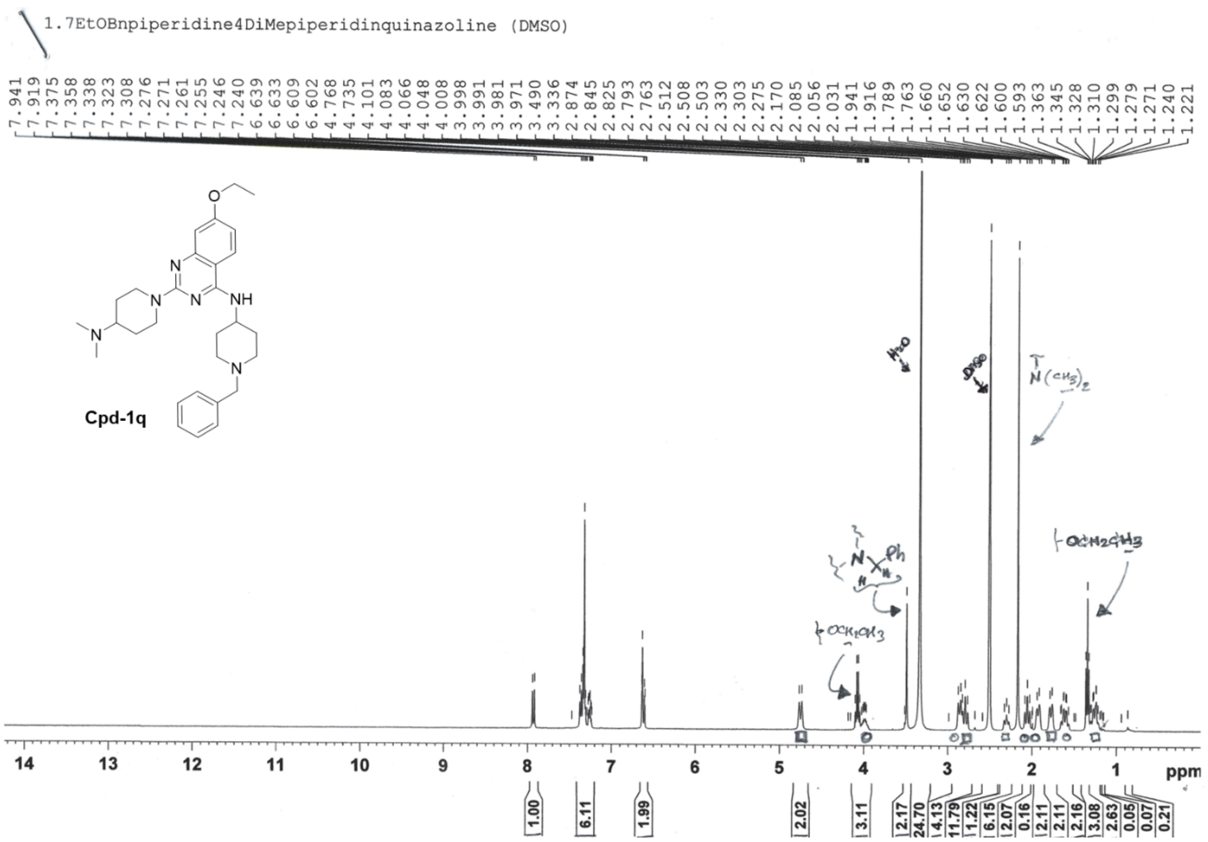

Figure S69: ^{1}H NMR spectrum of Compound **1q**.

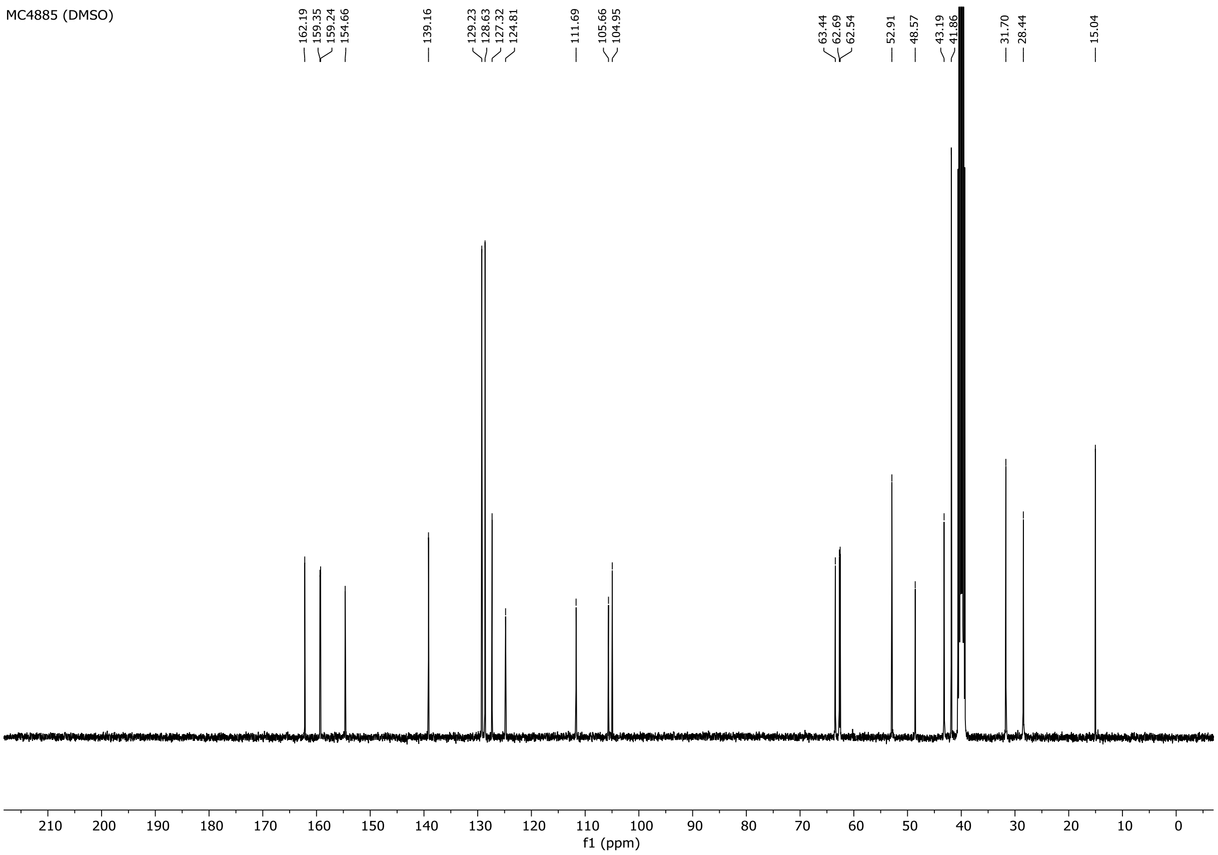

Figure S70: ^{13}C NMR spectrum of Compound **1q**.

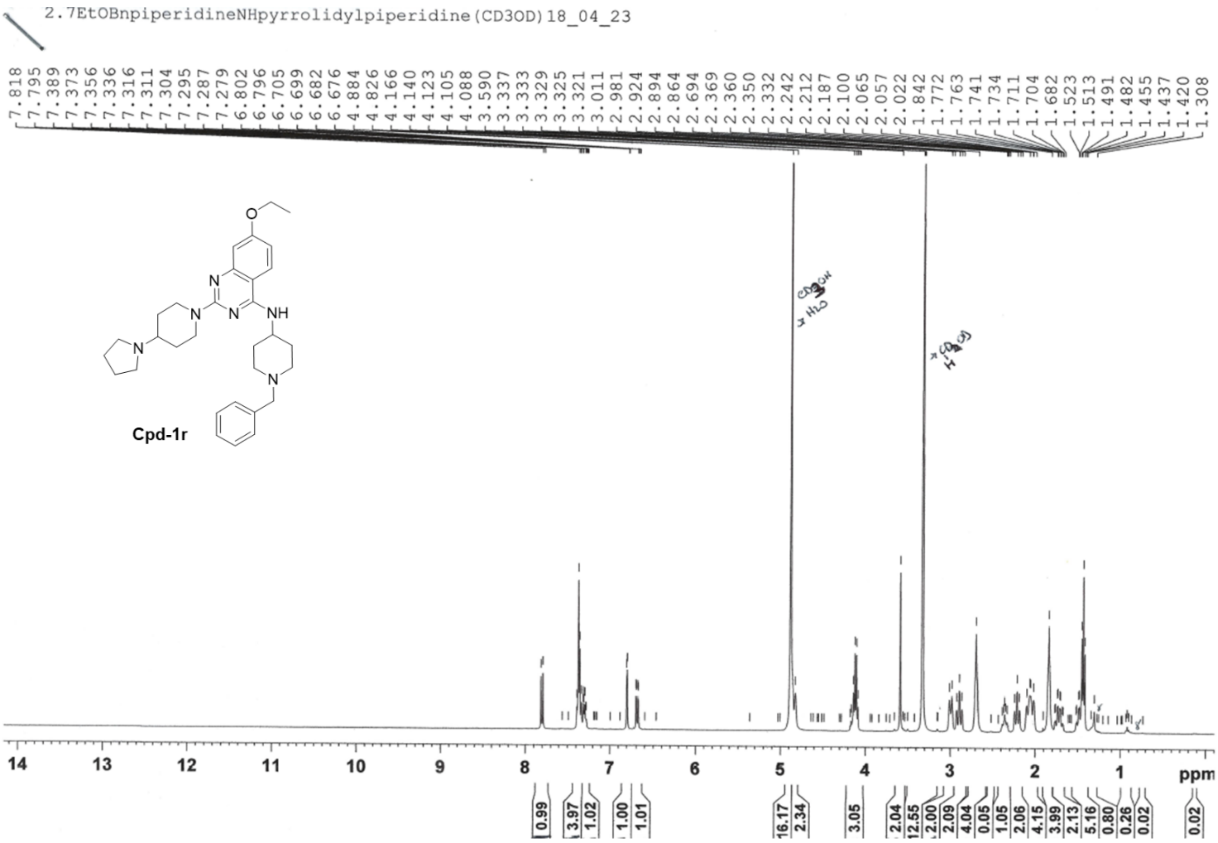

Figure S71: ^{1}H NMR spectrum of Compound **1r**.

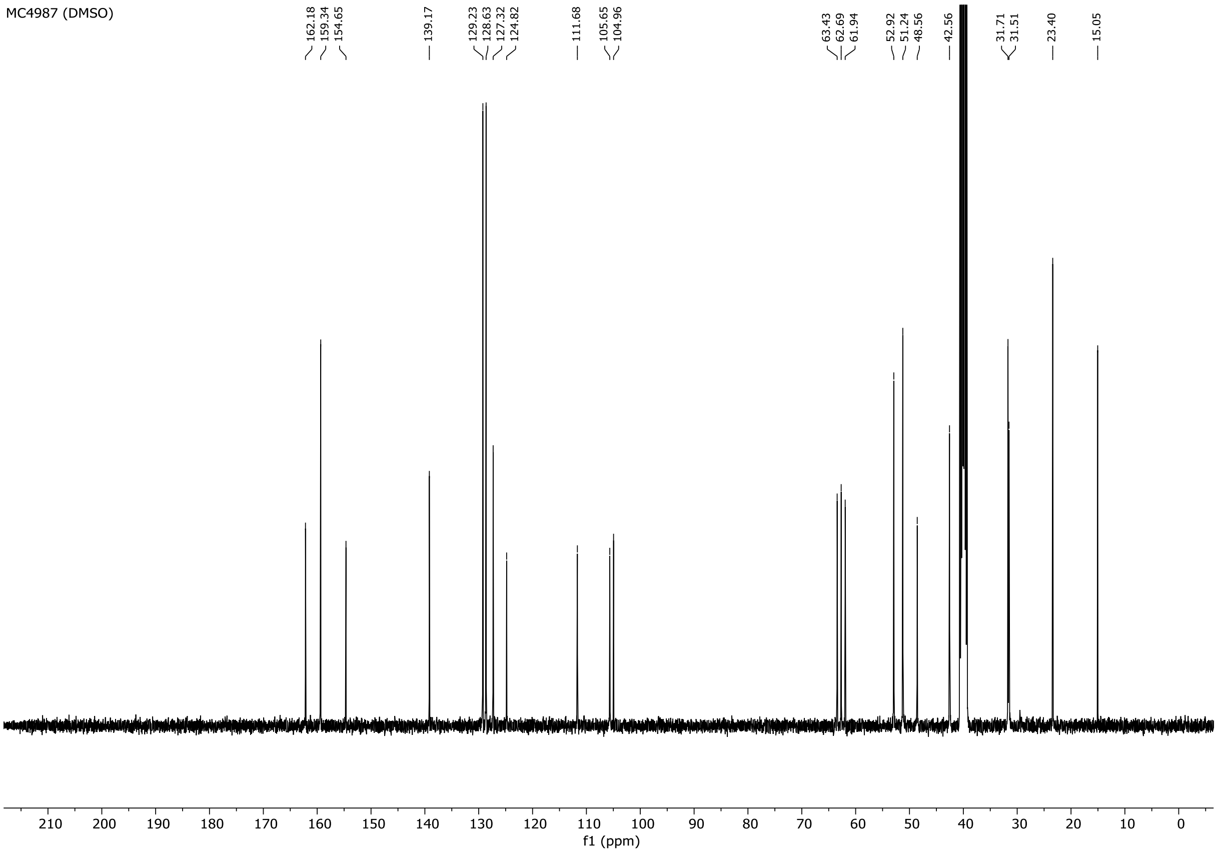

Figure S72: ^{13}C NMR spectrum of Compound **1r**.

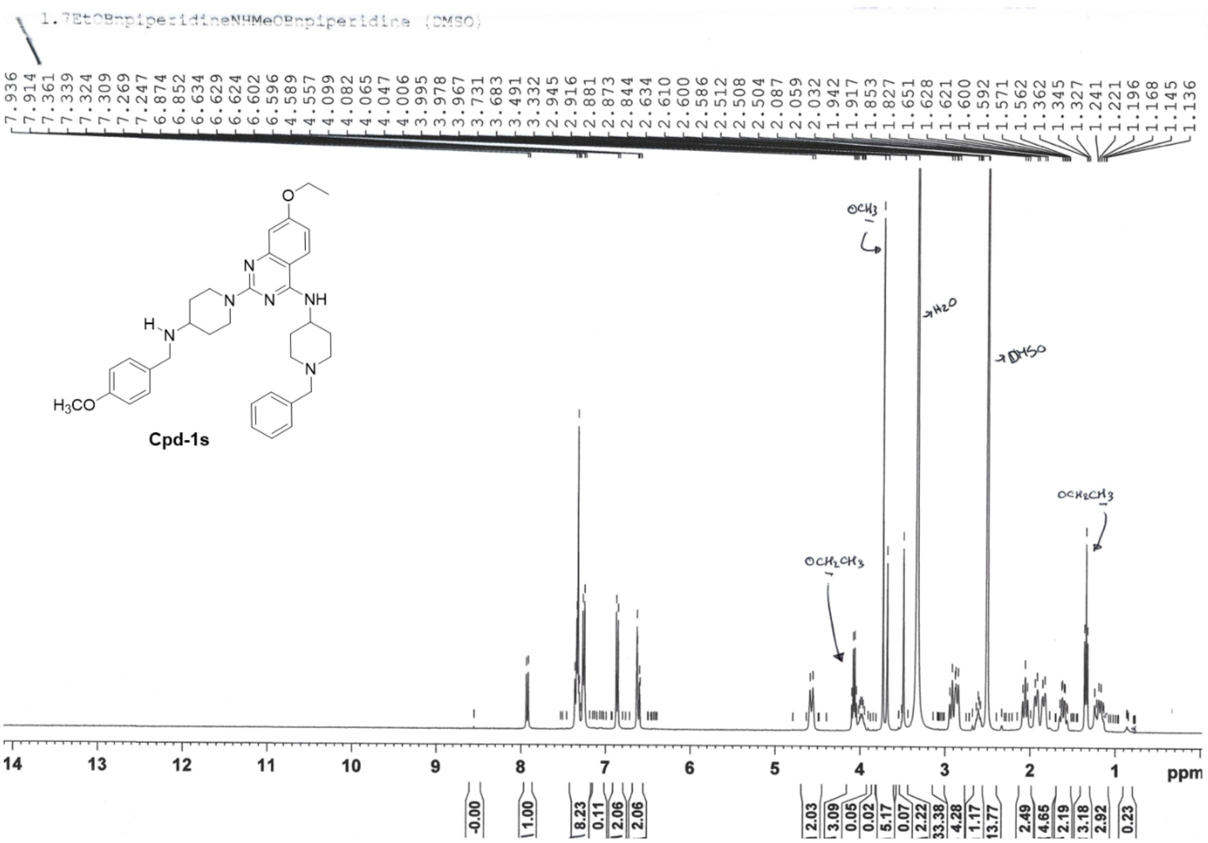

Figure S73: ^{1}H NMR spectrum of Compound **1s**.

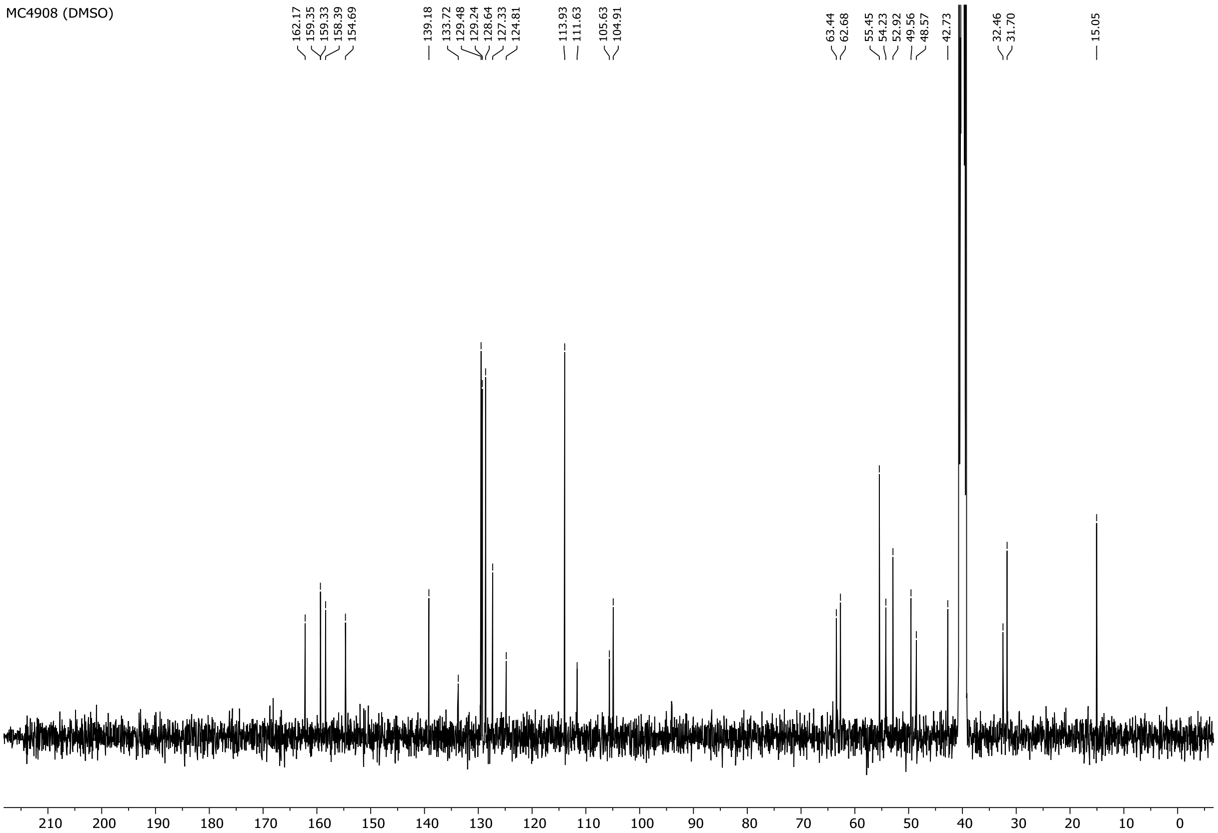

Figure S74: ^{13}C NMR spectrum of Compound **1s**.

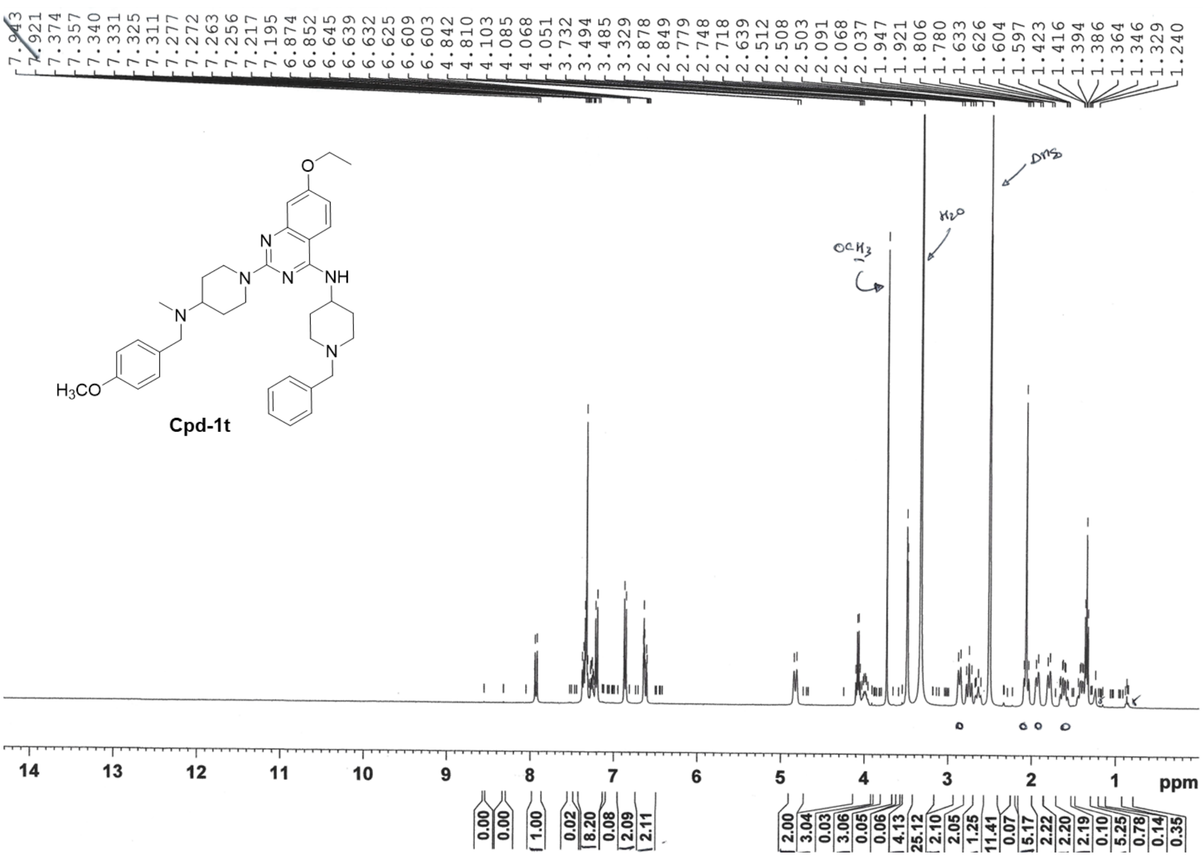

Figure S75: ^{1}H NMR spectrum of Compound **1t**.

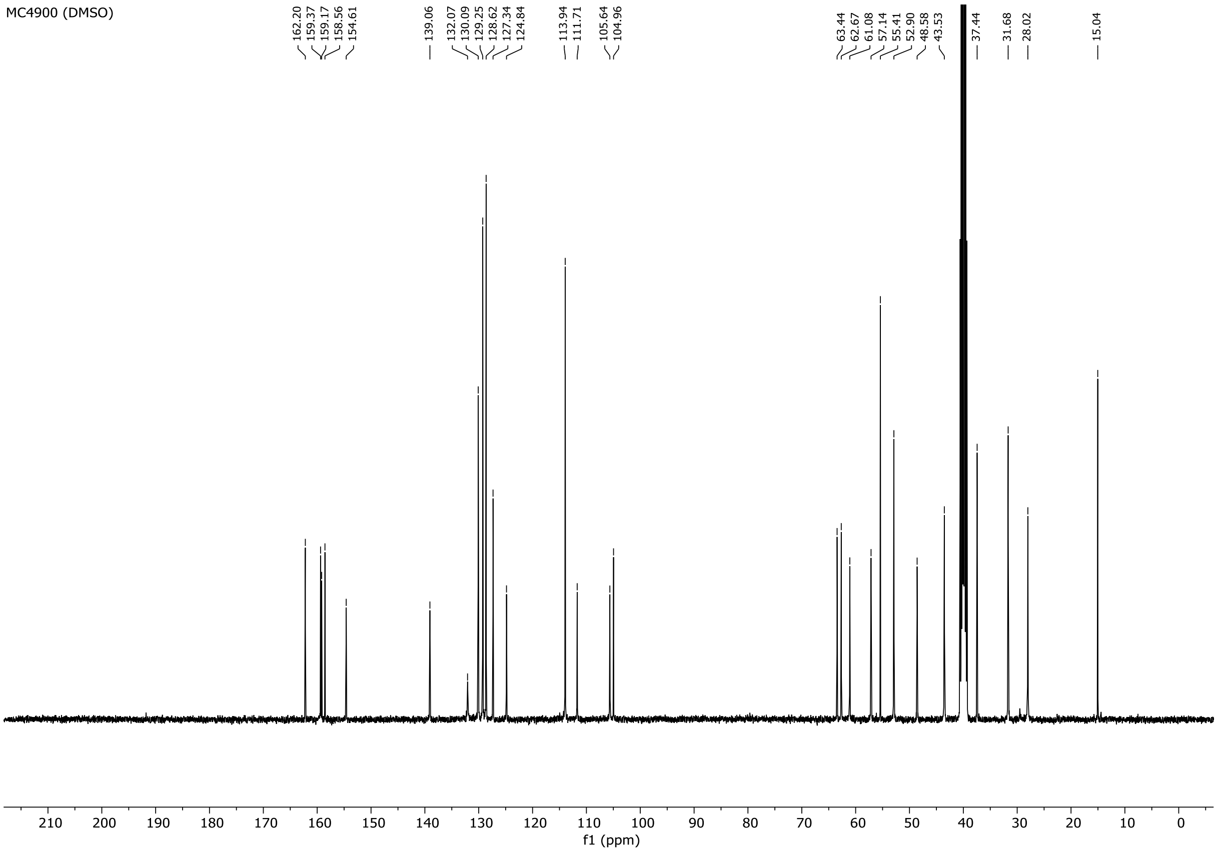

Figure S76: ^{13}C NMR spectrum of Compound **1t**.

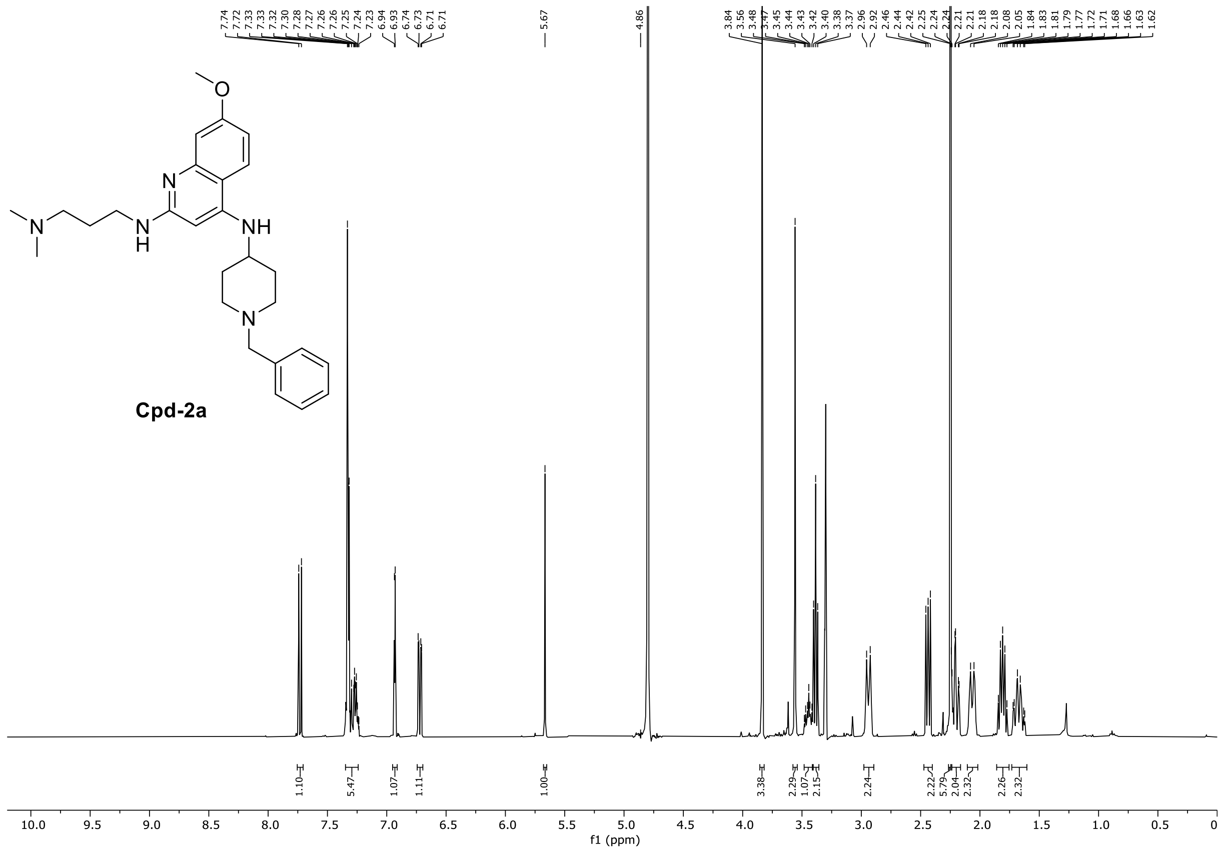

Figure S77: ^{1}H NMR spectrum of Compound **2a**.

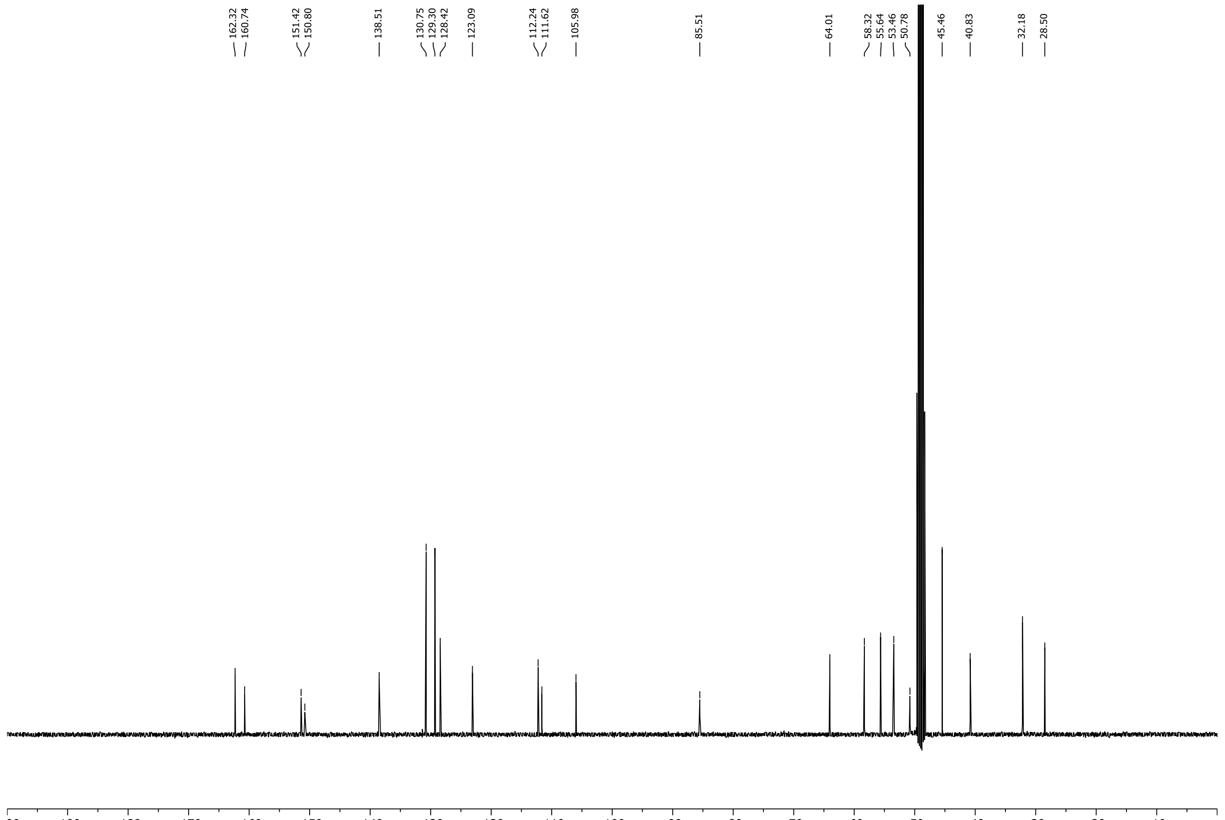

Figure S78: ^{13}C NMR spectrum of Compound **2a**.

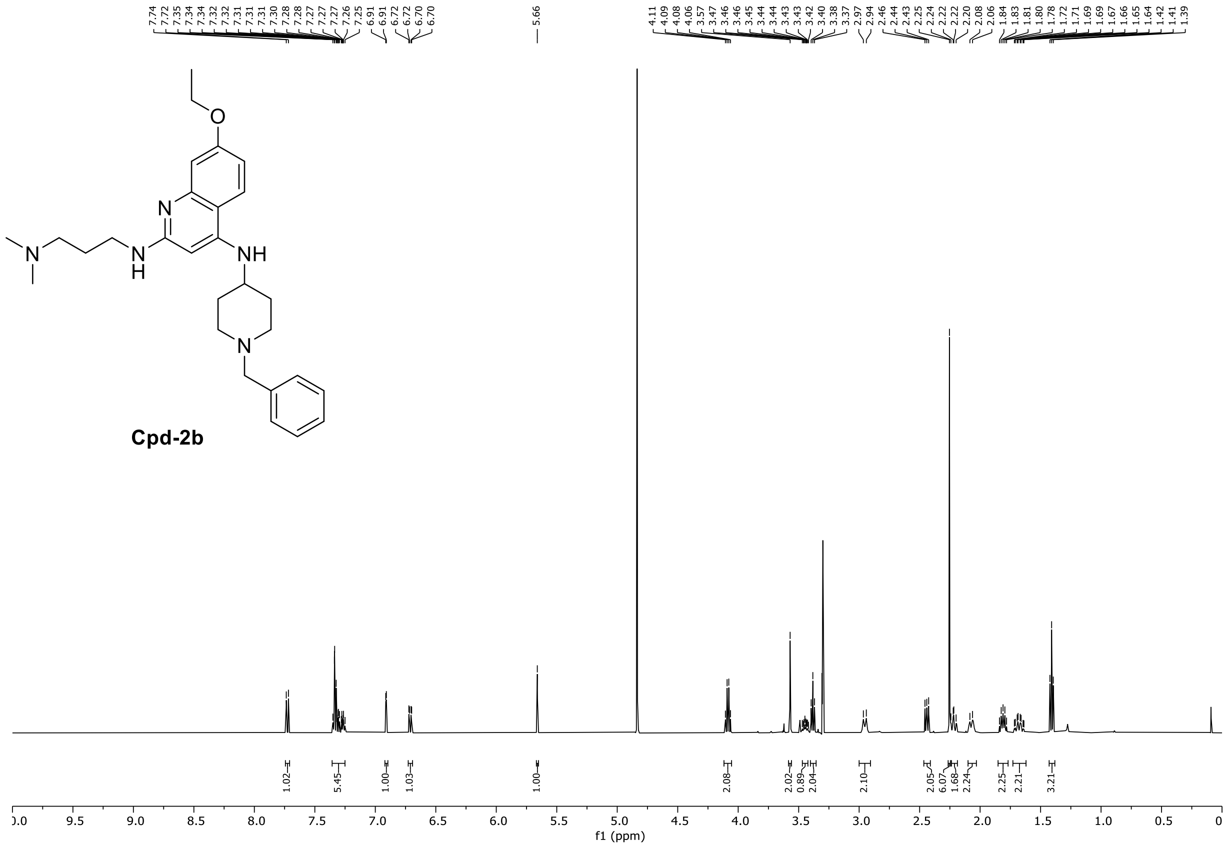

Figure S79: ^{1}H NMR spectrum of Compound **2b**.

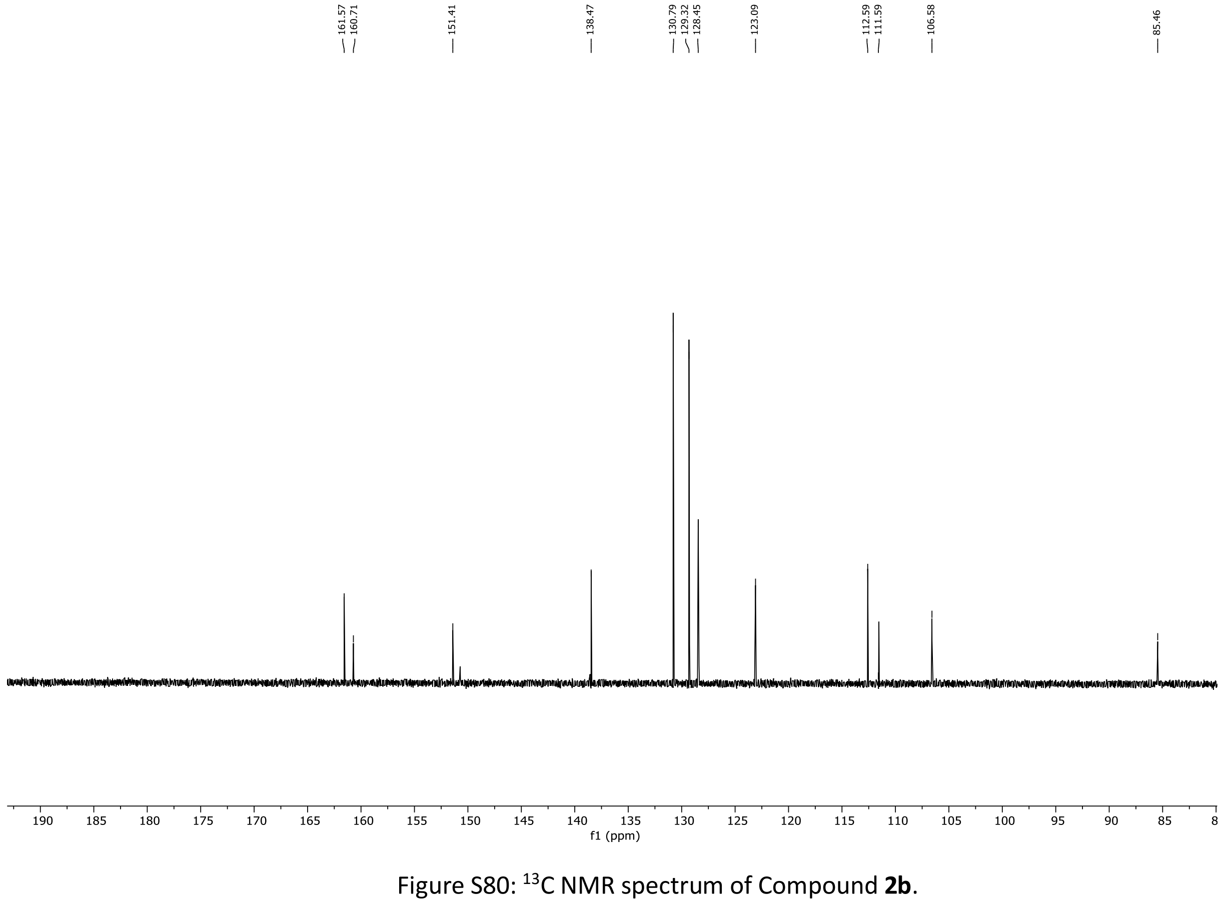

Figure S80: ^{13}C NMR spectrum of Compound **2b**.

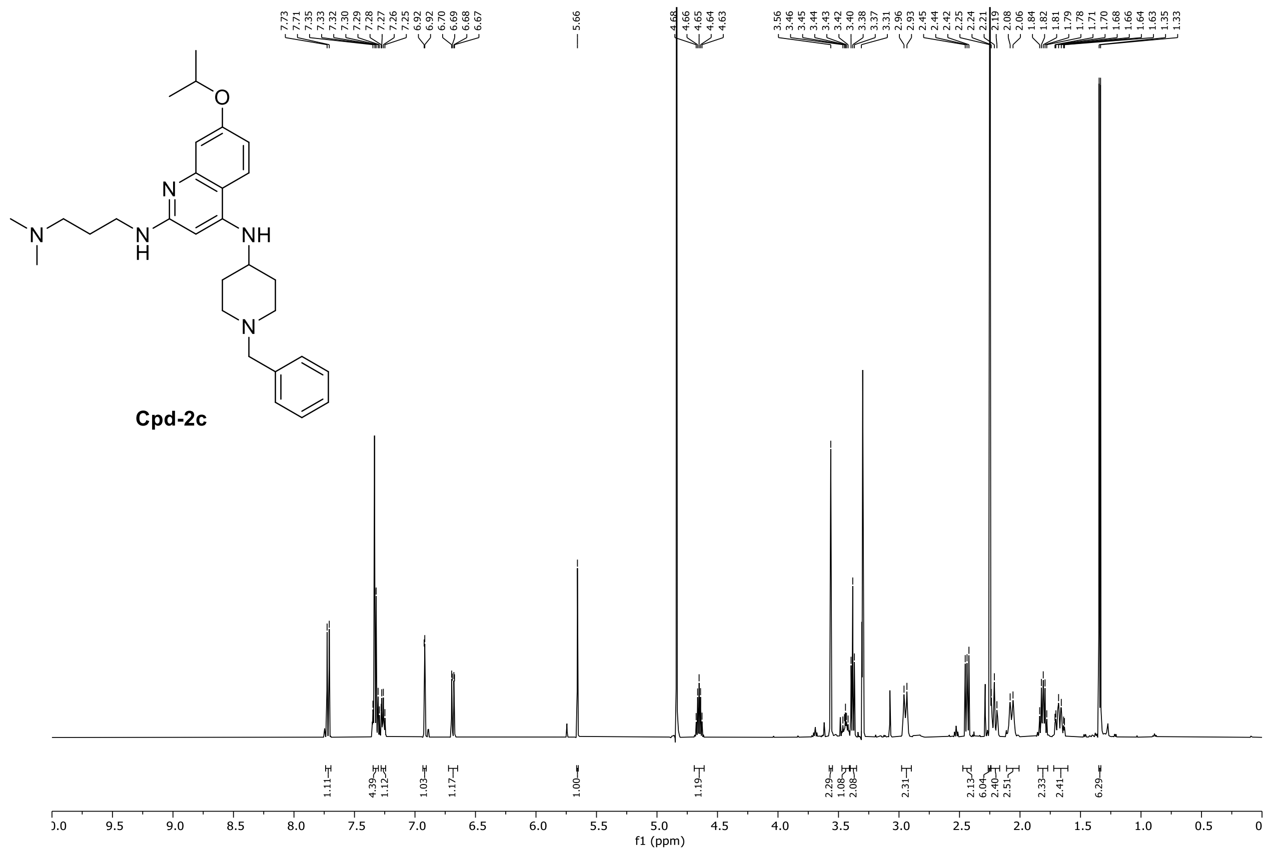

Figure S81: ^{1}H NMR spectrum of Compound **2c**.

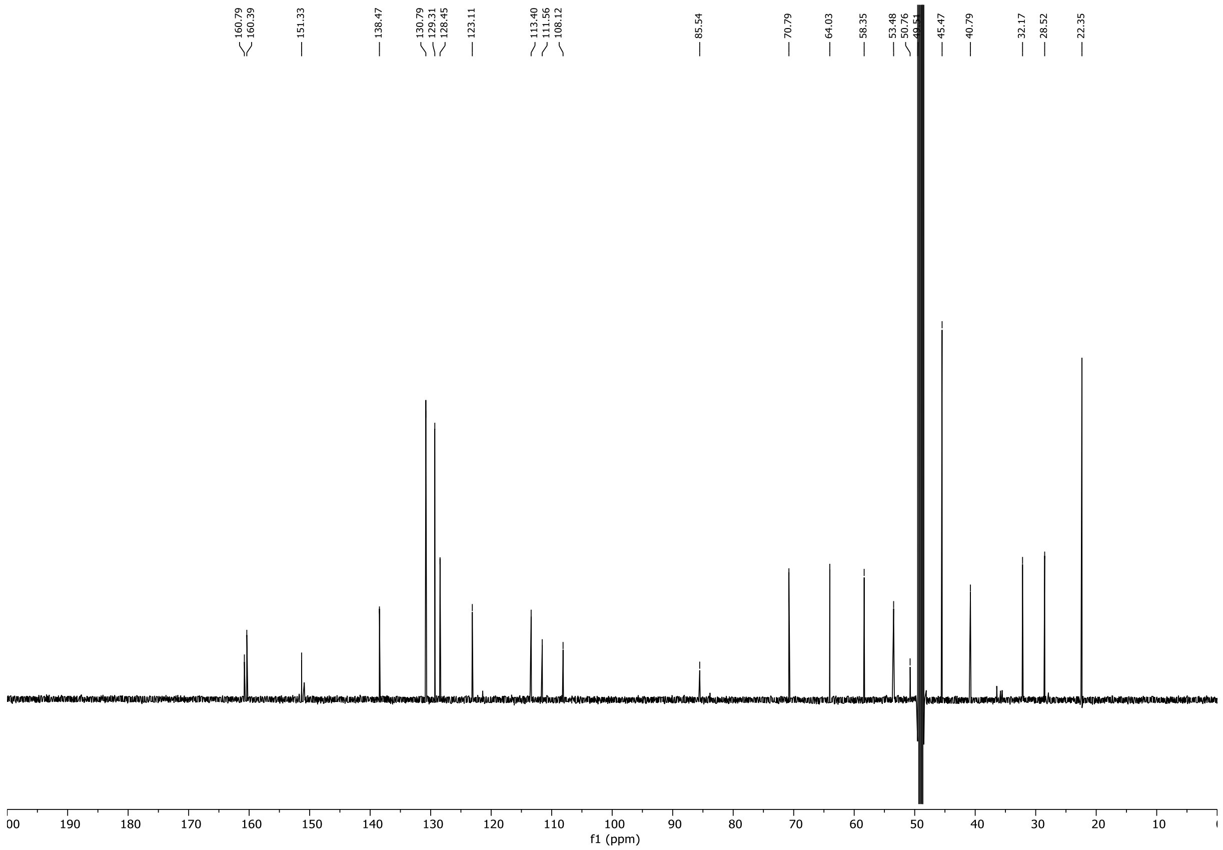

Figure S82: ^{13}C NMR spectrum of Compound **2c**.

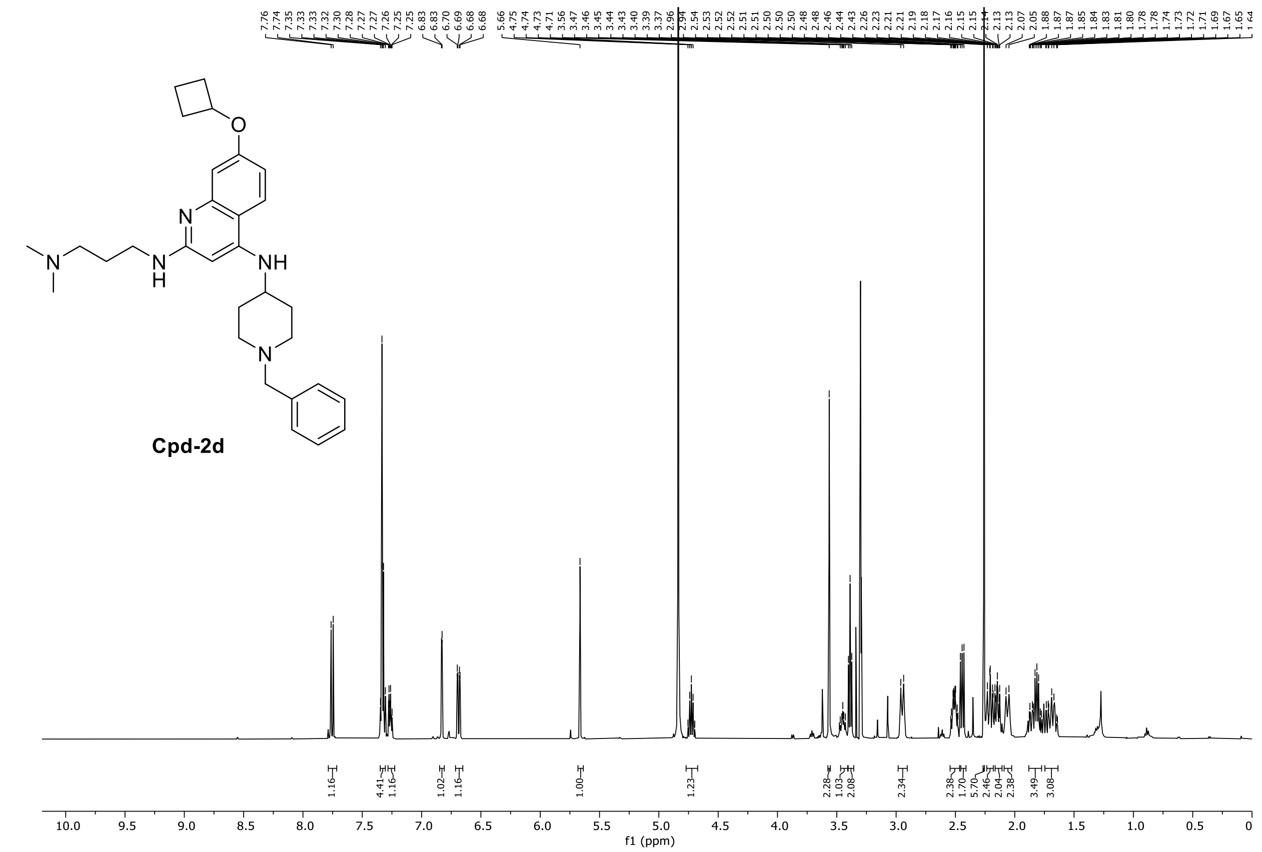

Figure S83: ^{1}H NMR spectrum of Compound **2d**.

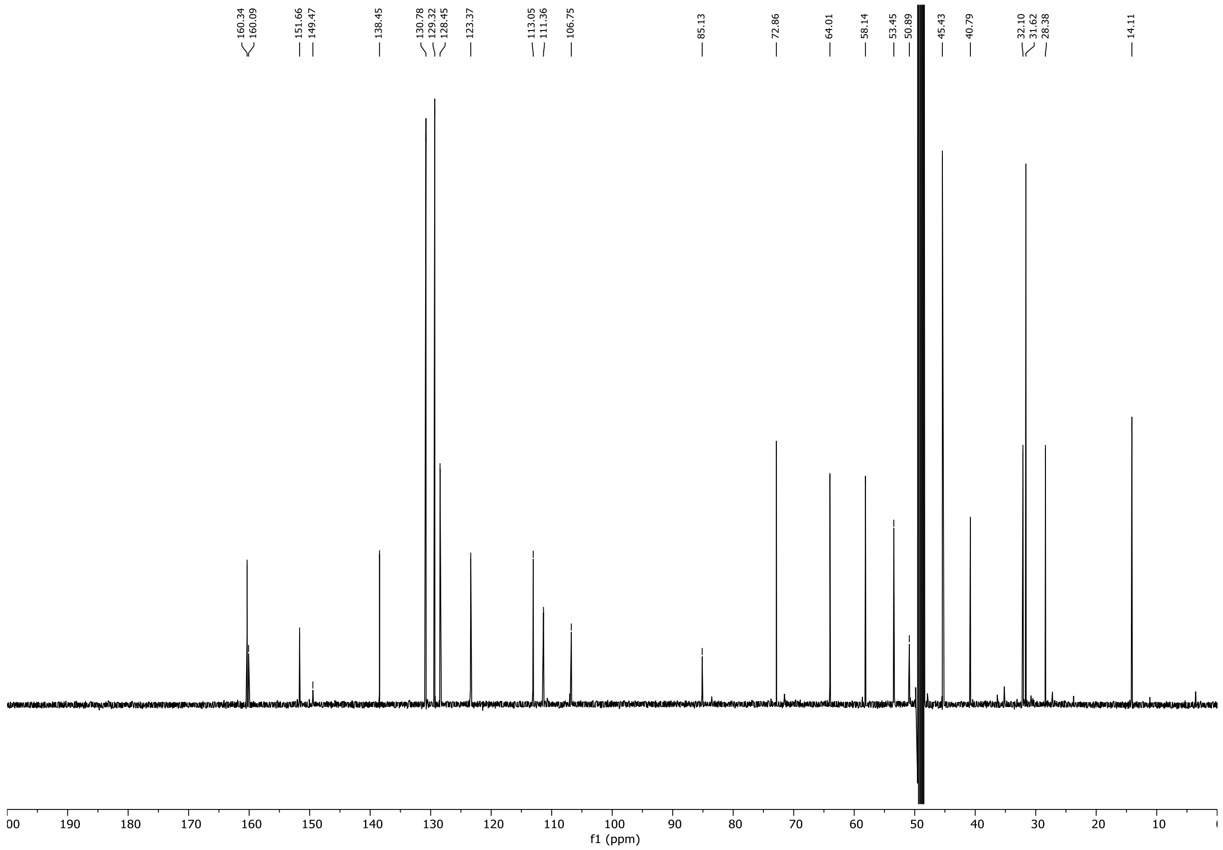

Figure S84: ^{13}C NMR spectrum of Compound **2d**.

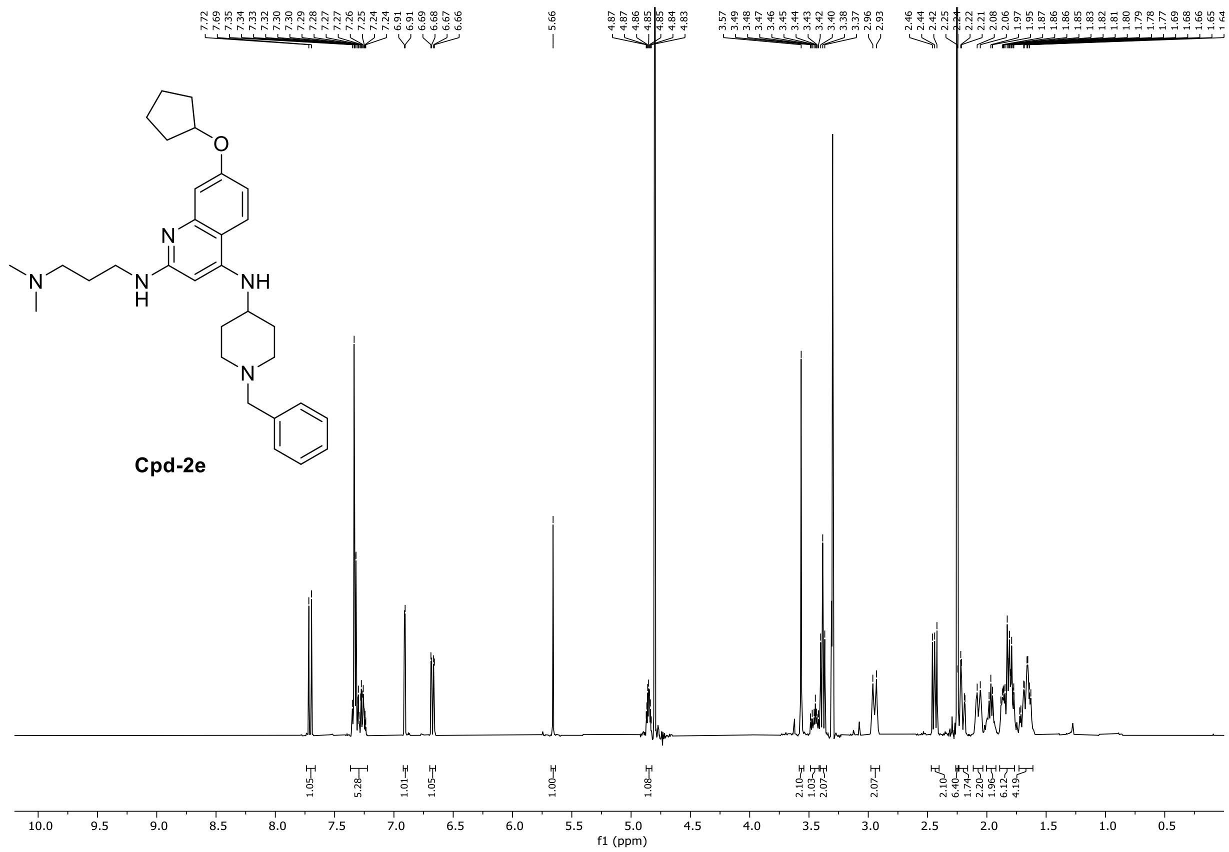

Figure S85: ^{1}H NMR spectrum of Compound **2e**.

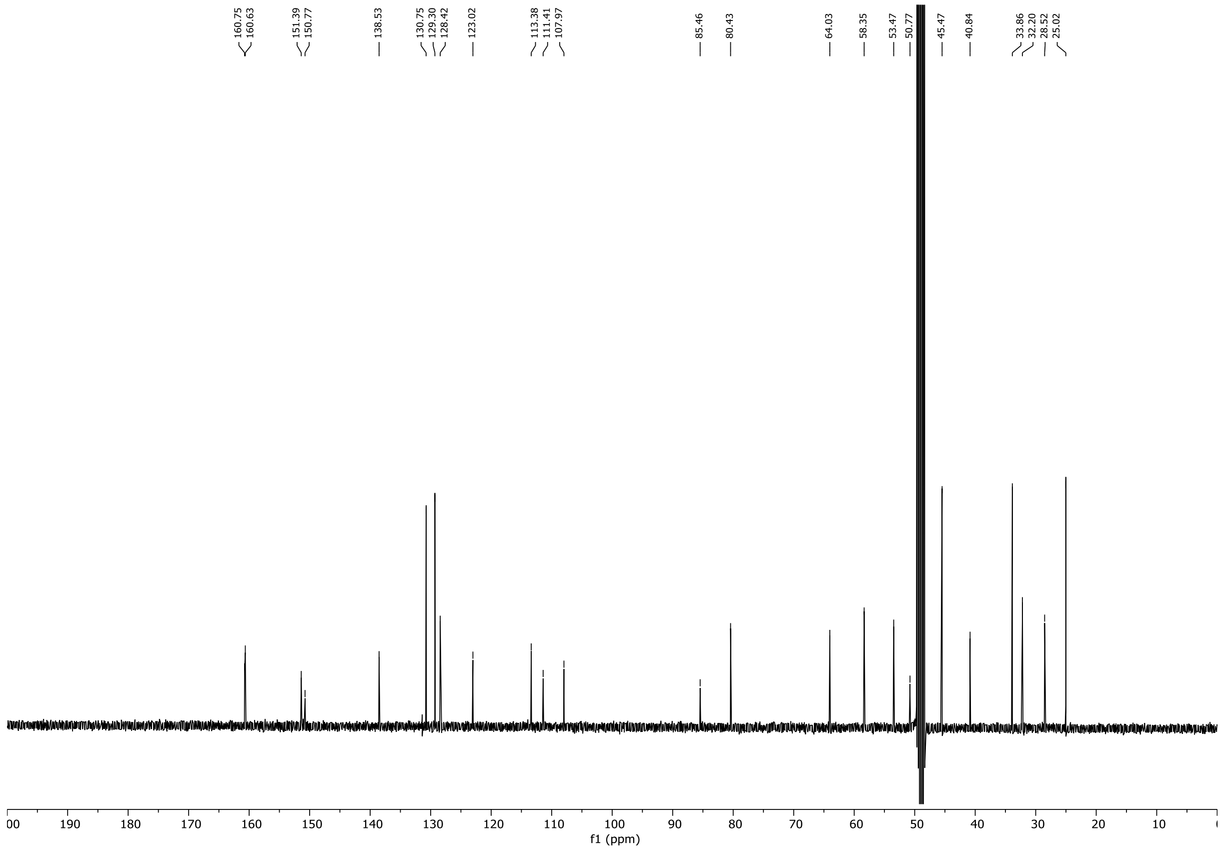

Figure S86: ^{13}C NMR spectrum of Compound **2e**.

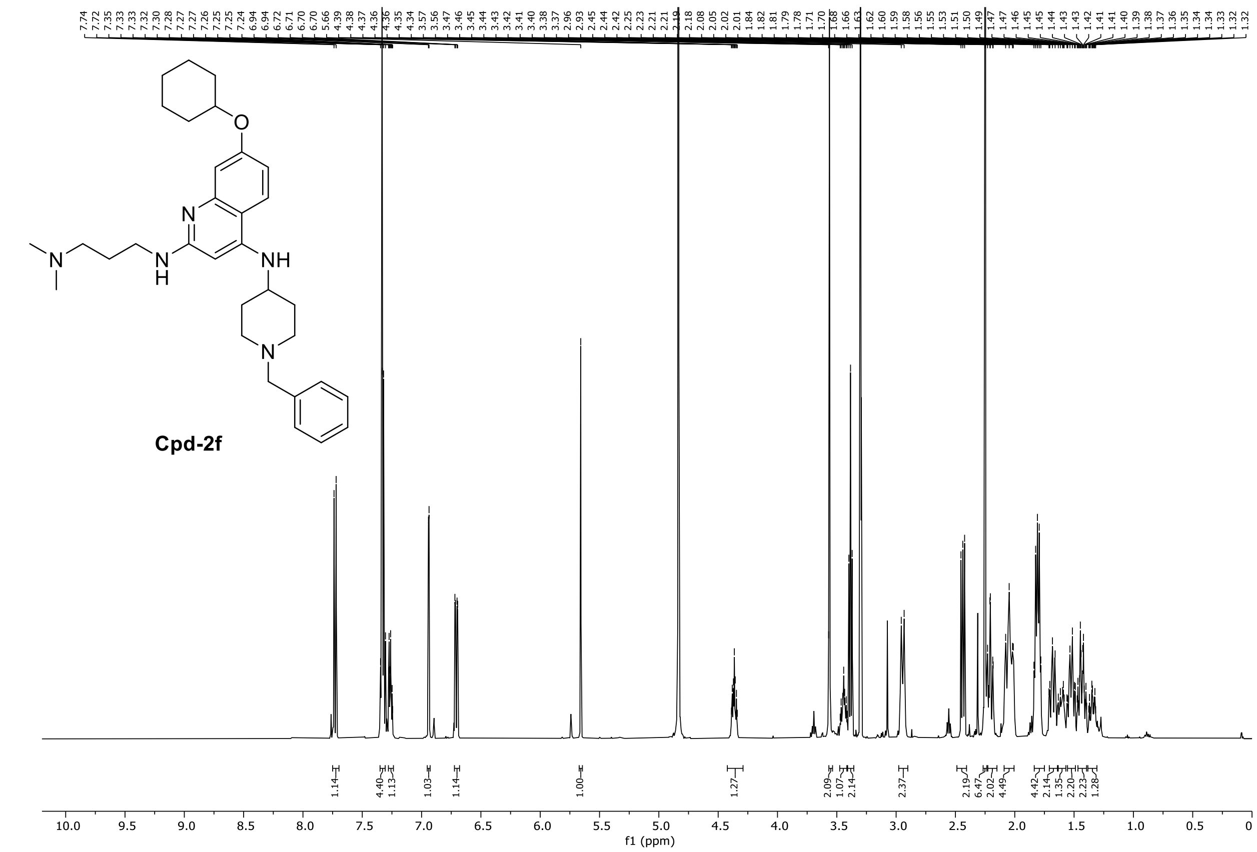

Figure S87: ^{1}H NMR spectrum of Compound **2f**.

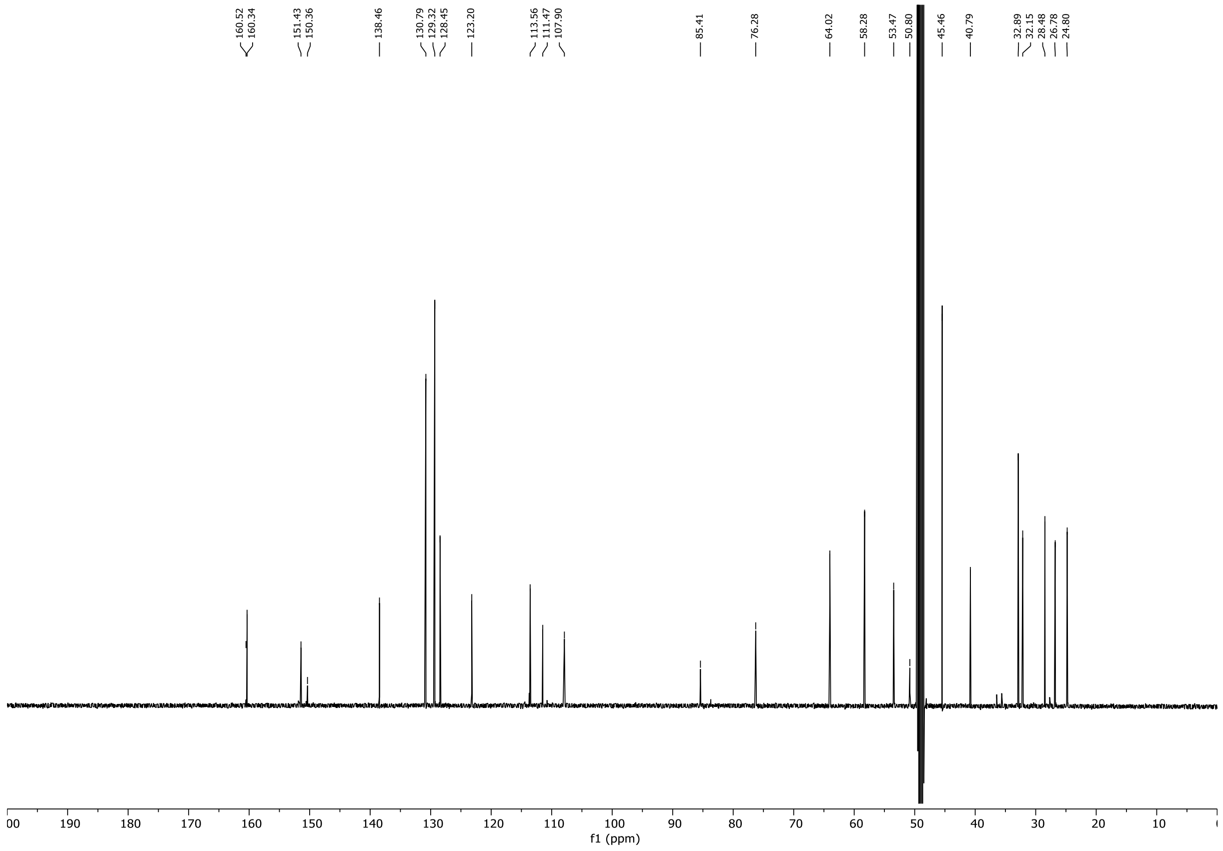

Figure S88: ^{13}C NMR spectrum of Compound **2f**.

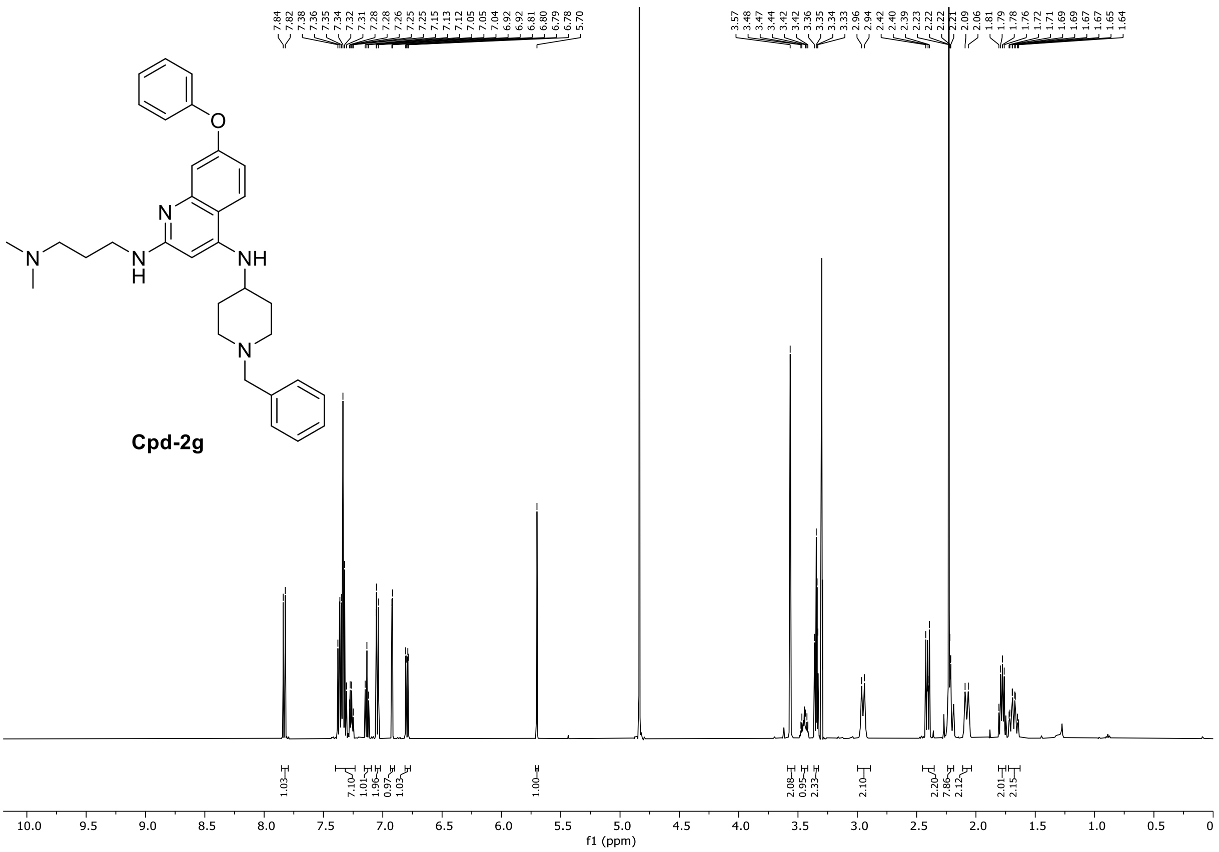

Figure S89: ^{1}H NMR spectrum of Compound **2g**.

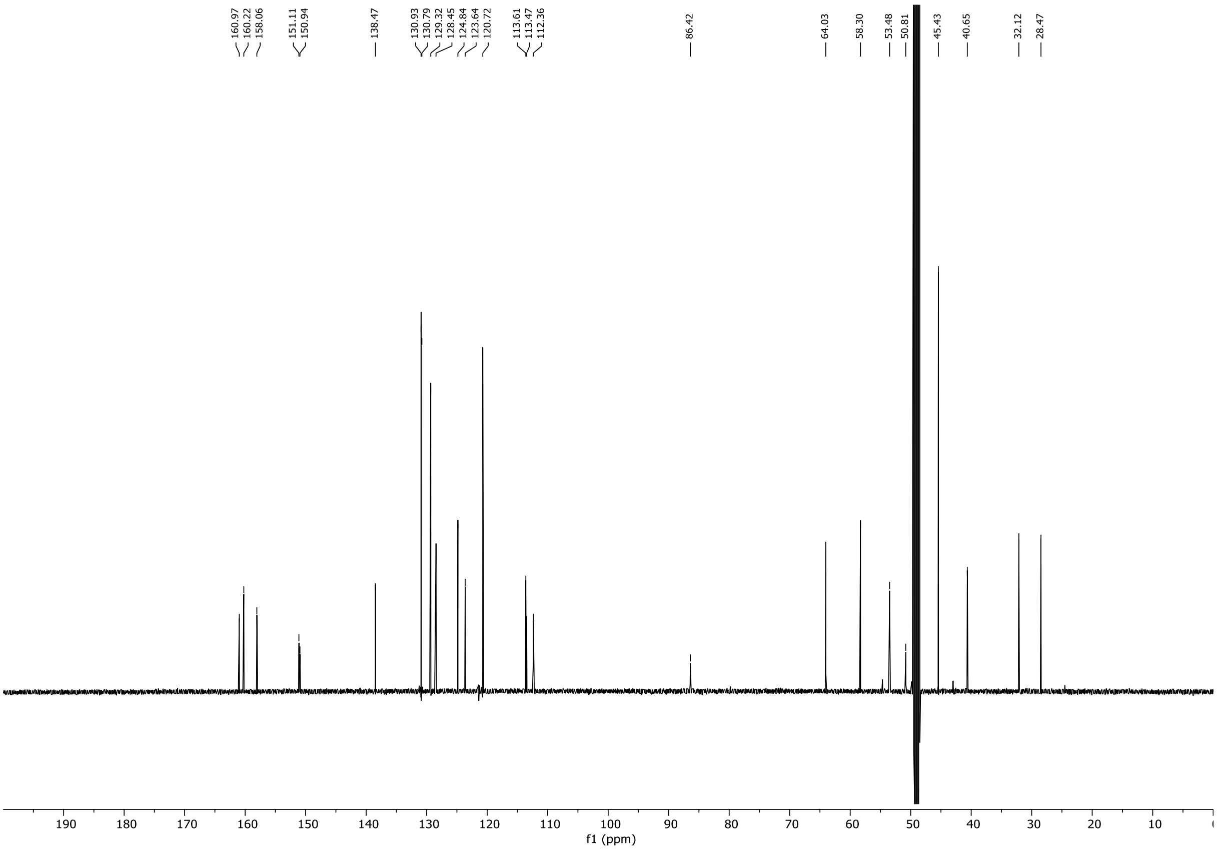

Figure S90: ^{13}C NMR spectrum of Compound **2g**.

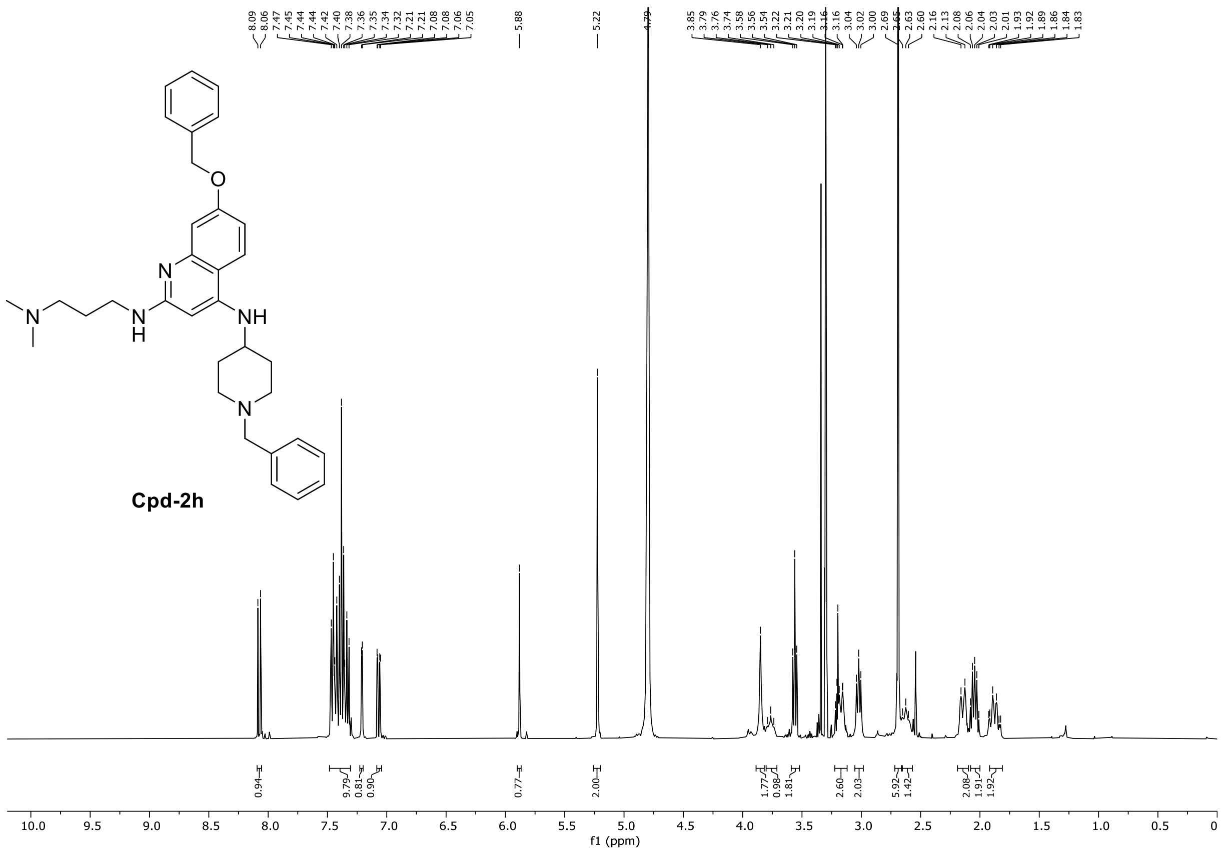

Figure S91: ^{1}H NMR spectrum of Compound **2h**.

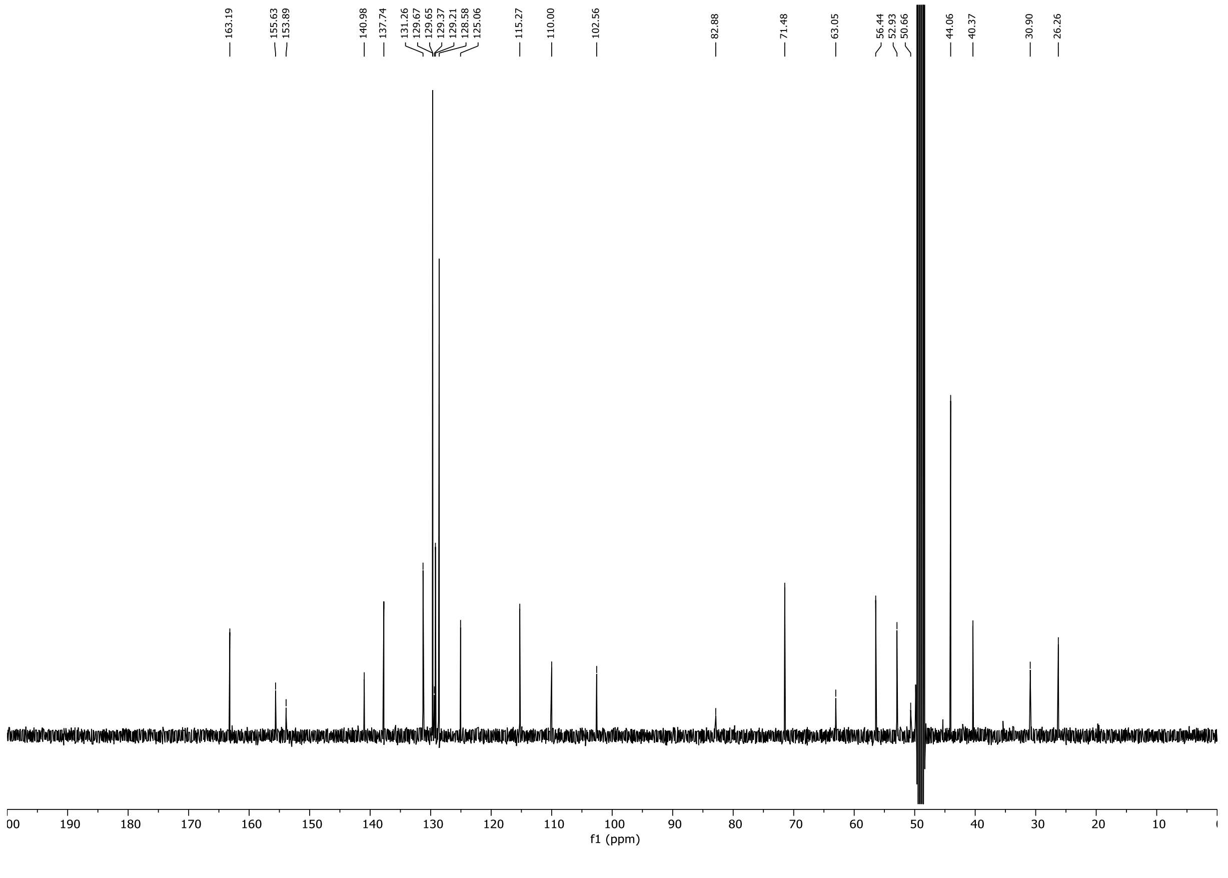

Figure S92: ^{13}C NMR spectrum of Compound **2h**.

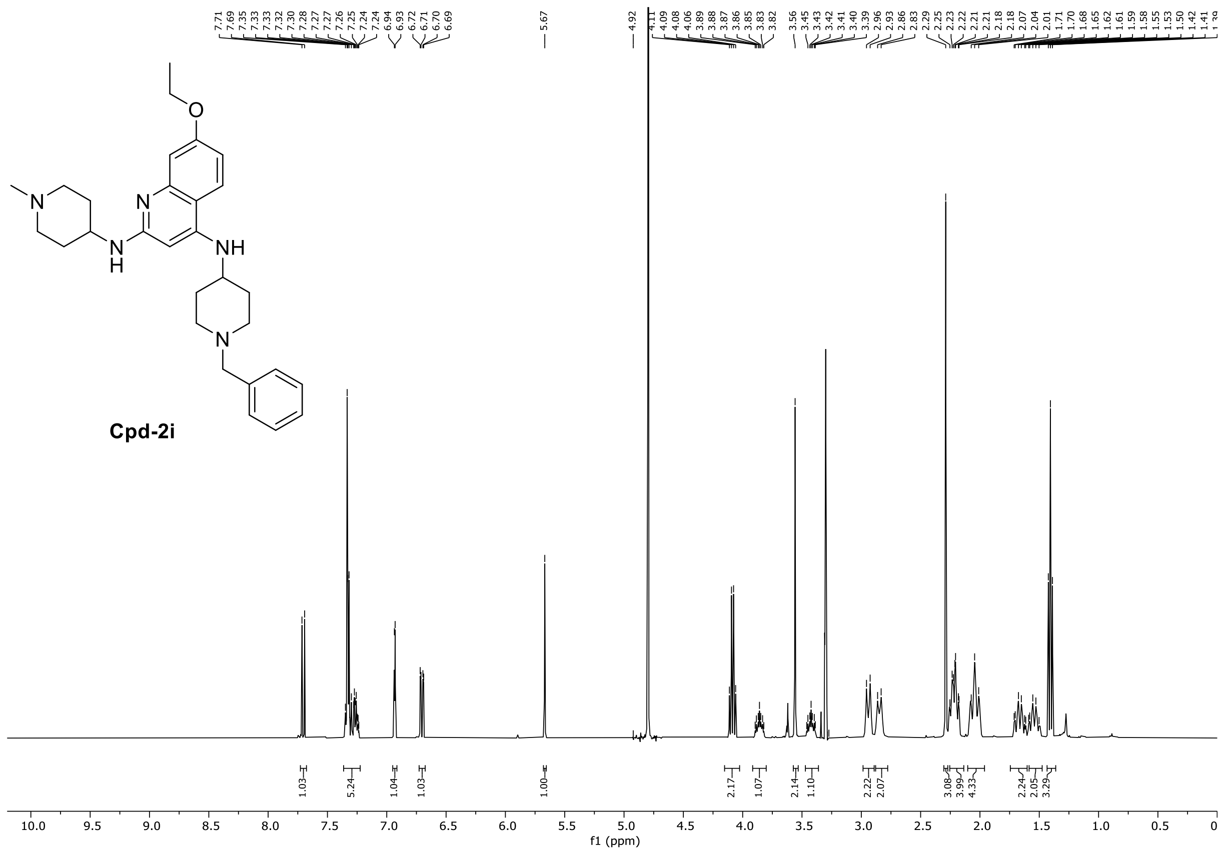

Figure S93: ^{1}H NMR spectrum of Compound **2i**.

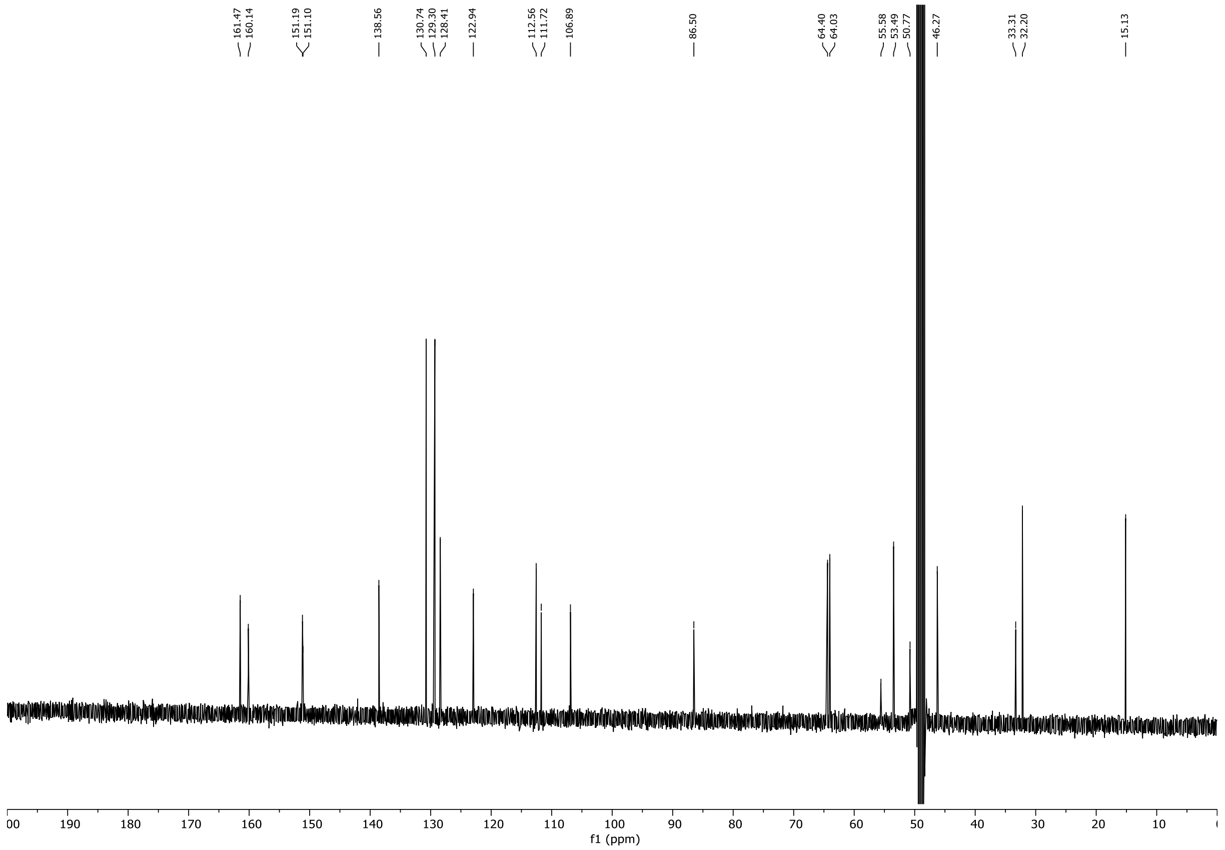

Figure S94: ^{13}C NMR spectrum of Compound **2i**.

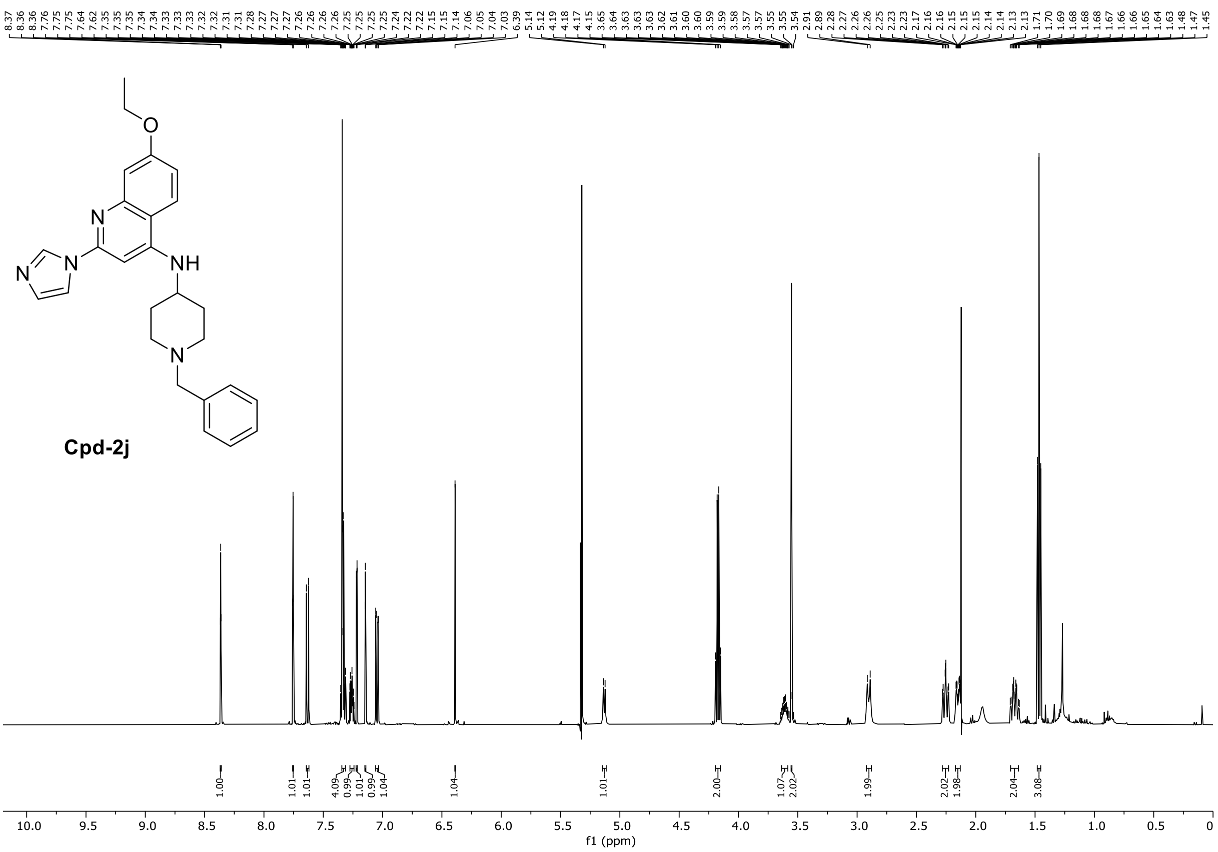

Figure S95: ^{1}H NMR spectrum of Compound **2j**.

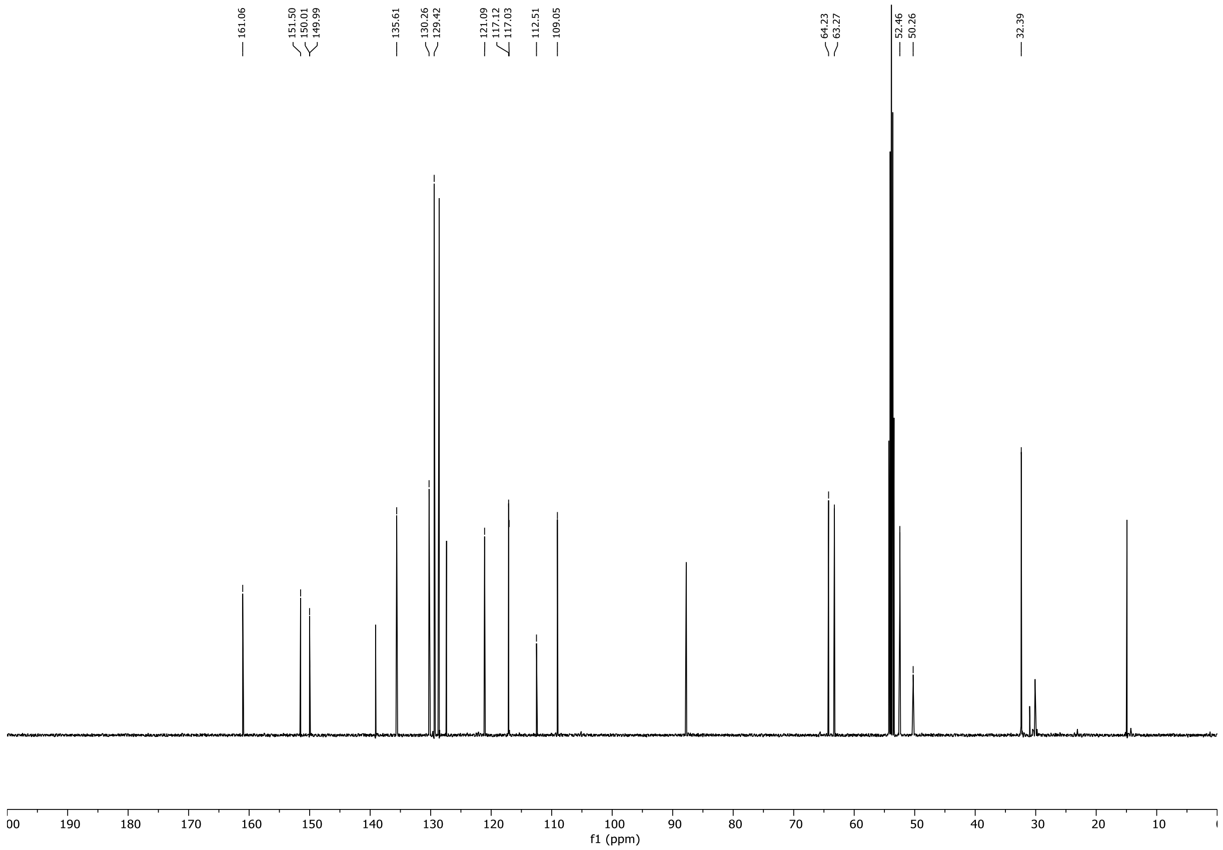

Figure S96: ^{13}C NMR spectrum of Compound **2j**.

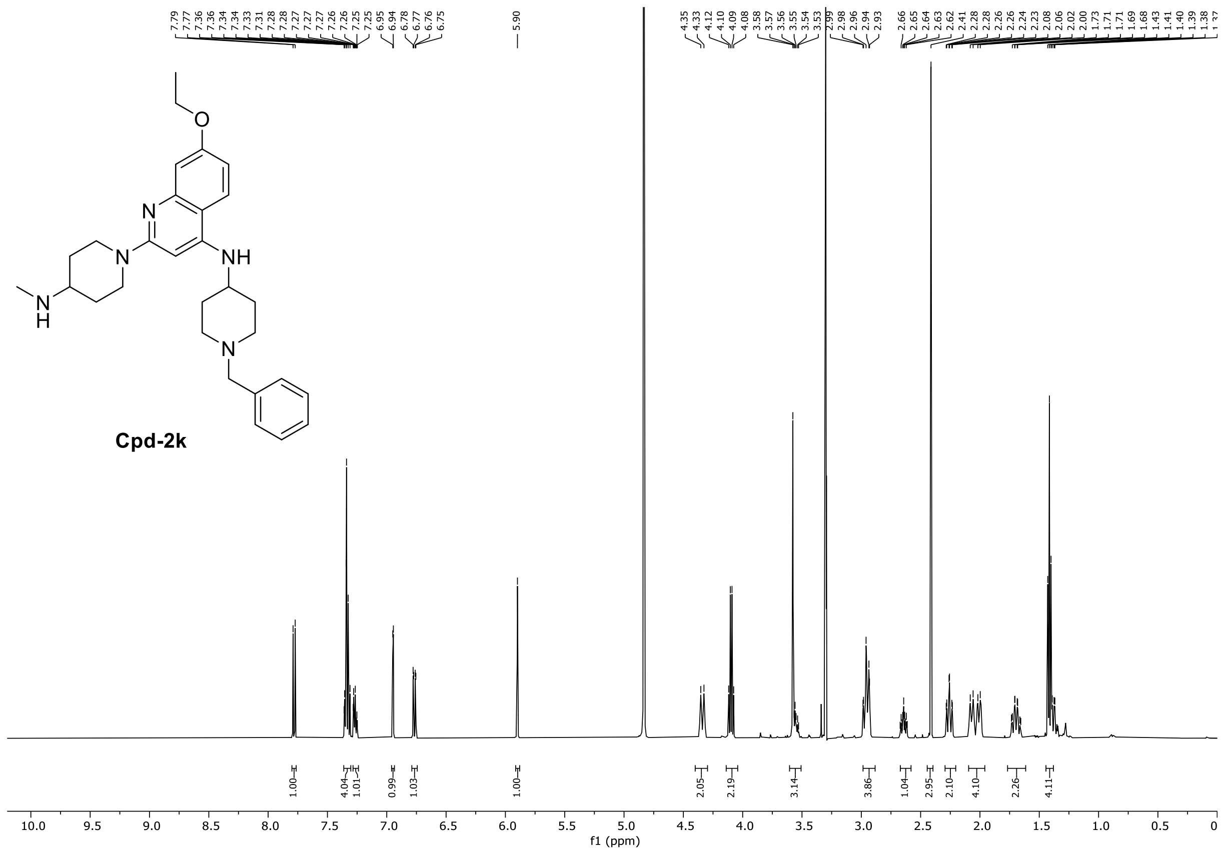

Figure S97: ^{1}H NMR spectrum of Compound **2k**.

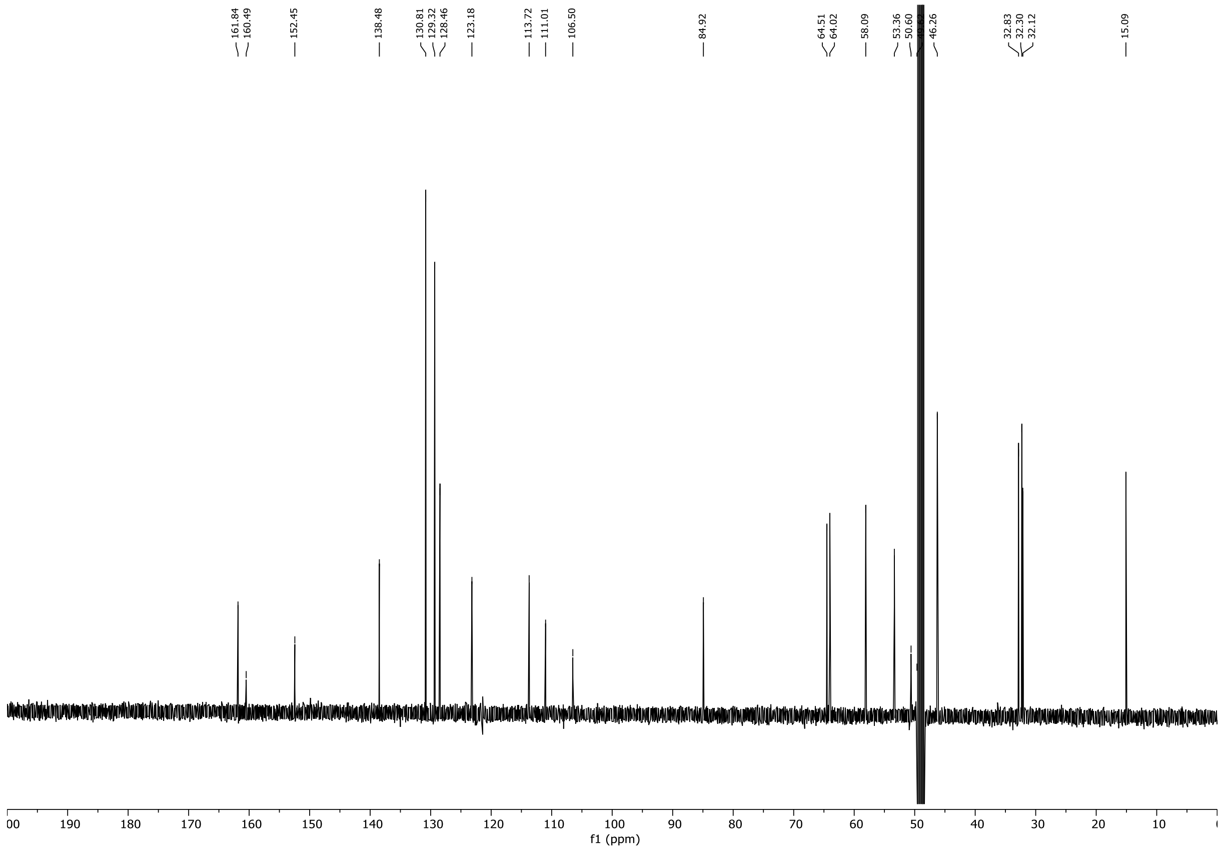

Figure S98: ^{13}C NMR spectrum of Compound **2k**.

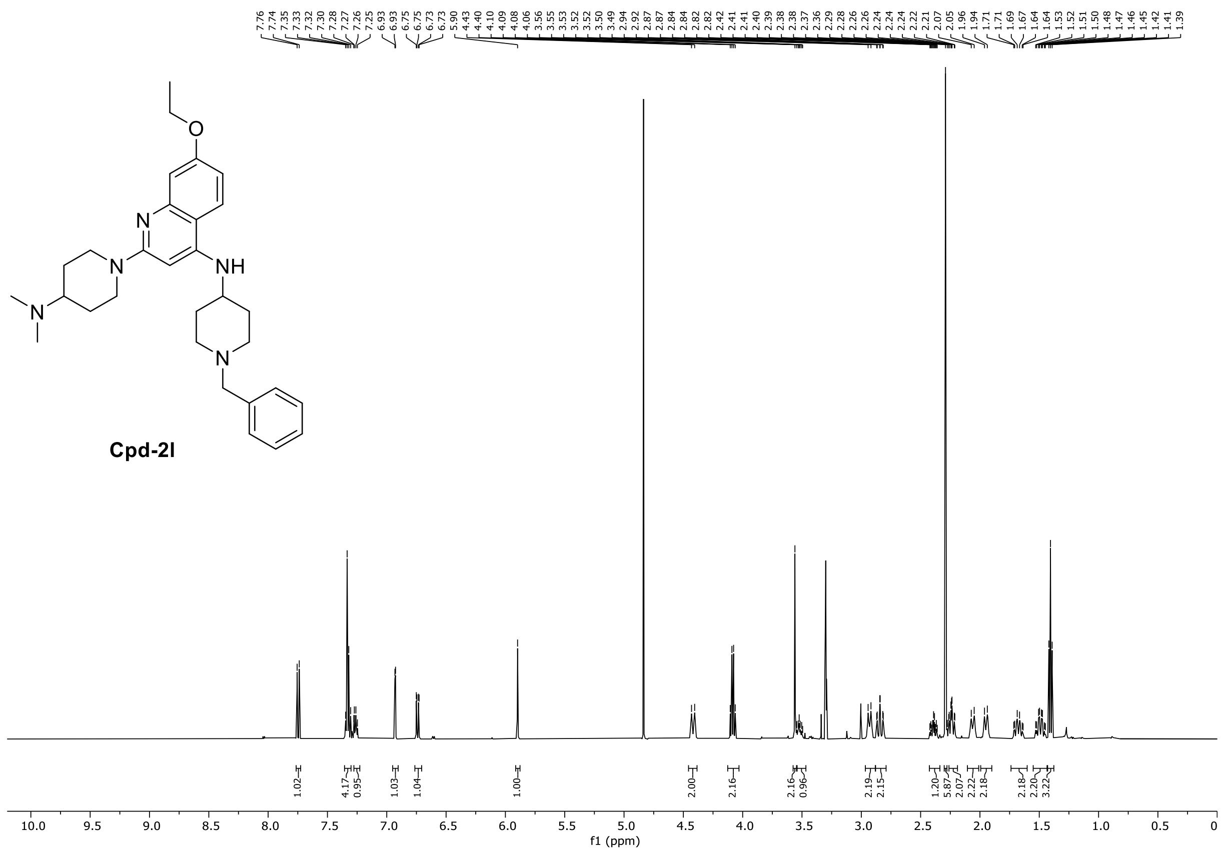

Figure S99: ^{1}H NMR spectrum of Compound **2l**.

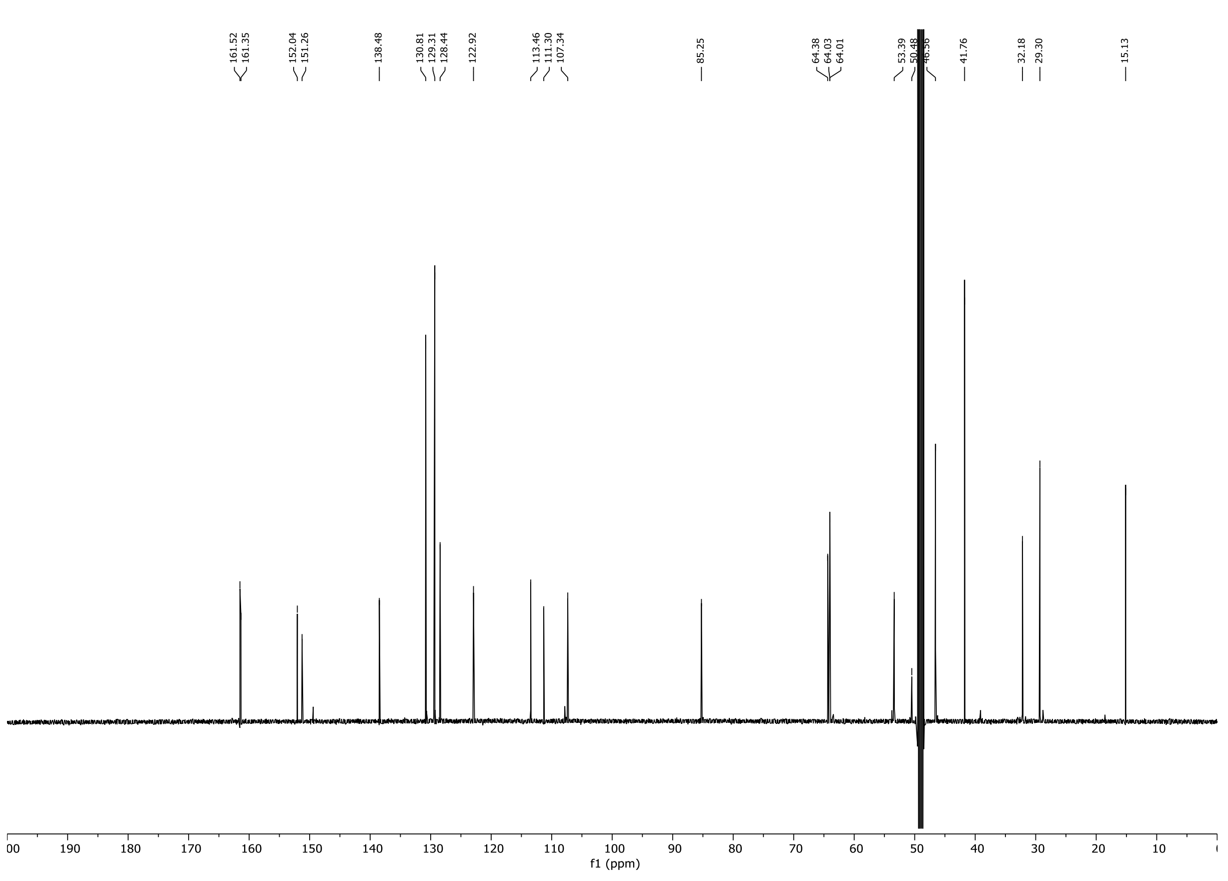

Figure S100: ^{13}C NMR spectrum of Compound **2l**.

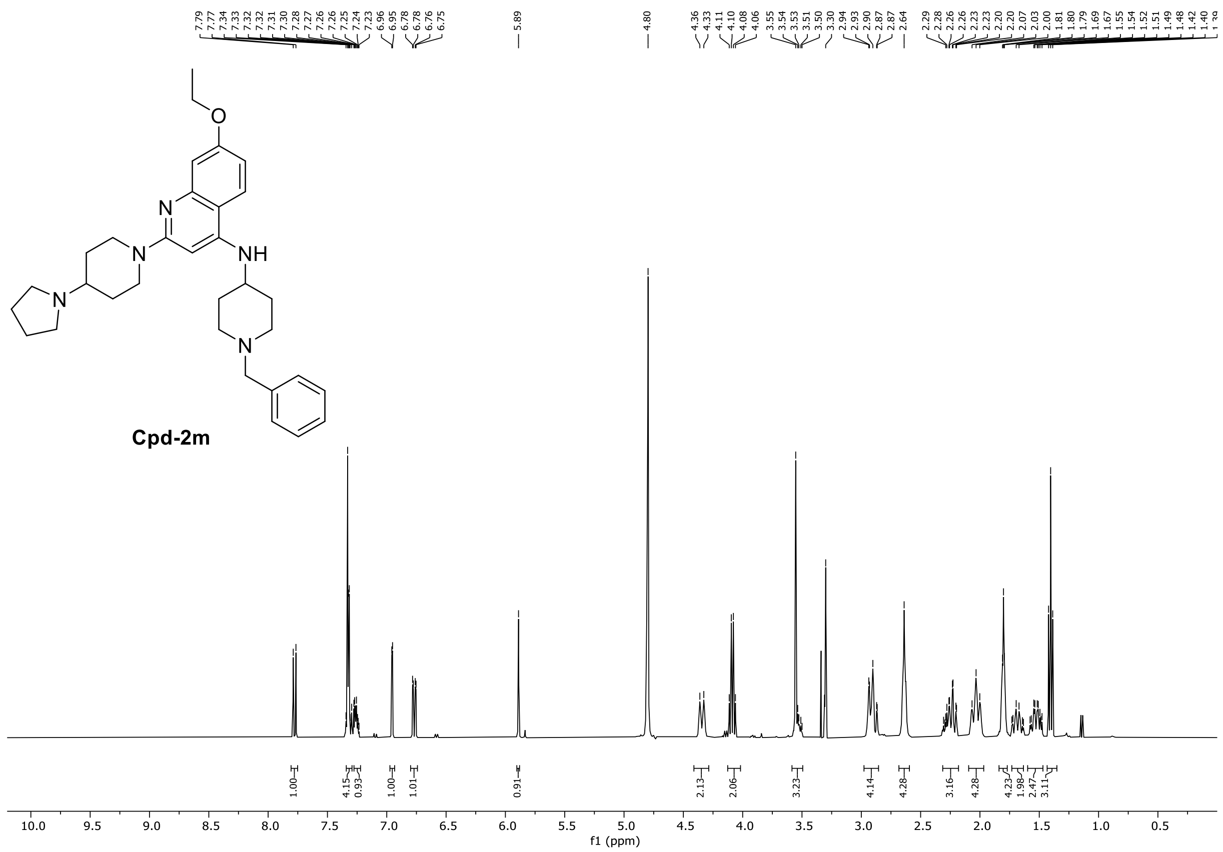

Figure S101: ^{1}H NMR spectrum of Compound **2m**.

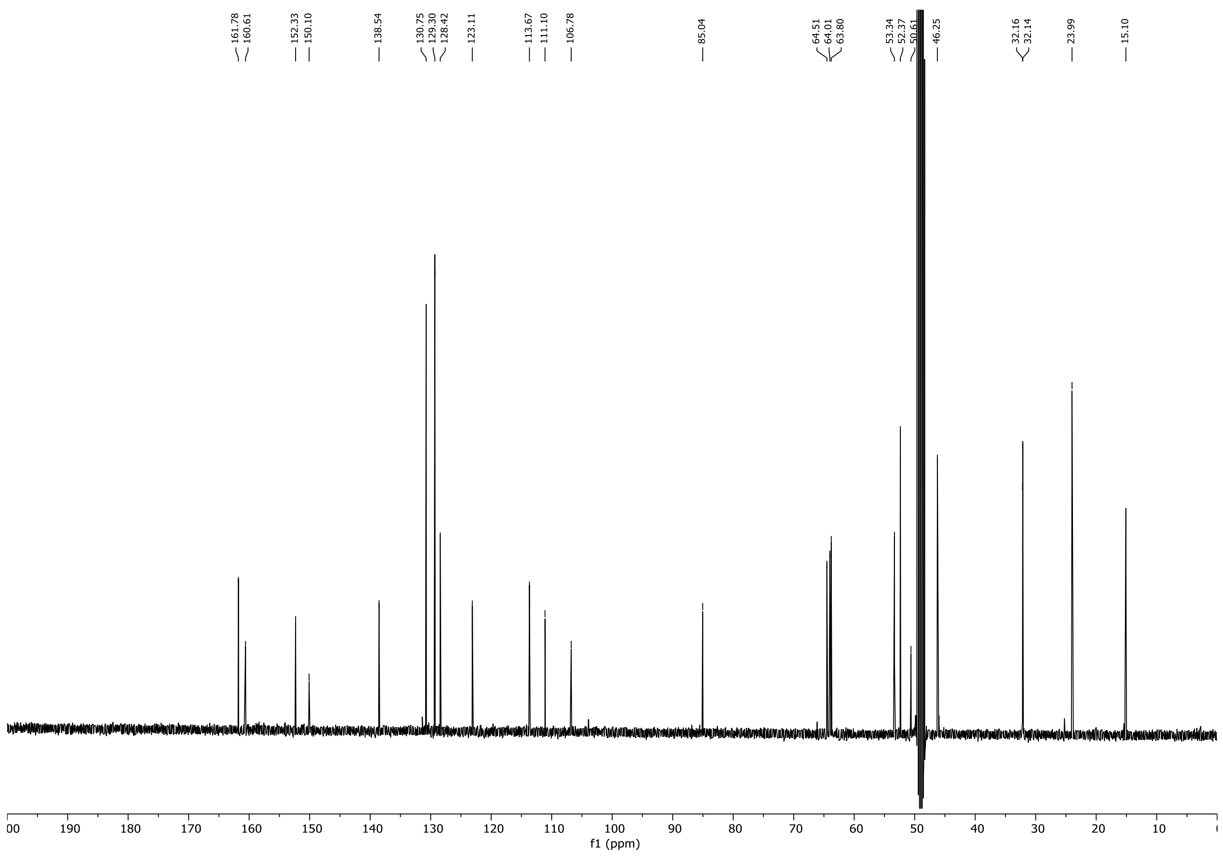

Figure 102: ^{13}C NMR spectrum of Compound **2m**.

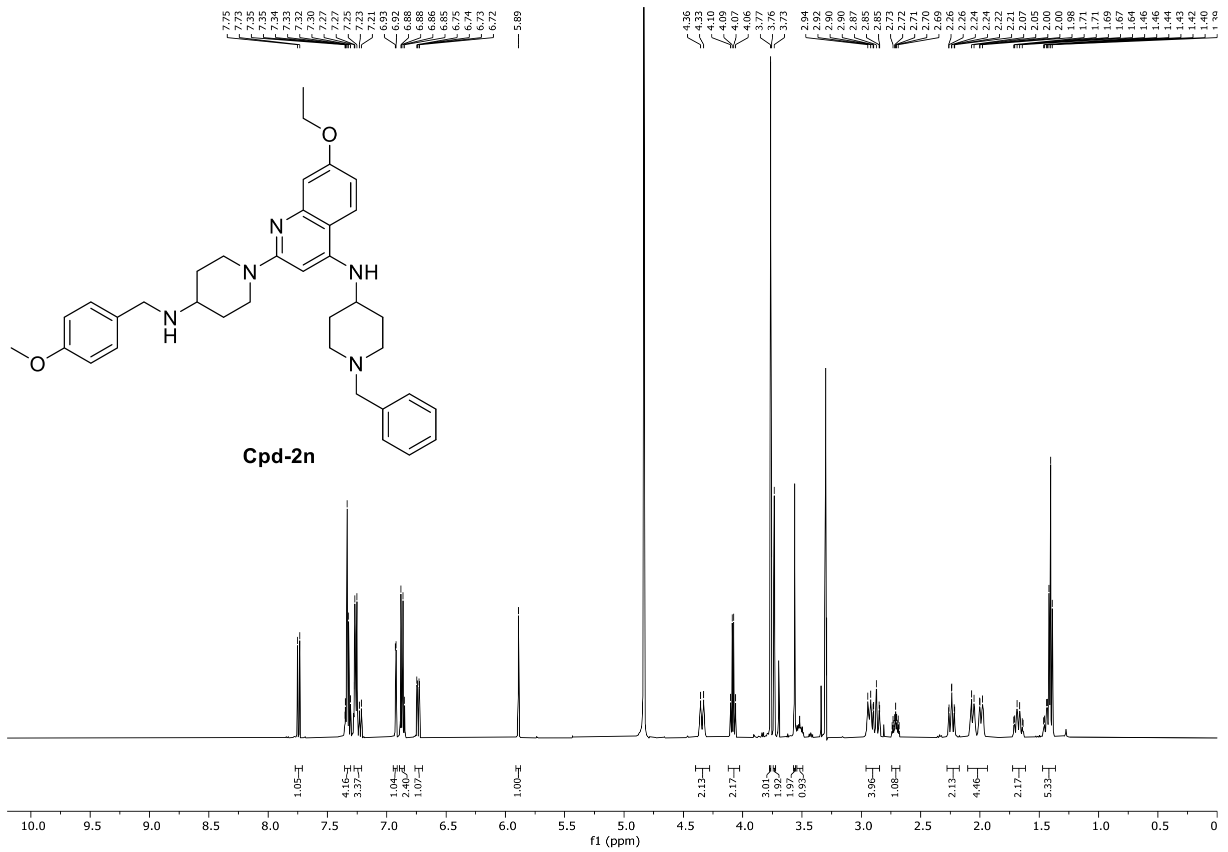

Figure S103: ^{1}H NMR spectrum of Compound **2n**.

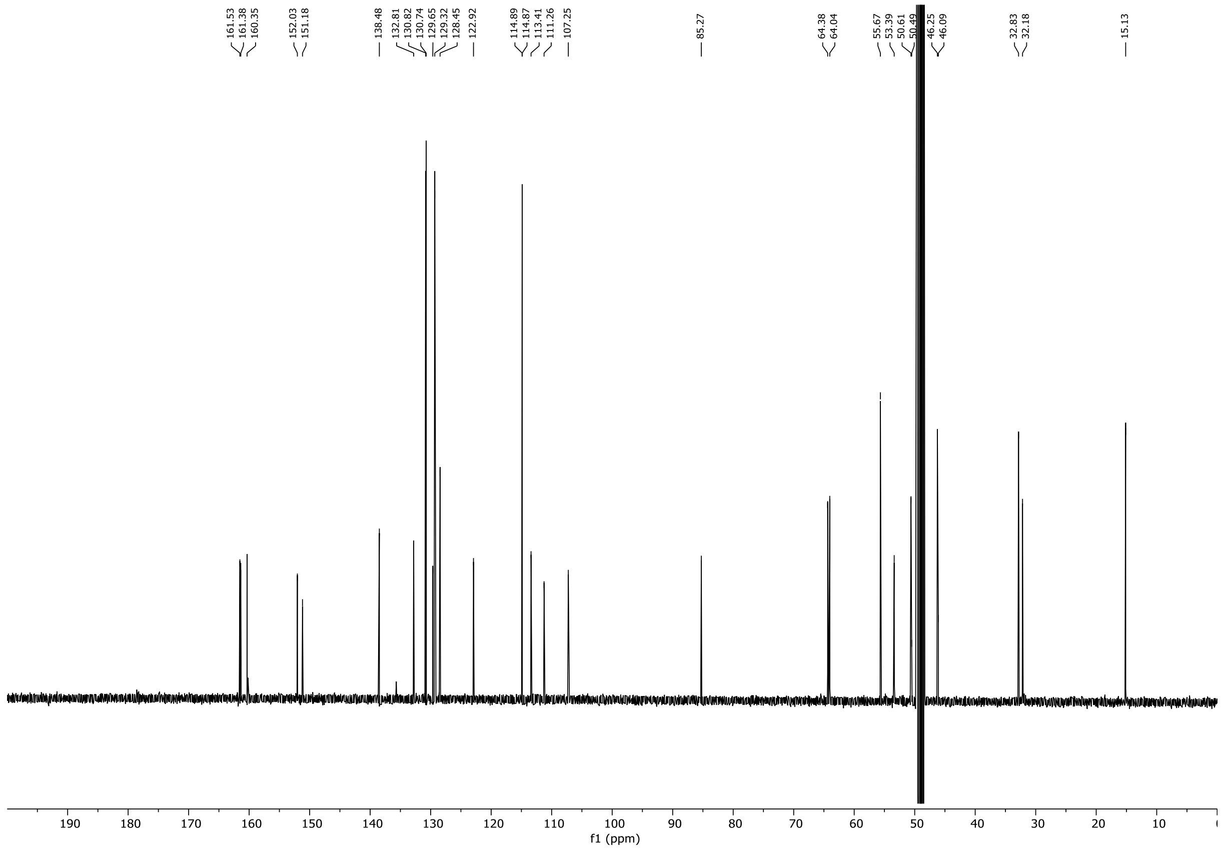

Figure S104: ^{13}C NMR spectrum of Compound **2n**.

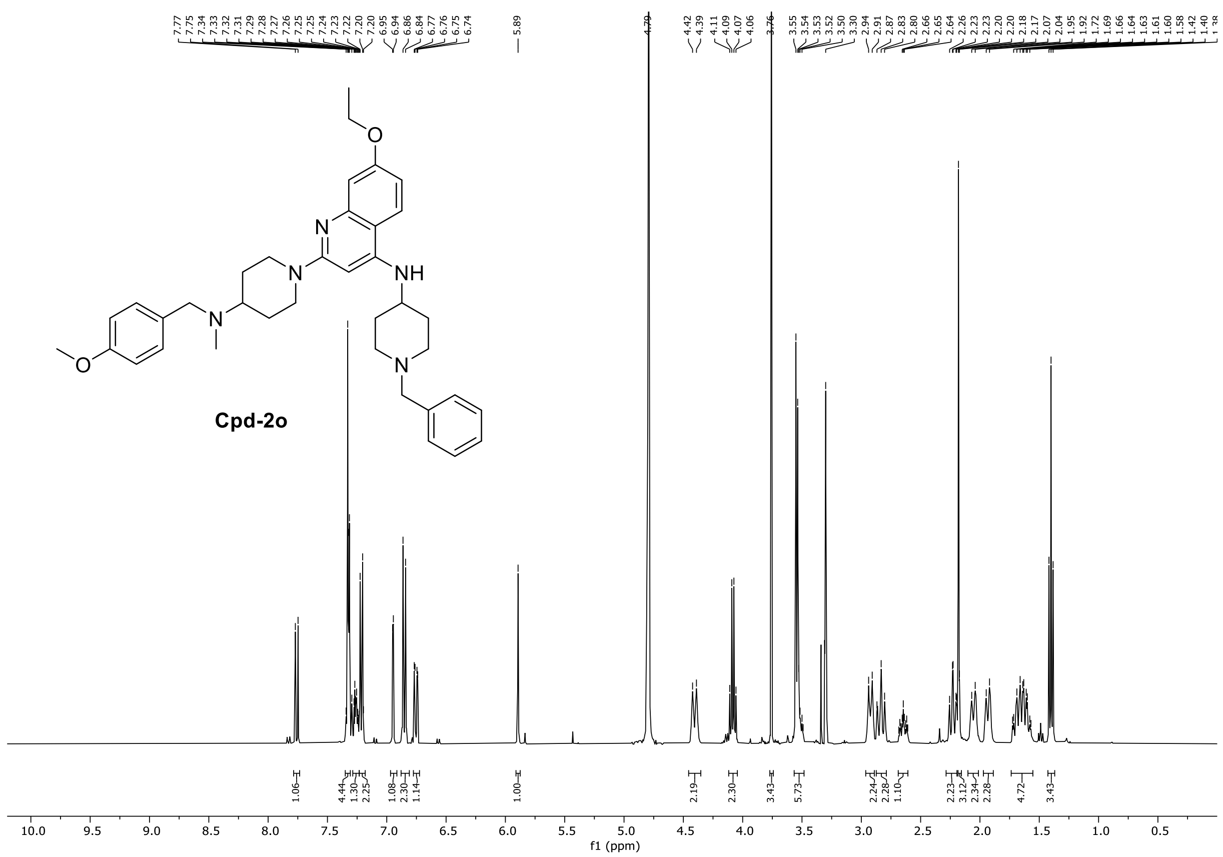

Figure S105: ^{1}H NMR spectrum of Compound **2o**.

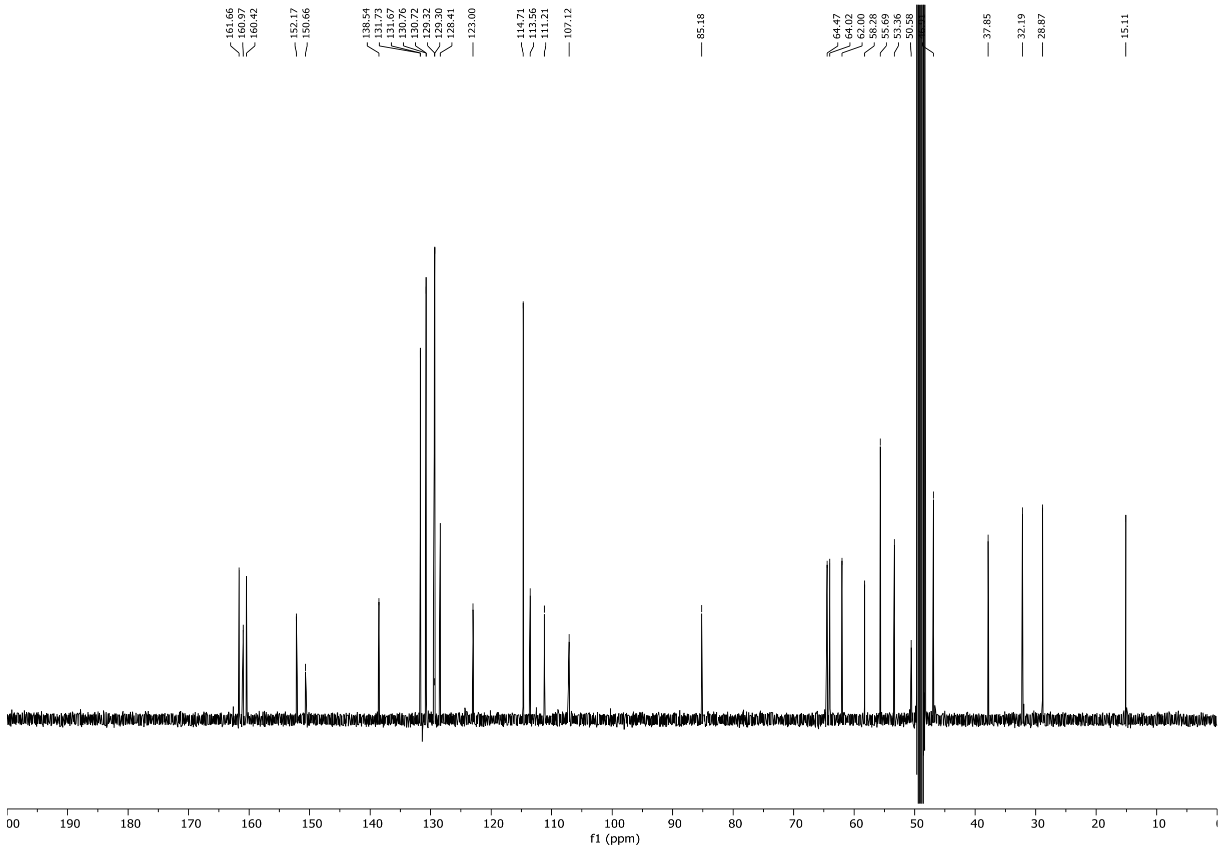

Figure S106: ^{13}C NMR spectrum of Compound **2o**.

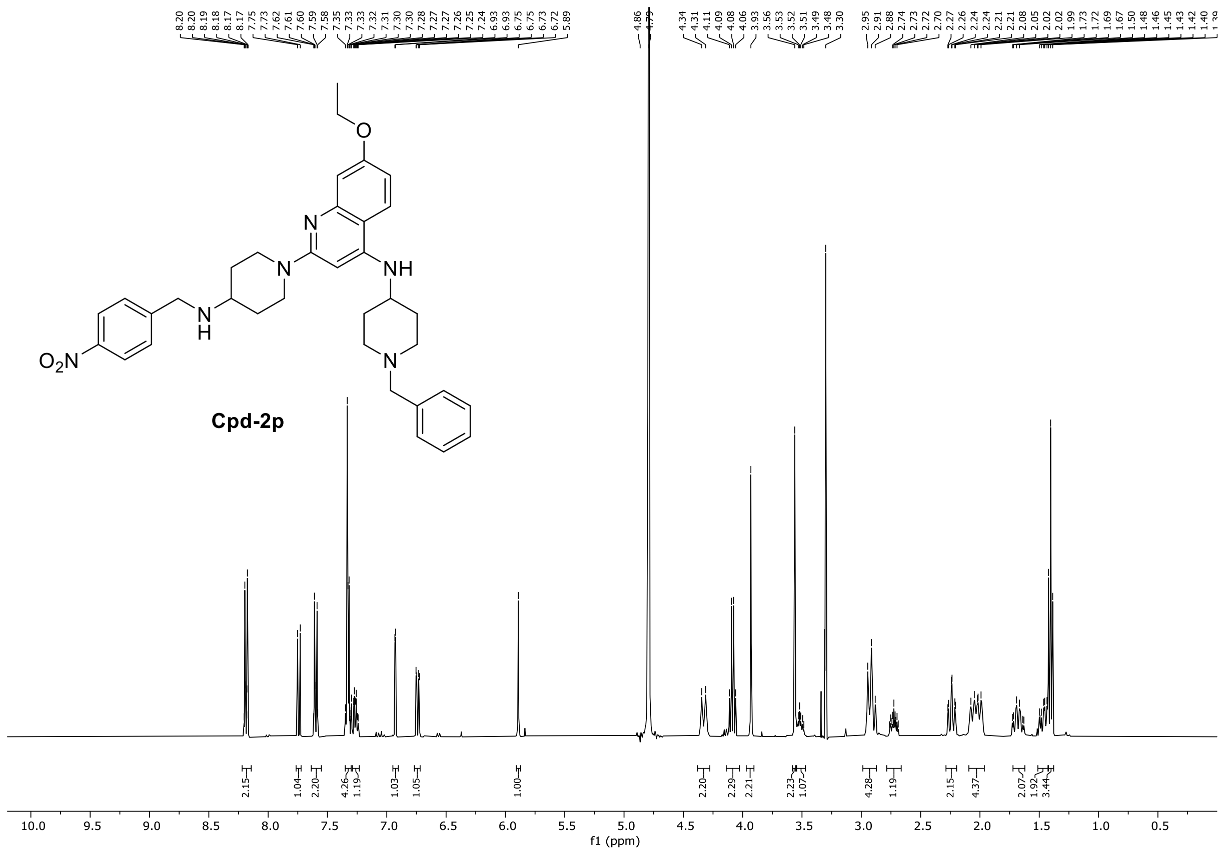

Figure S107: ^{1}H NMR spectrum of Compound **2p**.

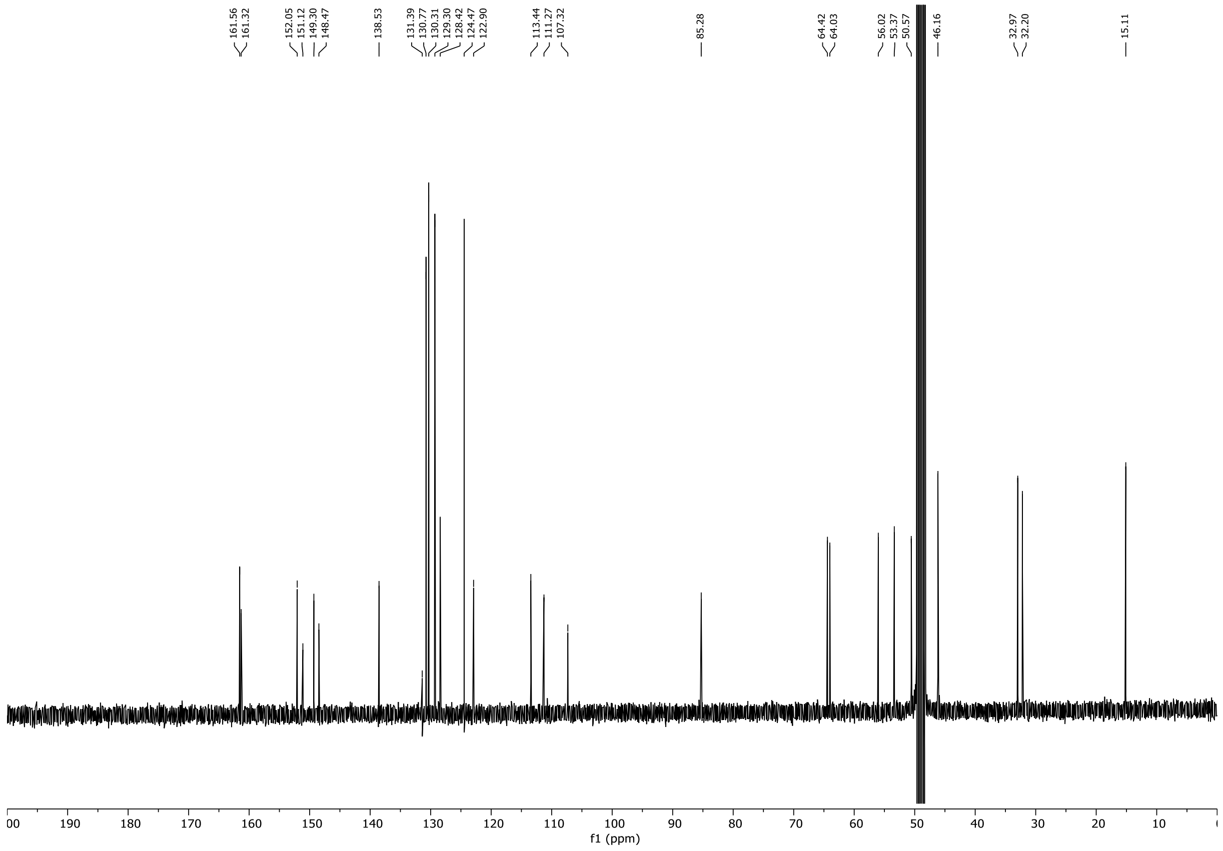

Figure S108: ^{13}C NMR spectrum of Compound **2p**.

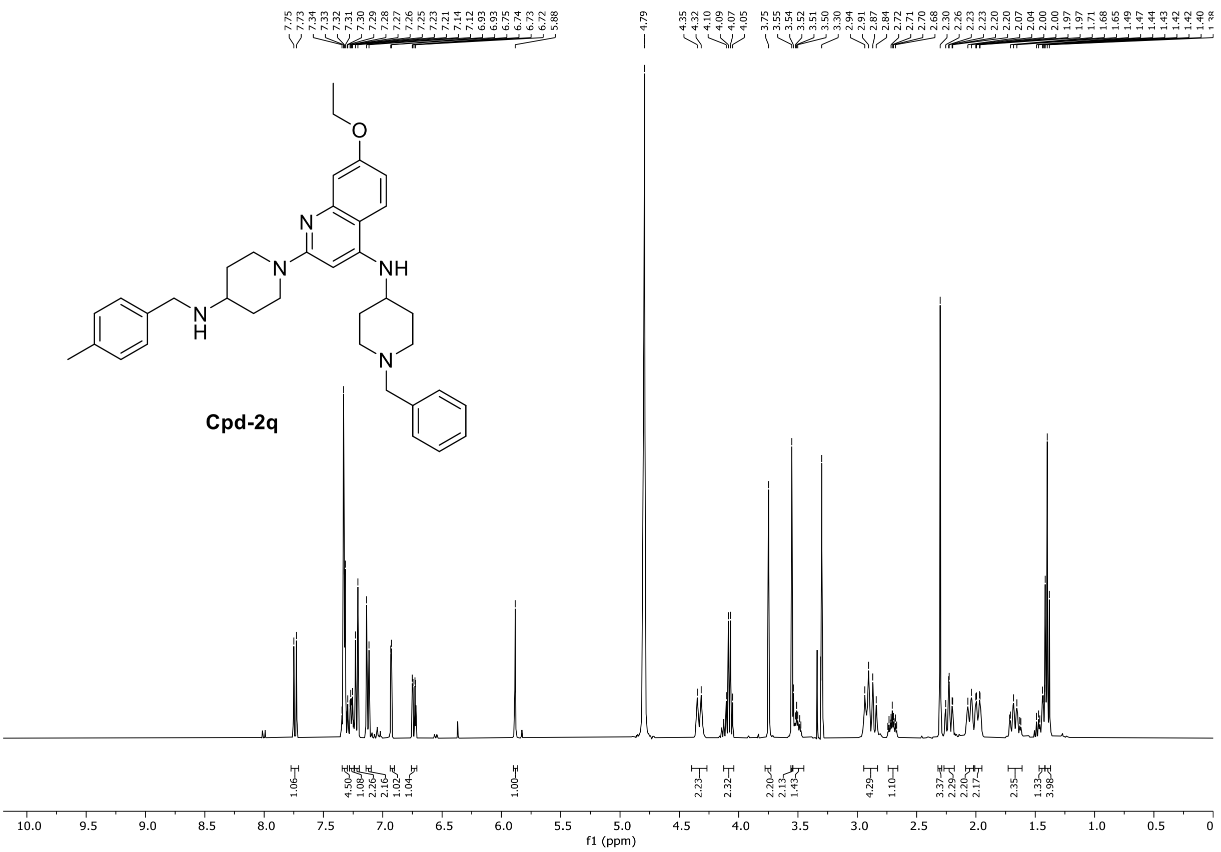

Figure S109: ^{1}H NMR spectrum of Compound **2q**.

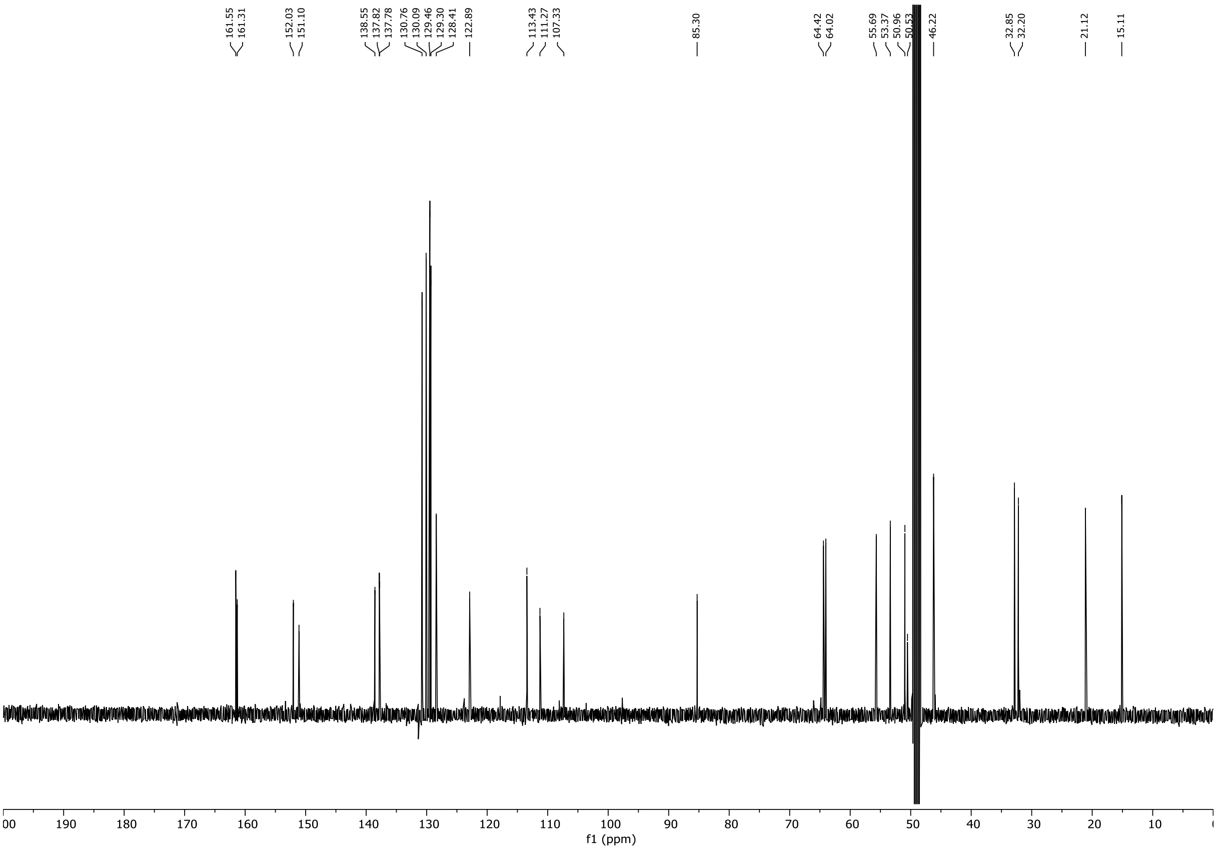

Figure S110: ^{13}C NMR spectrum of Compound **2q**.

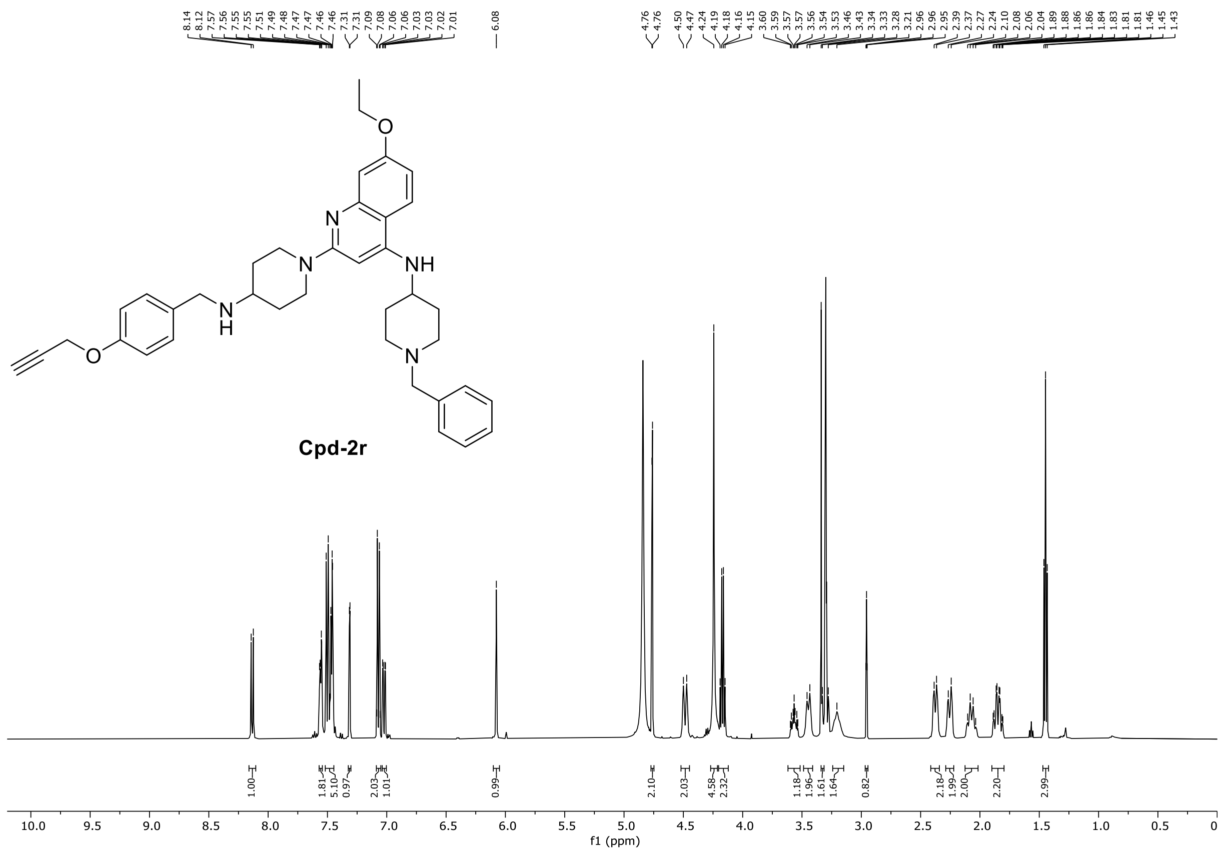

Figure S111: ^{1}H NMR spectrum of Compound **2r**.

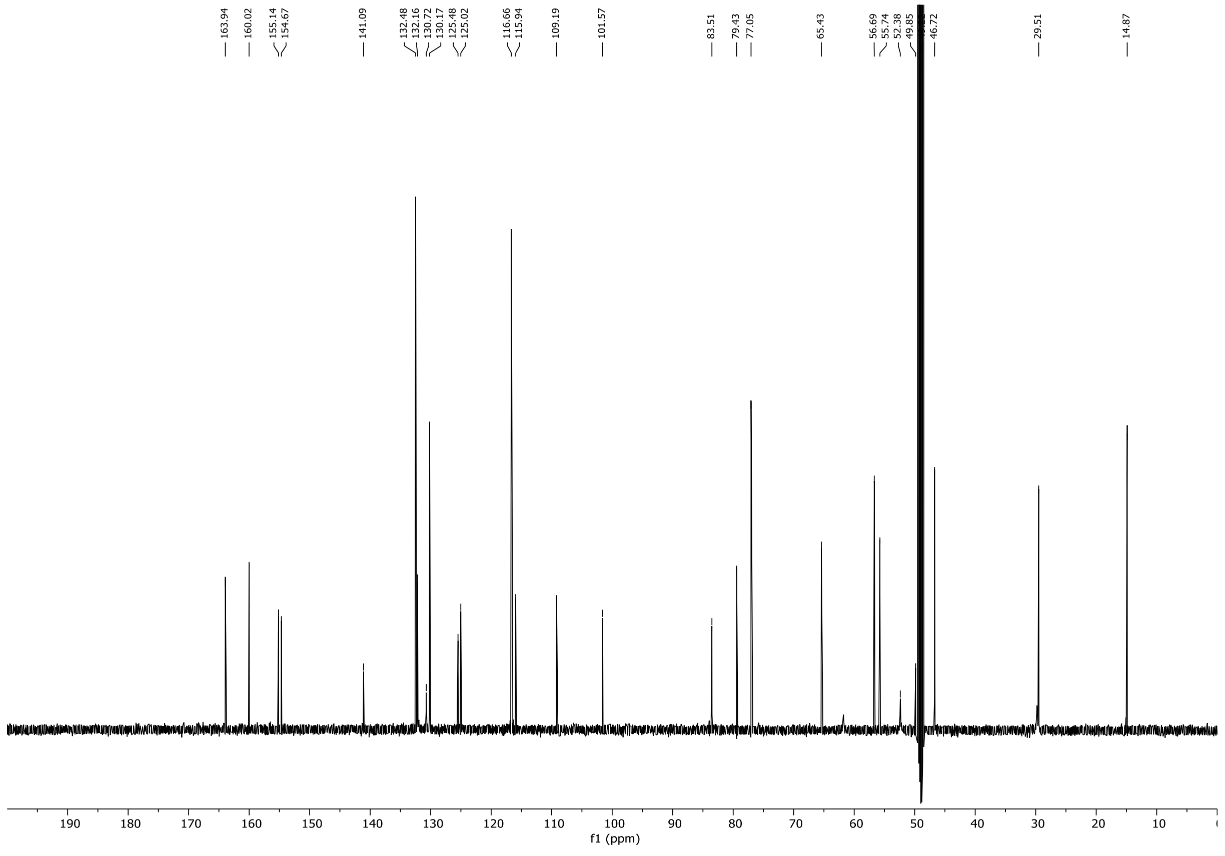

Figure S112: ^{13}C NMR spectrum of Compound **2r**.

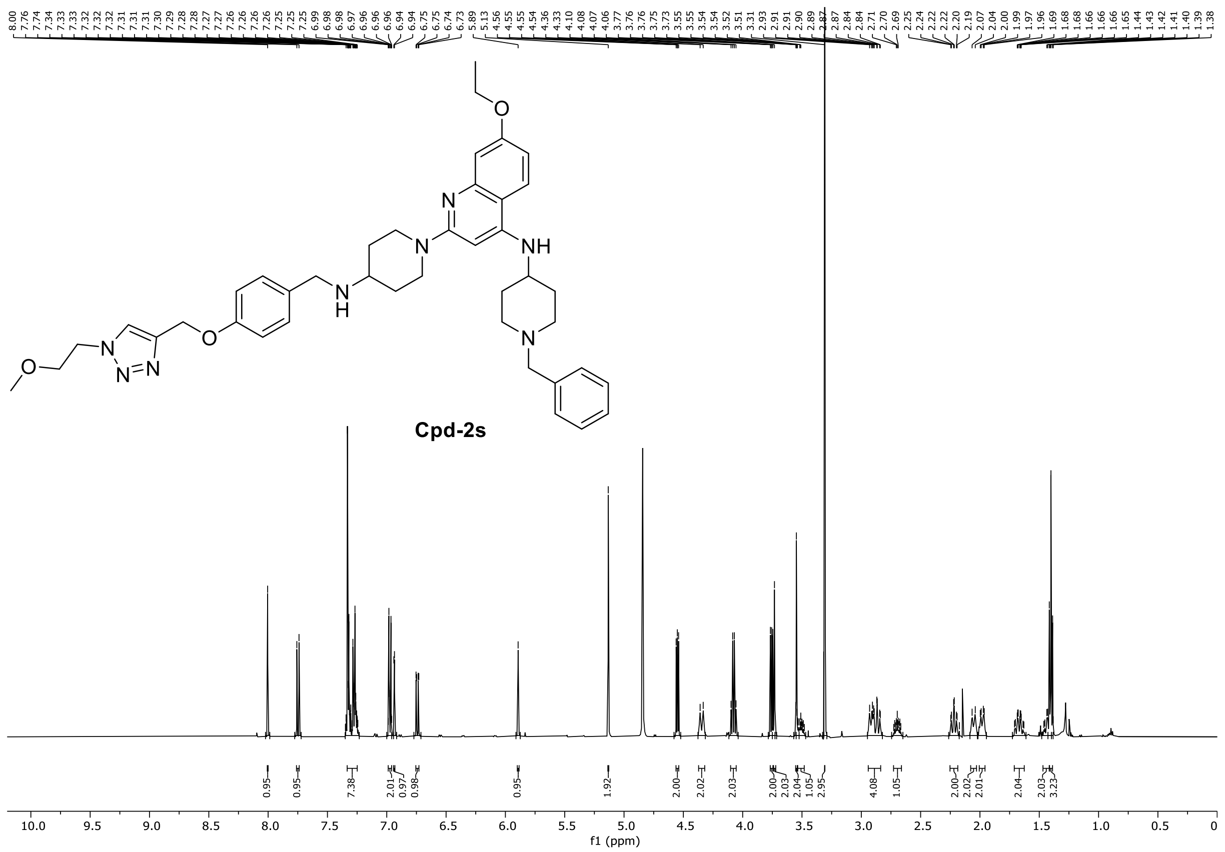

Figure 1: ^{1}H NMR spectrum of Compound **2s**.

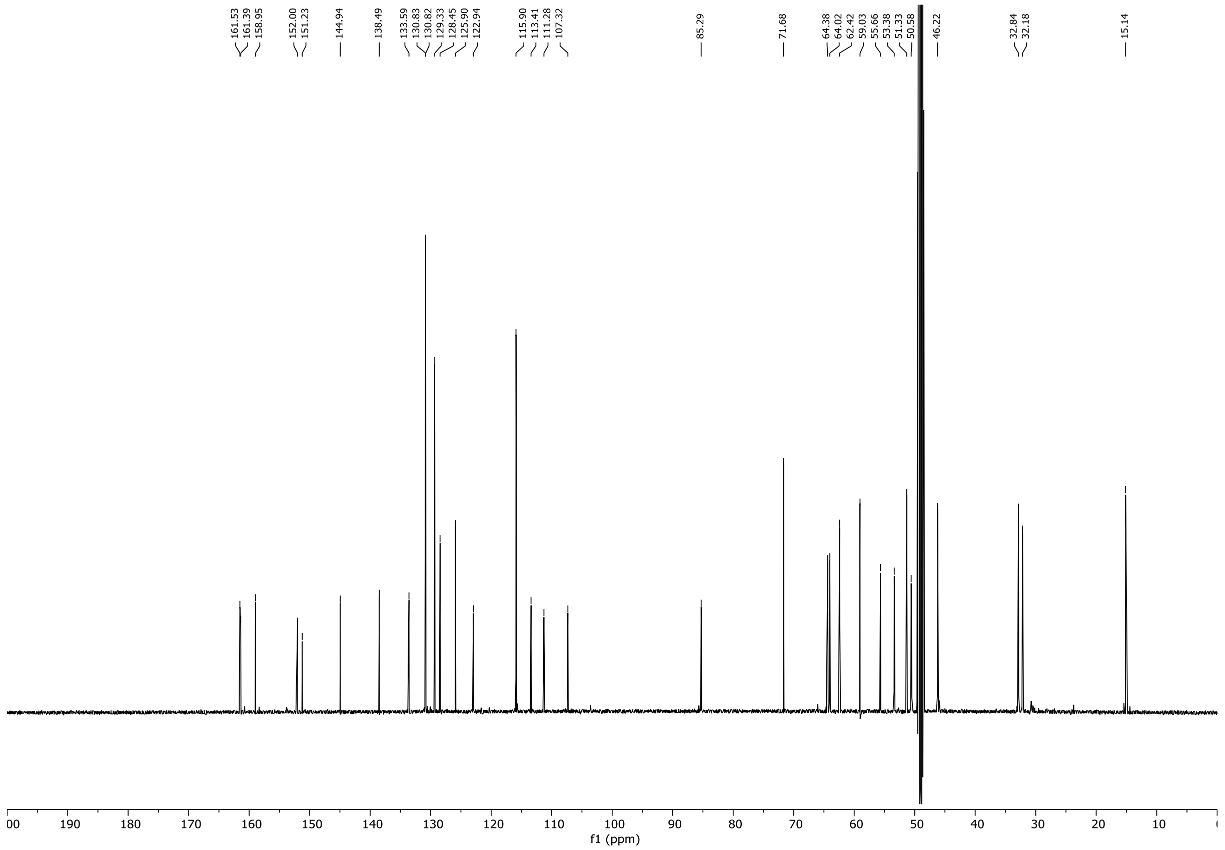

Figure S114: ^{13}C NMR spectrum of Compound **2s**.

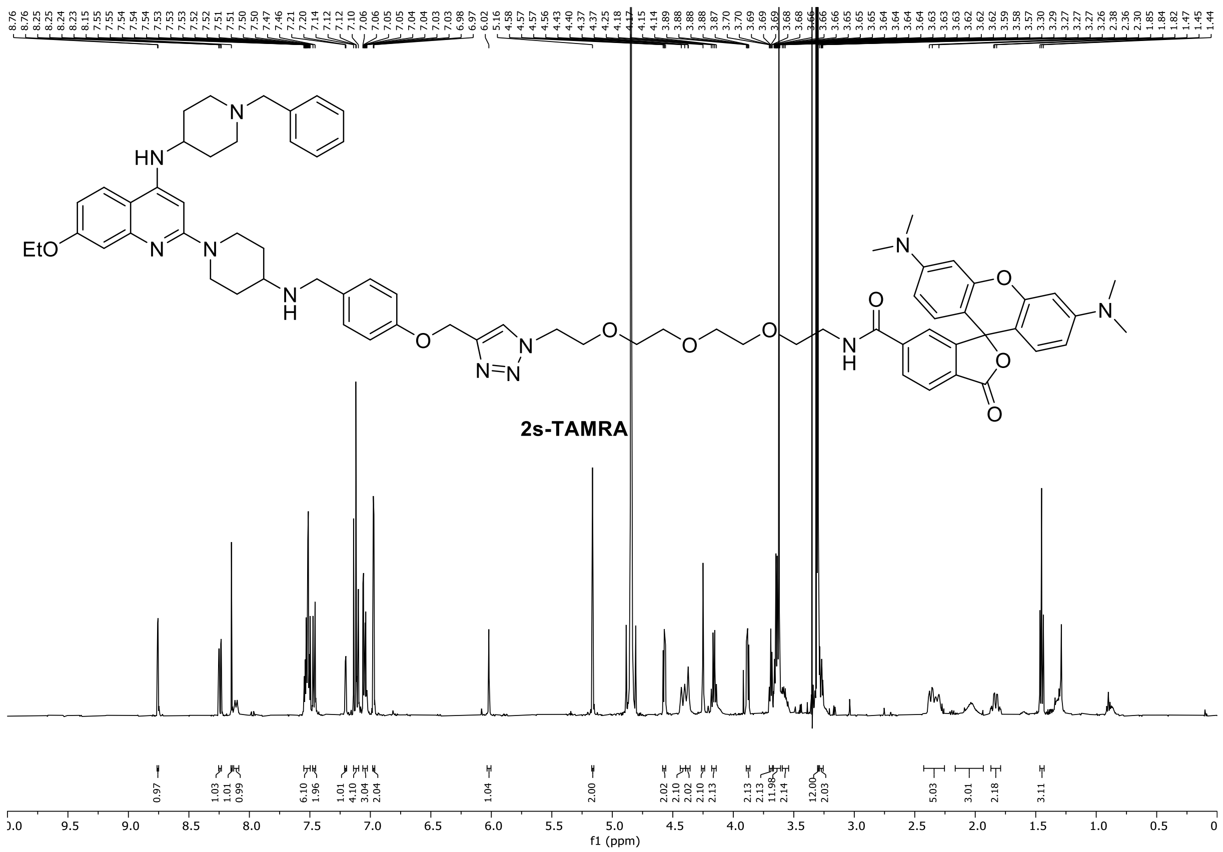

Figure S115: ^{1}H NMR spectrum of Compound **2s-TAMRA**.

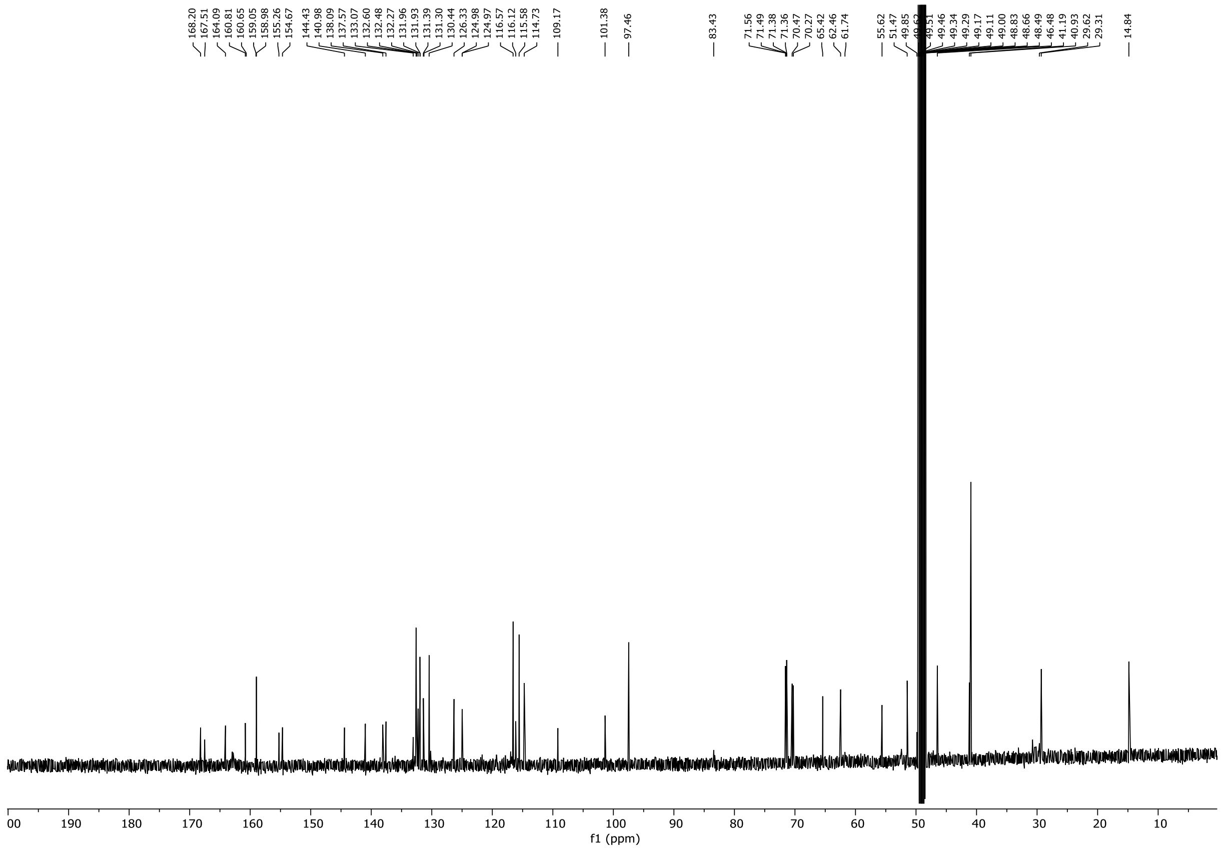

Figure S116: ^{13}C NMR spectrum of Compound **2s-TAMRA**.
